# Nuclear factor, erythroid 2-like 2-associated molecular signature predicts lung cancer survival

**DOI:** 10.1038/srep16889

**Published:** 2015-11-24

**Authors:** Zhongqing Qian, Tong Zhou, Christopher I. Gurguis, Xiaoyan Xu, Qing Wen, Jingzhu Lv, Fang Fang, Louise Hecker, Anne E. Cress, Viswanathan Natarajan, Jeffrey R. Jacobson, Donna D. Zhang, Joe G. N. Garcia, Ting Wang

**Affiliations:** 1 Key Laboratory of Anhui Province for Infection and Immunology, Bengbu Medical College, Bengbu 233003, China; 2 Arizona Respiratory Center and Department of Medicine, The University of Arizona, Tucson, Arizona, USA; 3 Department of Pharmacy, Jinan Central Hospital, Jinan, Shandong, China; 4 Department of Pharmacology and Toxicology, The University of Arizona, Tucson, Arizona, USA; 5 Department of Biochemistry and Molecular Biology, Bengbu Medical College, Bengbu 233003, China; 6 Southern Arizona VA Health Care System, Tucson, AZ 85723, USA; 7 Arizona Cancer Center and Department of Cellular and Molecular Medicine, University of Arizona, Tucson, AZ, USA; 8 Department of Pharmacology, The University of Illinois at Chicago, Chicago, IL, USA; 9 Department of Medicine, The University of Illinois at Chicago, Chicago, IL, USA

## Abstract

Nuclear factor, erythroid 2-like 2 (*NFE2L2*), a transcription factor also known as NF-E2-related factor 2 (Nrf2), is a key cytoprotective gene that regulates critical antioxidant and stress-responsive genes. Nrf2 has been demonstrated to be a promising therapeutic target and useful biomarker in malignant disease. We hypothesized that *NFE2L2*-mediated gene expression would reflect cancer severity and progression. We conducted a meta-analysis of microarray data for 240 *NFE2L2*-mediated genes that were enriched in tumor tissues. We then developed a risk scoring system based on *NFE2L2* gene expression profiling and designated 50 tumor-associated genes as the *NFE2L2*-associated molecular signature (NAMS). We tested the relationship between this gene expression signature and both recurrence-free survival and overall survival in lung cancer patients. We find that NAMS predicts clinical outcome in the training cohort and in 12 out of 20 validation cohorts. Cox proportional hazard regressions indicate that NAMS is a robust prognostic gene signature, independent of other clinical and pathological factors including patient age, gender, smoking, gene alteration, MYC level, and cancer stage. NAMS is an excellent predictor of recurrence-free survival and overall survival in human lung cancer. This gene signature represents a promising prognostic biomarker in human lung cancer.

Nuclear factor, erythroid 2-like 2 (*NFE2L2*), also known as NF-E2-Related Factor 2 (Nrf2), is a transcription factor encoded by the *NFE2L2* gene in humans^1^. Nrf2 regulates the transcription of a wide array of genes that code for antioxidants and other proteins responsible for the detoxification of xenobiotics and reactive oxygen species, including a battery of phase II enzymes, such as NAD(P)H quinone oxidoreductase 1 (Nqo1), Heme oxygenase-1 (HO-1), and glutathione S-transferase (GST)^2^. Under physiological conditions, cytosolic Nrf2 protein is maintained at very low levels by its selective negative regulator, Kelch-like ECH-associated protein 1 (KEAP1), a cytoplasmic protein which sequesters Nrf2 in the cytoplasm and directs it to CUL3 E3 ligase for ubiquitylation and subsequent degradation by the proteasome^3^,^4^. Under conditions of oxidative stress or in the presence of Nrf2 activating compounds, E3 activity is compromised and Nrf2 is stabilized, with increasing the amount of Nrf2 relative to Keap1^2^. Free Nrf2 moves to the nucleus, thereby activating expression of its downstream antioxidant genes^3^,^5^,^6^.

As a central regulator of antioxidant genes, *NFE2L2* has received great attention for its pivotal role in a number of pathologic conditions, including cancer^7^,^8^,^9^,^10^. Increasingly, data have demonstrated that *NFE2L2* is over expressed in a great number of solid and hematologic tumors 11,12,13,14,15,16. Many cancer cells have been found to exhibit increased expression and activity of Nrf2^17^. In addition, *NFE2L2* is highly up-regulated in various types of tumors and the prognosis of patients with tumors expressing high levels of *NFE2L2* is poor^18^.

As *NFE2L2* becomes dysregulated, cells may acquire several traits, including proliferation, apoptosis resistance, and a profound resistance to drugs and radiotherapy, which may promote tumor growth and cancer pathogenesis^19^. Nrf2 activators have been used in clinical trials for cancer therapy and the treatment of diseases associated with oxidative stress; on the other hand, constitutive activation of Nrf2 in many types of tumors contributes to the survival and growth of cancer cells, as well as resistance to anticancer therapy^20^. Therefore, manipulation of Nrf2 has become of great interest with regard to its use in therapy and the diagnosis/prognosis of malignant diseases.

In this present study, we conducted secondary analysis of genome-wide expression data to identify *NFE2L2*-associated genes implicated in cancer pathobiology. Because of its strong association with cancer, we hypothesized that *NFE2L2*-mediated genes could be used to indicate cancer progression. Specifically, we aimed to assess whether genes associated with *NFE2L2* could be used as a prognostic tool for lung cancer patients. We found that an *NFE2L2*-mediated gene signature could effectively predict lung cancer survival.

## Results

### NFE2L2 influences gene expression in human lung cancer

**Figure 1:**
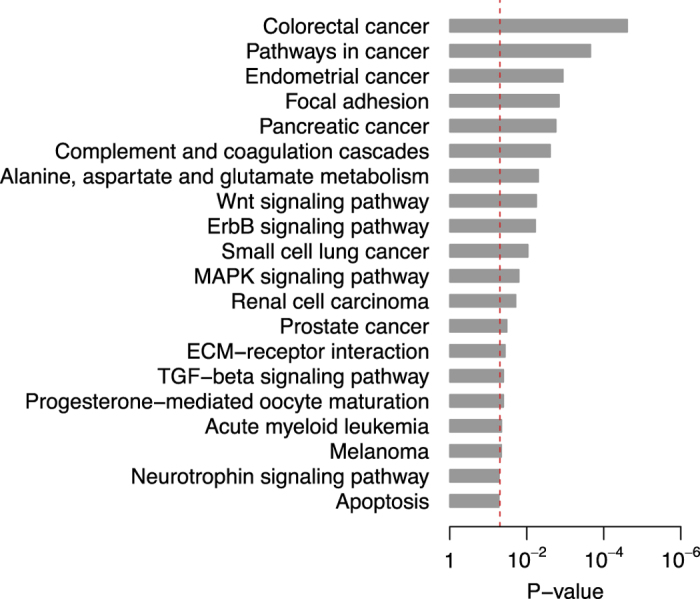
The top 20 KEGG pathways enriched in the *NFE2L2*-mediated genes in lung cancer cell. The *P*-values were calculated by Fisher’s exact test. The red dash line denotes the significance level of α = 0.05.

We compared the gene expression patterns of control cells to those of *NFE2L2* knockdown (KD) human lung cancer cells to identify genes potentially regulated by *NFE2L2*. One microarray dataset (GSE38332) containing gene expression information from both control and *NFE2L2* KD A549 lung cancer cells^21^ was downloaded from the Gene Expression Omnibus (GEO) database^22^. At the specified significance level of false discovery rate (FDR) <0.01 and fold change >2 (see Methods for details), 1631 probe sets encoding 1172 genes were found to be up-regulated in *NFE2L2* KD cells, while 792 probe sets for 593 genes were down-regulated in *NFE2L2* KD cells (Supplementary Table S1). We next searched the enriched Kyoto Encyclopedia of Genes and Genomes (KEGG)^23^ physiological pathways among the dysregulated genes revealing genes enriched in cancer-related KEGG terms (9 out of 20 significantly dysregulated pathways), such as “Colorectal cancer”, “Pathways in cancer”, “Endometrial cancer”, “Pancreatic cancer”, “Small cell Lung cancer”, “Renal cell carcinoma”, and “Prostate cancer” (Fig. 1). To address whether the *NFE2L2*-mediated genes (1765 differentially expressed genes) are statistically significantly associated with KEGG cancer pathways, we conducted a resampling test. We obtained 1,000 random gene sets by randomly selecting 1765 genes from human genome. For each random gene set, we counted the number of KEGG cancer pathways that are significantly associated with the random gene set. We found that the number of KEGG cancer pathways of the *NFE2L2*-mediated genes is significantly larger than that of the random gene sets (*P* = 0.002) (Supplementary Fig. S1). The results suggest that the *NFE2L2*-mediated genes are involved in human lung cancer pathology.

To explore the role of *NFE2L2*-mediated genes in human lung cancers, we compared the gene expression between the paired normal and tumor tissues in two lung cancer cohorts from Spain (GSE18842, ESP)^24^ and Taiwan (GSE19804, TWN)^25^. In total, 1695 probe sets encoding 1284 genes were found to be commonly differentially expressed (see Methods for details) between normal and tumor tissues in the two cohorts (Supplementary Table S2). Among these probe sets/genes, 299 probe sets encoding 240 genes were found to overlap with the *NFE2L2*-mediated genes (Supplementary Table S3), which is statistically significant (*P* = 2.2 × 10^−14^ by hypergeometric cumulative distribution function) and, thus, suggests that *NFE2L2*-mediated genes are significantly enriched among the lung cancer-related genes. The top KEGG pathways associated with the 240 overlapped genes includes “ECM-receptor interaction”, “Pathways in cancer”, “Complement and coagulation cascades”, “Jak-STAT signaling pathway”, “Focal adhesion”, “Small cell lung cancer”, and “TGF-beta signaling pathway” (Supplementary Fig. S2), which suggests the depth of involvement of *NFE2L2*-mediated genes in human cancer. Univariate Cox proportional hazards regression was applied to evaluate the relationship between lung cancer outcome and gene expression for the 240 genes. The association between recurrence-free survival and gene expression was computed in a lung cancer training cohort from Korea (GSE8894, KOR)^26^. We also calculated the association between overall survival and gene expression in another training cohort from the United States (GSE3141, USA)^27^. The Wald statistic (ratio of Cox regression coefficient to its standard error) of the 240 genes in the KOR cohort (*Z*_*R*_) is positively correlated with that in the USA cohort (*Z*_*D*_) (Supplementary Fig. S3). Among the 240 genes, we identified 50 genes with both |*Z*_*R*_| > 0.5 and |*Z*_*D*_| > 0.5. We hypothesized that the expression of the 50 genes would predict lung cancer outcome. We designated these genes as the *NFE2L2-*Associated Molecular Signature (NAMS) (Table 1 and Supplementary Fig. S4 and S5). The NAMS gene set (50 genes) is significantly associated with two KEGG pathways: ECM-receptor interaction and focal adhesion (Supplementary Fig. S6).

### NAMS predicts recurrence-free survival in lung cancer

**Figure 2:**
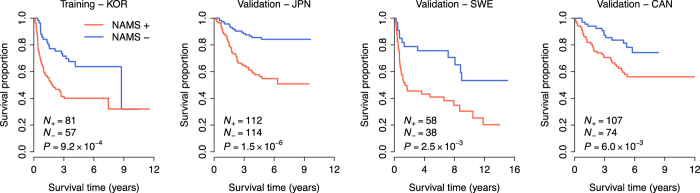
Kaplan-Meier curves of recurrence-free survival. The expression of the NAMS predicts poor recurrence-free survival in the discovery (KOR) and validation (JPN, SWE, and CAN) cohorts. The red curves are for the NAMS positive patients while the blue curves are for the NAMS negative patients. The NAMS positive patients were defined as those having a recurrence risk score greater than zero. P-values were calculated by log-rank tests for the differences in survival between the NAMS positive and negative groups.

To confirm that the NAMS would be predictive of tumor outcome in lung cancer, we constructed a scoring system to assign each patient a recurrence-risk score, representing a linear combination of the NAMS gene expression values weighted by the coefficients obtained from the training cohort (KOR) (see Methods for details). NAMS_R_^+^ patients were defined as those having recurrence-risk scores greater than zero, and the other patients were assigned as NAMS_R_^−^. As expected, we found a significantly reduced recurrence-free survival for the NAMS_R_^+^ patients in the training cohort (Fig. 2 and Table 2).

We next tested the prognostic power of the NAMS based recurrence-risk score in three validation cohorts from Japan (GSE31210, JPN)^28^, Sweden (GSE37745, SWE)^29^, and Canada (GSE50081, CAN)^30^. Kaplan-Meier survival curves demonstrated a significantly reduced recurrence-free survival for NAMS_R_^+^ patients in the three validation cohorts (log-rank test: *P* = 1.5 × 10^−6^ for JPN, *P* = 2.5 × 10^−3^ for SWE, and *P* = 6.0 × 10^−3^ for CAN) (Fig. 2). Univariate Cox proportional hazards regression indicated that NAMS_R_^+^ patients had an increased risk for recurrence of 3.59-, 2.65-, and 2.36-fold in the JPN, SWE, and CAN cohorts, respectively (Table 2). These findings suggest that the NAMS is predictive of recurrence-free survival in lung cancer.

In addition, we checked prognostic power of NAMS on different types of lung cancer (including adenocarcinoma, squamous cell carcinoma, and large cell carcinoma). JPN cohort are all Significantly reduced recurrence-free survival for NAMS_R_^+^ adenocarcinoma patients (*P* = 4.7 × 10^−4^), but not squamous cell carcinoma patients, was found in the CAN cohort (Supplementary Fig. S7). In the SWE cohort, significantly reduced recurrence-free survival for NAMS_R_^+^ squamous cell carcinoma patients (*P* = 2.2 × 10^−2^), but not the other two types, was observed (Supplementary Fig. S8). Regardless of statistical significance (mainly due to the limitation of the same size), all recurrence-free survival data in all types of cancers remain at the same trend that NAMS_R_^+^ patients have reduced survival.

### NAMS predicts overall survival in lung cancer

**Figure 3:**
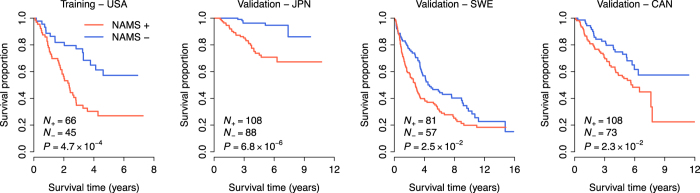
Kaplan-Meier curves of overall survival. The expression of the NAMS predicts poor overall survival in the discovery (USA) and validation (JPN, SWE, and CAN) cohorts. The red curves are for the NAMS positive patients while the blue curves are for the NAMS negative patients. The NAMS positive patients were defined as those having a death risk score greater than zero. P-values were calculated by log-rank tests for the differences in survival between the NAMS positive and negative groups.

We also tested the power of the NAMS in predicting overall survival in lung cancer. The USA cohort was used for training. Similarly, a scoring system was developed to assign each patient a death-risk score, calculated as a linear combination of the NAMS gene expression values weighted by the coefficients obtained from the USA cohort (see Methods for details). NAMS_D_^+^ patients were defined as those having death-risk scores greater than zero, while the other patients were NAMS_D_^−^. Kaplan-Meier survival curves demonstrated a significantly reduced overall survival for the NAMS_D_^+^ patients in both the training and validation cohorts (Fig. 3). Univariate Cox proportional hazards regression indicated that the NAMS_D_^+^ patients had an increased risk for death of 2.71-, 5.92-, 1.46-, and 1.76-fold in the USA, JPN, SWE, and CAN cohorts, respectively (Table 2). These results suggest that NAMS can be used to predict overall survival in lung cancer.

Same as the recurrence-free survival study, we examined the prognostic power of NAMS on subtypes of lung cancer on overall survival. Significantly reduced recurrence-free survival for NAMS_R_^+^ adenocarcinoma patients (*P* = 7.7 × 10^−3^), but not squamous cell carcinoma patients, was found in CAN cohort (Supplementary Fig. S9). In SWE cohort, NAMS fails to differentiate overall survival significantly in three subtypes of lung cancer (Supplementary Fig. S10). Same as recurrence-free survival study, all overall survival data in all lung cancer subtypes remain at the same trend that NAMS_R_^+^ patients have reduced survival, regardless of statistical significance.

### The prognostic power of NAMS is non-random

**Figure 4:**
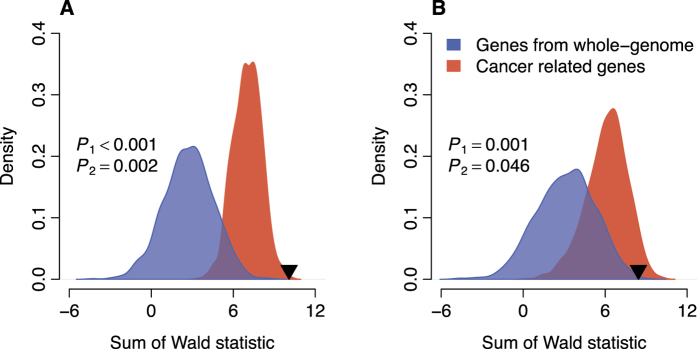
Non-random prognostic power of NAMS in lung cancer. The blue areas show the distributions of the sum of Wald statistic for the 1,000 resampled gene signatures picked up from whole human genome with identical size as NAMS. The red areas show the distributions of the sum of Wald statistic for the 1,000 resampled gene signatures picked up from the cancer related genes with identical size as NAMS. The black triangles stand for the sum of Wald statistic of NAMS. (**A**) Resampling pattern for recurrence-free survival; (**B**) Resampling pattern for overall survival. *P*_1_: the right-tailed P-values for the resampling test when the resampled gene signatures were picked up from whole human genome; *P*_2_: the right-tailed *P*-values for the resampling test when the resampled gene signatures were picked up from the cancer related genes. Figure S10. Kaplan-Meier curves of overall survival on lung cancer subtypes (SWE).

A computational study indicated that the prognostic power of most published gene signatures in breast cancer are not significantly better than that of random signatures with identical size^31^. Here, we performed a resampling test to check whether the NAMS performed better than random gene signatures. We constructed 1,000 random gene signatures with identical size as the NAMS (50 genes). Cox proportional hazards regression of survival was conducted for each resampled gene signature. The association between each random gene signature and recurrence-free/overall survival was measured by the sum of Wald statistic in the three validation cohorts. Our alternative hypothesis was that the sum of the Wald statistic of the NAMS would be more positive than expected by chance if the prognostic power of the NAMS was significantly better than that of random gene signatures. We found that the sum of the Wald statistic of the NAMS was significantly larger than that of randomized gene signatures (*P* < 0.001 for recurrence-free survival and *P* = 0.001 for overall survival) (Fig. 4).

We also compared the prognostic power of the NAMS against the other cancer related genes. Here, we defined the cancer related genes as those being differentially expressed between normal and tumor lung tissues (listed in Supplementary Table S2) and with |*Z*_*R*_| > 0.5 or |*Z*_*D*_| > 0.5. We performed a resampling test to check whether the prognostic power of NAMS was statistically better than the other cancer related genes. For each round of randomization, 50 genes were picked up from the pool of the cancer related genes. The performance of the random gene set was quantified by the sum of the Wald statistic of the validation cohorts. The prognostic power of the NAMS was significantly better than that of 1000 random gene signatures consisting of cancer related genes (*P* = 0.002 for recurrence-free survival and *P* = 0.046 for overall survival) (Fig. 4).

### Independence of NAMS from the traditional clinical and pathological factors

To confirm the strength of the NAMS as an independent predictor, we investigated the performance of the NAMS in comparison with the traditional clinical and pathological variables associated with prognosis in lung cancers. For the JPN cohort, we considered factors including age, gender, smoking history, stage, *EGFR*/*KRAS*/*ALK* mutation status (Gene alteration), and MYC level. For the SWE cohort, we included age, gender, and stage as covariate. For the CAN cohort, age, gender, smoking history, and stage were taken into account. Multivariate Cox proportional hazards regression of recurrence-free survival indicated that NAMS remained a significant covariate in relation to the clinical and pathological factors in all the validation cohorts (Table 3). Interestingly, NAMS was the most significant (lowest *P*-value) covariate in each cohort for recurrence-free survival (Table 3). We also conducted multivariate Cox proportional hazards regression against overall survival. We found that NAMS was still a significant covariate in the JPN and SWE cohorts (Table 3). However, the *P*-value of NAMS was slightly above the α-level of 0.05 (*P* = 0.075) in the CAN cohort (Table 3). Because the covariates differed among each cohort, we also repeated these analyses using the same three covariates (age, gender, and stage) for the three cohorts. These results did not dramatically change our interpretation (reported in Supplementary Table S4). Taken together, the NAMS is a survival predictor in cancer patients, independent of the traditional clinical and pathological factors.

## Discussion

Nrf2 is a transcription factor that acts as a master regulator for the expression of a wide array of anti-oxidant genes^32^. Up-regulation of *NFE2L2* by chemopreventive compounds confer protection against cancer initiation^15^,^16^. Several studies have indicated that dysregulation of *NFE2L2* is strongly associated with human cancer^7^,^8^. Though the full extent to which *NFE2L2* is involved in tumorigenesis is not known, genetic analyses have shown that *NFE2L2* has increased mutations or has been deregulated in human cancers^12^,^33^,^34^. The role of *NFE2L2* in pulmonary neoplasia, a diverse disease for which few biomarkers exist, is complicated and appears to depend on several factors, including the existence of activating mutations in *NFE2L2* and/or loss of function mutations in *KEAP1*^35^. Through a computational genomics approach, our current study confirmed a central role for *NFE2L2* in lung cancer. The complex functions of Nrf2 in carcinogenesis and chemotherapy resistance require more detailed characterization and mechanistic analyses, however, our data reveals some interesting patterns, in light of current knowledge about lung malignancy.

Lung cancer is the most frequently diagnosed cancers and leading cause of cancer death in males, comprising 17% of the total new cancer cases and 23% of the total cancer deaths^36^. With the rapid advances taking place in molecular testing and associated technologies, the landscape of targetable genomic alterations in lung cancer is beginning to uncover the true complexity of the disease^37^. In this study, we explored the prognostic value of those gene sets regulated by *NFE2L2* in lung cancer. First, we compared genes that were differentially expressed between lung cancer and *NFE2L2*-knockdown A549 cells. Over 2425 genes were found to be commonly differentially expressed between WT and *NFE2L2*-knockdown cells. We confirmed the critical role of *NFE2L2* in carcinogenesis by the gene ontology analysis of all *NFE2L2*-mediated genes: 12 of 20 significantly deregulated pathways are direct cancer pathways (Fig. 1). Second, we generated the *NFE2L2*-associated Molecular Signature (NAMS) by filtering gene express data sets through different cohorts. Third, we validated the NAMS as a powerful tool which provides important prognostic predictions in lung cancer, as we demonstrated that the NAMS was a significant and independent predictor of recurrence-free cancer survival. Although limited by the availability of the existing lung cancer microarray datasets deposited in GEO, the consistent findings from the population with diverse genetic background in discovery and validation cohorts (e.g., USA verses KOR) strongly suggest the NAMS in lung cancer is powerful and highly conserved molecular signature among populations. Finally, these NAMS predictions were independent of other known clinical pathological covariates in this study. This last result is especially intriguing given recent developments in the way that clinicians and cancer biologists have begun to think about the cancer disease process.

Although the origin of cancer is still hotly debated^38^, cancer is unequivocally a quantitative trait^39^. Our findings are consistent with a modern understanding of carcinogenesis as involving not only a somatic evolutionary process^40^,^41^, but also contributions from the tumor microenvironment^42^,^43^, as well as general dysregulation at the tissue level^44^. In fact, the 50 gene set that comprised our NAMS itself highlights that cancer is a complex trait with many gene pathways involved (a fact that seems to be of broad significance, see also^45^,^46^). These pathway-of-cancer enriched genes provide a set of *NFE2L2*-associated genes that might play critical roles in cancer pathogenesis. For example, RRM2 plays an important role in regulating expression of the anti-apoptotic protein Bcl-2 and reveal a critical link between RRM2 and Bcl-2 in apoptosis signaling and tumor developing^47^. Recent studies showed that concomitant low expression levels of RRM2 was predictive of a better outcome, and low expression of RRM2 could be used to predict the treatment response to platinum-based chemotherapy and survival in lung cancer^48^,^49^.

This study reinforces the value of re-examining available genomic/genetic data in the “big data” era with a novel translational approach. *NFE2L2* is confirmed to be a novel “oncogene” (broadly construed) with a central role in carcinogenesis. More work on *NFE2L2* will be necessary to fully elucidate the mechanisms underlying this pattern. In addition to cancer prognosis, now well validated in the current study, *NFE2L2* holds promise for the management of multiple cancers. We confirm that the NAMS represents a promising prognostic biomarker in human lung cancer predicting recurrence-free survival and validation survival.

## Methods

### Gene expression profiling

We obtained the gene expression data in control and *NFE2L2* KD A549 lung cancer cells from the Gene Expression Omnibus (GEO) database (GSE38332)^21^. Seven independent microarray lung cancer datasets from Spain (GSE18842, ESP)^24^, Taiwan (GSE19804, TWN)^25^, Korea (GSE8894, KOR)^26^, the United States (GSE3141, USA)^27^, Japan (GSE31210, JPN)^28^, Sweden (GSE37745, SWE)^29^, and Canada (GSE50081, CAN)^30^, were also downloaded from the GEO database (Supplementary Table S5). Expression data of paired normal and tumor tissues from lung cancer patients were available in the ESP and TWN cohorts. The information on recurrence-free survival was available in the KOR, JPN, SWE, and CAN cohorts. The information on overall survival was available for the USA, JPN, SWE, and CAN cohorts. The ESP and TWN cohorts were used to identify the differentially expressed genes between normal and tumor lung tissues. The KOR and USA cohorts were used for training purpose for recurrence-free and overall survival, respectively. The JPN, SWE, and CAN datasets were used as validation cohorts.

### Microarray data analysis

We used the GCRMA algorithm in Bioconductor to normalize the expression level of each probe set for the microarray data of the control and *NFE2L2* KD lung cancer cells and of the paired normal and tumor tissues. Only the probe sets with unique annotations were included in this study. The genes on chromosomes X and Y were excluded to avoid the potentially confounding factor. The significance analysis of microarrays (SAM) algorithm^50^, implemented in the samr library of the R Statistical Package, was used to compare log2-transformed gene expression levels between the control and *NFE2L2* KD cells. FDR was controlled using the q-value method^51^. The probe sets with a fold-change >2 and FDR <0.01 were deemed differentially expressed between the control and *NFE2L2* KD cells. For the ESP and TWN cohorts, paired t-test was used to identify the genes differentially expressed between normal and tumor tissues. The *P*-values were adjusted by Benjamini-Hochberg correction. Only the probe sets with a fold-change >2 and adjusted *P* < 0.01 were deemed differentially expressed between normal and tumor tissues.

### Risk score

For the two training cohorts (KOR and USA), univariate Cox proportional hazards regression was used to evaluate the association between recurrence-free survival/overall survival and gene expression. A recurrence-risk score and a death-risk score were then calculated for each patient, respectively, using a linear combination of gene expression weighted by the Wald statistic (ratio of regression coefficient to its standard error) as shown below:


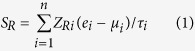



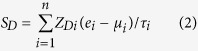


Here, *S*_*R*_ (Formula 1) and *S*_*D*_ (Formula 2) are the recurrence-risk and death-risk scores, respectively; *n* is the number of differentially expressed genes; *Z*_*Ri*_ denotes the Wald statistic of recurrence-free survival for the *i*^th^ gene, which was derived from the KOR cohort; Z_*Di*_ denotes the Wald statistic of overall survival for the *i*^th^ gene, which was derived from the USA cohort; *e*_*i*_ denotes the expression level of gene *i*; and *μ*_*i*_ and *τ*_*i*_ are the mean and standard deviation of the gene expression values for gene *i* across all samples, respectively. A higher risk score implies a poor outcome.

## Additional Information

**How to cite this article**: Qian, Z. *et al.* Nuclear factor, erythroid 2-like 2-associated molecular signature predicts lung cancer survival. *Sci. Rep.* **5**, 16889; doi: 10.1038/srep16889 (2015).

## Supplementary Material

Supplementary Information

## Figures and Tables

**Table 1:** NAMS gene set.

Gene symbol	Gene title
*ABCA8*	ATP-binding cassette, sub-family A (ABC1), member 8
*ABI3BP*	ABI family, member 3 (NESH) binding protein
*ADAM12*	ADAM metallopeptidase domain 12
*ADRB1*	adrenoceptor beta 1
*ANGPT1*	angiopoietin 1
*ANKRD29*	ankyrin repeat domain 29
*ANKRD44*	ankyrin repeat domain 44
*ATL3*	atlastin GTPase 3
*BCHE*	butyrylcholinesterase
*C15orf48*	chromosome 15 open reading frame 48
*COL3A1*	collagen, type III, alpha 1
*COL5A1*	collagen, type V, alpha 1
*DEPDC7*	DEP domain containing 7
*EGLN3*	egl nine homolog 3 (C. elegans)
*EHF*	ets homologous factor
*GALNT7*	UDP-N-acetyl-alpha-D-galactosamine:polypeptide N-acetylgalactosaminyltransferase 7 (GalNAc-T7)
*GPX3*	glutathione peroxidase 3 (plasma)
*HLA-E*	major histocompatibility complex, class I, E
*IPO4*	importin 4
*ITGB4*	integrin, beta 4
*LIFR*	leukemia inhibitory factor receptor alpha
*MARC2*	mitochondrial amidoxime reducing component 2
*MED20*	mediator complex subunit 20
*METTL7A*	methyltransferase like 7A
*NCALD*	neurocalcin delta
*PCM1*	pericentriolar material 1
*PLAU*	plasminogen activator, urokinase
*PLCB4*	phospholipase C, beta 4
*PLEKHH2*	pleckstrin homology domain containing, family H (with MyTH4 domain) member 2
*RECK*	reversion-inducing-cysteine-rich protein with kazal motifs
*RGCC*	regulator of cell cycle
*RILPL2*	Rab interacting lysosomal protein-like 2
*RRM2*	ribonucleotide reductase M2
*SEC14L4*	SEC14-like 4 (S. cerevisiae)
*SERPINH1*	serpin peptidase inhibitor, clade H (heat shock protein 47), member 1, (collagen binding protein 1)
*SFN*	stratifin
*SLIT3*	slit homolog 3 (Drosophila)
*SPP1*	secreted phosphoprotein 1
*TACC1*	transforming, acidic coiled-coil containing protein 1
*TBX2*	T-box 2
*TNS1*	tensin 1
*TOM1L2*	target of myb1-like 2 (chicken)
*TPPP*	tubulin polymerization promoting protein
*TSPAN5*	tetraspanin 5
*TTYH3*	tweety homolog 3 (Drosophila)
*TXNL1*	thioredoxin-like 1
*VARS*	valyl-tRNA synthetase
*VCAN*	versican
*VSIG10*	V-set and immunoglobulin domain containing 10
*ZNF25*	zinc finger protein 25

**Table 2:** Cox proportional hazards regression of survival by NAMS status.

	Recurrence-free survival				Overall survival			
	Cohort	HR	95% CI	*P*-value	Cohort	HR	95% CI	*P*-value
Training	KOR	2.35	(1.40, 3.96)	1.3 × 10^−3^	USA	2.71	(1.52, 4.85)	7.8 × 10^−4^
Validation	JPN	3.59	(2.06, 6.25)	6.7 × 10^−6^	JPN	5.92	(2.46, 14.28)	7.5 × 10^−5^
	SWE	2.65	(1.38, 5.12)	3.6 × 10^−3^	SWE	1.46	(1.05, 2.03)	2.6 × 10^−2^
	CAN	2.36	(1.25, 4.42)	7.7 × 10^−3^	CAN	1.76	(1.08, 2.87)	2.5 × 10^−2^

Note – HR: hazard ratio; CI: confidence interval.

**Table 3:** Multivariate Cox proportional hazards regression of survival in the validation cohorts.

Cohort	Covariate	Recurrence-free survival			Overall survival		
		HR	95% CI	*P*-value	HR	95% CI	*P*-value
JPN	NAMS + vs. -	2.84	(1.58, 5.12)	5.1 × 10^−4^	4.45	(1.76, 11.25)	1.6 × 10^−3^
	Age (per year)	1.04	(1.01, 1.08)	2.3 × 10^−2^	1.04	(0.99, 1.09)	1.5 × 10^−1^
	Gender male vs. female	0.90	(0.45, 1.79)	7.6 × 10^−1^	0.92	(0.36, 2.34)	8.6 × 10^−1^
	Smoking + vs. -	1.02	(0.51, 2.03)	9.6 × 10^−1^	1.06	(0.42, 2.69)	9.0 × × 10^−1^
	Stage (I and II)	2.43	(1.42, 4.14)	1.1 × 10^−3^	2.79	(1.38, 5.64)	4.3 × 10^−3^
	Gene alteration + vs. -	0.61	(0.37, 1.03)	6.4 × 10^−2^	0.53	(0.27, 1.07)	7.5 × 10^−2^
	MYC level high vs. low	1.06	(0.42, 2.71)	9.0 × 10^−1^	0.68	(0.16, 2.87)	6.0 × 10^−1^
SWE	NAMS + vs. -	2.72	(1.32, 5.61)	6.6 × 10^−3^	1.43	(1.01, 2.04)	4.5 × 10^−2^
	Age (per year)	1.00	(0.96, 1.03)	8.7 × 10^−1^	1.03	(1.01, 1.05)	5.7 × 10^−3^
	Gender male vs. female	0.80	(0.43, 1.48)	4.8 × 10^−1^	0.98	(0.69, 1.38)	9.1 × 10^−1^
	Stage (I-IV)	1.11	(0.75, 1.63)	6.1 × 10^−1^	1.24	(1.02, 1.50)	3.3 × 10^−2^
CAN	NAMS + vs. -	2.60	(1.33, 5.07)	5.1 × 10^−3^	1.65	(0.95, 2.85)	7.5 × 10^−2^
	Age (per year)	1.00	(0.97, 1.03)	9.6 × 10^−1^	1.01	(0.99, 1.04)	2.9 × 10^−1^
	Gender male vs. female	1.71	(0.94, 3.13)	8.1 × 10^−2^	1.89	(1.11, 3.24)	2.0 × 10^−2^
	Smoking + vs. -	0.43	(0.21, 0.90)	2.6 × 10^−2^	0.90	(0.42, 1.95)	7.9 × 10^−1^
	Stage (I and II)	1.90	(1.06, 3.39)	3.0 × 10^−2^	1.93	(1.15, 3.23)	1.2 × 10^−2^

Note – HR: hazard ratio; CI: confidence interval.
