# Supplementary material for: Nuclear factor, erythroid 2-like 2-associated molecular signature predicts lung cancer survival
Source: Sci Rep. 2015 Nov 24;5:16889. doi: 10.1038/srep16889 (PMC4657037; doi:10.1038/srep16889)
Supplement: Supplementary Information [file srep16889-s1.pdf]

Supplementary Figures and Tables

Nuclear factor, erythroid 2-like 2-associated molecular signature predicts lung cancer survival

Zhongqing Qian[1,2†], Tong Zhou[2†], Christopher I. Gurguis[2], Xiaoyan Xu[2], Qing Wen[3,4], Jingzhu Lv[5], Fang Fang[1], Louise Hecker[2,6], Anne E. Cress[7], Viswanathan Natarajan[8,9], Jeffrey R. Jacobson[9], Donna D. Zhang[4], Joe G. N. Garcia[2], Ting Wang*[2]

[1] Key Laboratory of Anhui Province for Infection and Immunology, Bengbu Medical College, Bengbu 233003, China

[2] Arizona Respiratory Center and Department of Medicine, The University of Arizona, Tucson, Arizona, USA

[3] Department of Pharmacy, Jinan Central Hospital, Jinan, Shandong, China

[4] Department of Pharmacology and Toxicology, The University of Arizona, Tucson, Arizona, USA

[5] Department of Biochemistry and Molecular Biology, Bengbu Medical College, Bengbu 233003, China

[6] Southern Arizona VA Health Care System, Tucson, AZ 85723

[7] Arizona Cancer Center and Department of Cellular and Molecular Medicine, University of Arizona, Tucson, AZ, USA

[8] Department of Pharmacology, The University of Illinois at Chicago, Chicago, IL, USA

[9] Department of Medicine, The University of Illinois at Chicago, Chicago, IL, USA

[†] These authors contributed equally to this work.

Corresponding author:
*Ting Wang, PhD, Email: twang@email.arizona.edu; Tel: (520) 626-4472

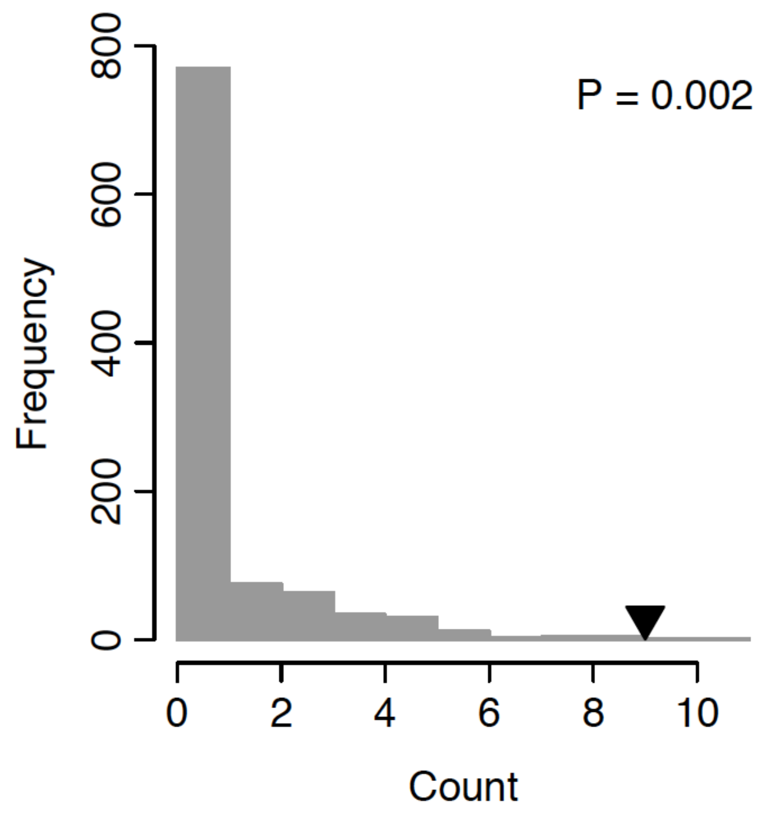

Figure S1. *NFE2L2*-mediated genes are significantly enriched in cancer pathways. 1,000 gene sets were randomly picked up from human genome with identical size as the *NFE2L2*-mediated genes. The histogram shows the distribution of the number of KEGG cancer pathways that were significantly associated the resampled gene sets were recorded. The black triangle stands for the number of KEGG cancer pathways associated the *NFE2L2*-mediated genes.

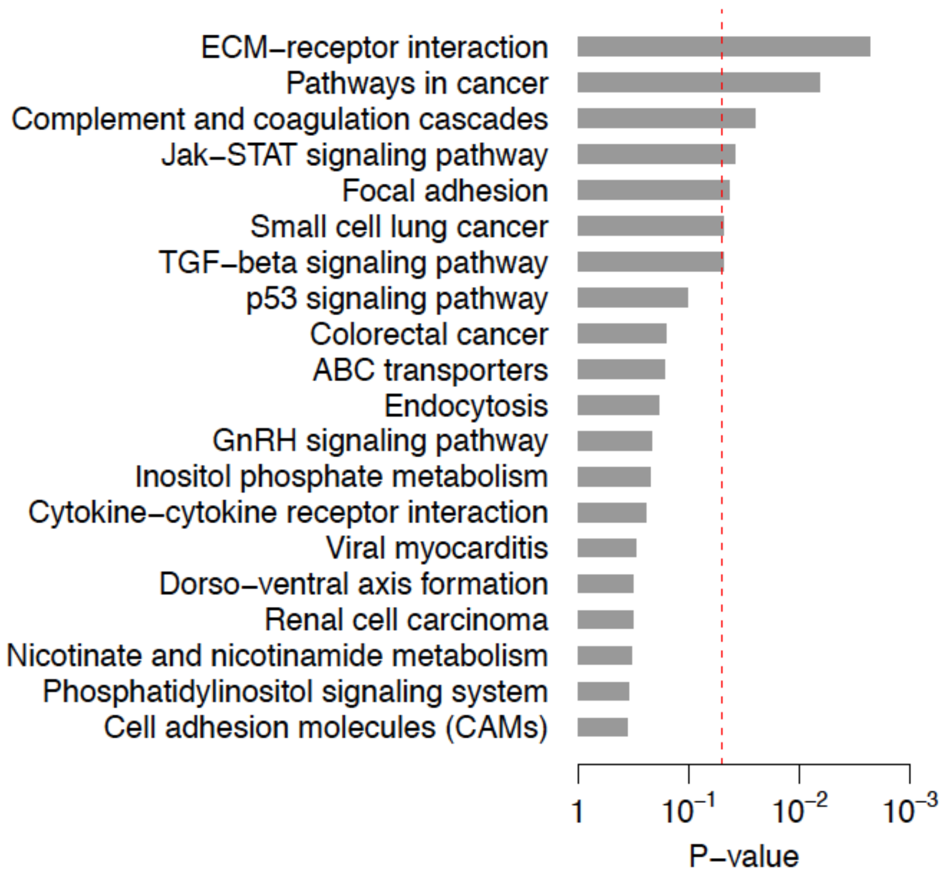

Figure S2. The pathways enriched in the genes that are mediated by *NFE2L2* and differentially expressed between normal and tumor tissues. The P-values were calculated by Fisher's exact test. The red dash line denotes the significance level of α = 0:05.

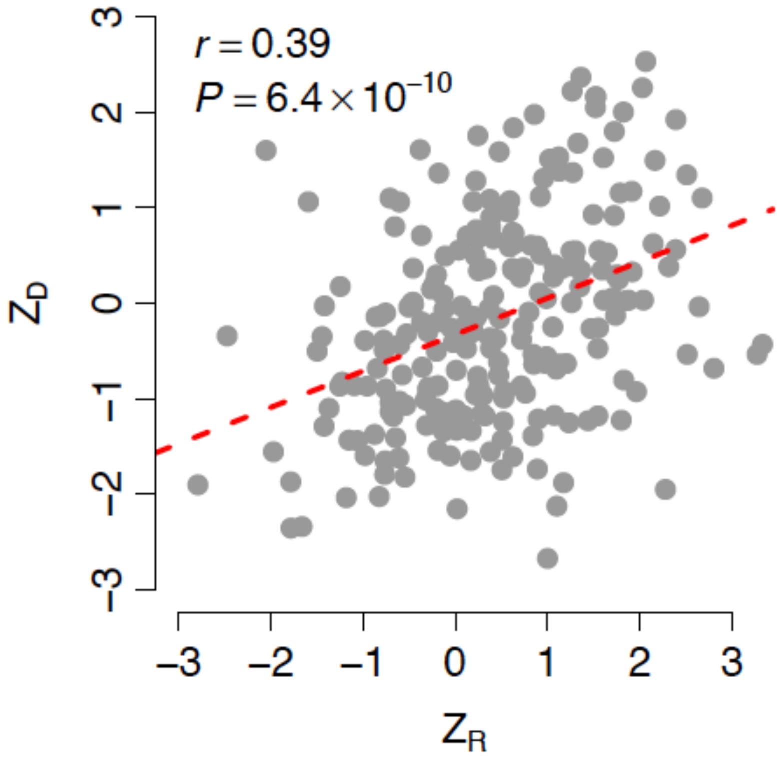

Figure S3. Correlation between the Wald statistic in the KOR and USA cohorts. Each dot denotes one gene. Z_R is the Wald statistic computed by univariate Cox proportional hazards regression between recurrence-free survival and gene expression. Z_D is the Wald statistic computed by univariate Cox proportional hazards regression between overall survival and gene expression. Pearson correlation test indicates that Z_R is significantly correlated with Z_D.

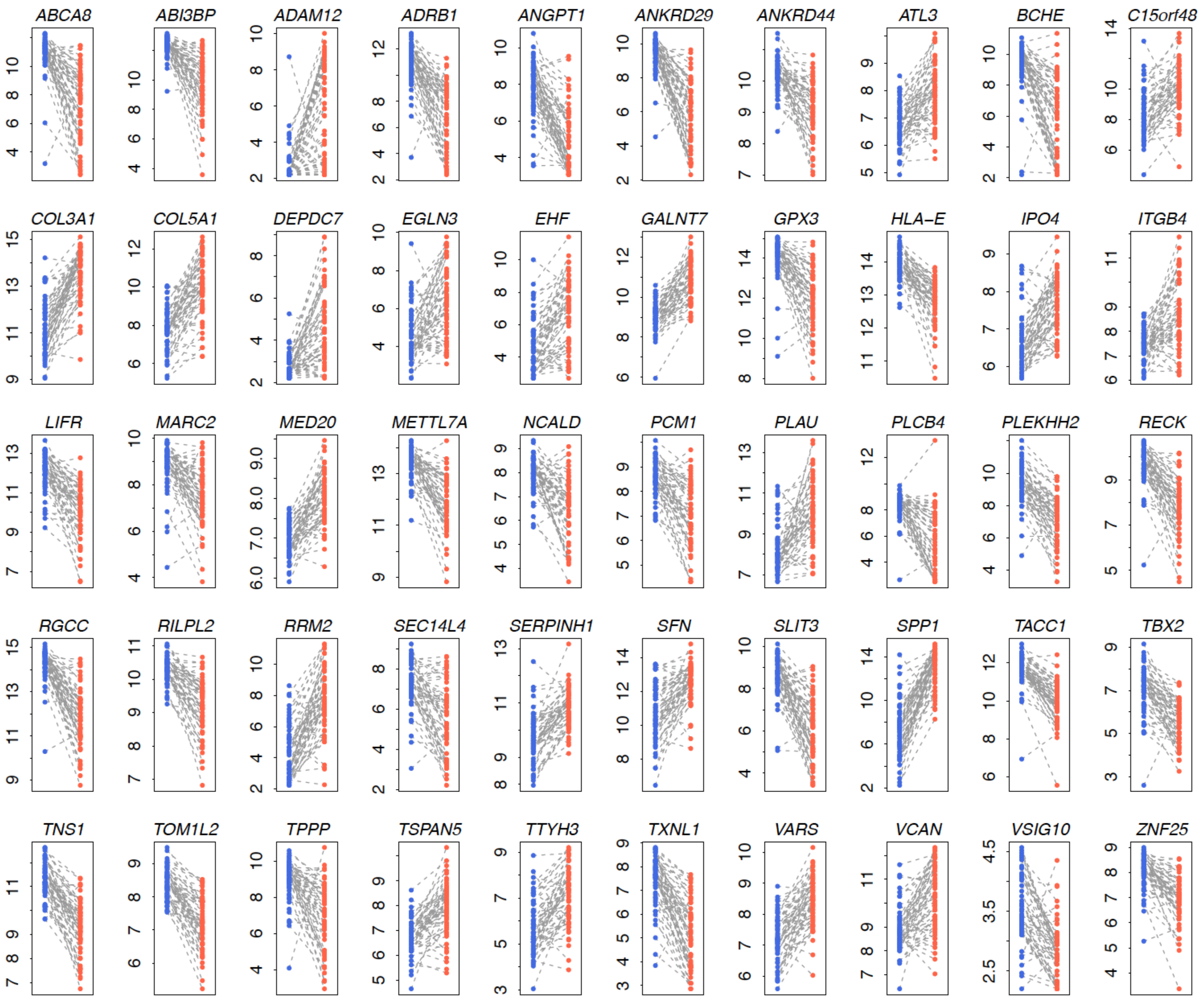

Figure S4. Comparison of NAM expression between the normal and tumor tissues in the TWN cohort. Paired normal and tumor tissues from 60 lung cancer patients were included in the comparison. The blue dots denote the normal tissues while the red ones stand for the tumor tissues. Y-axis: $\log_2$-transformed expression values.

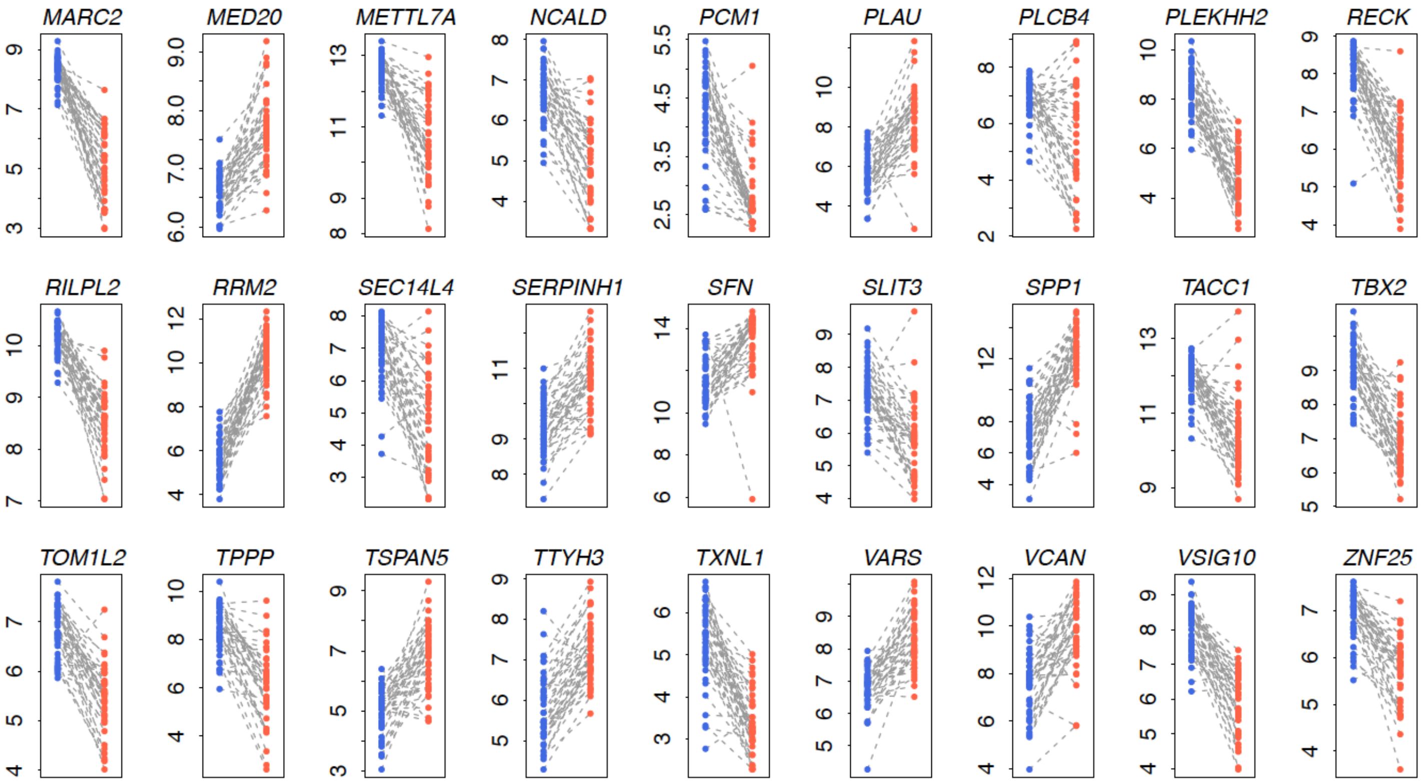

Figure S5. Comparison of NAM expression between the normal and tumor tissues in the ESP cohort. Paired normal and tumor tissues from 44 lung cancer patients were included in the comparison. The blue dots denote the normal tissues while the red ones stand for the tumor tissues. Y-axis: log_2-transformed expression values.

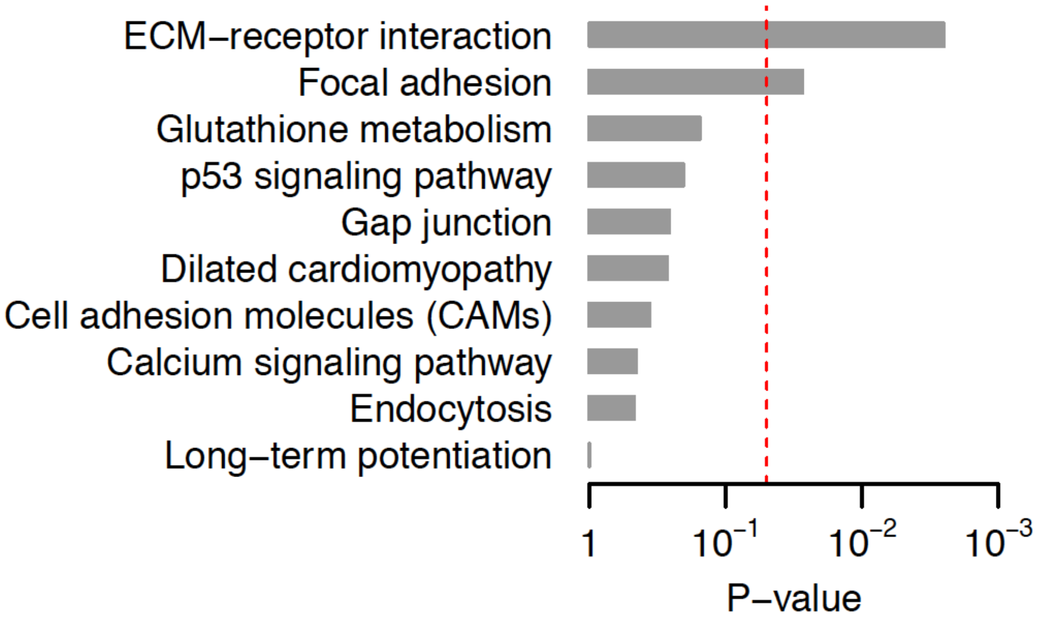

Figure S6. The top KEGG pathways enriched in the NAMS genes (50 genes). The *P*-values were calculated by Fisher's exact test. The red dash line denotes the significance level of $\alpha = 0.05$.

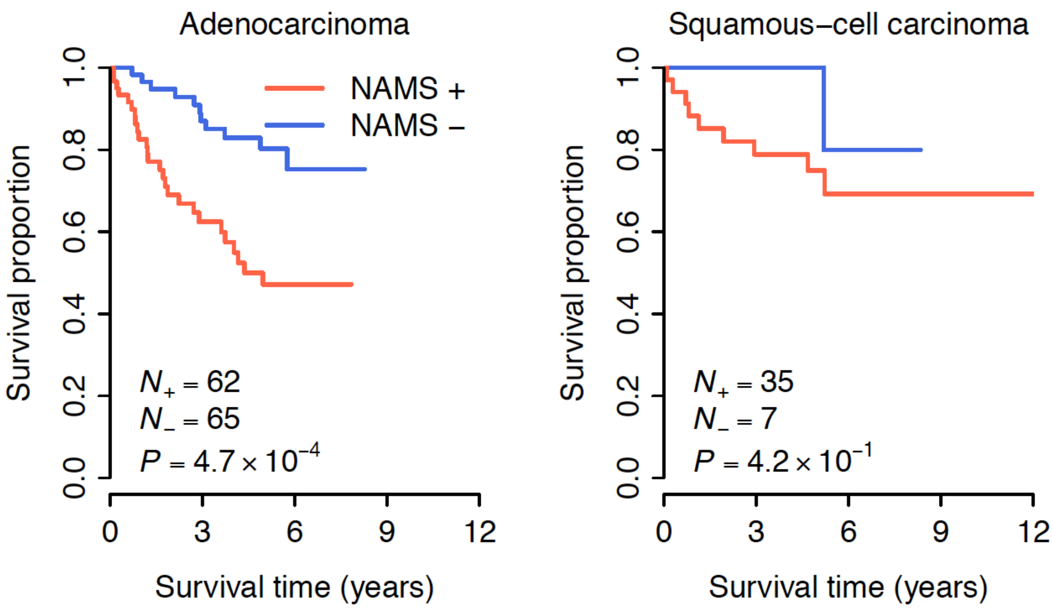

Figure S7. Kaplan-Meier curves of recurrence-free survival on lung cancer subtypes (CAN).

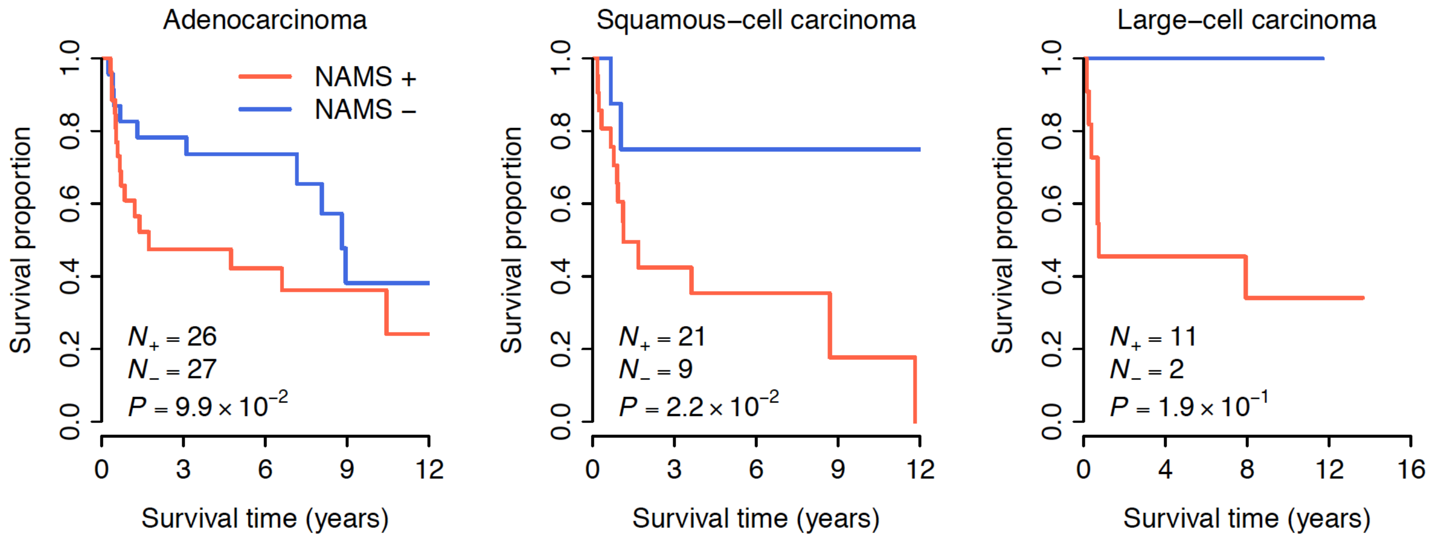

Figure S8. Kaplan-Meier curves of recurrence-free survival on lung cancer subtypes (SWE).

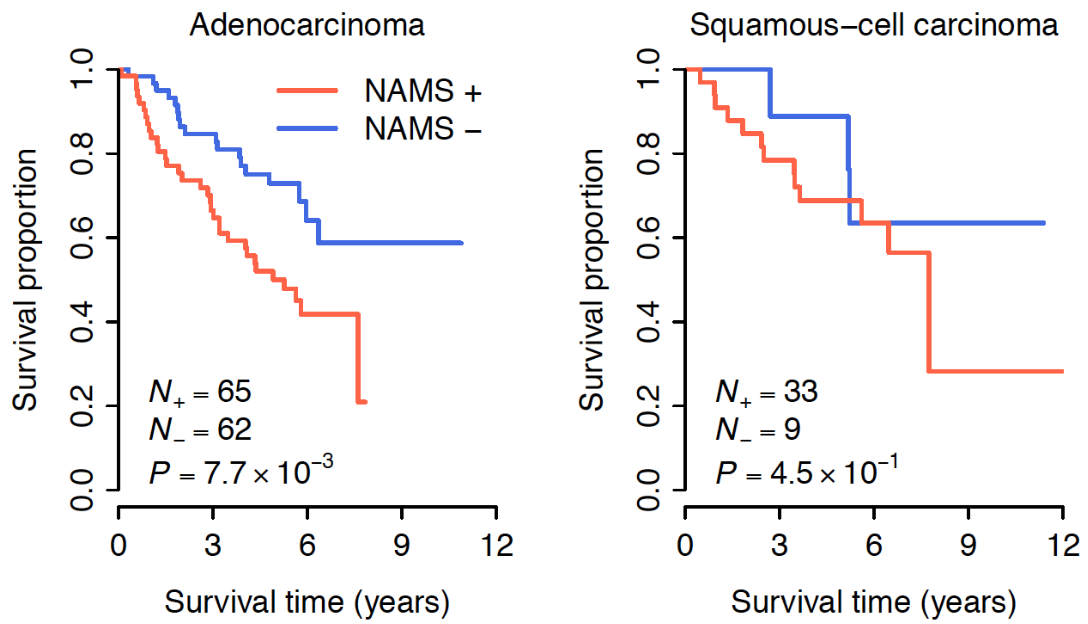

Figure S9. Kaplan-Meier curves of overall survival on lung cancer subtypes (CAN).

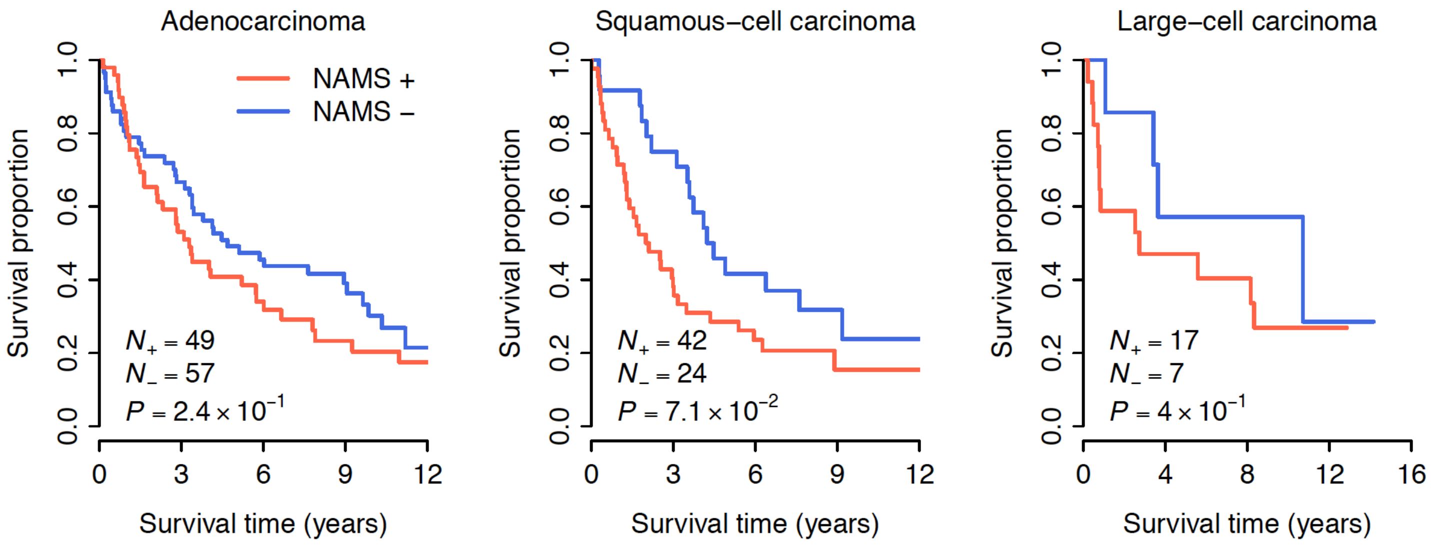

Figure S10. Kaplan-Meier curves of overall survival on lung cancer subtypes (SWE).

Table S1: Differentially expressed genes between control and NFE2L2 knockdown cells.

Probeset ID	Gene title	Gene symbol	Fold change	FDR (%)
213664_at	solute carrier family 1 (neuronal/epithelial high affinity glutamate transporter, system Xag), member 1	SLC1A1	12.45	0.0000
217287_s_at	transient receptor potential cation channel, subfamily C, member 6	TRPC6	60.26	0.0000
208323_s_at	annexin A13	ANXA13	12.81	0.0000
202688_at	tumor necrosis factor (ligand) superfamily, member 10	TNFSF10	17.19	0.0000
209821_at	interleukin 33	IL33	50.26	0.0000
206528_at	transient receptor potential cation channel, subfamily C, member 6	TRPC6	100.19	0.0000
227226_at	melanocortin 2 receptor accessory protein 2	MRAP2	35.62	0.0000
217590_s_at	transient receptor potential cation channel, subfamily A, member 1	TRPA1	6.60	0.0000
224189_x_at	ets homologous factor	EHF	45.99	0.0000
213228_at	phosphodiesterase 8B	PDE8B	14.20	0.0000
218204_s_at	FYVE and coiled-coil domain containing 1	FYCO1	27.88	0.0000
202687_s_at	tumor necrosis factor (ligand) superfamily, member 10	TNFSF10	22.04	0.0000
1555564_a_at	complement factor I	CFI	28.80	0.0000
219410_at	transmembrane protein 45A	TMEM45A	46.67	0.0000
208396_s_at	phosphodiesterase 1A, calmodulin-dependent	PDE1A	27.86	0.0000
1558680_s_at	phosphodiesterase 1A, calmodulin-dependent	PDE1A	18.26	0.0000
219429_at	fatty acid 2-hydroxylase	FA2H	8.16	0.0000
227948_at	FYVE, RhoGEF and PH domain containing 4	FGD4	7.23	0.0000
209581_at	phospholipase A2, group XVI	PLA2G16	16.31	0.0000
209631_s_at	G protein-coupled receptor 37 (endothelin receptor type B-like)	GPR37	9.66	0.0000
232361_s_at	ets homologous factor	EHF	10.98	0.0000
233340_at	serine peptidase inhibitor, Kazal type 13 (putative)	SPINK13	16.76	0.0000
208704_x_at	amyloid beta (A4) precursor-like protein 2	APLP2	7.42	0.0000
227819_at	leucine-rich repeat containing G protein-coupled receptor 6	LGR6	18.36	0.0000
203854_at	complement factor I	CFI	20.77	0.0000
225207_at	pyruvate dehydrogenase kinase, isozyme 4	PDK4	5.92	0.0000
206025_s_at	tumor necrosis factor, alpha-induced protein 6	TNFAIP6	53.06	0.0000
202769_at	cyclin G2	CCNG2	5.38	0.0000
227449_at	EPH receptor A4	EPHA4	52.56	0.0000
230645_at	FERM domain containing 3	FRMD3	7.95	0.0000
208703_s_at	amyloid beta (A4) precursor-like protein 2	APLP2	8.24	0.0000
223266_at	STE20-related kinase adaptor beta	STRADB	9.17	0.0000
232176_at	SLIT and NTRK-like family, member 6	SLITRK6	15.93	0.0000
232481_s_at	SLIT and NTRK-like family, member 6	SLITRK6	21.49	0.0000
208716_s_at	transmembrane and coiled-coil domains 1	TMCO1	6.38	0.0000
205765_at	cytochrome P450, family 3, subfamily A, polypeptide 5	CYP3A5	19.75	0.0000
206943_at	transforming growth factor, beta receptor 1	TGFBR1	3.72	0.0000
208248_x_at	amyloid beta (A4) precursor-like protein 2	APLP2	6.98	0.0000
212327_at	LIM and calponin homology domains 1	LIMCH1	4.54	0.0000
223571_at	C1q and tumor necrosis factor related protein 6	C1QTNF6	8.88	0.0000
201998_at	ST6 beta-galactosamide alpha-2,6-sialyltranferase 1	ST6GAL1	4.87	0.0000
224830_at	nudix (nucleoside diphosphate linked moiety X)-type motif 21	NUDT21	4.90	0.0000
221748_s_at	tensin 1	TNS1	7.46	0.0000
225645_at	ets homologous factor	EHF	32.25	0.0000
1554789_a_at	phosphodiesterase 8B	PDE8B	19.10	0.0000
1557795_s_at	neurotrophic tyrosine kinase, receptor, type 3	NTRK3	5.34	0.0000
204619_s_at	versican	VCAN	7.56	0.0000
206224_at	cystatin SN	CST1	14.19	0.0000
208096_s_at	collagen, type XXI, alpha 1	COL21A1	12.13	0.0000
1563933_a_at	phospholipase D family, member 5	PLD5	9.05	0.0000
230730_at	sarcoglycan, delta (35kDa dystrophin-associated glycoprotein)	SGCD	18.23	0.0000
223013_at	transducin (beta)-like 1 X-linked receptor 1	TBL1XR1	4.05	0.0000
228507_at	phosphodiesterase 3A, cGMP-inhibited	PDE3A	5.46	0.0000

Probeset ID	Gene title	Gene symbol	Fold change	FDR (%)
207334_s_at	transforming growth factor, beta receptor II (70/80kDa)	TGFBR2	7.84	0.0000
1553973_a_at	serine peptidase inhibitor, Kazal type 6	SPINK6	10.52	0.0000
206026_s_at	tumor necrosis factor, alpha-induced protein 6	TNFAIP6	39.91	0.0000
1555867_at	guanine nucleotide binding protein (G protein), gamma 4	GNG4	6.14	0.0000
205960_at	pyruvate dehydrogenase kinase, isozyme 4	PDK4	7.86	0.0000
228969_at	anterior gradient 2 homolog (Xenopus laevis)	AGR2	18.13	0.0000
225864_at	family with sequence similarity 84, member B	FAM84B	9.40	0.0000
201034_at	adducin 3 (gamma)	ADD3	4.20	0.0000
204622_x_at	nuclear receptor subfamily 4, group A, member 2	NR4A2	8.33	0.0000
219850_s_at	ets homologous factor	EHF	156.45	0.0000
219948_x_at	UDP glucuronosyltransferase 2 family, polypeptide A3	UGT2A3	19.21	0.0000
213543_at	sarcoglycan, delta (35kDa dystrophin-associated glycoprotein)	SGCD	16.05	0.0000
227870_at	immunoglobulin superfamily, DCC subclass, member 4	IGDCC4	18.35	0.0000
217478_s_at	major histocompatibility complex, class II, DM alpha	HLA-DMA	25.34	0.0000
200617_at	malectin	MLEC	5.63	0.0000
213800_at	complement factor H	CFH	8.39	0.0000
227020_at	yippee-like 2 (Drosophila)	YPEL2	6.61	0.0000
205654_at	complement component 4 binding protein, alpha	C4BPA	17.95	0.0000
209498_at	carcinoembryonic antigen-related cell adhesion molecule 1 (biliary glycoprotein)	CEACAM1	11.45	0.0000
202888_s_at	alanyl (membrane) aminopeptidase	ANPEP	15.90	0.0000
209863_s_at	tumor protein p63	TP63	7.54	0.0000
209711_at	solute carrier family 35 (UDP-glucuronic acid/UDP-N-acetylgalactosamine dual transporter), member D1	SLC35D1	5.81	0.0000
218313_s_at	UDP-N-acetyl-alpha-D-galactosamine:polypeptide N-acetylgalactosaminyltransferase 7 (GalNAc-T7)	GALNT7	4.65	0.0000
228748_at	CD59 molecule, complement regulatory protein	CD59	14.69	0.0000
212012_at	peroxidasin homolog (Drosophila)	PXDN	6.14	0.0000
205489_at	crystallin, mu	CRYM	22.62	0.0000
224209_s_at	guanine deaminase	GDA	28.81	0.0000
229242_at	tumor necrosis factor (ligand) superfamily, member 15	TNFSF15	19.49	0.0000
230924_at	tubulin tyrosine ligase-like family, member 6	TTLL6	12.42	0.0000
235309_at	ribosomal protein S15a	RPS15A	44.18	0.0000
219872_at	family with sequence similarity 198, member B	FAM198B	13.92	0.0000
205083_at	aldehyde oxidase 1	AOX1	4.26	0.0000
201744_s_at	lumican	LUM	13.55	0.0000
218361_at	golgi phosphoprotein 3-like	GOLPH3L	5.53	0.0000
218175_at	coiled-coil domain containing 92	CCDC92	3.80	0.0000
201185_at	HtrA serine peptidase 1	HTRA1	8.31	0.0000
222940_at	sulfotransferase family 1E, estrogen-preferring, member 1	SULT1E1	18.23	0.0000
206391_at	retinoic acid receptor responder (tazarotene induced) 1	RARRES1	26.92	0.0000
211571_s_at	versican	VCAN	17.53	0.0000
215076_s_at	collagen, type III, alpha 1	COL3A1	8.81	0.0000
224999_at	epidermal growth factor receptor	EGFR	6.30	0.0000
230104_s_at	tubulin polymerization promoting protein	TPPP	7.77	0.0000
208715_at	transmembrane and coiled-coil domains 1	TMCO1	9.57	0.0000
230183_at	exostosin 1	EXT1	23.56	0.0000
216733_s_at	glycine amidinotransferase (L-arginine:glycine amidinotransferase)	GATM	7.96	0.0000
209459_s_at	4-aminobutyrate aminotransferase	ABAT	12.58	0.0000
212586_at	calpastatin	CAST	3.52	0.0000
211098_x_at	transmembrane and coiled-coil domains 1	TMCO1	7.43	0.0000
222784_at	SPARC related modular calcium binding 1	SMOC1	13.08	0.0000
205226_at	platelet-derived growth factor receptor-like	PDGFRL	5.86	0.0000
218966_at	myosin VC	MYO5C	4.21	0.0000
207761_s_at	methyltransferase like 7A	METTL7A	9.40	0.0000
1554375_a_at	nuclear receptor subfamily 1, group H, member 4	NR1H4	63.79	0.0000

Probeset ID	Gene title	Gene symbol	Fold change	FDR (%)
222978_at	surfeit 4	SURF4	3.53	0.0000
200969_at	stress-associated endoplasmic reticulum protein 1	SERP1	5.28	0.0000
203939_at	5'-nucleotidase, ecto (CD73)	NT5E	5.77	0.0000
218242_s_at	suppressor of variegation 4-20 homolog 1 (Drosophila)	SUV420H1	3.10	0.0000
1554436_a_at	regenerating islet-derived family, member 4	REG4	51.62	0.0000
204686_at	insulin receptor substrate 1	IRS1	3.68	0.0000
208944_at	transforming growth factor, beta receptor II (70/80kDa)	TGFBR2	4.90	0.0000
204035_at	secretogranin II	SCG2	7.55	0.0000
228006_at	phosphatase and tensin homolog	PTEN	7.02	0.0000
203641_s_at	COBL-like 1	COBLL1	9.64	0.0000
212771_at	family with sequence similarity 171, member A1	FAM171A1	4.06	0.0000
209651_at	transforming growth factor beta 1 induced transcript 1	TGFB1I1	3.02	0.0000
205417_s_at	dystroglycan 1 (dystrophin-associated glycoprotein 1)	DAG1	3.31	0.0000
204221_x_at	GLI pathogenesis-related 1	GLIPR1	3.29	0.0000
218017_s_at	heparan-alpha-glucosaminide N-acetyltransferase	HGSNAT	4.37	0.0000
200970_s_at	stress-associated endoplasmic reticulum protein 1	SERP1	4.81	0.0000
214234_s_at	cytochrome P450, family 3, subfamily A, polypeptide 5	CYP3A5	11.21	0.0000
37892_at	collagen, type XI, alpha 1	COL11A1	5.87	0.0000
207714_s_at	serpin peptidase inhibitor, clade H (heat shock protein 47), member 1, (collagen binding protein 1)	SERPINH1	4.37	0.0000
241342_at	transmembrane protein 65	TMEM65	3.32	0.0000
201431_s_at	dihydropyrimidinase-like 3	DPYSL3	4.65	0.0000
225415_at	deltex 3-like (Drosophila)	DTX3L	3.17	0.0000
226136_at	GLI pathogenesis-related 1	GLIPR1	4.93	0.0000
205513_at	transcobalamin I (vitamin B12 binding protein, R binder family)	TCN1	12.06	0.0000
202202_s_at	laminin, alpha 4	LAMA4	7.22	0.0000
212339_at	erythrocyte membrane protein band 4.1-like 1	EPB41L1	4.37	0.0000
227630_at	protein phosphatase 2, regulatory subunit B', epsilon isoform	PPP2R5E	9.04	0.0000
202507_s_at	synaptosomal-associated protein, 25kDa	SNAP25	3.39	0.0000
215108_x_at	TOX high mobility group box family member 3	TOX3	16.23	0.0000
221773_at	ELK3, ETS-domain protein (SRF accessory protein 2)	ELK3	3.17	0.0000
210768_x_at	transmembrane and coiled-coil domains 1	TMCO1	6.88	0.0000
200645_at	GABA(A) receptor-associated protein	GABARAP	2.86	0.0000
221911_at	ets variant 1	ETV1	14.28	0.0000
227405_s_at	frizzled family receptor 8	FZD8	2.78	0.0000
212328_at	LIM and calponin homology domains 1	LIMCH1	4.29	0.0000
201578_at	podocalyxin-like	PODXL	4.90	0.0000
204260_at	chromogranin B (secretogranin 1)	CHGB	8.55	0.0000
218319_at	pellino E3 ubiquitin protein ligase 1	PELI1	12.58	0.0000
212345_s_at	cAMP responsive element binding protein 3-like 2	CREB3L2	5.55	0.0000
214954_at	sushi domain containing 5	SUSD5	28.29	0.0000
230563_at	RasGEF domain family, member 1A	RASGEF1A	52.56	0.0000
208025_s_at	high mobility group AT-hook 2	HMGA2	7.77	0.0000
225759_x_at	calmin (calponin-like, transmembrane)	CLMN	10.18	0.0000
203914_x_at	hydroxyprostaglandin dehydrogenase 15-(NAD)	HPGD	2.65	0.0000
206549_at	insulin-like 4 (placenta)	INSL4	5.57	0.0000
201117_s_at	carboxypeptidase E	CPE	8.82	0.0000
236300_at	phosphodiesterase 3A, cGMP-inhibited	PDE3A	6.86	0.0000
206389_s_at	phosphodiesterase 3A, cGMP-inhibited	PDE3A	5.17	0.0000
218876_at	tubulin polymerization-promoting protein family member 3	TPPP3	8.40	0.0000
213836_s_at	WD repeat domain, phosphoinositide interacting 1	WIPI1	24.83	0.0000
235046_at	inositol polyphosphate-4-phosphatase, type II, 105kDa	INPP4B	3.36	0.0000
226016_at	CD47 molecule	CD47	4.30	0.0000
218692_at	syntabulin (syntaxin-interacting)	SYBU	5.89	0.0000
225274_at	prenylcysteine oxidase 1	PCYOX1	3.91	0.0000
209015_s_at	DnaJ (Hsp40) homolog, subfamily B, member 6	DNAJB6	3.99	0.0000

Probeset ID	Gene title	Gene symbol	Fold change	FDR (%)
226142_at	GLI pathogenesis-related 1	GLIPR1	4.29	0.0000
207463_x_at	protease, serine, 3	PRSS3	8.60	0.0000
224836_at	tumor protein p53 inducible nuclear protein 2	TP53INP2	5.84	0.0000
215506_s_at	DIRAS family, GTP-binding RAS-like 3	DIRAS3	14.34	0.0000
210740_s_at	inositol-tetrakisphosphate 1-kinase	ITPK1	3.61	0.0000
212169_at	FK506 binding protein 9, 63 kDa	FKBP9	3.30	0.0000
212463_at	CD59 molecule, complement regulatory protein	CD59	3.74	0.0000
205421_at	solute carrier family 22 (extraneuronal monoamine transporter), member 3	SLC22A3	3.77	0.0000
205579_at	histamine receptor H1	HRH1	6.24	0.0000
202196_s_at	dickkopf 3 homolog (Xenopus laevis)	DKK3	11.88	0.0000
210130_s_at	transmembrane 7 superfamily member 2	TM7SF2	4.52	0.0000
203178_at	glycine amidinotransferase (L-arginine:glycine amidinotransferase)	GATM	6.41	0.0000
222712_s_at	mucin 13, cell surface associated	MUC13	5.98	0.0000
200613_at	adaptor-related protein complex 2, mu 1 subunit	AP2M1	2.83	0.0000
200631_s_at	SET nuclear oncogene	SET	3.04	0.0000
226145_s_at	Fraser syndrome 1	FRAS1	15.17	0.0000
212655_at	zinc finger, CCHC domain containing 14	ZCCHC14	7.47	0.0000
205433_at	butyrylcholinesterase	BCHE	3.63	0.0000
205743_at	SH3 and cysteine rich domain	STAC	3.90	0.0000
219667_s_at	B-cell scaffold protein with ankyrin repeats 1	BANK1	3.81	0.0000
242979_at	insulin receptor substrate 1	IRS1	6.59	0.0000
218309_at	calcium/calmodulin-dependent protein kinase II inhibitor 1	CAMK2N1	3.15	0.0000
201315_x_at	interferon induced transmembrane protein 2	IFITM2	3.79	0.0000
202838_at	fucosidase, alpha-L- 1, tissue	FUCA1	3.79	0.0000
219934_s_at	sulfotransferase family 1E, estrogen-preferring, member 1	SULT1E1	36.17	0.0000
226506_at	thrombospondin, type I, domain containing 4	THSD4	4.13	0.0000
211548_s_at	hydroxyprostaglandin dehydrogenase 15-(NAD)	HPGD	2.82	0.0000
223165_s_at	inositol hexakisphosphate kinase 2	IP6K2	2.58	0.0000
225821_s_at	biorientation of chromosomes in cell division 1-like 1	BOD1L1	2.76	0.0000
225732_at	kelch domain containing 5	KLHDC5	3.44	0.0000
223447_at	regenerating islet-derived family, member 4	REG4	29.09	0.0000
212203_x_at	interferon induced transmembrane protein 3	IFITM3	5.37	0.0000
223136_at	androgen-induced 1	AIG1	6.94	0.0000
208908_s_at	calpastatin	CAST	2.78	0.0000
228551_at	DENN/MADD domain containing 5B	DENND5B	4.94	0.0000
201207_at	tumor necrosis factor, alpha-induced protein 1 (endothelial)	TNFAIP1	2.74	0.0000
206392_s_at	retinoic acid receptor responder (tazarotene induced) 1	RARRES1	32.47	0.0000
204797_s_at	echinoderm microtubule associated protein like 1	EML1	3.57	0.0000
213301_x_at	tripartite motif containing 24	TRIM24	3.25	0.0000
228950_s_at	wntless homolog (Drosophila)	WLS	2.74	0.0000
223218_s_at	nuclear factor of kappa light polypeptide gene enhancer in B-cells inhibitor, zeta	NFKBIZ	4.34	0.0000
223592_s_at	ring finger protein 135	RNF135	3.05	0.0000
203038_at	protein tyrosine phosphatase, receptor type, K	PTPRK	4.39	0.0000
220016_at	AHNAK nucleoprotein	AHNAK	7.40	0.0000
221428_s_at	transducin (beta)-like 1 X-linked receptor 1	TBL1XR1	4.38	0.0000
205141_at	angiogenin, ribonuclease, RNase A family, 5	ANG	7.77	0.0000
209173_at	anterior gradient 2 homolog (Xenopus laevis)	AGR2	5.01	0.0000
225414_at	ring finger protein 149	RNF149	3.09	0.0000
222557_at	stathmin-like 3	STMN3	6.91	0.0000
226325_at	adenylosuccinate synthase like 1	ADSSL1	12.09	0.0000
215646_s_at	versican	VCAN	12.82	0.0000
202007_at	nidogen 1	NID1	5.58	0.0000
204831_at	cyclin-dependent kinase 8	CDK8	3.58	0.0000
212580_at	calpastatin	CAST	5.40	0.0000
206913_at	bile acid CoA: amino acid N-acyltransferase (glycine N-choloyltransferase)	BAAT	3.09	0.0000

Probeset ID	Gene title	Gene symbol	Fold change	FDR (%)
227865_at	idnK, gluconokinase homolog (E. coli)	IDNK	3.10	0.0000
225544_at	T-box 3	TBX3	3.38	0.0000
208892_s_at	dual specificity phosphatase 6	DUSP6	3.41	0.0000
224700_at	STT3, subunit of the oligosaccharyltransferase complex, homolog B (S. cerevisiae)	STT3B	2.89	0.0000
218901_at	phospholipid scramblase 4	PLSCR4	5.18	0.0000
224901_at	stearoyl-CoA desaturase 5	SCD5	28.69	0.0000
213695_at	paraoxonase 3	PON3	3.52	0.0000
229151_at	solute carrier family 14 (urea transporter), member 1 (Kidd blood group)	SLC14A1	25.98	0.0000
209373_at	mal, T-cell differentiation protein-like	MALL	4.06	0.0000
226086_at	synaptotagmin XIII	SYT13	4.46	0.0000
203216_s_at	myosin VI	MYO6	3.67	0.0000
204391_x_at	tripartite motif containing 24	TRIM24	3.07	0.0000
221011_s_at	limb bud and heart development homolog (mouse)	LBH	3.02	0.0000
213148_at	chromosome 2 open reading frame 72	C2orf72	3.91	0.0000
226748_at	LysM, putative peptidoglycan-binding, domain containing 2	LYSMD2	3.58	0.0000
213960_at	neurotrophic tyrosine kinase, receptor, type 3	NTRK3	5.42	0.0000
234963_s_at	fatty acid 2-hydroxylase	FA2H	9.63	0.0000
219909_at	matrix metallopeptidase 28	MMP28	67.27	0.0000
223597_at	intelectin 1 (galactofuranose binding)	ITLN1	19.27	0.0000
221766_s_at	family with sequence similarity 46, member A	FAM46A	9.32	0.0000
208939_at	selenophosphate synthetase 1	SEPHS1	2.64	0.0000
219734_at	SID1 transmembrane family, member 1	SIDT1	3.84	0.0000
202766_s_at	fibrillin 1	FBN1	6.60	0.0000
212325_at	LIM and calponin homology domains 1	LIMCH1	2.52	0.0000
211203_s_at	contactin 1	CNTN1	3.36	0.0000
204684_at	neuronal pentraxin I	NPTX1	3.86	0.0000
219682_s_at	T-box 3	TBX3	3.65	0.0000
214247_s_at	dickkopf 3 homolog (Xenopus laevis)	DKK3	16.62	0.0000
202729_s_at	latent transforming growth factor beta binding protein 1	LTBP1	3.04	0.0000
223194_s_at	solute carrier family 22, member 23	SLC22A23	5.61	0.0000
227722_at	ribosomal protein S23	RPS23	2.50	0.0000
201852_x_at	collagen, type III, alpha 1	COL3A1	10.24	0.0000
223204_at	family with sequence similarity 198, member B	FAM198B	4.73	0.0000
228302_x_at	calcium/calmodulin-dependent protein kinase II inhibitor 1	CAMK2N1	6.55	0.0000
207513_s_at	zinc finger protein 189	ZNF189	4.49	0.0000
209605_at	thiosulfate sulfurtransferase (rhodanese)	TST	2.59	0.0000
206340_at	nuclear receptor subfamily 1, group H, member 4	NR1H4	40.82	0.0000
201362_at	influenza virus NS1A binding protein	IVNS1ABP	2.81	0.0000
208891_at	dual specificity phosphatase 6	DUSP6	3.19	0.0000
203642_s_at	COBL-like 1	COBLL1	6.18	0.0000
222587_s_at	UDP-N-acetyl-alpha-D-galactosamine:polypeptide N-acetylgalactosaminyltransferase 7 (GalNAc-T7)	GALNT7	4.50	0.0000
222585_x_at	lysine-rich coiled-coil 1	KRCC1	5.41	0.0000
204620_s_at	versican	VCAN	9.31	0.0000
216623_x_at	TOX high mobility group box family member 3	TOX3	37.58	0.0000
201984_s_at	epidermal growth factor receptor	EGFR	6.40	0.0000
235199_at	ring finger protein 125, E3 ubiquitin protein ligase	RNF125	13.50	0.0000
227476_at	lysophosphatidylglycerol acyltransferase 1	LPGAT1	3.05	0.0000
229720_at	BCL2-associated athanogene	BAG1	4.90	0.0000
229441_at	protease, serine, 23	PRSS23	8.10	0.0000
204602_at	dickkopf 1 homolog (Xenopus laevis)	DKK1	2.94	0.0000
213629_x_at	metallothionein 1F	MT1F	7.57	0.0000
213652_at	proprotein convertase subtilisin/kexin type 5	PCSK5	4.01	0.0000
216238_s_at	fibrinogen beta chain	FGB	5.38	0.0000
206332_s_at	interferon, gamma-inducible protein 16	IFI16	4.98	0.0000

Probeset ID	Gene title	Gene symbol	Fold change	FDR (%)
202962_at	kinesin family member 13B	KIF13B	3.41	0.0000
242444_at	C1q and tumor necrosis factor related protein 6	C1QTNF6	11.05	0.0000
212128_s_at	dystroglycan 1 (dystrophin-associated glycoprotein 1)	DAG1	2.80	0.0000
212444_at	G protein-coupled receptor, family C, group 5, member A	GPRC5A	2.31	0.0000
212013_at	peroxidasin homolog (Drosophila)	PXDN	12.00	0.0000
226576_at	Rho GTPase activating protein 26	ARHGAP26	5.60	0.0000
221589_s_at	aldehyde dehydrogenase 6 family, member A1	ALDH6A1	5.65	0.0000
214746_s_at	zinc finger protein 467	ZNF467	14.49	0.0000
203085_s_at	transforming growth factor, beta 1	TGFB1	3.05	0.0000
203474_at	IQ motif containing GTPase activating protein 2	IQGAP2	5.12	0.0000
205305_at	fibrinogen-like 1	FGL1	3.78	0.0000
210330_at	sarcoglycan, delta (35kDa dystrophin-associated glycoprotein)	SGCD	5.36	0.0000
222787_s_at	transmembrane protein 106B	TMEM106B	4.99	0.0000
202562_s_at	chromosome 14 open reading frame 1	C14orf1	2.75	0.0000
219993_at	SRY (sex determining region Y)-box 17	SOX17	7.72	0.0000
208702_x_at	amyloid beta (A4) precursor-like protein 2	APLP2	3.21	0.0000
221872_at	retinoic acid receptor responder (tazarotene induced) 1	RARRES1	21.74	0.0000
205216_s_at	apolipoprotein H (beta-2-glycoprotein I)	APOH	4.85	0.0000
210095_s_at	insulin-like growth factor binding protein 3	IGFBP3	3.69	0.0000
203060_s_at	3'-phosphoadenosine 5'-phosphosulfate synthase 2	PAPSS2	2.61	0.0000
214321_at	nephroblastoma overexpressed	NOV	16.28	0.0000
229596_at	amidohydrolase domain containing 1	AMDHD1	5.25	0.0000
1553995_a_at	5'-nucleotidase, ecto (CD73)	NT5E	4.33	0.0000
202508_s_at	synaptosomal-associated protein, 25kDa	SNAP25	2.74	0.0000
226154_at	dynamin 1-like	DNM1L	3.24	0.0000
218303_x_at	lysine-rich coiled-coil 1	KRCC1	4.44	0.0000
204326_x_at	metallothionein 1X	MT1X	3.84	0.0000
225299_at	myosin VB	MYO5B	4.29	0.0000
225144_at	bone morphogenetic protein receptor, type II (serine/threonine kinase)	BMPR2	2.52	0.0000
213894_at	thrombospondin, type I, domain containing 7A	THSD7A	3.78	0.0000
215025_at	neurotrophic tyrosine kinase, receptor, type 3	NTRK3	7.11	0.0000
230720_at	ring finger protein 182	RNF182	3.33	0.0000
225835_at	solute carrier family 12 (sodium/potassium/chloride transporters), member 2	SLC12A2	4.07	0.0000
214203_s_at	proline dehydrogenase (oxidase) 1	PRODH	24.48	0.0000
205352_at	serpin peptidase inhibitor, clade I (neuroserpin), member 1	SERPINI1	5.53	0.0000
226535_at	integrin, beta 6	ITGB6	4.28	0.0000
213675_at	parvin, alpha	PARVA	5.69	0.0000
222853_at	fibronectin leucine rich transmembrane protein 3	FLRT3	3.76	0.0000
233329_s_at	lysine-rich coiled-coil 1	KRCC1	4.14	0.0000
227239_at	family with sequence similarity 126, member A	FAM126A	2.50	0.0000
221731_x_at	versican	VCAN	10.19	0.0000
212143_s_at	insulin-like growth factor binding protein 3	IGFBP3	3.53	0.0000
201069_at	matrix metallopeptidase 2 (gelatinase A, 72kDa gelatinase, 72kDa type IV collagenase)	MMP2	5.65	0.0000
238127_at	GAS6 antisense RNA 1 (non-protein coding)	GAS6-AS1	2.99	0.0000
226321_at	LysM, putative peptidoglycan-binding, domain containing 3	LYSMD3	3.17	0.0000
222937_s_at	matrix metallopeptidase 28	MMP28	6.05	0.0000
222641_s_at	family with sequence similarity 222, member B	FAM222B	2.86	0.0000
225996_at	LON peptidase N-terminal domain and ring finger 2	LONRF2	2.71	0.0000
34726_at	calcium channel, voltage-dependent, beta 3 subunit	CACNB3	3.47	0.0000
201324_at	epithelial membrane protein 1	EMP1	2.43	0.0000
204748_at	prostaglandin-endoperoxide synthase 2 (prostaglandin G/H synthase and cyclooxygenase)	PTGS2	3.57	0.0000
224707_at	cysteine-rich transmembrane module containing 1	CYSTM1	3.26	0.0000
202948_at	interleukin 1 receptor, type I	IL1R1	2.29	0.0000
201650_at	keratin 19	KRT19	2.59	0.0000

Probeset ID	Gene title	Gene symbol	Fold change	FDR (%)
200616_s_at	malectin	MLEC	4.46	0.0000
229004_at	ADAM metallopeptidase with thrombospondin type 1 motif, 15	ADAMTS15	5.54	0.0000
223217_s_at	nuclear factor of kappa light polypeptide gene enhancer in B-cells inhibitor, zeta	NFKBIZ	5.83	0.0000
211161_s_at	collagen, type III, alpha 1	COL3A1	9.83	0.0000
213241_at	plexin C1	PLXNC1	4.27	0.0000
229584_at	leucine-rich repeat kinase 2	LRRK2	5.34	0.0000
224331_s_at	mitochondrial ribosomal protein L36	MRPL36	3.08	0.0000
220613_s_at	synaptotagmin-like 2	SYTL2	4.46	0.0000
214459_x_at	major histocompatibility complex, class I, C	HLA-C	2.59	0.0000
228726_at	serpin peptidase inhibitor, clade B (ovalbumin), member 1	SERPINB1	4.16	0.0000
201753_s_at	adducin 3 (gamma)	ADD3	4.56	0.0000
223667_at	FK506 binding protein 7	FKBP7	5.56	0.0000
226722_at	family with sequence similarity 20, member C	FAM20C	2.47	0.0000
212521_s_at	phosphodiesterase 8A	PDE8A	2.70	0.0000
210454_s_at	potassium inwardly-rectifying channel, subfamily J, member 6	KCNJ6	4.86	0.0000
225731_at	ankyrin repeat domain 50	ANKRD50	3.02	0.0000
224826_at	glycerophosphocholine phosphodiesterase GDE1 homolog (S. cerevisiae)	GPCPD1	3.18	0.0000
202728_s_at	latent transforming growth factor beta binding protein 1	LTBP1	4.33	0.0000
205803_s_at	transient receptor potential cation channel, subfamily C, member 1	TRPC1	2.79	0.0000
214774_x_at	TOX high mobility group box family member 3	TOX3	27.65	0.0000
204534_at	vitronectin	VTN	3.09	0.0000
228821_at	ST6 beta-galactosamide alpha-2,6-sialyltranferase 2	ST6GAL2	4.45	0.0000
202770_s_at	cyclin G2	CCNG2	5.55	0.0000
204199_at	Ral GEF with PH domain and SH3 binding motif 1	RALGPS1	9.59	0.0000
226961_at	proline rich 15	PRR15	4.52	0.0000
211404_s_at	amyloid beta (A4) precursor-like protein 2	APLP2	3.28	0.0000
225524_at	anthrax toxin receptor 2	ANTXR2	3.38	0.0000
202388_at	regulator of G-protein signaling 2, 24kDa	RGS2	3.88	0.0000
235635_at	Rho GTPase activating protein 5	ARHGAP5	4.83	0.0000
212231_at	F-box protein 21	FBXO21	2.17	0.0000
229893_at	FERM domain containing 3	FRMD3	5.68	0.0000
35846_at	thyroid hormone receptor, alpha	THRA	2.91	0.0000
225327_at	family with sequence similarity 214, member A	FAM214A	2.61	0.0000
204099_at	SWI/SNF related, matrix associated, actin dependent regulator of chromatin, subfamily d, member 3	SMARCD3	6.56	0.0000
225270_at	neogenin 1	NEO1	2.50	0.0000
229850_at	3-ketodihydrosphingosine reductase	KDSR	3.87	0.0000
219612_s_at	fibrinogen gamma chain	FGG	4.61	0.0000
216248_s_at	nuclear receptor subfamily 4, group A, member 2	NR4A2	7.06	0.0000
219372_at	intraflagellar transport 81 homolog (Chlamydomonas)	IFT81	2.20	0.0000
211685_s_at	neurocalcin delta	NCALD	3.32	0.0000
222846_at	RAB8B, member RAS oncogene family	RAB8B	2.47	0.0000
218516_s_at	inositol monophosphatase domain containing 1	IMPAD1	2.37	0.0000
223821_s_at	sushi domain containing 4	SUSD4	4.91	0.0000
201244_s_at	v-raf-1 murine leukemia viral oncogene homolog 1	RAF1	2.63	0.0000
225412_at	transmembrane protein 87B	TMEM87B	9.31	0.0000
226656_at	cartilage associated protein	CRTAP	2.24	0.0000
219543_at	phenazine biosynthesis-like protein domain containing	PBLD	5.32	0.0000
204688_at	sarcoglycan, epsilon	SGCE	2.18	0.0000
209691_s_at	docking protein 4	DOK4	6.14	0.0000
217914_at	two pore segment channel 1	TPCN1	2.99	0.0000
211756_at	parathyroid hormone-like hormone	PTHLH	3.02	0.0000
213047_x_at	SET nuclear oncogene	SET	2.56	0.0000
201124_at	integrin, beta 5	ITGB5	3.37	0.0000
208940_at	selenophosphate synthetase 1	SEPHS1	3.10	0.0000

Probeset ID	Gene title	Gene symbol	Fold change	FDR (%)
1556629_a_at	Synaptosomal-associated protein, 25kDa	SNAP25	2.57	0.0000
200905_x_at	major histocompatibility complex, class I, E	HLA-E	2.79	0.0000
220327_at	vestigial like 3 (Drosophila)	VGLL3	15.00	0.0000
218729_at	latexin	LXN	2.74	0.0000
59697_at	RAB15, member RAS oncogene family	RAB15	3.26	0.0000
213123_at	microfibrillar-associated protein 3	MFAP3	3.68	0.0000
1553132_a_at	tandem C2 domains, nuclear	TC2N	8.57	0.0000
201079_at	synaptogyrin 2	SYNGR2	2.11	0.0000
217908_s_at	DDB1 and CUL4 associated factor 6	DCAF6	2.40	0.0000
202794_at	inositol polyphosphate-1-phosphatase	INPP1	2.51	0.0000
227038_at	sphingomyelin synthase 2	SGMS2	2.47	0.0000
215177_s_at	integrin, alpha 6	ITGA6	3.77	0.0000
226445_s_at	tripartite motif containing 41	TRIM41	2.75	0.0000
211559_s_at	cyclin G2	CCNG2	8.81	0.0000
211034_s_at	chromosome 12 open reading frame 51	C12orf51	2.56	0.0000
222760_at	zinc finger protein 703	ZNF703	3.32	0.0000
201581_at	thioredoxin-related transmembrane protein 4	TMX4	2.71	0.0000
203932_at	major histocompatibility complex, class II, DM beta	HLA-DMB	6.73	0.0000
204276_at	thymidine kinase 2, mitochondrial	TK2	3.57	0.0000
48825_at	inhibitor of growth family, member 4	ING4	2.61	0.0000
239367_at	brain-derived neurotrophic factor	BDNF	2.82	0.0000
222453_at	cytochrome b reductase 1	CYBRD1	3.07	0.0000
203184_at	fibrillin 2	FBN2	2.41	0.0000
205174_s_at	glutaminyl-peptide cyclotransferase	QPCT	3.12	0.0000
204621_s_at	nuclear receptor subfamily 4, group A, member 2	NR4A2	6.47	0.0000
226189_at	integrin, beta 8	ITGB8	7.15	0.0000
1554997_a_at	prostaglandin-endoperoxide synthase 2 (prostaglandin G/H synthase and cyclooxygenase)	PTGS2	4.31	0.0000
230008_at	thrombospondin, type I, domain containing 7A	THSD7A	8.68	0.0000
200804_at	transmembrane BAX inhibitor motif containing 6	TMBIM6	2.40	0.0000
204714_s_at	coagulation factor V (proaccelerin, labile factor)	F5	3.57	0.0000
208933_s_at	lectin, galactoside-binding, soluble, 8	LGALS8	2.49	0.0000
238808_at	homeobox A13	HOXA13	5.25	0.0000
225809_at	prostate androgen-regulated mucin-like protein 1	PARM1	12.33	0.0000
213075_at	olfactomedin-like 2A	OLFML2A	4.37	0.0000
208812_x_at	major histocompatibility complex, class I, C	HLA-C	2.57	0.0000
203453_at	sodium channel, non-voltage-gated 1 alpha subunit	SCNN1A	3.59	0.0000
227458_at	CD274 molecule	CD274	4.98	0.0000
204988_at	fibrinogen beta chain	FGB	4.81	0.0000
224827_at	ubiquitin domain containing 2	UBTD2	2.10	0.0000
202238_s_at	nicotinamide N-methyltransferase	NNMT	2.38	0.0000
214783_s_at	annexin A11	ANXA11	3.37	0.0000
225961_at	kelch domain containing 5	KLHDC5	2.79	0.0000
212148_at	pre-B-cell leukemia homeobox 1	PBX1	2.20	0.0000
224647_at	cyclin Y	CCNY	3.50	0.0000
211574_s_at	CD46 molecule, complement regulatory protein	CD46	2.35	0.0000
222835_at	thrombospondin, type I, domain containing 4	THSD4	3.99	0.0000
1555292_at	family with sequence similarity 40, member B	FAM40B	3.26	0.0000
226529_at	transmembrane protein 106B	TMEM106B	3.19	0.0000
224724_at	sulfatase 2	SULF2	3.73	0.0000
205407_at	reversion-inducing-cysteine-rich protein with kazal motifs	RECK	10.83	0.0000
40093_at	basal cell adhesion molecule (Lutheran blood group)	BCAM	3.75	0.0000
203791_at	Dmx-like 1	DMXL1	2.87	0.0000
212094_at	paternally expressed 10	PEG10	3.04	0.0000
214806_at	bicaudal D homolog 1 (Drosophila)	BICD1	2.89	0.0000
214235_at	cytochrome P450, family 3, subfamily A, polypeptide 5	CYP3A5	25.32	0.0000

Probeset ID	Gene title	Gene symbol	Fold change	FDR (%)
231130_at	FK506 binding protein 7	FKBP7	6.68	0.0000
227046_at	solute carrier family 39 (metal ion transporter), member 11	SLC39A11	2.42	0.0000
212558_at	sprouty homolog 1, antagonist of FGF signaling (Drosophila)	SPRY1	5.03	0.0000
214812_s_at	MOB kinase activator 1A	MOB1A	2.04	0.0000
203058_s_at	3'-phosphoadenosine 5'-phosphosulfate synthase 2	PAPSS2	3.08	0.0000
208387_s_at	matrix metallopeptidase 24 (membrane-inserted)	MMP24	3.77	0.0000
201752_s_at	adducin 3 (gamma)	ADD3	4.50	0.0000
225525_at	KIAA1671	KIAA1671	2.44	0.0000
219274_at	tetraspanin 12	TSPAN12	7.18	0.0000
208782_at	follistatin-like 1	FSTL1	3.40	0.0000
228336_at	PWWP domain containing 2A	PWWP2A	3.37	0.0000
225325_at	major facilitator superfamily domain containing 6	MFSD6	2.41	0.0000
238846_at	tumor necrosis factor receptor superfamily, member 11a, NFKB activator	TNFRSF11A	8.67	0.0000
228038_at	SRY (sex determining region Y)-box 2	SOX2	3.51	0.0000
231579_s_at	TIMP metallopeptidase inhibitor 2	TIMP2	2.17	0.0000
219201_s_at	twisted gastrulation homolog 1 (Drosophila)	TWSG1	2.32	0.0000
223677_at	autophagy related 10	ATG10	3.76	0.0000
228142_at	ubiquinol-cytochrome c reductase, complex III subunit X	UQCR10	5.75	0.0000
219250_s_at	fibronectin leucine rich transmembrane protein 3	FLRT3	2.59	0.0000
203888_at	thrombomodulin	THBD	2.32	0.0000
229638_at	iroquois homeobox 3	IRX3	3.38	0.0000
217165_x_at	metallothionein 1F	MT1F	5.05	0.0000
209772_s_at	CD24 molecule	CD24	2.37	0.0000
226705_at	fibroblast growth factor receptor 1	FGFR1	3.17	0.0000
208893_s_at	dual specificity phosphatase 6	DUSP6	4.90	0.0000
211194_s_at	tumor protein p63	TP63	6.81	0.0000
224694_at	anthrax toxin receptor 1	ANTXR1	2.16	0.0000
201724_s_at	UDP-N-acetyl-alpha-D-galactosamine:polypeptide N-acetylgalactosaminyltransferase 1 (GalNAc-T1)	GALNT1	2.76	0.0000
219010_at	chromosome 1 open reading frame 106	C1orf106	2.47	0.0000
226820_at	zinc finger protein 362	ZNF362	3.09	0.0000
242761_s_at	zinc finger protein 420	ZNF420	5.27	0.0000
235766_x_at	RAB27A, member RAS oncogene family	RAB27A	8.92	0.0000
219732_at	lipid phosphate phosphatase-related protein type 1	LPPR1	2.82	0.0000
201056_at	golgin B1	GOLGB1	2.59	0.0000
204678_s_at	potassium channel, subfamily K, member 1	KCNK1	3.06	0.0000
228057_at	DNA-damage-inducible transcript 4-like	DDIT4L	7.74	0.0000
231945_at	filamin A interacting protein 1	FILIP1	3.04	0.0000
218696_at	eukaryotic translation initiation factor 2-alpha kinase 3	EIF2AK3	2.61	0.0000
227983_at	Rab interacting lysosomal protein-like 2	RILPL2	2.11	0.0000
1568618_a_at	UDP-N-acetyl-alpha-D-galactosamine:polypeptide N-acetylgalactosaminyltransferase 1 (GalNAc-T1)	GALNT1	2.43	0.0000
228737_at	TOX high mobility group box family member 2	TOX2	3.20	0.0000
228306_at	cornichon homolog 4 (Drosophila)	CNIH4	2.31	0.0000
221489_s_at	sprouty homolog 4 (Drosophila)	SPRY4	3.38	0.0000
1552312_a_at	microfibrillar-associated protein 3	MFAP3	6.30	0.0000
223395_at	ABI family, member 3 (NESH) binding protein	ABI3BP	9.34	0.0000
203887_s_at	thrombomodulin	THBD	2.54	0.0000
209159_s_at	NDRG family member 4	NDRG4	2.15	0.0000
239273_s_at	matrix metallopeptidase 28	MMP28	23.96	0.0000
212092_at	paternally expressed 10	PEG10	4.20	0.0000
221478_at	BCL2/adenovirus E1B 19kDa interacting protein 3-like	BNIP3L	3.03	0.0000
212070_at	G protein-coupled receptor 56	GPR56	2.79	0.0000
224325_at	frizzled family receptor 8	FZD8	3.51	0.0000
226183_at	glycogen synthase kinase 3 beta	GSK3B	7.74	0.0000
222692_s_at	fibronectin type III domain containing 3B	FNDC3B	2.84	0.0000

Probeset ID	Gene title	Gene symbol	Fold change	FDR (%)
202295_s_at	cathepsin H	CTSH	2.98	0.0000
217771_at	golgi membrane protein 1	GOLM1	2.02	0.0000
218736_s_at	palmdelphin	PALMD	11.24	0.0000
40189_at	SET nuclear oncogene	SET	2.80	0.0000
206127_at	ELK3, ETS-domain protein (SRF accessory protein 2)	ELK3	6.92	0.0000
208966_x_at	interferon, gamma-inducible protein 16	IFI16	5.95	0.0000
214109_at	LPS-responsive vesicle trafficking, beach and anchor containing	LRBA	9.40	0.0000
202036_s_at	secreted frizzled-related protein 1	SFRP1	3.08	0.0000
224789_at	DDB1 and CUL4 associated factor 12	DCAF12	2.95	0.0000
208786_s_at	microtubule-associated protein 1 light chain 3 beta	MAP1LC3B	2.57	0.0000
204404_at	solute carrier family 12 (sodium/potassium/chloride transporters), member 2	SLC12A2	2.80	0.0000
205942_s_at	acyl-CoA synthetase medium-chain family member 3	ACSM3	4.62	0.0000
211549_s_at	hydroxyprostaglandin dehydrogenase 15-(NAD)	HPGD	2.25	0.0000
205552_s_at	2'-5'-oligoadenylate synthetase 1, 40/46kDa	OAS1	3.41	0.0000
200847_s_at	transmembrane protein 66	TMEM66	2.15	0.0000
202117_at	Rho GTPase activating protein 1	ARHGAP1	2.23	0.0000
230570_at	eukaryotic translation initiation factor 3, subunit H	EIF3H	3.96	0.0000
201482_at	quiescin Q6 sulfhydryl oxidase 1	QSOX1	2.06	0.0000
222468_at	KIAA0319-like	KIAA0319L	2.18	0.0000
210145_at	phospholipase A2, group IVA (cytosolic, calcium-dependent)	PLA2G4A	3.59	0.0000
204920_at	carbamoyl-phosphate synthase 1, mitochondrial	CPS1	2.81	0.0000
204070_at	retinoic acid receptor responder (tazarotene induced) 3	RARRES3	4.32	0.0000
202037_s_at	secreted frizzled-related protein 1	SFRP1	2.23	0.0000
201302_at	annexin A4	ANXA4	2.28	0.0000
224984_at	nuclear factor of activated T-cells 5, tonicity-responsive	NFAT5	2.78	0.0000
227210_at	Scm-like with four mbt domains 2	SFMBT2	3.26	0.0000
224946_s_at	coiled-coil domain containing 115	CCDC115	2.39	0.0000
225171_at	Rho GTPase activating protein 18	ARHGAP18	2.21	0.0000
201286_at	syndecan 1	SDC1	2.45	0.0000
202504_at	tripartite motif containing 29	TRIM29	2.77	0.0000
222209_s_at	transmembrane protein 135	TMEM135	2.14	0.0000
212336_at	erythrocyte membrane protein band 4.1-like 1	EPB41L1	3.61	0.0000
202013_s_at	exostosin 2	EXT2	3.37	0.0000
212488_at	collagen, type V, alpha 1	COL5A1	2.29	0.0000
212190_at	serpin peptidase inhibitor, clade E (nexin, plasminogen activator inhibitor type 1), member 2	SERPINE2	2.46	0.0000
227478_at	SET binding protein 1	SETBP1	3.16	0.0000
201656_at	integrin, alpha 6	ITGA6	3.04	0.0000
224002_s_at	FK506 binding protein 7	FKBP7	2.78	0.0000
225735_at	ankyrin repeat domain 50	ANKRD50	3.87	0.0000
202304_at	fibronectin type III domain containing 3A	FNDC3A	3.41	0.0000
201910_at	FERM, RhoGEF (ARHGEF) and pleckstrin domain protein 1 (chondrocyte-derived)	FARP1	2.58	0.0000
204011_at	sprouty homolog 2 (Drosophila)	SPRY2	2.60	0.0000
224659_at	selenoprotein N, 1	SEPN1	3.07	0.0000
32094_at	carbohydrate (chondroitin 6) sulfotransferase 3	CHST3	3.27	0.0000
223721_s_at	DnaJ (Hsp40) homolog, subfamily C, member 12	DNAJC12	2.49	0.0000
203080_s_at	bromodomain adjacent to zinc finger domain, 2B	BAZ2B	2.36	0.0000
202869_at	2'-5'-oligoadenylate synthetase 1, 40/46kDa	OAS1	9.62	0.0000
212256_at	UDP-N-acetyl-alpha-D-galactosamine:polypeptide N-acetylgalactosaminyltransferase 10 (GalNAc-T10)	GALNT10	2.04	0.0000
225481_at	FERM domain containing 6	FRMD6	2.06	0.0000
228962_at	phosphodiesterase 4D, cAMP-specific	PDE4D	2.61	0.0000
222654_at	inositol monophosphatase domain containing 1	IMPAD1	2.43	0.0000
203859_s_at	paralemmin	PALM	3.22	0.0000
229842_at	E74-like factor 3 (ets domain transcription factor, epithelial-specific)	ELF3	3.11	0.0000

Probeset ID	Gene title	Gene symbol	Fold change	FDR (%)
209712_at	solute carrier family 35 (UDP-glucuronic acid/UDP-N-acetylgalactosamine dual transporter), member D1	SLC35D1	4.36	0.0000
226408_at	TEA domain family member 2	TEAD2	7.56	0.0000
222566_at	suppressor of variegation 4-20 homolog 1 (Drosophila)	SUV420H1	6.20	0.0000
201194_at	selenoprotein W, 1	SEPW1	2.23	0.0000
213572_s_at	serpin peptidase inhibitor, clade B (ovalbumin), member 1	SERPINB1	3.10	0.0000
202365_at	unc-119 homolog B (C. elegans)	UNC119B	2.52	0.0000
226577_at	presenilin 1	PSEN1	3.06	0.0000
232014_at	zinc finger protein 30	ZNF30	2.61	0.0000
219460_s_at	transmembrane protein 127	TMEM127	2.86	0.0000
214683_s_at	CDC-like kinase 1	CLK1	2.31	0.0000
236026_at	G patch domain containing 2	GPATCH2	4.19	0.0000
209286_at	CDC42 effector protein (Rho GTPase binding) 3	CDC42EP3	3.59	0.0000
200813_s_at	platelet-activating factor acetylhydrolase 1b, regulatory subunit 1 (45kDa)	PAFAH1B1	2.61	0.0000
213131_at	olfactomedin 1	OLFM1	4.27	0.0000
218584_at	tectonic family member 1	TCTN1	3.44	0.0000
229839_at	scavenger receptor class A, member 5 (putative)	SCARA5	3.73	0.0000
222633_at	transducin (beta)-like 1 X-linked receptor 1	TBL1XR1	4.10	0.0000
227422_at	striatin, calmodulin binding protein	STRN	3.22	0.0000
213171_s_at	matrix metallopeptidase 24 (membrane-inserted)	MMP24	4.59	0.0000
221864_at	ORAI calcium release-activated calcium modulator 3	ORAI3	2.49	0.0000
203420_at	family with sequence similarity 8, member A1	FAM8A1	8.98	0.0000
227776_at	alkaline ceramidase 3	ACER3	2.11	0.0000
203786_s_at	tumor protein D52-like 1	TPD52L1	2.67	0.0000
225221_at	zinc finger with KRAB and SCAN domains 1	ZKSCAN1	2.62	0.0000
209568_s_at	ral guanine nucleotide dissociation stimulator-like 1	RGL1	2.22	0.0000
220092_s_at	anthrax toxin receptor 1	ANTXR1	2.22	0.0000
1557905_s_at	CD44 molecule (Indian blood group)	CD44	2.24	0.0000
218648_at	CREB regulated transcription coactivator 3	CRTC3	2.20	0.0000
226132_s_at	mannosidase, endo-alpha-like	MANEAL	3.54	0.0000
208760_at	ubiquitin-conjugating enzyme E2I	UBE2I	2.72	0.0000
226684_at	autophagy related 2B	ATG2B	4.99	0.0000
217889_s_at	cytochrome b reductase 1	CYBRD1	2.53	0.0000
213891_s_at	transcription factor 4	TCF4	4.16	0.0000
228402_at	zinc finger, BED-type containing 3	ZBED3	3.63	0.0000
226374_at	coxsackie virus and adenovirus receptor	CXADR	3.71	0.0000
231964_at	bicaudal D homolog 1 (Drosophila)	BICD1	3.98	0.0000
210372_s_at	tumor protein D52-like 1	TPD52L1	3.53	0.0000
212646_at	raftlin, lipid raft linker 1	RFTN1	4.38	0.0000
225611_at	microtubule associated serine/threonine kinase family member 4	MAST4	3.42	0.0000
1552931_a_at	phosphodiesterase 8A	PDE8A	2.93	0.0000
226861_at	ankyrin repeat and SOCS box containing 8	ASB8	2.02	0.0000
238520_at	transcriptional regulating factor 1	TRERF1	4.36	0.0000
225685_at	CDC42 effector protein (Rho GTPase binding) 3	CDC42EP3	2.84	0.0000
225182_at	transmembrane protein 50B	TMEM50B	2.87	0.0000
201633_s_at	cytochrome b5 type B (outer mitochondrial membrane)	CYB5B	2.06	0.0000
209082_s_at	collagen, type XVIII, alpha 1	COL18A1	2.20	0.0000
212692_s_at	LPS-responsive vesicle trafficking, beach and anchor containing	LRBA	3.14	0.0000
201095_at	death-associated protein	DAP	2.81	0.0000
223085_at	ring finger protein 19A, E3 ubiquitin protein ligase	RNF19A	2.20	0.0000
228375_at	immunoglobulin superfamily, member 11	IGSF11	2.79	0.0000
200791_s_at	IQ motif containing GTPase activating protein 1	IQGAP1	2.04	0.0000
223591_at	ring finger protein 135	RNF135	2.51	0.0000
226279_at	protease, serine, 23	PRSS23	2.27	0.0000
209130_at	synaptosomal-associated protein, 23kDa	SNAP23	2.43	0.0000
225295_at	solute carrier family 39 (zinc transporter), member 10	SLC39A10	2.05	0.0000

Probeset ID	Gene title	Gene symbol	Fold change	FDR (%)
219330_at	vang-like 1 (van gogh, Drosophila)	VANGL1	2.76	0.0000
220079_s_at	ubiquitin specific peptidase 48	USP48	2.06	0.0000
209287_s_at	CDC42 effector protein (Rho GTPase binding) 3	CDC42EP3	2.72	0.0000
1555123_at	ST6 beta-galactosamide alpha-2,6-sialyltranferase 2	ST6GAL2	4.38	0.0000
201510_at	E74-like factor 3 (ets domain transcription factor, epithelial-specific)	ELF3	3.14	0.0000
204517_at	peptidylprolyl isomerase C (cyclophilin C)	PPIC	2.26	0.0000
230403_at	regulatory factor X, 3 (influences HLA class II expression)	RFX3	2.80	0.0000
228520_s_at	Amyloid beta (A4) precursor-like protein 2	APLP2	6.92	0.0000
218976_at	DnaJ (Hsp40) homolog, subfamily C, member 12	DNAJC12	2.16	0.0000
228630_at	zinc finger protein 84	ZNF84	4.53	0.0000
222932_at	ets homologous factor	EHF	10.72	0.0000
233555_s_at	sulfatase 2	SULF2	3.69	0.0000
228602_at	sarcoglycan, delta (35kDa dystrophin-associated glycoprotein)	SGCD	8.89	0.0000
204908_s_at	B-cell CLL/lymphoma 3	BCL3	2.26	0.0000
226754_at	zinc finger protein 251	ZNF251	3.26	0.0000
227256_at	ubiquitin specific peptidase 31	USP31	2.31	0.0000
203882_at	interferon regulatory factor 9	IRF9	2.89	0.0000
201363_s_at	influenza virus NS1A binding protein	IVNS1ABP	2.65	0.0000
214875_x_at	amyloid beta (A4) precursor-like protein 2	APLP2	2.93	0.0000
223475_at	cysteine-rich secretory protein LCCL domain containing 1	CRISPLD1	5.33	0.0000
235012_at	leucine-rich repeats and calponin homology (CH) domain containing 1	LRCH1	3.56	0.0000
227209_at	contactin 1	CNTN1	3.15	0.0000
203999_at	synaptotagmin I	SYT1	2.59	0.0000
214738_s_at	NIMA (never in mitosis gene a)- related kinase 9	NEK9	2.44	0.0000
209288_s_at	CDC42 effector protein (Rho GTPase binding) 3	CDC42EP3	3.10	0.0000
208807_s_at	chromodomain helicase DNA binding protein 3	CHD3	2.94	0.0000
226104_at	ring finger protein 170	RNF170	2.53	0.0000
223058_at	family with sequence similarity 107, member B	FAM107B	2.16	0.0000
225150_s_at	rhotekin	RTKN	4.05	0.0000
217862_at	protein inhibitor of activated STAT, 1	PIAS1	2.64	0.0000
201628_s_at	Ras-related GTP binding A	RRAGA	2.04	0.0000
229459_at	family with sequence similarity 19 (chemokine (C-C motif)-like), member A5	FAM19A5	5.66	0.0000
202433_at	solute carrier family 35, member B1	SLC35B1	2.90	0.0000
212119_at	ras homolog family member Q	RHOQ	2.04	0.0000
204284_at	protein phosphatase 1, regulatory subunit 3C	PPP1R3C	2.72	0.0000
222497_x_at	NMD3 homolog (S. cerevisiae)	NMD3	2.06	0.0000
212372_at	myosin, heavy chain 10, non-muscle	MYH10	2.01	0.0000
203803_at	prenylcysteine oxidase 1	PCYOX1	3.37	0.0000
219164_s_at	autophagy related 2B	ATG2B	2.86	0.0000
228384_s_at	pyridine nucleotide-disulphide oxidoreductase domain 2	PYROXD2	2.85	0.0000
228072_at	synaptotagmin XII	SYT12	3.40	0.0000
201325_s_at	epithelial membrane protein 1	EMP1	2.32	0.0000
228506_at	non-SMC element 4 homolog A (S. cerevisiae)	NSMCE4A	4.42	0.0000
229576_s_at	T-box 3	TBX3	4.05	0.0000
206461_x_at	metallothionein 1H	MT1H	2.88	0.0000
201339_s_at	sterol carrier protein 2	SCP2	2.06	0.0000
227407_at	transmembrane anterior posterior transformation 1	TAPT1	2.76	0.0000
209007_s_at	chromosome 1 open reading frame 63	C1orf63	2.52	0.0000
205500_at	complement component 5	C5	3.05	0.0000
225060_at	low density lipoprotein receptor-related protein 11	LRP11	2.52	0.0000
235110_at	phospholipase A2, group XVI	PLA2G16	4.11	0.0000
201220_x_at	C-terminal binding protein 2	CTBP2	2.02	0.0000
228131_at	excision repair cross-complementing rodent repair deficiency, complementation group 1 (includes overlapping antisense sequence)	ERCC1	2.63	0.0000
228796_at	copine IV	CPNE4	2.82	0.0000
202237_at	nicotinamide N-methyltransferase	NNMT	2.10	0.0000

Probeset ID	Gene title	Gene symbol	Fold change	FDR (%)
231234_at	cathepsin C	CTSC	3.90	0.0000
217989_at	hydroxysteroid (17-beta) dehydrogenase 11	HSD17B11	2.12	0.0000
227690_at	gamma-aminobutyric acid (GABA) A receptor, beta 3	GABRB3	2.06	0.0000
209462_at	amyloid beta (A4) precursor-like protein 1	APLP1	2.39	0.0000
221588_x_at	aldehyde dehydrogenase 6 family, member A1	ALDH6A1	3.07	0.0000
202743_at	phosphoinositide-3-kinase, regulatory subunit 3 (gamma)	PIK3R3	2.74	0.0000
222725_s_at	palmdelphin	PALMD	8.64	0.0000
227138_at	cartilage associated protein	CRTAP	3.71	0.0000
208796_s_at	cyclin G1	CCNG1	2.58	0.0000
221590_s_at	aldehyde dehydrogenase 6 family, member A1	ALDH6A1	8.43	0.0000
226100_at	myeloid/lymphoid or mixed-lineage leukemia 5 (trithorax homolog, Drosophila)	MLL5	2.95	0.0000
226118_at	centromere protein O	CENPO	2.73	0.0000
204497_at	adenylate cyclase 9	ADCY9	4.28	0.0000
202651_at	lysophosphatidylglycerol acyltransferase 1	LPGAT1	2.30	0.0000
204990_s_at	integrin, beta 4	ITGB4	4.16	0.0000
205698_s_at	mitogen-activated protein kinase kinase 6	MAP2K6	2.12	0.0000
212522_at	phosphodiesterase 8A	PDE8A	2.70	0.0000
225599_s_at	triple QxxK/R motif containing	TRIQK	2.60	0.0000
222494_at	forkhead box N3	FOXN3	4.57	0.0000
209018_s_at	PTEN induced putative kinase 1	PINK1	2.15	0.0000
210065_s_at	uroplakin 1B	UPK1B	2.31	0.0000
227288_at	SREK1-interacting protein 1	SREK1IP1	2.87	0.0000
201843_s_at	EGF containing fibulin-like extracellular matrix protein 1	EFEMP1	2.38	0.0000
205650_s_at	fibrinogen alpha chain	FGA	2.34	0.0000
1553113_s_at	cyclin-dependent kinase 8	CDK8	3.06	0.0000
202079_s_at	trafficking protein, kinesin binding 1	TRAK1	2.77	0.0000
214198_s_at	DiGeorge syndrome critical region gene 2	DGCR2	2.30	0.0000
217188_s_at	chromosome 14 open reading frame 1	C14orf1	3.12	0.0000
234998_at	RAB11A, member RAS oncogene family	RAB11A	3.48	0.0000
41047_at	chromosome 9 open reading frame 16	C9orf16	2.02	0.0000
224973_at	family with sequence similarity 46, member A	FAM46A	15.73	0.0000
224391_s_at	sialic acid acetylesterase	SIAE	2.23	0.0000
205463_s_at	platelet-derived growth factor alpha polypeptide	PDGFA	2.78	0.0000
224560_at	TIMP metallopeptidase inhibitor 2	TIMP2	2.28	0.0000
219383_at	proline rich 5 like	PRR5L	7.06	0.0000
235023_at	Vacuolar protein sorting 13 homolog C (S. cerevisiae)	VPS13C	4.29	0.0000
202593_s_at	glycerophosphodiester phosphodiesterase 1	GDE1	2.16	0.0000
203758_at	cathepsin O	CTSO	2.51	0.0000
224835_at	glycerophosphocholine phosphodiesterase GDE1 homolog (S. cerevisiae)	GPCPD1	3.59	0.0000
203421_at	tumor protein p53 inducible protein 11	TP53I11	3.40	0.0000
226660_at	ribosomal protein S6 kinase, 70kDa, polypeptide 1	RPS6KB1	2.35	0.0000
215071_s_at	histone cluster 1, H2ac	HIST1H2AC	3.47	0.0000
204490_s_at	CD44 molecule (Indian blood group)	CD44	2.06	0.0000
201299_s_at	MOB kinase activator 1A	MOB1A	2.37	0.0000
212062_at	ATPase, class II, type 9A	ATP9A	2.13	0.0000
217729_s_at	amino-terminal enhancer of split	AES	2.48	0.0000
218476_at	protein-O-mannosyltransferase 1	POMT1	2.38	0.0000
34408_at	reticulon 2	RTN2	3.06	0.0000
226364_at	huntingtin interacting protein 1	HIP1	2.37	0.0000
203372_s_at	suppressor of cytokine signaling 2	SOCS2	2.00	0.0000
207467_x_at	calpastatin	CAST	2.20	0.0000
204290_s_at	aldehyde dehydrogenase 6 family, member A1	ALDH6A1	4.82	0.0000
215058_at	DENN/MADD domain containing 5B	DENND5B	6.20	0.0000
202959_at	methylmalonyl CoA mutase	MUT	3.50	0.0000
212899_at	cyclin-dependent kinase 19	CDK19	2.46	0.0000

Probeset ID	Gene title	Gene symbol	Fold change	FDR (%)
208683_at	calpain 2, (m/II) large subunit	CAPN2	2.72	0.0000
228905_at	pericentriolar material 1	PCM1	6.36	0.0000
224891_at	forkhead box O3	FOXO3	2.05	0.0000
204017_at	KDEL (Lys-Asp-Glu-Leu) endoplasmic reticulum protein retention receptor 3	KDELR3	2.12	0.0000
202008_s_at	nidogen 1	NID1	5.46	0.0000
220035_at	nucleoporin 210kDa	NUP210	4.94	0.0000
203186_s_at	S100 calcium binding protein A4	S100A4	2.10	0.0000
201646_at	scavenger receptor class B, member 2	SCARB2	2.06	0.0000
200911_s_at	transforming, acidic coiled-coil containing protein 1	TACC1	2.09	0.0000
210835_s_at	C-terminal binding protein 2	CTBP2	2.19	0.0000
227669_at	brain protein 44	BRP44	2.47	0.0000
203185_at	Ras association (RalGDS/AF-6) domain family member 2	RASSF2	2.38	0.0000
242224_at	G patch domain containing 2	GPATCH2	3.18	0.0000
231786_at	homeobox A13	HOXA13	7.35	0.0000
233547_x_at	phosphodiesterase 1A, calmodulin-dependent	PDE1A	3.75	0.0000
235736_at	uncharacterized LOC100287482	LOC1002874<br>82	3.12	0.0000
213032_at	nuclear factor I/B	NFIB	3.79	0.0000
218687_s_at	mucin 13, cell surface associated	MUC13	6.19	0.0000
214177_s_at	pre-B-cell leukemia homeobox interacting protein 1	PBXIP1	3.35	0.0000
209834_at	carbohydrate (chondroitin 6) sulfotransferase 3	CHST3	2.86	0.0000
209690_s_at	docking protein 4	DOK4	4.30	0.0000
201995_at	exostosin 1	EXT1	2.33	0.0000
228256_s_at	erythrocyte membrane protein band 4.1 like 4A	EPB41L4A	8.18	0.0000
226214_at	glycerophosphodiester phosphodiesterase 1	GDE1	2.07	0.0000
220999_s_at	cytoplasmic FMR1 interacting protein 2	CYFIP2	2.96	0.0000
224688_at	transmembrane protein 248	TMEM248	2.25	0.0000
91816_f_at	mex-3 homolog D (C. elegans)	MEX3D	3.21	0.0000
227126_at	protein tyrosine phosphatase, receptor type, G	PTPRG	3.76	0.0000
202919_at	MOB family member 4, phocein	MOB4	3.76	0.0000
207747_s_at	docking protein 4	DOK4	7.87	0.0000
219062_s_at	zinc finger, CCHC domain containing 2	ZCCHC2	2.64	0.0000
226402_at	cytochrome P450, family 2, subfamily U, polypeptide 1	CYP2U1	6.08	0.0000
205856_at	solute carrier family 14 (urea transporter), member 1 (Kidd blood group)	SLC14A1	12.18	0.0000
229975_at	bone morphogenetic protein receptor, type IB	BMPR1B	2.19	0.0000
206300_s_at	parathyroid hormone-like hormone	PTHLH	2.82	0.0000
220119_at	erythrocyte membrane protein band 4.1 like 4A	EPB41L4A	2.97	0.0000
204519_s_at	plasmolipin	PLLP	2.37	0.0000
226795_at	leucine-rich repeats and calponin homology (CH) domain containing 1	LRCH1	2.95	0.0000
238907_at	zinc finger protein 780A	ZNF780A	2.53	0.0000
212612_at	REST corepressor 1	RCOR1	2.37	0.0000
205882_x_at	adducin 3 (gamma)	ADD3	3.62	0.0000
224844_at	SLAIN motif family, member 2	SLAIN2	4.29	0.0000
202363_at	sparc/osteonectin, cwcv and kazal-like domains proteoglycan (testican) 1	SPOCK1	2.96	0.0000
225180_at	tetratricopeptide repeat domain 14	TTC14	2.35	0.0000
223318_s_at	alkB, alkylation repair homolog 7 (E. coli)	ALKBH7	2.43	0.0000
218454_at	phospholipase B domain containing 1	PLBD1	3.01	0.0000
213421_x_at	protease, serine, 3	PRSS3	6.70	0.0000
1555486_a_at	proline rich 5 like	PRR5L	3.43	0.0000
227873_at	thioredoxin domain containing 15	TXNDC15	2.17	0.0000
206385_s_at	ankyrin 3, node of Ranvier (ankyrin G)	ANK3	6.20	0.0000
214440_at	N-acetyltransferase 1 (arylamine N-acetyltransferase)	NAT1	2.31	0.0000
203217_s_at	ST3 beta-galactoside alpha-2,3-sialyltransferase 5	ST3GAL5	2.04	0.0000
238445_x_at	mannosyl (alpha-1,6-)-glycoprotein beta-1,6-N-acetyl-glucosaminyltransferase, isozyme B	MGAT5B	2.82	0.0000
204518_s_at	peptidylprolyl isomerase C (cyclophilin C)	PPIC	2.53	0.0000

Probeset ID	Gene title	Gene symbol	Fold change	FDR (%)
224850_at	ATPase family, AAA domain containing 1	ATAD1	2.06	0.0000
238078_at	SEC22 vesicle trafficking protein homolog A (S. cerevisiae)	SEC22A	2.41	0.0000
215001_s_at	glutamate-ammonia ligase	GLUL	2.00	0.0000
203908_at	solute carrier family 4, sodium bicarbonate cotransporter, member 4	SLC4A4	3.73	0.0000
209530_at	calcium channel, voltage-dependent, beta 3 subunit	CACNB3	2.47	0.0000
212120_at	ras homolog family member Q	RHOQ	2.10	0.0000
226390_at	StAR-related lipid transfer (START) domain containing 4	STARD4	3.48	0.0000
225237_s_at	musashi homolog 2 (Drosophila)	MSI2	2.69	0.0000
226433_at	ring finger protein 157	RNF157	2.61	0.0000
217863_at	protein inhibitor of activated STAT, 1	PIAS1	2.42	0.0000
205794_s_at	neuro-oncological ventral antigen 1	NOVA1	3.44	0.0000
228904_at	homeobox B3	HOXB3	2.47	0.0000
226736_at	churchill domain containing 1	CHURC1	3.40	0.0000
229744_at	sperm specific antigen 2	SSFA2	2.23	0.0000
211202_s_at	lysine (K)-specific demethylase 5B	KDM5B	2.63	0.0000
209124_at	myeloid differentiation primary response gene (88)	MYD88	2.41	0.0000
224970_at	nuclear factor I/A	NFIA	2.00	0.0000
205802_at	transient receptor potential cation channel, subfamily C, member 1	TRPC1	2.46	0.0000
241741_at	cardiolipin synthase 1	CRLS1	4.08	0.0000
203827_at	WD repeat domain, phosphoinositide interacting 1	WIPI1	26.50	0.0000
201057_s_at	golgin B1	GOLGB1	2.17	0.0000
209518_at	SWI/SNF related, matrix associated, actin dependent regulator of chromatin, subfamily d, member 1	SMARCD1	2.14	0.0000
205289_at	bone morphogenetic protein 2	BMP2	2.62	0.0000
202893_at	unc-13 homolog B (C. elegans)	UNC13B	3.24	0.0000
225603_s_at	triple QxxK/R motif containing	TRIQK	3.14	0.0000
201941_at	carboxypeptidase D	CPD	2.09	0.0000
213225_at	protein phosphatase, Mg2+/Mn2+ dependent, 1B	PPM1B	3.17	0.0000
209815_at	patched 1	PTCH1	3.29	0.0000
202051_s_at	zinc finger, MYM-type 4	ZMYM4	2.11	0.0000
208806_at	chromodomain helicase DNA binding protein 3	CHD3	6.02	0.0000
227584_at	neuron navigator 1	NAV1	3.06	0.0000
205022_s_at	forkhead box N3	FOXN3	2.71	0.0000
200998_s_at	cytoskeleton-associated protein 4	CKAP4	2.14	0.0000
200602_at	amyloid beta (A4) precursor protein	APP	2.53	0.0000
200648_s_at	glutamate-ammonia ligase	GLUL	2.08	0.0000
222693_at	fibronectin type III domain containing 3B	FNDC3B	2.74	0.0000
202181_at	KIAA0247	KIAA0247	2.50	0.0000
212386_at	transcription factor 4	TCF4	4.63	0.0000
226776_at	enhancer of yellow 2 homolog (Drosophila)	ENY2	3.98	0.0000
229053_at	synaptotagmin XVII	SYT17	3.24	0.0000
221497_x_at	egl nine homolog 1 (C. elegans)	EGLN1	2.11	0.0000
227193_at	solute carrier family 30 (zinc transporter), member 4	SLC30A4	3.59	0.0000
218518_at	family with sequence similarity 13, member B	FAM13B	3.09	0.0000
227895_at	family with sequence similarity 120B	FAM120B	2.18	0.0000
238868_at	uveal autoantigen with coiled-coil domains and ankyrin repeats	UACA	6.71	0.0000
226986_at	WD repeat domain, phosphoinositide interacting 2	WIPI2	3.54	0.0000
232360_at	ets homologous factor	EHF	2.16	0.0000
204201_s_at	protein tyrosine phosphatase, non-receptor type 13 (APO-1/CD95 (Fas)-associated phosphatase)	PTPN13	2.93	0.0000
201212_at	legumain	LGMN	2.25	0.0000
228537_at	GLI family zinc finger 2	GLI2	2.07	0.0000
219181_at	lipase, endothelial	LIPG	11.13	0.0000
224793_s_at	transforming growth factor, beta receptor 1	TGFBR1	2.38	0.0000
204719_at	ATP-binding cassette, sub-family A (ABC1), member 8	ABCA8	5.14	0.0000
1557049_at	BTB (POZ) domain containing 19	BTBD19	5.86	0.0000

Probeset ID	Gene title	Gene symbol	Fold change	FDR (%)
207069_s_at	SMAD family member 6	SMAD6	2.39	0.0000
226847_at	follistatin	FST	3.39	0.0000
238323_at	TEA domain family member 2	TEAD2	7.88	0.0000
201549_x_at	lysine (K)-specific demethylase 5B	KDM5B	2.78	0.0000
207549_x_at	CD46 molecule, complement regulatory protein	CD46	2.26	0.0000
209566_at	insulin induced gene 2	INSIG2	2.34	0.0000
230492_s_at	glycerophosphocholine phosphodiesterase GDE1 homolog (S. cerevisiae)	GPCPD1	2.51	0.0000
202211_at	ADP-ribosylation factor GTPase activating protein 3	ARFGAP3	2.36	0.0000
230109_at	phosphodiesterase 7B	PDE7B	2.32	0.0000
209212_s_at	Kruppel-like factor 5 (intestinal)	KLF5	2.84	0.0000
220084_at	chromosome 14 open reading frame 105	C14orf105	4.27	0.0000
200904_at	major histocompatibility complex, class I, E	HLA-E	4.11	0.0000
221810_at	RAB15, member RAS oncogene family	RAB15	3.22	0.0000
222146_s_at	transcription factor 4	TCF4	3.93	0.0000
225946_at	Ras association (RalGDS/AF-6) domain family (N-terminal) member 8	RASSF8	2.60	0.0000
201561_s_at	calsyntenin 1	CLSTN1	2.20	0.0000
201484_at	suppressor of Ty 4 homolog 1 (S. cerevisiae)	SUPT4H1	2.73	0.0000
226806_s_at	nuclear factor I/A	NFIA	3.10	0.0000
231870_s_at	NMD3 homolog (S. cerevisiae)	NMD3	2.35	0.0000
224652_at	cyclin Y	CCNY	2.97	0.0000
45526_g_at	N(alpha)-acetyltransferase 60, NatF catalytic subunit	NAA60	2.00	0.0000
1555175_a_at	phenazine biosynthesis-like protein domain containing	PBLD	3.62	0.0000
205992_s_at	interleukin 15	IL15	2.76	0.0000
226633_at	RAB8B, member RAS oncogene family	RAB8B	2.48	0.0000
201430_s_at	dihydropyrimidinase-like 3	DPYSL3	3.26	0.0000
220945_x_at	MANSC domain containing 1	MANSC1	2.02	0.0000
208581_x_at	metallothionein 1X	MT1X	3.27	0.0000
214085_x_at	GLI pathogenesis-related 1	GLIPR1	3.47	0.0000
228293_at	DEP domain containing 7	DEPDC7	2.29	0.0000
228920_at	zinc finger protein 260	ZNF260	2.05	0.0000
202497_x_at	solute carrier family 2 (facilitated glucose transporter), member 3	SLC2A3	2.03	0.0000
237252_at	thrombomodulin	THBD	2.60	0.0000
208978_at	cysteine-rich protein 2	CRIP2	2.44	0.0000
203325_s_at	collagen, type V, alpha 1	COL5A1	2.57	0.0000
202745_at	ubiquitin specific peptidase 8	USP8	2.16	0.0000
211547_s_at	platelet-activating factor acetylhydrolase 1b, regulatory subunit 1 (45kDa)	PAFAH1B1	2.30	0.0000
224810_s_at	ankyrin repeat domain 13A	ANKRD13A	4.05	0.0000
208926_at	sialidase 1 (lysosomal sialidase)	NEU1	2.18	0.0000
212259_s_at	pre-B-cell leukemia homeobox interacting protein 1	PBXIP1	3.15	0.0000
224797_at	arrestin domain containing 3	ARRDC3	2.34	0.0000
208848_at	alcohol dehydrogenase 5 (class III), chi polypeptide	ADH5	2.10	0.0000
209538_at	zinc finger protein 32	ZNF32	2.51	0.0000
203896_s_at	phospholipase C, beta 4	PLCB4	3.06	0.0000
207318_s_at	cyclin-dependent kinase 13	CDK13	2.02	0.0000
209515_s_at	RAB27A, member RAS oncogene family	RAB27A	2.52	0.0000
212489_at	collagen, type V, alpha 1	COL5A1	2.51	0.0000
207606_s_at	Rho GTPase activating protein 12	ARHGAP12	2.06	0.0000
214745_at	phospholipase C, eta 1	PLCH1	3.03	0.0000
238867_at	transmembrane protein 182	TMEM182	4.36	0.0000
238554_at	cytochrome b5 type B (outer mitochondrial membrane)	CYB5B	7.87	0.0000
210946_at	phosphatidic acid phosphatase type 2A	PPAP2A	2.05	0.0000
200678_x_at	granulin	GRN	2.04	0.0000
229331_at	spermatogenesis associated 18	SPATA18	2.34	0.0000
224977_at	chromosome 6 open reading frame 89	C6orf89	2.20	0.0000
222731_at	zinc finger, DHHC-type containing 2	ZDHHC2	2.01	0.0000

Probeset ID	Gene title	Gene symbol	Fold change	FDR (%)
200816_s_at	platelet-activating factor acetylhydrolase 1b, regulatory subunit 1 (45kDa)	PAFAH1B1	2.13	0.0000
213725_x_at	xylosyltransferase I	XYLT1	2.40	0.0000
209488_s_at	RNA binding protein with multiple splicing	RBPMS	2.02	0.0000
216250_s_at	leupaxin	LPXN	3.57	0.0000
220180_at	coiled-coil domain containing 68	CCDC68	2.65	0.0000
212900_at	SEC24 family, member A (S. cerevisiae)	SEC24A	2.14	0.0000
238816_at	presenilin 1	PSEN1	3.31	0.0000
204421_s_at	fibroblast growth factor 2 (basic)	FGF2	2.13	0.0000
226150_at	phosphatidic acid phosphatase type 2 domain containing 1B	PPAPDC1B	2.01	0.0000
225240_s_at	musashi homolog 2 (Drosophila)	MSI2	2.58	0.0000
211456_x_at	metallothionein 1 pseudogene 2	MT1P2	2.61	0.0000
210729_at	neuropeptide Y receptor Y2	NPY2R	7.62	0.0000
201369_s_at	zinc finger protein 36, C3H type-like 2	ZFP36L2	2.22	0.0000
218434_s_at	acetoacetyl-CoA synthetase	AACS	2.03	0.0000
233917_s_at	Mov10, Moloney leukemia virus 10, homolog (mouse)	MOV10	2.66	0.0000
50221_at	transcription factor EB	TFEB	2.56	0.0000
227202_at	contactin 1	CNTN1	3.45	0.0000
238020_at	proteasome (prosome, macropain) 26S subunit, ATPase, 2	PSMC2	2.74	0.0000
202897_at	signal-regulatory protein alpha	SIRPA	2.33	0.0000
219197_s_at	signal peptide, CUB domain, EGF-like 2	SCUBE2	2.83	0.0000
202289_s_at	transforming, acidic coiled-coil containing protein 2	TACC2	2.09	0.0000
223849_s_at	Mov10, Moloney leukemia virus 10, homolog (mouse)	MOV10	3.84	0.0000
201877_s_at	protein phosphatase 2, regulatory subunit B', gamma	PPP2R5C	2.06	0.0000
212229_s_at	F-box protein 21	FBXO21	2.02	0.0000
228569_at	poly(A) polymerase alpha	PAPOLA	2.37	0.0000
213223_at	ribosomal protein L28	RPL28	2.80	0.0000
203037_s_at	metastasis suppressor 1	MTSS1	4.01	0.0000
206582_s_at	G protein-coupled receptor 56	GPR56	3.77	0.0000
231851_at	ribonucleoprotein, PTB-binding 2	RAVER2	4.04	0.0000
214268_s_at	myotubularin related protein 4	MTMR4	2.45	0.0000
225876_at	NIPA-like domain containing 3	NIPAL3	2.82	0.0000
235976_at	SLIT and NTRK-like family, member 6	SLITRK6	19.22	0.0000
226485_at	V-set and immunoglobulin domain containing 10	VSIG10	2.25	0.0000
238002_at	golgi integral membrane protein 4	GOLIM4	2.28	0.0000
228391_at	cytochrome P450, family 4, subfamily V, polypeptide 2	CYP4V2	7.42	0.0000
206087_x_at	hemochromatosis	HFE	2.40	0.0000
223312_at	protease-associated domain containing 1	PRADC1	6.13	0.0000
239650_at	NCK-associated protein 5	NCKAP5	4.36	0.0000
219806_s_at	chromosome 11 open reading frame 75	C11orf75	2.53	0.0000
213839_at	calmin (calponin-like, transmembrane)	CLMN	2.20	0.0000
226393_at	cytochrome P450, family 2, subfamily U, polypeptide 1	CYP2U1	3.03	0.0000
215399_s_at	osteosarcoma amplified 9, endoplasmic reticulum lectin	OS9	2.05	0.0000
235227_at	syntaxin binding protein 5 (tomosyn)	STXBP5	11.16	0.0000
227769_at	G protein-coupled receptor 27	GPR27	2.70	0.0000
217844_at	CTD (carboxy-terminal domain, RNA polymerase II, polypeptide A) small phosphatase 1	CTDSP1	2.28	0.0000
213143_at	chromosome 2 open reading frame 72	C2orf72	2.32	0.0000
230369_at	G protein-coupled receptor 161	GPR161	5.25	0.0000
222294_s_at	RAB27A, member RAS oncogene family	RAB27A	5.50	0.0000
204073_s_at	chromosome 11 open reading frame 9	C11orf9	2.27	0.0000
206858_s_at	homeobox C6	HOXC6	2.23	0.0000
204489_s_at	CD44 molecule (Indian blood group)	CD44	2.20	0.0000
205425_at	huntingtin interacting protein 1	HIP1	2.84	0.0000
225502_at	dedicator of cytokinesis 8	DOCK8	4.17	0.0000
203895_at	phospholipase C, beta 4	PLCB4	2.35	0.0000
229145_at	anaphase promoting complex subunit 16	ANAPC16	2.68	0.0000

Probeset ID	Gene title	Gene symbol	Fold change	FDR (%)
204567_s_at	ATP-binding cassette, sub-family G (WHITE), member 1	ABCG1	7.08	0.0000
1553994_at	5'-nucleotidase, ecto (CD73)	NT5E	2.66	0.0000
235787_at	cell division cycle 37 homolog (S. cerevisiae)-like 1	CDC37L1	3.39	0.0000
205302_at	insulin-like growth factor binding protein 1	IGFBP1	3.38	0.0000
204217_s_at	reticulon 2	RTN2	3.86	0.0000
204401_at	potassium intermediate/small conductance calcium-activated channel, subfamily N, member 4	KCNN4	2.81	0.0000
219029_at	chromosome 5 open reading frame 28	C5orf28	3.24	0.0000
212185_x_at	metallothionein 2A	MT2A	2.27	0.0000
1553734_at	adenylate kinase 7	AK7	2.52	0.0000
225019_at	calcium/calmodulin-dependent protein kinase II delta	CAMK2D	2.17	0.0000
201842_s_at	EGF containing fibulin-like extracellular matrix protein 1	EFEMP1	2.46	0.0000
216526_x_at	major histocompatibility complex, class I, C	HLA-C	2.32	0.0000
224983_at	scavenger receptor class B, member 2	SCARB2	2.06	0.0000
241898_at	lipase, member H	LIPH	4.02	0.0000
218311_at	mitogen-activated protein kinase kinase kinase kinase 3	MAP4K3	2.04	0.0000
220355_s_at	polybromo 1	PBRM1	2.01	0.0000
230056_at	bromodomain PHD finger transcription factor	BPTF	4.89	0.0000
208920_at	sorcin	SRI	3.62	0.0000
1555058_a_at	lysophosphatidylglycerol acyltransferase 1	LPGAT1	2.36	0.0000
227585_at	ATPase family, AAA domain containing 1	ATAD1	5.33	0.0000
212122_at	ras homolog family member Q	RHOQ	2.40	0.0000
212299_at	NIMA (never in mitosis gene a)- related kinase 9	NEK9	2.21	0.0000
224772_at	neuron navigator 1	NAV1	4.47	0.0000
207076_s_at	argininosuccinate synthase 1	ASS1	3.12	0.0000
219858_s_at	major facilitator superfamily domain containing 6	MFSD6	2.55	0.0000
225775_at	tetraspanin 33	TSPAN33	3.01	0.0000
1558279_a_at	3-ketodihydrosphingosine reductase	KDSR	2.51	0.0000
204745_x_at	metallothionein 1G	MT1G	2.10	0.0000
200677_at	pituitary tumor-transforming 1 interacting protein	PTTG1IP	2.33	0.0000
210665_at	tissue factor pathway inhibitor (lipoprotein-associated coagulation inhibitor)	TFPI	3.28	0.0000
225390_s_at	Kruppel-like factor 13	KLF13	2.17	0.0000
208965_s_at	interferon, gamma-inducible protein 16	IFI16	8.44	0.0000
1552275_s_at	PX domain containing serine/threonine kinase	PXK	2.19	0.0000
226511_at	DDB1 and CUL4 associated factor 10	DCAF10	2.36	0.0000
209189_at	FBJ murine osteosarcoma viral oncogene homolog	FOS	5.25	0.0000
205234_at	solute carrier family 16, member 4 (monocarboxylic acid transporter 5)	SLC16A4	2.34	0.0000
209468_at	low density lipoprotein receptor-related protein 5	LRP5	2.20	0.0000
228849_at	neurotrophic tyrosine kinase, receptor, type 3	NTRK3	3.75	0.0000
231880_at	family with sequence similarity 40, member B	FAM40B	3.50	0.0000
205601_s_at	homeobox B5	HOXB5	2.76	0.0000
218396_at	vacuolar protein sorting 13 homolog C (S. cerevisiae)	VPS13C	2.11	0.0000
212775_at	obscurin-like 1	OBSL1	2.83	0.0000
238649_at	phosphatidylinositol transfer protein, cytoplasmic 1	PITPNC1	3.46	0.0000
209283_at	crystallin, alpha B	CRYAB	2.65	0.0000
212614_at	AT rich interactive domain 5B (MRF1-like)	ARID5B	5.73	0.0000
207808_s_at	protein S (alpha)	PROS1	3.05	0.0000
232053_x_at	rhomboid domain containing 2	RHBDD2	2.01	0.0000
214306_at	optic atrophy 1 (autosomal dominant)	OPA1	2.03	0.0000
217456_x_at	major histocompatibility complex, class I, E	HLA-E	2.23	0.0000
225600_at	triple QxxK/R motif containing	TRIQK	3.05	0.0000
222916_s_at	high density lipoprotein binding protein	HDLBP	2.69	0.0000
202269_x_at	guanylate binding protein 1, interferon-inducible	GBP1	3.03	0.0000
218031_s_at	forkhead box N3	FOXN3	15.26	0.0000
34858_at	potassium channel tetramerisation domain containing 2	KCTD2	2.29	0.0000
214778_at	multiple EGF-like-domains 8	MEGF8	2.12	0.0000

Probeset ID	Gene title	Gene symbol	Fold change	FDR (%)
213010_at	protein kinase C, delta binding protein	PRKCDBP	3.82	0.0000
227382_at	cytochrome b5 type B (outer mitochondrial membrane)	CYB5B	5.17	0.0000
200621_at	cysteine and glycine-rich protein 1	CSRP1	2.06	0.0000
229313_at	anoctamin 5	ANO5	2.92	0.0000
202367_at	cut-like homeobox 1	CUX1	2.34	0.0000
222462_s_at	beta-site APP-cleaving enzyme 1	BACE1	5.16	0.0000
222764_at	asparaginase like 1	ASRGL1	2.58	0.0000
202765_s_at	fibrillin 1	FBN1	8.40	0.0000
202000_at	NADH dehydrogenase (ubiquinone) 1 alpha subcomplex, 6, 14kDa	NDUFA6	2.62	0.0000
221103_s_at	WD repeat domain 52	WDR52	3.45	0.0000
209102_s_at	HMG-box transcription factor 1	HBP1	2.76	0.0000
243864_at	coiled-coil domain containing 80	CCDC80	3.75	0.0000
209487_at	RNA binding protein with multiple splicing	RBPMS	2.12	0.0000
218036_x_at	NMD3 homolog (S. cerevisiae)	NMD3	2.14	0.0000
225173_at	Rho GTPase activating protein 18	ARHGAP18	2.83	0.0000
222804_x_at	DDB1 and CUL4 associated factor 10	DCAF10	2.17	0.0000
203394_s_at	hairy and enhancer of split 1, (Drosophila)	HES1	3.65	0.0000
227417_at	mitochondrial amidoxime reducing component 2	MARC2	4.35	0.0000
204505_s_at	erythrocyte membrane protein band 4.9 (dematin)	EPB49	2.14	0.0000
230291_s_at	Nuclear factor I/B	NFIB	2.02	0.0000
212293_at	homeodomain interacting protein kinase 1	HIPK1	2.22	0.0000
218706_s_at	GRAM domain containing 3	GRAMD3	5.37	0.0000
204091_at	phosphodiesterase 6D, cGMP-specific, rod, delta	PDE6D	2.09	0.0000
217168_s_at	homocysteine-inducible, endoplasmic reticulum stress-inducible, ubiquitin-like domain member 1	HERPUD1	2.04	0.0000
214544_s_at	synaptosomal-associated protein, 23kDa	SNAP23	2.03	0.0000
201634_s_at	cytochrome b5 type B (outer mitochondrial membrane)	CYB5B	2.03	0.0000
204115_at	guanine nucleotide binding protein (G protein), gamma 11	GNG11	2.02	0.0000
235561_at	thioredoxin-like 1	TXNL1	4.32	0.0000
226590_at	zinc finger protein 618	ZNF618	2.89	0.0000
209835_x_at	CD44 molecule (Indian blood group)	CD44	2.18	0.0000
225012_at	high density lipoprotein binding protein	HDLBP	2.50	0.0000
203035_s_at	protein inhibitor of activated STAT, 3	PIAS3	2.31	0.0000
205383_s_at	zinc finger and BTB domain containing 20	ZBTB20	2.28	0.0000
214920_at	thrombospondin, type I, domain containing 7A	THSD7A	3.02	0.0000
227642_at	transcription factor CP2-like 1	TFCP2L1	3.64	0.0000
226063_at	vav 2 guanine nucleotide exchange factor	VAV2	2.50	0.0000
218086_at	neural proliferation, differentiation and control, 1	NPDC1	2.76	0.0000
228437_at	cornichon homolog 4 (Drosophila)	CNIH4	2.08	0.0000
210916_s_at	CD44 molecule (Indian blood group)	CD44	2.09	0.0000
202080_s_at	trafficking protein, kinesin binding 1	TRAK1	2.04	0.0000
225509_at	SAP30-like	SAP30L	2.98	0.0000
202772_at	3-hydroxymethyl-3-methylglutaryl-CoA lyase	HMGCL	2.11	0.0000
219338_s_at	leucine rich repeat containing 49	LRRC49	2.08	0.0000
225613_at	microtubule associated serine/threonine kinase family member 4	MAST4	5.95	0.0000
203423_at	retinol binding protein 1, cellular	RBP1	2.87	0.0000
204159_at	cyclin-dependent kinase inhibitor 2C (p18, inhibits CDK4)	CDKN2C	2.48	0.0000
231406_at	ORAI calcium release-activated calcium modulator 2	ORAI2	2.18	0.0000
229997_at	vang-like 1 (van gogh, Drosophila)	VANGL1	2.05	0.0000
243198_at	testis expressed 9	TEX9	2.96	0.0000
227327_at	multiple EGF-like-domains 8	MEGF8	2.46	0.0000
226607_at	chromosome 20 open reading frame 194	C20orf194	4.66	0.0000
212014_x_at	CD44 molecule (Indian blood group)	CD44	2.22	0.0000
212423_at	zinc finger, CCHC domain containing 24	ZCCHC24	3.22	0.0000
224744_at	inositol monophosphatase domain containing 1	IMPAD1	2.07	0.0000
225558_at	G protein-coupled receptor kinase interacting ArfGAP 2	GIT2	2.17	0.0000

Probeset ID	Gene title	Gene symbol	Fold change	FDR (%)
200714_x_at	osteosarcoma amplified 9, endoplasmic reticulum lectin	OS9	2.01	0.0000
222925_at	doublecortin domain containing 2	DCDC2	2.18	0.0000
206818_s_at	cyclin M2	CNNM2	5.25	0.0000
203364_s_at	autophagy related 13	ATG13	2.08	0.0000
201983_s_at	epidermal growth factor receptor	EGFR	5.40	0.0000
229159_at	thrombospondin, type I, domain containing 7A	THSD7A	3.72	0.0000
219001_s_at	DDB1 and CUL4 associated factor 10	DCAF10	2.80	0.0000
201743_at	CD14 molecule	CD14	3.28	0.0000
225516_at	solute carrier family 7 (cationic amino acid transporter, y+ system), member 2	SLC7A2	2.15	0.0000
224773_at	neuron navigator 1	NAV1	2.47	0.0000
202499_s_at	solute carrier family 2 (facilitated glucose transporter), member 3	SLC2A3	2.07	0.0000
200948_at	myeloid leukemia factor 2	MLF2	2.01	0.0000
217864_s_at	protein inhibitor of activated STAT, 1	PIAS1	2.11	0.0000
229657_at	thyroid hormone receptor, beta	THRB	2.92	0.0000
212812_at	serine incorporator 5	SERINC5	2.04	0.0000
205609_at	angiopoietin 1	ANGPT1	4.40	0.0000
226281_at	delta/notch-like EGF repeat containing	DNER	5.19	0.0000
212034_s_at	exocyst complex component 7	EXOC7	2.04	0.0000
209230_s_at	nuclear protein, transcriptional regulator, 1	NUPR1	2.78	0.0000
217564_s_at	carbamoyl-phosphate synthase 1, mitochondrial	CPS1	2.41	0.0000
209598_at	paraneoplastic Ma antigen 2	PNMA2	2.44	0.0000
230061_at	transmembrane 4 L six family member 18	TM4SF18	2.21	0.0000
212427_at	KIAA0368	KIAA0368	2.13	0.0000
218464_s_at	family with sequence similarity 222, member B	FAM222B	2.28	0.0000
218084_x_at	FXYD domain containing ion transport regulator 5	FXYD5	2.90	0.0000
225123_at	sestrin 3	SESN3	4.06	0.0000
221216_s_at	sex comb on midleg homolog 1 (Drosophila)	SCMH1	2.28	0.0000
1555962_at	UDP-GlcNAc:betaGal beta-1,3-N-acetylglucosaminyltransferase 7	B3GNT7	2.04	0.0000
230100_x_at	p21 protein (Cdc42/Rac)-activated kinase 1	PAK1	3.65	0.0000
229603_at	Bardet-Biedl syndrome 12	BBS12	2.69	0.0000
225407_at	myelin basic protein	MBP	2.02	0.0000
204206_at	MAX binding protein	MNT	2.10	0.0000
218522_s_at	microtubule-associated protein 1S	MAP1S	2.42	0.0000
225598_at	solute carrier family 45, member 4	SLC45A4	2.96	0.0000
207722_s_at	BTB (POZ) domain containing 2	BTBD2	2.26	0.0000
227660_at	anthrax toxin receptor 1	ANTXR1	2.46	0.0000
228561_at	cell division cycle 37 homolog (S. cerevisiae)-like 1	CDC37L1	2.13	0.0000
202969_at	dual-specificity tyrosine-(Y)-phosphorylation regulated kinase 2	DYRK2	2.15	0.0000
225411_at	transmembrane protein 87B	TMEM87B	6.16	0.0000
210705_s_at	tripartite motif containing 5	TRIM5	2.68	0.0000
243010_at	musashi homolog 2 (Drosophila)	MSI2	4.61	0.0000
202481_at	dehydrogenase/reductase (SDR family) member 3	DHRS3	2.17	0.0000
201915_at	SEC63 homolog (S. cerevisiae)	SEC63	2.03	0.0000
234936_s_at	coiled-coil and C2 domain containing 2A	CC2D2A	2.19	0.0000
224252_s_at	FXYD domain containing ion transport regulator 5	FXYD5	2.85	0.0000
241412_at	betacellulin	BTC	5.24	0.0000
221958_s_at	wntless homolog (Drosophila)	WLS	2.17	0.0000
226777_at	ADAM metallopeptidase domain 12	ADAM12	2.16	0.0000
213587_s_at	ATPase, H+ transporting V0 subunit e2	ATP6V0E2	2.23	0.0000
203813_s_at	slit homolog 3 (Drosophila)	SLIT3	2.28	0.0000
204679_at	potassium channel, subfamily K, member 1	KCNK1	2.30	0.0000
204796_at	echinoderm microtubule associated protein like 1	EML1	3.31	0.0000
203998_s_at	synaptotagmin I	SYT1	2.98	0.0000
202103_at	bromodomain containing 4	BRD4	3.48	0.0000
1555963_x_at	UDP-GlcNAc:betaGal beta-1,3-N-acetylglucosaminyltransferase 7	B3GNT7	2.87	0.0000

Probeset ID	Gene title	Gene symbol	Fold change	FDR (%)
228987_at	family with sequence similarity 49, member B	FAM49B	2.62	0.0000
219194_at	sema domain, immunoglobulin domain (Ig), transmembrane domain (TM) and short cytoplasmic domain, (semaphorin) 4G	SEMA4G	3.76	0.0000
217731_s_at	integral membrane protein 2B	ITM2B	2.14	0.0000
226164_x_at	ribosomal modification protein rimK-like family member B	RIMKLB	2.51	0.0000
225338_at	zyg-11 homolog B (C. elegans)	ZYG11B	2.27	0.0000
217921_at	mannosidase, alpha, class 1A, member 2	MAN1A2	2.17	0.0000
221249_s_at	family with sequence similarity 117, member A	FAM117A	2.31	0.0000
204140_at	tyrosylprotein sulfotransferase 1	TPST1	2.36	0.0000
214672_at	tubulin tyrosine ligase-like family, member 5	TTLL5	2.15	0.0000
205952_at	potassium channel, subfamily K, member 3	KCNK3	12.10	0.0000
225872_at	solute carrier family 35, member F5	SLC35F5	2.05	0.0000
232079_s_at	poliovirus receptor-related 2 (herpesvirus entry mediator B)	PVRL2	2.09	0.0000
207847_s_at	mucin 1, cell surface associated	MUC1	2.66	0.0000
231793_s_at	calcium/calmodulin-dependent protein kinase II delta	CAMK2D	2.37	0.0000
205649_s_at	fibrinogen alpha chain	FGA	4.26	0.0000
224477_s_at	nudix (nucleoside diphosphate linked moiety X)-type motif 16-like 1	NUDT16L1	2.13	0.0000
228070_at	protein phosphatase 2, regulatory subunit B', epsilon isoform	PPP2R5E	6.56	0.0000
204308_s_at	tectonin beta-propeller repeat containing 2	TECPR2	2.29	0.0000
242056_at	tripartite motif containing 45	TRIM45	2.43	0.0000
204612_at	protein kinase (cAMP-dependent, catalytic) inhibitor alpha	PKIA	5.13	0.0000
212462_at	K(lysine) acetyltransferase 6B	KAT6B	2.15	0.0000
223434_at	guanylate binding protein 3	GBP3	13.03	0.0000
212660_at	PHD finger protein 15	PHF15	2.15	0.0000
219564_at	potassium inwardly-rectifying channel, subfamily J, member 16	KCNJ16	13.73	0.0000
211578_s_at	ribosomal protein S6 kinase, 70kDa, polypeptide 1	RPS6KB1	2.05	0.0000
202125_s_at	trafficking protein, kinesin binding 2	TRAK2	2.03	0.0000
226713_at	coiled-coil domain containing 50	CCDC50	2.52	0.0000
204667_at	forkhead box A1	FOXA1	2.16	0.0000
228531_at	sterile alpha motif domain containing 9	SAMD9	2.30	0.0000
204165_at	WAS protein family, member 1	WASF1	2.00	0.0000
201723_s_at	UDP-N-acetyl-alpha-D-galactosamine:polypeptide N-acetylgalactosaminyltransferase 1 (GalNAc-T1)	GALNT1	2.11	0.0000
227001_at	NIPA-like domain containing 2	NIPAL2	2.69	0.0000
229588_at	DnaJ (Hsp40) homolog, subfamily C, member 10	DNAJC10	3.67	0.0000
203753_at	transcription factor 4	TCF4	2.96	0.0000
225666_at	transmembrane and tetratricopeptide repeat containing 4	TMTC4	2.14	0.0000
1554806_a_at	F-box protein 8	FBXO8	2.50	0.0000
202500_at	DnaJ (Hsp40) homolog, subfamily B, member 2	DNAJB2	3.73	0.0000
227878_s_at	alkB, alkylation repair homolog 7 (E. coli)	ALKBH7	2.57	0.0000
229838_at	nucleobindin 2	NUCB2	3.68	0.0000
202960_s_at	methylmalonyl CoA mutase	MUT	2.05	0.0000
209290_s_at	nuclear factor I/B	NFIB	2.31	0.0000
209289_at	nuclear factor I/B	NFIB	2.49	0.0000
239082_at	frizzled family receptor 3	FZD3	3.11	0.0000
226004_at	Cdk5 and Abl enzyme substrate 2	CABLES2	2.17	0.0000
241739_at	2-oxoglutarate and iron-dependent oxygenase domain containing 1	OGFOD1	2.16	0.0000
227863_at	interferon induced transmembrane protein 10	IFITM10	3.15	0.0000
224743_at	inositol monophosphatase domain containing 1	IMPAD1	2.02	0.0000
223541_at	hyaluronan synthase 3	HAS3	2.27	0.0000
224859_at	CD276 molecule	CD276	2.05	0.0000
210329_s_at	sarcoglycan, delta (35kDa dystrophin-associated glycoprotein)	SGCD	2.42	0.0000
222791_at	round spermatid basic protein 1	RSBN1	2.05	0.0000
238937_at	zinc finger protein 420	ZNF420	3.83	0.0000
209681_at	solute carrier family 19 (thiamine transporter), member 2	SLC19A2	2.10	0.0000
218589_at	lysophosphatidic acid receptor 6	LPAR6	3.00	0.0000

Probeset ID	Gene title	Gene symbol	Fold change	FDR (%)
218546_at	chromosome 1 open reading frame 115	C1orf115	2.36	0.0000
223492_s_at	leucine rich repeat (in FLII) interacting protein 1	LRRFIP1	2.63	0.0000
205560_at	proprotein convertase subtilisin/kexin type 5	PCSK5	3.06	0.0000
211256_x_at	butyrophilin, subfamily 2, member A1	BTN2A1	2.32	0.0000
203297_s_at	jumonji, AT rich interactive domain 2	JARID2	2.12	0.0000
226326_at	polycomb group ring finger 5	PCGF5	2.15	0.0000
227692_at	guanine nucleotide binding protein (G protein), alpha inhibiting activity polypeptide 1	GNAI1	2.14	0.0000
223220_s_at	poly (ADP-ribose) polymerase family, member 9	PARP9	4.13	0.0000
206179_s_at	tubulin polymerization promoting protein	TPPP	2.29	0.0000
210377_at	acyl-CoA synthetase medium-chain family member 3	ACSM3	3.60	0.0000
201067_at	proteasome (prosome, macropain) 26S subunit, ATPase, 2	PSMC2	2.97	0.0000
224638_at	signal peptide peptidase like 3	SPPL3	2.06	0.0000
218857_s_at	asparaginase like 1	ASRGL1	2.16	0.0000
212984_at	activating transcription factor 2	ATF2	2.19	0.0000
226158_at	kelch-like 24 (Drosophila)	KLHL24	8.97	0.0000
211255_x_at	death effector domain containing	DEDD	3.06	0.0000
230906_at	UDP-N-acetyl-alpha-D-galactosamine:polypeptide N-acetylgalactosaminyltransferase 10 (GalNAc-T10)	GALNT10	2.58	0.0000
225473_at	suppressor of glucose, autophagy associated 1	SOGA1	2.50	0.0000
1555812_a_at	Rho GDP dissociation inhibitor (GDI) beta	ARHGDIB	4.16	0.0000
203917_at	coxsackie virus and adenovirus receptor	CXADR	2.62	0.0000
205508_at	sodium channel, voltage-gated, type I, beta subunit	SCN1B	2.75	0.0000
239218_at	phosphodiesterase 1C, calmodulin-dependent 70kDa	PDE1C	5.61	0.0000
235390_at	SREK1-interacting protein 1	SREK1IP1	3.41	0.0000
235343_at	vasohibin 2	VASH2	2.24	0.0000
238030_at	zinc finger protein 268	ZNF268	2.86	0.0000
202572_s_at	discs, large (Drosophila) homolog-associated protein 4	DLGAP4	2.49	0.0000
210480_s_at	myosin VI	MYO6	2.62	0.0000
224674_at	tweety homolog 3 (Drosophila)	TTYH3	2.03	0.0000
229245_at	pleckstrin homology domain containing, family A member 6	PLEKHA6	5.88	0.0000
213848_at	dual specificity phosphatase 7	DUSP7	2.20	0.0000
217523_at	CD44 molecule (Indian blood group)	CD44	2.43	0.0000
238461_at	eukaryotic translation initiation factor 4E family member 3	EIF4E3	3.15	0.0000
212909_at	LY6/PLAUR domain containing 1	LYPD1	2.48	0.0000
238933_at	insulin receptor substrate 1	IRS1	2.78	0.0000
232603_at	doublecortin domain containing 5	DCDC5	3.56	0.0000
226774_at	family with sequence similarity 120B	FAM120B	2.93	0.0000
213624_at	sphingomyelin phosphodiesterase, acid-like 3A	SMPDL3A	2.26	0.0000
204646_at	dihydropyrimidine dehydrogenase	DPYD	2.37	0.0000
207265_s_at	KDEL (Lys-Asp-Glu-Leu) endoplasmic reticulum protein retention receptor 3	KDELR3	2.07	0.0000
235964_x_at	SAM domain and HD domain 1	SAMHD1	3.18	0.0000
205559_s_at	proprotein convertase subtilisin/kexin type 5	PCSK5	2.99	0.0000
232349_x_at	DDB1 and CUL4 associated factor 6	DCAF6	2.07	0.0000
201611_s_at	isoprenylcysteine carboxyl methyltransferase	ICMT	3.59	0.0000
204597_x_at	stanniocalcin 1	STC1	2.20	0.0000
211602_s_at	transient receptor potential cation channel, subfamily C, member 1	TRPC1	3.93	0.0000
227033_at	protein disulfide isomerase family A, member 3	PDIA3	2.00	0.0000
227055_at	methyltransferase like 7B	METTL7B	2.04	0.0000
226198_at	target of myb1-like 2 (chicken)	TOM1L2	2.72	0.0000
203395_s_at	hairy and enhancer of split 1, (Drosophila)	HES1	2.12	0.0000
225941_at	eukaryotic translation initiation factor 4E family member 3	EIF4E3	3.93	0.0000
209205_s_at	LIM domain only 4	LMO4	2.20	0.0000
239065_at	DnaJ (Hsp40) homolog, subfamily C, member 22	DNAJC22	3.63	0.0000
213333_at	malate dehydrogenase 2, NAD (mitochondrial)	MDH2	2.35	0.0000
203514_at	mitogen-activated protein kinase kinase kinase 3	MAP3K3	2.29	0.0000

Probeset ID	Gene title	Gene symbol	Fold change	FDR (%)
230243_at	tRNA methyltransferase 10 homolog A (S. cerevisiae)	TRMT10A	3.16	0.0000
224651_at	cyclin Y	CCNY	3.07	0.0000
227056_at	KIAA0141	KIAA0141	3.41	0.0000
219495_s_at	zinc finger protein 180	ZNF180	2.06	0.0000
205896_at	solute carrier family 22 (organic cation/ergothioneine transporter), member 4	SLC22A4	2.18	0.0000
220441_at	DnaJ (Hsp40) homolog, subfamily C, member 22	DNAJC22	2.08	0.0000
222455_s_at	parvin, alpha	PARVA	2.01	0.0000
233230_s_at	SLAIN motif family, member 2	SLAIN2	5.73	0.0000
223722_at	DnaJ (Hsp40) homolog, subfamily C, member 12	DNAJC12	2.29	0.0000
224727_at	ER membrane protein complex subunit 10	EMC10	2.05	0.0000
221636_s_at	mitochondrial amidoxime reducing component 2	MARC2	2.32	0.0000
230178_s_at	elongation protein 2 homolog (S. cerevisiae)	ELP2	2.18	0.0000
213029_at	nuclear factor I/B	NFIB	2.63	0.0000
238444_at	zinc finger protein 618	ZNF618	2.57	0.0000
225387_at	tetraspanin 5	TSPAN5	2.29	0.0000
227148_at	pleckstrin homology domain containing, family H (with MyTH4 domain) member 2	PLEKHH2	8.42	0.0000
1553112_s_at	cyclin-dependent kinase 8	CDK8	2.89	0.0000
1552329_at	retinoblastoma binding protein 6	RBBP6	2.74	0.0000
224489_at	KAT8 regulatory NSL complex subunit 1	KANSL1	3.08	0.0000
238877_at	eyes absent homolog 4 (Drosophila)	EYA4	2.14	0.0000
242579_at	bone morphogenetic protein receptor, type IB	BMPR1B	2.54	0.0000
1554784_at	contactin 1	CNTN1	2.04	0.0000
235890_at	transducin (beta)-like 1 X-linked receptor 1	TBL1XR1	3.24	0.0000
1558692_at	chromosome 1 open reading frame 85	C1orf85	2.63	0.0000
223594_at	transmembrane protein 117	TMEM117	2.14	0.0000
225020_at	DAB2 interacting protein	DAB2IP	2.47	0.0000
209292_at	inhibitor of DNA binding 4, dominant negative helix-loop-helix protein	ID4	2.54	0.0000
204039_at	CCAAT/enhancer binding protein (C/EBP), alpha	CEBPA	2.05	0.0000
209989_at	zinc finger protein 268	ZNF268	2.15	0.0000
232078_at	poliovirus receptor-related 2 (herpesvirus entry mediator B)	PVRL2	2.20	0.0000
225450_at	angiomotin like 1	AMOTL1	2.67	0.0000
209847_at	cadherin 17, LI cadherin (liver-intestine)	CDH17	2.62	0.0000
223608_at	EF-hand calcium binding domain 2	EFCAB2	2.13	0.0000
243816_at	Zinc finger protein 70	ZNF70	7.44	0.0000
1558142_at	trinucleotide repeat containing 6B	TNRC6B	2.68	0.0000
212338_at	myosin ID	MYO1D	2.88	0.0000
211329_x_at	hemochromatosis	HFE	3.13	0.0000
209710_at	GATA binding protein 2	GATA2	2.06	0.0000
228463_at	forkhead box A3	FOXA3	4.05	0.0000
226013_at	trafficking protein, kinesin binding 1	TRAK1	4.36	0.0000
223240_at	F-box protein 8	FBXO8	2.15	0.0000
228703_at	prolyl 4-hydroxylase, alpha polypeptide III	P4HA3	2.09	0.0000
211834_s_at	tumor protein p63	TP63	2.79	0.0000
212897_at	cyclin-dependent kinase 19	CDK19	2.19	0.0000
1558117_s_at	ubiquitin specific peptidase 31	USP31	2.33	0.0000
226292_at	calpain 5	CAPN5	2.71	0.0000
213280_at	RAP1 GTPase activating protein 2	RAP1GAP2	2.33	0.0000
218718_at	platelet derived growth factor C	PDGFC	2.20	0.0000
218362_s_at	DIS3 mitotic control homolog (S. cerevisiae)	DIS3	3.43	0.0000
215910_s_at	fibronectin type III domain containing 3A	FNDC3A	2.11	0.0000
225001_at	RAB3D, member RAS oncogene family	RAB3D	2.05	0.0000
231835_at	family with sequence similarity 213, member B	FAM213B	2.64	0.0000
226779_at	LMBR1 domain containing 2	LMBRD2	2.34	0.0000
1556051_a_at	bicaudal D homolog 1 (Drosophila)	BICD1	2.25	0.0000
224649_x_at	cyclin Y	CCNY	2.35	0.0000

Probeset ID	Gene title	Gene symbol	Fold change	FDR (%)
205547_s_at	transgelin	TAGLN	2.23	0.0000
235427_at	CASP8 and FADD-like apoptosis regulator	CFLAR	4.85	0.0000
208337_s_at	nuclear receptor subfamily 5, group A, member 2	NR5A2	2.38	0.0000
225796_at	PX domain containing serine/threonine kinase	PXK	2.72	0.0000
211852_s_at	attractin	ATRN	2.25	0.0000
228168_at	ATP synthase, H+ transporting, mitochondrial Fo complex, subunit C3 (subunit 9)	ATP5G3	3.04	0.0000
214433_s_at	selenium binding protein 1	SELENBP1	6.07	0.0000
228341_at	nudix (nucleoside diphosphate linked moiety X)-type motif 16	NUDT16	2.82	0.0000
217608_at	SREK1-interacting protein 1	SREK1IP1	2.11	0.0000
210105_s_at	FYN oncogene related to SRC, FGR, YES	FYN	3.89	0.0000
228890_at	atonal homolog 8 (Drosophila)	ATOH8	2.00	0.0000
233559_s_at	WD repeat and FYVE domain containing 1	WDFY1	2.52	0.0000
202430_s_at	phospholipid scramblase 1	PLSCR1	2.03	0.0000
238513_at	proline rich Gla (G-carboxyglutamic acid) 4 (transmembrane)	PRRG4	2.53	0.0000
222150_s_at	pigeon homolog (Drosophila)	PION	2.30	0.0000
223189_x_at	myeloid/lymphoid or mixed-lineage leukemia 5 (trithorax homolog, Drosophila)	MLL5	2.34	0.0000
231336_at	copine IV	CPNE4	3.69	0.0000
223093_at	ankylosis, progressive homolog (mouse)	ANKH	2.18	0.0000
230479_at	eukaryotic translation initiation factor 3, subunit F	EIF3F	3.78	0.0000
224975_at	nuclear factor I/A	NFIA	2.06	0.0000
204470_at	chemokine (C-X-C motif) ligand 1 (melanoma growth stimulating activity, alpha)	CXCL1	2.19	0.0000
227701_at	chromosome 10 open reading frame 118	C10orf118	2.01	0.0000
231008_at	unc-5 homolog C (C. elegans)-like	UNC5CL	3.38	0.0000
203741_s_at	adenylate cyclase 7	ADCY7	2.12	0.0000
217127_at	cystathionase (cystathionine gamma-lyase)	CTH	3.16	0.0000
227606_s_at	STAM binding protein-like 1	STAMBPL1	2.18	0.0000
214670_at	zinc finger with KRAB and SCAN domains 1	ZKSCAN1	2.46	0.0000
241355_at	hairless homolog (mouse)	HR	2.16	0.0000
228255_at	transmembrane protein 237	TMEM237	8.12	0.0000
209147_s_at	phosphatidic acid phosphatase type 2A	PPAP2A	2.05	0.0000
217967_s_at	family with sequence similarity 129, member A	FAM129A	2.17	0.0000
212382_at	transcription factor 4	TCF4	3.55	0.0000
227822_at	zinc finger protein 605	ZNF605	3.03	0.0000
207705_s_at	ninein-like	NINL	2.20	0.0000
222456_s_at	LIM domain and actin binding 1	LIMA1	2.93	0.0000
60471_at	Ras and Rab interactor 3	RIN3	2.01	0.0000
209131_s_at	synaptosomal-associated protein, 23kDa	SNAP23	2.25	0.0000
219352_at	HECT and RLD domain containing E3 ubiquitin protein ligase family member 6	HERC6	3.35	0.0000
201219_at	C-terminal binding protein 2	CTBP2	2.09	0.0000
228024_at	vacuolar protein sorting 37 homolog A (S. cerevisiae)	VPS37A	2.41	0.0000
202270_at	guanylate binding protein 1, interferon-inducible	GBP1	3.40	0.0000
203373_at	suppressor of cytokine signaling 2	SOCS2	2.20	0.0000
238222_at	gastrokine 2	GKN2	5.05	0.0000
227812_at	tumor necrosis factor receptor superfamily, member 19	TNFRSF19	3.11	0.0000
225935_at	cut-like homeobox 1	CUX1	2.12	0.0000
212736_at	chromosome 16 open reading frame 45	C16orf45	2.43	0.0000
224197_s_at	C1q and tumor necrosis factor related protein 1	C1QTNF1	4.71	0.0000
230679_at	DDB1 and CUL4 associated factor 10	DCAF10	2.67	0.0000
225178_at	tetratricopeptide repeat domain 14	TTC14	2.04	0.0000
227259_at	CD47 molecule	CD47	2.13	0.0000
205536_at	vav 2 guanine nucleotide exchange factor	VAV2	2.96	0.0000
218674_at	chromosome 5 open reading frame 44	C5orf44	2.99	0.0000
226592_at	zinc finger protein 618	ZNF618	11.29	0.0000
210971_s_at	aryl hydrocarbon receptor nuclear translocator-like	ARNTL	2.58	0.0000

Probeset ID	Gene title	Gene symbol	Fold change	FDR (%)
225886_at	DEAD (Asp-Glu-Ala-Asp) box helicase 5	DDX5	2.03	0.0000
49111_at	arrestin, beta 1	ARRB1	2.82	0.0000
201977_s_at	KIAA0141	KIAA0141	2.23	0.0000
214586_at	G protein-coupled receptor 37 (endothelin receptor type B-like)	GPR37	2.45	0.0000
234987_at	SAM domain and HD domain 1	SAMHD1	3.96	0.0000
212636_at	QKI, KH domain containing, RNA binding	QKI	2.70	0.0000
204466_s_at	synuclein, alpha (non A4 component of amyloid precursor)	SNCA	3.67	0.0000
230076_at	PITPNM family member 3	PITPNM3	3.39	0.0000
212161_at	adaptor-related protein complex 2, alpha 2 subunit	AP2A2	2.74	0.0000
222126_at	ArfGAP with FG repeats 2	AGFG2	2.25	0.0000
222917_s_at	T-box 3	TBX3	3.62	0.0000
212362_at	ATPase, Ca++ transporting, cardiac muscle, slow twitch 2	ATP2A2	2.02	0.0000
219848_s_at	zinc finger protein 432	ZNF432	3.21	0.0000
214667_s_at	tumor protein p53 inducible protein 11	TP53I11	3.49	0.0000
239741_at	pygopus homolog 1 (Drosophila)	PYGO1	2.07	0.0000
203275_at	interferon regulatory factor 2	IRF2	2.09	0.0000
229802_at	WNT1 inducible signaling pathway protein 1	WISP1	4.59	0.0000
205670_at	galactose-3-O-sulfotransferase 1	GAL3ST1	3.54	0.0000
243690_at	TRIO and F-actin binding protein	TRIOBP	2.41	0.0000
207592_s_at	hyperpolarization activated cyclic nucleotide-gated potassium channel 2	HCN2	2.55	0.0000
202660_at	inositol 1,4,5-trisphosphate receptor, type 2	ITPR2	3.26	0.0000
225166_at	Rho GTPase activating protein 18	ARHGAP18	2.68	0.0000
223190_s_at	myeloid/lymphoid or mixed-lineage leukemia 5 (trithorax homolog, Drosophila)	MLL5	2.27	0.0000
204112_s_at	histamine N-methyltransferase	HNMT	2.72	0.0000
214924_s_at	trafficking protein, kinesin binding 1	TRAK1	2.04	0.0000
215493_x_at	butyrophilin, subfamily 2, member A1	BTN2A1	2.20	0.0000
204085_s_at	ceroid-lipofuscinosis, neuronal 5	CLN5	2.40	0.0000
226029_at	vang-like 2 (van gogh, Drosophila)	VANGL2	2.60	0.0000
221747_at	tensin 1	TNS1	2.78	0.0000
222482_at	single stranded DNA binding protein 3	SSBP3	2.28	0.0000
210276_s_at	TRIO and F-actin binding protein	TRIOBP	2.54	0.0000
210346_s_at	CDC-like kinase 4	CLK4	2.02	0.0000
227045_at	zinc finger protein 614	ZNF614	3.18	0.0000
223517_at	F-box protein 44	FBXO44	2.07	0.0000
230152_at	WD repeat domain 52	WDR52	2.43	0.0000
229969_at	SEC63 homolog (S. cerevisiae)	SEC63	2.46	0.0000
211421_s_at	ret proto-oncogene	RET	7.16	0.0000
222817_at	hydroxy-delta-5-steroid dehydrogenase, 3 beta- and steroid delta-isomerase 7	HSD3B7	2.15	0.0000
1559094_at	F-box protein 9	FBXO9	3.86	0.0000
212450_at	SECIS binding protein 2-like	SECISBP2L	2.28	0.0000
218540_at	thiamine triphosphatase	THTPA	2.31	0.0000
203148_s_at	tripartite motif containing 14	TRIM14	5.52	0.0000
208957_at	endoplasmic reticulum protein 44	ERP44	3.13	0.0000
59644_at	BMP2 inducible kinase	BMP2K	2.24	0.0000
235486_at	chromosome 11 open reading frame 41	C11orf41	2.42	0.0000
211889_x_at	carcinoembryonic antigen-related cell adhesion molecule 1 (biliary glycoprotein)	CEACAM1	2.53	0.0000
225355_at	neuralized homolog 1B (Drosophila)	NEURL1B	2.56	0.0000
225838_at	enhancer of polycomb homolog 2 (Drosophila)	EPC2	2.44	0.0000
213142_x_at	pigeon homolog (Drosophila)	PION	2.76	0.0000
221563_at	dual specificity phosphatase 10	DUSP10	2.28	0.0000
204720_s_at	DnaJ (Hsp40) homolog, subfamily C, member 6	DNAJC6	2.16	0.0000
243904_at	syntaxin binding protein 5 (tomosyn)	STXBP5	7.86	0.0000
227131_at	mitogen-activated protein kinase kinase kinase 3	MAP3K3	2.19	0.0000
225350_s_at	zyg-11 homolog B (C. elegans)	ZYG11B	2.07	0.0000
226893_at	v-abl Abelson murine leukemia viral oncogene homolog 2	ABL2	2.40	0.0000

Probeset ID	Gene title	Gene symbol	Fold change	FDR (%)
231815_at	PHD finger protein 12	PHF12	2.17	0.0000
202085_at	tight junction protein 2 (zona occludens 2)	TJP2	2.19	0.0000
238007_at	zinc finger protein 271	ZNF271	2.22	0.0000
202553_s_at	SYF2 homolog, RNA splicing factor (S. cerevisiae)	SYF2	2.40	0.0000
218618_s_at	fibronectin type III domain containing 3B	FNDC3B	2.29	0.0000
205732_s_at	nuclear receptor coactivator 2	NCOA2	2.12	0.0000
238542_at	UL16 binding protein 2	ULBP2	3.23	0.0000
243829_at	v-raf murine sarcoma viral oncogene homolog B1	BRAF	2.91	0.0000
227379_at	membrane bound O-acyltransferase domain containing 1	MBOAT1	2.14	0.0000
222778_s_at	Wolf-Hirschhorn syndrome candidate 1	WHSC1	2.68	0.0000
206247_at	MHC class I polypeptide-related sequence B	MICB	2.16	0.0000
223314_at	tetraspanin 14	TSPAN14	2.02	0.0000
229310_at	kelch-like 29 (Drosophila)	KLHL29	2.20	0.0000
202480_s_at	death effector domain containing	DEDD	2.57	0.0000
235318_at	fibrillin 1	FBN1	6.49	0.0000
230081_at	phosphatidylinositol-specific phospholipase C, X domain containing 3	PLCXD3	4.05	0.0000
1557953_at	zinc finger with KRAB and SCAN domains 1	ZKSCAN1	4.11	0.0000
231877_at	tRNA methyltransferase 10 homolog A (S. cerevisiae)	TRMT10A	2.17	0.0000
212239_at	phosphoinositide-3-kinase, regulatory subunit 1 (alpha)	PIK3R1	2.12	0.0000
213530_at	RAB3 GTPase activating protein subunit 1 (catalytic)	RAB3GAP1	2.16	0.0000
226808_at	zinc finger protein 862	ZNF862	2.87	0.0000
222809_x_at	coiled-coil domain containing 85C	CCDC85C	2.50	0.0000
210738_s_at	solute carrier family 4, sodium bicarbonate cotransporter, member 4	SLC4A4	3.33	0.0000
218847_at	insulin-like growth factor 2 mRNA binding protein 2	IGF2BP2	2.11	0.0000
228555_at	calcium/calmodulin-dependent protein kinase II delta	CAMK2D	2.06	0.0000
1565759_at	ribosomal protein L13	RPL13	2.79	0.0000
227188_at	family with sequence similarity 176, member C	FAM176C	2.57	0.0000
233938_at	chromosome 11 open reading frame 86	C11orf86	2.31	0.0000
1569129_s_at	chromosome 3 open reading frame 38	C3orf38	2.14	0.0000
227087_at	inositol polyphosphate-4-phosphatase, type I, 107kDa	INPP4A	2.02	0.0000
232617_at	cathepsin S	CTSS	2.68	0.0000
214104_at	G protein-coupled receptor 161	GPR161	2.49	0.0000
207839_s_at	transmembrane protein 8B	TMEM8B	2.06	0.0000
212932_at	RAB3 GTPase activating protein subunit 1 (catalytic)	RAB3GAP1	2.20	0.0000
206243_at	TIMP metallopeptidase inhibitor 4	TIMP4	2.36	0.0000
230831_at	FERM domain containing 5	FRMD5	2.00	0.0000
226384_at	phosphatidic acid phosphatase type 2 domain containing 1B	PPAPDC1B	2.04	0.0000
244650_at	family with sequence similarity 105, member A	FAM105A	2.75	0.0000
227918_s_at	zyg-11 homolog B (C. elegans)	ZYG11B	2.18	0.0000
204989_s_at	integrin, beta 4	ITGB4	3.58	0.0000
225976_at	basic transcription factor 3-like 4	BTF3L4	2.05	0.0000
1559096_x_at	F-box protein 9	FBXO9	3.95	0.0000
204596_s_at	stanniocalcin 1	STC1	2.56	0.0000
235195_at	F-box and WD repeat domain containing 2	FBXW2	2.74	0.0000
230766_at	phosphoribosylglycinamide formyltransferase, phosphoribosylglycinamide synthetase, phosphoribosylaminoimidazole synthetase	GART	3.20	0.0000
236016_at	SWI/SNF related, matrix associated, actin dependent regulator of chromatin, subfamily e, member 1	SMARCE1	3.49	0.0000
221701_s_at	stimulated by retinoic acid gene 6 homolog (mouse)	STRA6	2.85	0.0000
219694_at	family with sequence similarity 105, member A	FAM105A	2.66	0.0000
212565_at	serine/threonine kinase 38 like	STK38L	2.02	0.0000
230063_at	zinc finger protein 264	ZNF264	3.01	0.0000
217297_s_at	myosin IXB	MYO9B	2.15	0.0000
204907_s_at	B-cell CLL/lymphoma 3	BCL3	2.45	0.0000
223276_at	chromosome 5 open reading frame 62	C5orf62	2.04	0.0000
230300_at	proteasome (prosome, macropain) subunit, alpha type, 5	PSMA5	6.08	0.0000

Probeset ID	Gene title	Gene symbol	Fold change	FDR (%)
226747_at	thioredoxin domain containing 16	TXNDC16	2.07	0.0000
217920_at	mannosidase, alpha, class 1A, member 2	MAN1A2	2.03	0.0000
227079_at	DEAH (Asp-Glu-Ala-His) box polypeptide 8	DHX8	2.09	0.0000
219683_at	frizzled family receptor 3	FZD3	3.11	0.0000
220961_s_at	transforming growth factor beta regulator 4	TBRG4	2.88	0.0000
224492_s_at	zinc finger protein 627	ZNF627	2.32	0.0000
228603_at	ARP3 actin-related protein 3 homolog (yeast)	ACTR3	2.27	0.0000
219768_at	V-set domain containing T cell activation inhibitor 1	VTCN1	2.10	0.0000
227261_at	Kruppel-like factor 12	KLF12	2.25	0.0000
210653_s_at	branched chain keto acid dehydrogenase E1, beta polypeptide	BCKDHB	2.05	0.0000
242943_at	ST8 alpha-N-acetyl-neuraminide alpha-2,8-sialyltransferase 4	ST8SIA4	3.24	0.0000
204343_at	ATP-binding cassette, sub-family A (ABC1), member 3	ABCA3	3.42	0.0000
204131_s_at	forkhead box O3	FOXO3	2.05	0.0000
222820_at	trinucleotide repeat containing 6C	TNRC6C	2.07	0.0000
214492_at	sarcoglycan, delta (35kDa dystrophin-associated glycoprotein)	SGCD	2.70	0.0000
219155_at	phosphatidylinositol transfer protein, cytoplasmic 1	PITPNC1	2.05	0.0000
207298_at	solute carrier family 17 (sodium phosphate), member 3	SLC17A3	5.06	0.0000
226649_at	pantothenate kinase 1	PANK1	3.36	0.0000
209211_at	Kruppel-like factor 5 (intestinal)	KLF5	2.05	0.0000
224817_at	SH3 and PX domains 2A	SH3PXD2A	4.01	0.0000
205442_at	microfibrillar-associated protein 3-like	MFAP3L	5.15	0.0000
228991_at	cyclin-dependent kinase 13	CDK13	2.06	0.0000
231873_at	bone morphogenetic protein receptor, type II (serine/threonine kinase)	BMPR2	2.17	0.0000
202718_at	insulin-like growth factor binding protein 2, 36kDa	IGFBP2	2.07	0.0000
213040_s_at	neuronal pentraxin receptor	NPTXR	5.40	0.0000
219583_s_at	spermatogenesis associated 7	SPATA7	2.05	0.0000
218147_s_at	glycosyltransferase 8 domain containing 1	GLT8D1	2.01	0.0000
228200_at	zinc finger protein 252, pseudogene	ZNF252P	4.00	0.0000
228400_at	shroom family member 3	SHROOM3	2.09	0.0000
227717_at	Rho guanine nucleotide exchange factor (GEF) 37	ARHGEF37	2.08	0.0000
219234_x_at	secernin 3	SCRN3	2.26	0.0000
208502_s_at	paired-like homeodomain 1	PITX1	2.06	0.0000
222634_s_at	transducin (beta)-like 1 X-linked receptor 1	TBL1XR1	2.13	0.0000
222491_at	heparan-alpha-glucosaminide N-acetyltransferase	HGSNAT	5.42	0.0000
226615_at	xenotropic and polytropic retrovirus receptor 1	XPR1	2.93	0.0000
229866_at	serine/threonine kinase 32A	STK32A	3.13	0.0000
200856_x_at	nuclear receptor corepressor 1	NCOR1	2.16	0.0000
231990_at	ubiquitin specific peptidase 15	USP15	2.44	0.0000
244111_at	keratin 222	KRT222	5.58	0.0000
214647_s_at	hemochromatosis	HFE	2.50	0.0000
225956_at	CREB3 regulatory factor	CREBRF	4.40	0.0000
209846_s_at	butyrophilin, subfamily 3, member A2	BTN3A2	2.61	0.0000
228813_at	histone deacetylase 4	HDAC4	3.45	0.0000
230141_at	AT rich interactive domain 4A (RBP1-like)	ARID4A	2.16	0.0000
1553117_a_at	serine/threonine kinase 38	STK38	2.00	0.0000
241950_at	WW and C2 domain containing 1	WWC1	2.18	0.0000
205076_s_at	myotubularin related protein 11	MTMR11	2.50	0.0000
208935_s_at	lectin, galactoside-binding, soluble, 8	LGALS8	2.05	0.0000
222111_at	family with sequence similarity 63, member B	FAM63B	2.23	0.0000
200962_at	ribosomal protein L31	RPL31	2.32	0.0000
211330_s_at	hemochromatosis	HFE	6.04	0.0000
207717_s_at	plakophilin 2	PKP2	3.04	0.0000
227979_at	RNA binding motif protein 4	RBM4	2.17	0.0000
228709_at	translocated promoter region, nuclear basket protein	TPR	4.33	0.0000
231944_at	ERO1-like beta (S. cerevisiae)	ERO1LB	2.32	0.0000

Probeset ID	Gene title	Gene symbol	Fold change	FDR (%)
228221_at	solute carrier family 44, member 3	SLC44A3	2.49	0.0000
232865_at	AF4/FMR2 family, member 4	AFF4	2.29	0.0000
201860_s_at	plasminogen activator, tissue	PLAT	3.45	0.0000
204949_at	intercellular adhesion molecule 3	ICAM3	2.92	0.0000
1558700_s_at	zinc finger protein 260	ZNF260	2.04	0.0000
211906_s_at	serpin peptidase inhibitor, clade B (ovalbumin), member 4	SERPINB4	5.45	0.0000
207052_at	hepatitis A virus cellular receptor 1	HAVCR1	3.41	0.0000
205092_x_at	zinc finger and BTB domain containing 1	ZBTB1	3.70	0.0000
207425_s_at	septin 9	SEPT9	2.43	0.0000
238948_at	Transmembrane 9 superfamily member 1	TM9SF1	2.17	0.0000
212026_s_at	exocyst complex component 7	EXOC7	2.08	0.0000
222719_s_at	platelet derived growth factor C	PDGFC	2.75	0.0000
238860_at	chromosome 6 open reading frame 130	C6orf130	2.38	0.0000
206576_s_at	carcinoembryonic antigen-related cell adhesion molecule 1 (biliary glycoprotein)	CEACAM1	3.08	0.0000
225817_at	cingulin-like 1	CGNL1	3.05	0.0000
1554522_at	cyclin M2	CNNM2	2.63	0.0000
229667_s_at	homeobox B8	HOXB8	3.67	0.0000
203521_s_at	zinc finger protein 318	ZNF318	2.31	0.0000
239272_at	matrix metallopeptidase 28	MMP28	5.35	0.0000
223458_at	seizure related 6 homolog (mouse)-like 2	SEZ6L2	2.04	0.0000
1555724_s_at	transgelin	TAGLN	2.03	0.0000
226657_at	chromosome 17 open reading frame 103	C17orf103	2.89	0.0000
220975_s_at	C1q and tumor necrosis factor related protein 1	C1QTNF1	2.85	0.0000
201367_s_at	zinc finger protein 36, C3H type-like 2	ZFP36L2	2.10	0.0000
202995_s_at	fibulin 1	FBLN1	4.97	0.0000
1562321_at	pyruvate dehydrogenase kinase, isozyme 4	PDK4	3.25	0.0000
208733_at	RAB2A, member RAS oncogene family	RAB2A	2.48	0.0000
222807_at	chromosome 11 open reading frame 30	C11orf30	2.74	0.0000
218980_at	formin homology 2 domain containing 3	FHOD3	2.46	0.0000
228305_at	zinc finger protein 565	ZNF565	2.86	0.0000
228304_at	RNA binding motif protein 43	RBM43	2.65	0.0000
220397_at	Mdm1 nuclear protein homolog (mouse)	MDM1	2.71	0.0000
226770_at	membrane associated guanylate kinase, WW and PDZ domain containing 3	MAGI3	2.36	0.0000
224976_at	nuclear factor I/A	NFIA	2.52	0.0000
207392_x_at	UDP glucuronosyltransferase 2 family, polypeptide B15	UGT2B15	3.45	0.0000
221909_at	ring finger protein, transmembrane 2	RNFT2	2.06	0.0000
1552502_s_at	rhomboid, veinlet-like 2 (Drosophila)	RHBDL2	2.01	0.0000
211478_s_at	dipeptidyl-peptidase 4	DPP4	4.56	0.0000
207437_at	neuro-oncological ventral antigen 1	NOVA1	5.11	0.0000
238430_x_at	schlafen family member 5	SLFN5	2.09	0.0000
221276_s_at	syncoilin, intermediate filament protein	SYNC	2.48	0.0000
206053_at	zinc finger protein 510	ZNF510	2.19	0.0000
218273_s_at	pyruvate dehyrogenase phosphatase catalytic subunit 1	PDP1	2.14	0.0000
213480_at	vesicle-associated membrane protein 4	VAMP4	2.07	0.0000
226745_at	cytochrome P450, family 4, subfamily V, polypeptide 2	CYP4V2	4.21	0.0000
211009_s_at	zinc finger protein 271	ZNF271	4.24	0.0000
235019_at	carboxypeptidase M	CPM	2.33	0.0000
242705_x_at	low density lipoprotein receptor-related protein associated protein 1	LRPAP1	2.16	0.0000
230559_x_at	FYVE, RhoGEF and PH domain containing 4	FGD4	3.44	0.0000
210961_s_at	adrenoceptor alpha 1D	ADRA1D	2.16	0.0000
212599_at	autism susceptibility candidate 2	AUTS2	2.58	0.0000
217371_s_at	interleukin 15	IL15	2.16	0.0000
214953_s_at	amyloid beta (A4) precursor protein	APP	2.05	0.0000
224943_at	BTB (POZ) domain containing 7	BTBD7	2.09	0.0000
1569788_at	ST8 alpha-N-acetyl-neuraminide alpha-2,8-sialyltransferase 1	ST8SIA1	4.07	0.0000

Probeset ID	Gene title	Gene symbol	Fold change	FDR (%)
213272_s_at	transmembrane protein 159	TMEM159	2.39	0.0000
235308_at	zinc finger and BTB domain containing 20	ZBTB20	2.21	0.0000
219077_s_at	WW domain containing oxidoreductase	WWOX	2.23	0.0000
215543_s_at	like-glycosyltransferase	LARGE	2.35	0.0000
244614_at	TRK-fused gene	TFG	2.30	0.0000
228145_s_at	zinc finger protein 398	ZNF398	2.12	0.0063
208832_at	ataxin 10	ATXN10	2.13	0.0063
1555829_at	extended synaptotagmin-like protein 2	ESYT2	2.27	0.0063
41660_at	cadherin, EGF LAG seven-pass G-type receptor 1 (flamingo homolog, Drosophila)	CELSR1	2.11	0.0063
228751_at	CDC-like kinase 4	CLK4	2.09	0.0063
1570130_at	spermatogenesis associated, serine-rich 2	SPATS2	3.05	0.0063
209355_s_at	phosphatidic acid phosphatase type 2B	PPAP2B	2.08	0.0063
235052_at	zinc finger protein 792	ZNF792	2.37	0.0063
231577_s_at	guanylate binding protein 1, interferon-inducible	GBP1	3.05	0.0063
214329_x_at	tumor necrosis factor (ligand) superfamily, member 10	TNFSF10	8.56	0.0063
1555905_a_at	chromosome 3 open reading frame 23	C3orf23	2.15	0.0063
1558662_s_at	B-cell scaffold protein with ankyrin repeats 1	BANK1	2.11	0.0063
200855_at	nuclear receptor corepressor 1	NCOR1	2.21	0.0063
225276_at	G1 to S phase transition 1	GSPT1	2.01	0.0063
204798_at	v-myb myeloblastosis viral oncogene homolog (avian)	MYB	2.50	0.0063
213721_at	SRY (sex determining region Y)-box 2	SOX2	2.35	0.0063
205107_s_at	ephrin-A4	EFNA4	2.34	0.0063
1553402_a_at	hemochromatosis	HFE	3.13	0.0063
212439_at	inositol hexakisphosphate kinase 1	IP6K1	2.30	0.0063
227937_at	Myb-related transcription factor, partner of profilin	MYPOP	2.53	0.0063
241031_at	C2 calcium-dependent domain containing 4A	C2CD4A	3.36	0.0063
237206_at	myocardin	MYOCD	2.97	0.0063
228241_at	anterior gradient 3 homolog (Xenopus laevis)	AGR3	4.30	0.0063
207565_s_at	major histocompatibility complex, class I-related	MR1	2.01	0.0063
221085_at	tumor necrosis factor (ligand) superfamily, member 15	TNFSF15	2.04	0.0063
227499_at	frizzled family receptor 3	FZD3	2.68	0.0063
1554411_at	catenin (cadherin-associated protein), beta 1, 88kDa	CTNNB1	2.88	0.0063
205674_x_at	FXYD domain containing ion transport regulator 2	FXYD2	2.15	0.0063
207996_s_at	chromosome 18 open reading frame 1	C18orf1	2.95	0.0063
228316_at	chromosome 2 open reading frame 63	C2orf63	2.85	0.0063
1557302_at	zinc finger protein 585B	ZNF585B	4.81	0.0063
219048_at	phosphatidylinositol glycan anchor biosynthesis, class N	PIGN	2.21	0.0063
238647_at	chromosome 14 open reading frame 28	C14orf28	2.23	0.0063
235529_x_at	SAM domain and HD domain 1	SAMHD1	2.31	0.0063
223444_at	SUMO1/sentrin specific peptidase 7	SENP7	2.01	0.0063
205759_s_at	sulfotransferase family, cytosolic, 2B, member 1	SULT2B1	2.12	0.0063
1556389_at	canopy 3 homolog (zebrafish)	CNPY3	3.08	0.0063
228115_at	family with sequence similarity 59, member A	FAM59A	2.19	0.0063
241364_at	transmembrane protein 57	TMEM57	2.06	0.0063
1553589_a_at	PDZK1 interacting protein 1	PDZK1IP1	2.70	0.0063
202073_at	optineurin	OPTN	2.24	0.0063
223627_at	mex-3 homolog B (C. elegans)	MEX3B	2.18	0.0063
226641_at	ankyrin repeat domain 44	ANKRD44	3.68	0.0063
227461_at	stonin 2	STON2	2.32	0.0063
229199_at	sodium channel, voltage-gated, type IX, alpha subunit	SCN9A	2.66	0.0063
239492_at	SEC14-like 4 (S. cerevisiae)	SEC14L4	2.02	0.0063
226873_at	family with sequence similarity 63, member B	FAM63B	2.13	0.0063
206950_at	sodium channel, voltage-gated, type IX, alpha subunit	SCN9A	2.10	0.0063
223557_s_at	transmembrane protein with EGF-like and two follistatin-like domains 2	TMEFF2	5.02	0.0063
212385_at	transcription factor 4	TCF4	2.25	0.0063

Probeset ID	Gene title	Gene symbol	Fold change	FDR (%)
207558_s_at	paired-like homeodomain 2	PITX2	2.12	0.0063
212978_at	leucine rich repeat containing 8 family, member B	LRRC8B	2.07	0.0063
206448_at	zinc finger protein 365	ZNF365	2.77	0.0063
40016_g_at	microtubule associated serine/threonine kinase family member 4	MAST4	2.26	0.0063
225619_at	SLAIN motif family, member 1	SLAIN1	2.56	0.0063
219630_at	PDZK1 interacting protein 1	PDZK1IP1	2.06	0.0063
203339_at	solute carrier family 25 (aspartate/glutamate carrier), member 12	SLC25A12	3.12	0.0063
221788_at	phosphoglucomutase 3	PGM3	2.20	0.0063
232271_at	hepatocyte nuclear factor 4, gamma	HNF4G	2.90	0.0063
243880_at	golgi SNAP receptor complex member 2	GOSR2	2.55	0.0063
203717_at	dipeptidyl-peptidase 4	DPP4	2.37	0.0063
229377_at	growth hormone regulated TBC protein 1	GRTP1	2.36	0.0063
203543_s_at	Kruppel-like factor 9	KLF9	5.80	0.0063
202662_s_at	inositol 1,4,5-trisphosphate receptor, type 2	ITPR2	2.19	0.0063
1552365_at	scinderin	SCIN	2.21	0.0063
215617_at	spermatogenesis associated, serine-rich 2-like	SPATS2L	2.87	0.0063
238571_at	homeobox A13	HOXA13	2.50	0.0063
1558697_a_at	KIAA0430	KIAA0430	2.51	0.0063
213436_at	cannabinoid receptor 1 (brain)	CNR1	2.06	0.0063
227250_at	kringle containing transmembrane protein 1	KREMEN1	2.66	0.0063
201647_s_at	scavenger receptor class B, member 2	SCARB2	2.01	0.0063
203683_s_at	vascular endothelial growth factor B	VEGFB	2.35	0.0063
213245_at	adenylate cyclase 1 (brain)	ADCY1	3.36	0.0164
219973_at	arylsulfatase family, member J	ARSJ	2.04	0.0164
244354_at	striatin, calmodulin binding protein	STRN	2.04	0.0164
213693_s_at	mucin 1, cell surface associated	MUC1	2.09	0.0164
215465_at	ATP-binding cassette, sub-family A (ABC1), member 12	ABCA12	2.57	0.0164
204897_at	prostaglandin E receptor 4 (subtype EP4)	PTGER4	2.15	0.0164
222696_at	axin 2	AXIN2	2.49	0.0164
209006_s_at	chromosome 1 open reading frame 63	C1orf63	3.23	0.0164
209701_at	endoplasmic reticulum aminopeptidase 1	ERAP1	2.30	0.0164
205466_s_at	heparan sulfate (glucosamine) 3-O-sulfotransferase 1	HS3ST1	2.20	0.0164
222838_at	SLAM family member 7	SLAMF7	6.17	0.0164
235279_at	parvin, alpha	PARVA	2.23	0.0164
236335_at	guanylate cyclase 1, soluble, alpha 2	GUCY1A2	2.56	0.0164
229374_at	EPH receptor A4	EPHA4	2.54	0.0164
236561_at	transforming growth factor, beta receptor 1	TGFBR1	2.11	0.0164
228113_at	RAB37, member RAS oncogene family	RAB37	2.76	0.0164
204320_at	collagen, type XI, alpha 1	COL11A1	2.29	0.0480
1558722_at	zinc finger protein 252, pseudogene	ZNF252P	2.74	0.0480
214464_at	CDC42 binding protein kinase alpha (DMPK-like)	CDC42BPA	2.11	0.0480
1559496_at	pyrophosphatase (inorganic) 2	PPA2	2.44	0.0480
220087_at	beta-carotene 15,15'-monooxygenase 1	BCMO1	2.44	0.0480
212942_s_at	KIAA1199	KIAA1199	2.25	0.0480
223799_at	Myb/SANT-like DNA-binding domain containing 4 with coiled-coils	MSANTD4	2.26	0.0480
235556_at	CREB3 regulatory factor	CREBRF	2.78	0.0480
228185_at	zinc finger protein 25	ZNF25	2.87	0.1147
215671_at	phosphodiesterase 4B, cAMP-specific	PDE4B	2.05	0.1147
214652_at	dopamine receptor D1	DRD1	2.23	0.1147
232304_at	Pellino E3 ubiquitin protein ligase 1	PELI1	6.62	0.1147
211776_s_at	erythrocyte membrane protein band 4.1-like 3	EPB41L3	2.24	0.1147
231029_at	coagulation factor V (proaccelerin, labile factor)	F5	2.14	0.1147
230147_at	coagulation factor II (thrombin) receptor-like 2	F2RL2	0.07	0.0000
225540_at	microtubule-associated protein 2	MAP2	0.02	0.0000
209337_at	PC4 and SFRS1 interacting protein 1	PSIP1	0.11	0.0000

Probeset ID	Gene title	Gene symbol	Fold change	FDR (%)
228824_s_at	prostaglandin reductase 1	PTGR1	0.12	0.0000
218468_s_at	gremlin 1	GREM1	0.04	0.0000
207528_s_at	solute carrier family 7 (anionic amino acid transporter light chain, xc- system), member 11	SLC7A11	0.02	0.0000
213112_s_at	sequestosome 1	SQSTM1	0.06	0.0000
203925_at	glutamate-cysteine ligase, modifier subunit	GCLM	0.15	0.0000
202628_s_at	serpin peptidase inhibitor, clade E (nexin, plasminogen activator inhibitor type 1), member 1	SERPINE1	0.12	0.0000
231963_at	ankyrin repeat domain 33B	ANKRD33B	0.12	0.0000
205681_at	BCL2-related protein A1	BCL2A1	0.05	0.0000
1555167_s_at	nicotinamide phosphoribosyltransferase	NAMPT	0.16	0.0000
209921_at	solute carrier family 7 (anionic amino acid transporter light chain, xc- system), member 11	SLC7A11	0.17	0.0000
228825_at	prostaglandin reductase 1	PTGR1	0.03	0.0000
218469_at	gremlin 1	GREM1	0.06	0.0000
202627_s_at	serpin peptidase inhibitor, clade E (nexin, plasminogen activator inhibitor type 1), member 1	SERPINE1	0.12	0.0000
203700_s_at	deiodinase, iodothyronine, type II	DIO2	0.21	0.0000
219789_at	natriuretic peptide receptor C/guanylate cyclase C (atrionatriuretic peptide receptor C)	NPR3	0.16	0.0000
217739_s_at	nicotinamide phosphoribosyltransferase	NAMPT	0.22	0.0000
223484_at	chromosome 15 open reading frame 48	C15orf48	0.09	0.0000
243296_at	Nicotinamide phosphoribosyltransferase	NAMPT	0.04	0.0000
231897_at	prostaglandin reductase 1	PTGR1	0.12	0.0000
212977_at	chemokine (C-X-C motif) receptor 7	CXCR7	0.14	0.0000
225252_at	sulfiredoxin 1	SRXN1	0.18	0.0000
203665_at	heme oxygenase (decycling) 1	HMOX1	0.09	0.0000
203002_at	angiomotin like 2	AMOTL2	0.23	0.0000
205192_at	mitogen-activated protein kinase kinase kinase 14	MAP3K14	0.22	0.0000
1558322_a_at	progestin and adipoQ receptor family member IX	PAQR9	0.20	0.0000
217626_at	aldo-keto reductase family 1, member C1 (dihydrodiol dehydrogenase 1; 20-alpha (3-alpha)-hydroxysteroid dehydrogenase)	AKR1C1	0.11	0.0000
217738_at	nicotinamide phosphoribosyltransferase	NAMPT	0.24	0.0000
227614_at	hexokinase domain containing 1	HKDC1	0.09	0.0000
229778_at	chromosome 12 open reading frame 39	C12orf39	0.33	0.0000
237324_s_at	hexokinase domain containing 1	HKDC1	0.16	0.0000
242037_at	aspartate beta-hydroxylase	ASPH	0.17	0.0000
205920_at	solute carrier family 6 (neurotransmitter transporter, taurine), member 6	SLC6A6	0.25	0.0000
210663_s_at	kynureninase	KYNU	0.33	0.0000
1552648_a_at	tumor necrosis factor receptor superfamily, member 10a	TNFRSF10A	0.30	0.0000
218261_at	adaptor-related protein complex 1, mu 2 subunit	AP1M2	0.24	0.0000
33323_r_at	stratifin	SFN	0.22	0.0000
206153_at	cytochrome P450, family 4, subfamily F, polypeptide 11	CYP4F11	0.01	0.0000
220407_s_at	transforming growth factor, beta 2	TGFB2	0.21	0.0000
201700_at	cyclin D3	CCND3	0.32	0.0000
204341_at	tripartite motif containing 16	TRIM16	0.14	0.0000
204105_s_at	neuronal cell adhesion molecule	NRCAM	0.31	0.0000
204420_at	FOS-like antigen 1	FOSL1	0.16	0.0000
33322_i_at	stratifin	SFN	0.28	0.0000
201146_at	nuclear factor (erythroid-derived 2)-like 2	NFE2L2	0.22	0.0000
218726_at	Holliday junction recognition protein	HJURP	0.33	0.0000
204059_s_at	malic enzyme 1, NADP(+)-dependent, cytosolic	ME1	0.23	0.0000
206118_at	signal transducer and activator of transcription 4	STAT4	0.18	0.0000
209909_s_at	transforming growth factor, beta 2	TGFB2	0.24	0.0000
219952_s_at	mucolipin 1	MCOLN1	0.28	0.0000
201490_s_at	peptidylprolyl isomerase F	PPIF	0.26	0.0000
227342_s_at	myeloma overexpressed (in a subset of t(11;14) positive multiple myelomas)	MYEOV	0.25	0.0000

Probeset ID	Gene title	Gene symbol	Fold change	FDR (%)
239595_at	glutathione peroxidase 2 (gastrointestinal)	GPX2	0.20	0.0000
209260_at	stratifin	SFN	0.19	0.0000
202923_s_at	glutamate-cysteine ligase, catalytic subunit	GCLC	0.15	0.0000
209735_at	ATP-binding cassette, sub-family G (WHITE), member 2	ABCG2	0.24	0.0000
206155_at	ATP-binding cassette, sub-family C (CFTR/MRP), member 2	ABCC2	0.29	0.0000
231094_s_at	methylenetetrahydrofolate dehydrogenase (NADP+ dependent) 1-like	MTHFD1L	0.32	0.0000
209928_s_at	musculin	MSC	0.33	0.0000
220333_at	progestin and adipoQ receptor family member V	PAQR5	0.42	0.0000
212767_at	mitochondrial GTPase 1 homolog (S. cerevisiae)	MTG1	0.33	0.0000
206368_at	complexin 2	CPLX2	0.26	0.0000
206825_at	oxytocin receptor	OXTR	0.32	0.0000
228205_at	transketolase	TKT	0.03	0.0000
201471_s_at	sequestosome 1	SQSTM1	0.28	0.0000
237215_s_at	transferrin receptor (p90, CD71)	TFRC	0.24	0.0000
201348_at	glutathione peroxidase 3 (plasma)	GPX3	0.02	0.0000
206653_at	polymerase (RNA) III (DNA directed) polypeptide G (32kD)	POLR3G	0.21	0.0000
232021_at	glucoside xylosyltransferase 1	GXYLT1	0.06	0.0000
202347_s_at	ubiquitin-conjugating enzyme E2K	UBE2K	0.39	0.0000
209969_s_at	signal transducer and activator of transcription 1, 91kDa	STAT1	0.26	0.0000
203427_at	ASF1 anti-silencing function 1 homolog A (S. cerevisiae)	ASF1A	0.24	0.0000
236140_at	glutamate-cysteine ligase, modifier subunit	GCLM	0.10	0.0000
209875_s_at	secreted phosphoprotein 1	SPP1	0.28	0.0000
207180_s_at	HIV-1 Tat interactive protein 2, 30kDa	HTATIP2	0.40	0.0000
202053_s_at	aldehyde dehydrogenase 3 family, member A2	ALDH3A2	0.40	0.0000
220234_at	carbonic anhydrase VIII	CA8	0.03	0.0000
235177_at	methyltransferase like 21A	METTL21A	0.27	0.0000
203163_at	katanin p80 (WD repeat containing) subunit B 1	KATNB1	0.48	0.0000
201489_at	peptidylprolyl isomerase F	PPIF	0.30	0.0000
203701_s_at	tRNA methyltransferase 1 homolog (S. cerevisiae)	TRMT1	0.32	0.0000
206654_s_at	polymerase (RNA) III (DNA directed) polypeptide G (32kD)	POLR3G	0.19	0.0000
234986_at	glutamate-cysteine ligase, modifier subunit	GCLM	0.10	0.0000
201463_s_at	transaldolase 1	TALDO1	0.29	0.0000
221541_at	cysteine-rich secretory protein LCCL domain containing 2	CRISPLD2	0.08	0.0000
240042_at	fibrinogen C domain containing 1	FIBCD1	0.35	0.0000
223666_at	sorting nexin 5	SNX5	0.16	0.0000
203234_at	uridine phosphorylase 1	UPP1	0.21	0.0000
204465_s_at	internexin neuronal intermediate filament protein, alpha	INA	0.24	0.0000
209213_at	carbonyl reductase 1	CBR1	0.38	0.0000
202922_at	glutamate-cysteine ligase, catalytic subunit	GCLC	0.16	0.0000
227697_at	suppressor of cytokine signaling 3	SOCS3	0.21	0.0000
217678_at	solute carrier family 7 (anionic amino acid transporter light chain, xc- system), member 11	SLC7A11	0.17	0.0000
203699_s_at	deiodinase, iodothyronine, type II	DIO2	0.06	0.0000
202266_at	tyrosyl-DNA phosphodiesterase 2	TDP2	0.40	0.0000
227280_s_at	cyclin Y-like 1	CCNYL1	0.37	0.0000
205623_at	aldehyde dehydrogenase 3 family, member A1	ALDH3A1	0.10	0.0000
202912_at	adrenomedullin	ADM	0.38	0.0000
227771_at	leukemia inhibitory factor receptor alpha	LIFR	0.34	0.0000
228186_s_at	R-spondin 3	RSPO3	0.18	0.0000
244519_at	additional sex combs like 1 (Drosophila)	ASXL1	0.37	0.0000
222703_s_at	yrdC domain containing (E. coli)	YRDC	0.23	0.0000
209908_s_at	transforming growth factor, beta 2	TGFB2	0.09	0.0000
227167_s_at	Ras association (RalGDS/AF-6) domain family member 3	RASSF3	0.32	0.0000
1562484_at	chromosome 17 open reading frame 104	C17orf104	0.25	0.0000
214096_s_at	serine hydroxymethyltransferase 2 (mitochondrial)	SHMT2	0.38	0.0000
224739_at	pim-3 oncogene	PIM3	0.34	0.0000

Probeset ID	Gene title	Gene symbol	Fold change	FDR (%)
209636_at	nuclear factor of kappa light polypeptide gene enhancer in B-cells 2 (p49/p100)	NFKB2	0.18	0.0000
211150_s_at	dihydrolipoamide S-acetyltransferase	DLAT	0.42	0.0000
205895_s_at	nucleolar and coiled-body phosphoprotein 1	NOLC1	0.39	0.0000
203612_at	bystin-like	BYSL	0.30	0.0000
209348_s_at	v-maf musculoaponeurotic fibrosarcoma oncogene homolog (avian)	MAF	0.14	0.0000
201266_at	thioredoxin reductase 1	TXNRD1	0.26	0.0000
207332_s_at	transferrin receptor (p90, CD71)	TFRC	0.35	0.0000
236471_at	nuclear factor (erythroid-derived 2)-like 3	NFE2L3	0.08	0.0000
211949_s_at	nucleolar and coiled-body phosphoprotein 1	NOLC1	0.34	0.0000
219502_at	nei endonuclease VIII-like 3 (E. coli)	NEIL3	0.16	0.0000
201858_s_at	serglycin	SRGN	0.21	0.0000
212872_s_at	mediator complex subunit 20	MED20	0.43	0.0000
221064_s_at	unkempt homolog (Drosophila)-like	UNKL	0.17	0.0000
205633_s_at	aminolevulinate, delta-, synthase 1	ALAS1	0.44	0.0000
203428_s_at	ASF1 anti-silencing function 1 homolog A (S. cerevisiae)	ASF1A	0.21	0.0000
219054_at	natriuretic peptide receptor C/guanylate cyclase C (atrionatriuretic peptide receptor C)	NPR3	0.31	0.0000
241371_at	tumor necrosis factor receptor superfamily, member 10a	TNFRSF10A	0.25	0.0000
208456_s_at	related RAS viral (r-ras) oncogene homolog 2	RRAS2	0.45	0.0000
226585_at	nei endonuclease VIII-like 2 (E. coli)	NEIL2	0.48	0.0000
204702_s_at	nuclear factor (erythroid-derived 2)-like 3	NFE2L3	0.12	0.0000
212563_at	block of proliferation 1	BOP1	0.36	0.0000
205961_s_at	PC4 and SFRS1 interacting protein 1	PSIP1	0.12	0.0000
230788_at	glucosaminyl (N-acetyl) transferase 2, I-branching enzyme (I blood group)	GCNT2	0.33	0.0000
224966_s_at	dihydrouridine synthase 3-like (S. cerevisiae)	DUS3L	0.45	0.0000
235020_at	TAF4b RNA polymerase II, TATA box binding protein (TBP)-associated factor, 105kDa	TAF4B	0.28	0.0000
1553764_a_at	ajuba LIM protein	AJUBA	0.41	0.0000
227006_at	protein phosphatase 1, regulatory (inhibitor) subunit 14A	PPP1R14A	0.37	0.0000
216959_x_at	neuronal cell adhesion molecule	NRCAM	0.24	0.0000
226868_at	glucoside xylosyltransferase 1	GXYLT1	0.34	0.0000
208799_at	proteasome (prosome, macropain) subunit, beta type, 5	PSMB5	0.46	0.0000
221841_s_at	Kruppel-like factor 4 (gut)	KLF4	0.43	0.0000
218647_s_at	yrdC domain containing (E. coli)	YRDC	0.33	0.0000
204133_at	ribosomal RNA processing 9, small subunit (SSU) processome component, homolog (yeast)	RRP9	0.46	0.0000
203386_at	TBC1 domain family, member 4	TBC1D4	0.45	0.0000
218105_s_at	mitochondrial ribosomal protein L4	MRPL4	0.48	0.0000
202267_at	laminin, gamma 2	LAMC2	0.35	0.0000
206074_s_at	high mobility group AT-hook 1	HMGA1	0.39	0.0000
225179_at	ubiquitin-conjugating enzyme E2K	UBE2K	0.41	0.0000
220639_at	transmembrane 4 L six family member 20	TM4SF20	0.27	0.0000
208231_at	neuregulin 1	NRG1	0.29	0.0000
218880_at	FOS-like antigen 2	FOSL2	0.43	0.0000
209448_at	HIV-1 Tat interactive protein 2, 30kDa	HTATIP2	0.44	0.0000
201859_at	serglycin	SRGN	0.28	0.0000
209859_at	tripartite motif containing 9	TRIM9	0.39	0.0000
209132_s_at	COMM domain containing 4	COMMD4	0.47	0.0000
225815_at	complexin 2	CPLX2	0.08	0.0000
221027_s_at	phospholipase A2, group XIIA	PLA2G12A	0.41	0.0000
207348_s_at	ligase III, DNA, ATP-dependent	LIG3	0.29	0.0000
202352_s_at	proteasome (prosome, macropain) 26S subunit, non-ATPase, 12	PSMD12	0.45	0.0000
203177_x_at	transcription factor A, mitochondrial	TFAM	0.47	0.0000
209357_at	Cbp/p300-interacting transactivator, with Glu/Asp-rich carboxy-terminal domain, 2	CITED2	0.46	0.0000
204351_at	S100 calcium binding protein P	S100P	0.26	0.0000

Probeset ID	Gene title	Gene symbol	Fold change	FDR (%)
201387_s_at	ubiquitin carboxyl-terminal esterase L1 (ubiquitin thiolesterase)	UCHL1	0.42	0.0000
210138_at	regulator of G-protein signaling 20	RGS20	0.18	0.0000
227896_at	BRCA2 and CDKN1A interacting protein	BCCIP	0.20	0.0000
225866_at	ribosome production factor 2 homolog (S. cerevisiae)	RPF2	0.49	0.0000
202432_at	protein phosphatase 3, catalytic subunit, beta isozyme	PPP3CB	0.39	0.0000
226905_at	family with sequence similarity 101, member B	FAM101B	0.30	0.0000
234915_s_at	density-regulated protein	DENR	0.43	0.0000
208747_s_at	complement component 1, s subcomponent	C1S	0.40	0.0000
201037_at	phosphofructokinase, platelet	PFKP	0.38	0.0000
202831_at	glutathione peroxidase 2 (gastrointestinal)	GPX2	0.29	0.0000
213906_at	v-myb myeloblastosis viral oncogene homolog (avian)-like 1	MYBL1	0.41	0.0000
209949_at	neutrophil cytosolic factor 2	NCF2	0.05	0.0000
225352_at	SEC62 homolog (S. cerevisiae)	SEC62	0.39	0.0000
206027_at	S100 calcium binding protein A3	S100A3	0.21	0.0000
203536_s_at	cytosolic iron-sulfur protein assembly 1	CIAO1	0.47	0.0000
219258_at	TIMELESS interacting protein	TIPIN	0.32	0.0000
244103_at	SDE2 telomere maintenance homolog (S. pombe)	SDE2	0.42	0.0000
208872_s_at	receptor accessory protein 5	REEP5	0.40	0.0000
1555330_at	glutamate-cysteine ligase, catalytic subunit	GCLC	0.34	0.0000
220585_at	hexokinase domain containing 1	HKDC1	0.11	0.0000
217388_s_at	kynureninase	KYNU	0.41	0.0000
218177_at	charged multivesicular body protein 1B	CHMP1B	0.47	0.0000
204385_at	kynureninase	KYNU	0.33	0.0000
225806_at	ajuba LIM protein	AJUBA	0.40	0.0000
203743_s_at	thymine-DNA glycosylase	TDG	0.40	0.0000
227072_at	rotatin	RTTN	0.41	0.0000
218123_at	chromosome 21 open reading frame 59	C21orf59	0.45	0.0000
1555772_a_at	cell division cycle 25 homolog A (S. pombe)	CDC25A	0.31	0.0000
204058_at	malic enzyme 1, NADP(+)-dependent, cytosolic	ME1	0.29	0.0000
1555225_at	chromosome 1 open reading frame 43	C1orf43	0.14	0.0000
204151_x_at	aldo-keto reductase family 1, member C1 (dihydrodiol dehydrogenase 1; 20-alpha (3-alpha)-hydroxysteroid dehydrogenase)	AKR1C1	0.46	0.0000
206363_at	v-maf musculoaponeurotic fibrosarcoma oncogene homolog (avian)	MAF	0.17	0.0000
201467_s_at	NAD(P)H dehydrogenase, quinone 1	NQO1	0.32	0.0000
203390_s_at	kinesin family member 3C	KIF3C	0.50	0.0000
225943_at	neurolysin (metallopeptidase M3 family)	NLN	0.33	0.0000
217934_x_at	STIP1 homology and U-box containing protein 1, E3 ubiquitin protein ligase	STUB1	0.47	0.0000
225575_at	leukemia inhibitory factor receptor alpha	LIFR	0.16	0.0000
218969_at	presequence translocase-associated motor 16 homolog (S. cerevisiae)	PAM16	0.45	0.0000
223870_at	F-box and WD repeat domain containing 10	FBXW10	0.17	0.0000
229099_at	chromosome 11 open reading frame 83	C11orf83	0.48	0.0000
210463_x_at	tRNA methyltransferase 1 homolog (S. cerevisiae)	TRMT1	0.30	0.0000
226463_at	ATPase, H+ transporting, lysosomal 42kDa, V1 subunit C1	ATP6V1C1	0.32	0.0000
206441_s_at	COMM domain containing 4	COMMD4	0.39	0.0000
217992_s_at	EF-hand domain family, member D2	EFHD2	0.35	0.0000
231240_at	deiodinase, iodothyronine, type II	DIO2	0.10	0.0000
206157_at	pentraxin 3, long	PTX3	0.04	0.0000
214830_at	solute carrier family 38, member 6	SLC38A6	0.38	0.0000
215136_s_at	exosome component 8	EXOSC8	0.44	0.0000
227322_s_at	BRCA2 and CDKN1A interacting protein	BCCIP	0.31	0.0000
205273_s_at	pitrilysin metallopeptidase 1	PITRM1	0.40	0.0000
202201_at	biliverdin reductase B (flavin reductase (NADPH))	BLVRB	0.49	0.0000
206237_s_at	neuregulin 1	NRG1	0.40	0.0000
212212_s_at	integrator complex subunit 1	INTS1	0.38	0.0000
214439_x_at	bridging integrator 1	BIN1	0.35	0.0000
205409_at	FOS-like antigen 2	FOSL2	0.41	0.0000

Probeset ID	Gene title	Gene symbol	Fold change	FDR (%)
217754_at	DEAD (Asp-Glu-Ala-Asp) box helicase 56	DDX56	0.45	0.0000
222803_at	phosphoribosyl transferase domain containing 1	PRTFDC1	0.28	0.0000
216594_x_at	aldo-keto reductase family 1, member C1 (dihydrodiol dehydrogenase 1; 20-alpha (3-alpha)-hydroxysteroid dehydrogenase)	AKR1C1	0.38	0.0000
219489_s_at	nucleoredoxin	NXN	0.37	0.0000
225944_at	neurolysin (metallopeptidase M3 family)	NLN	0.50	0.0000
213331_s_at	NIMA (never in mitosis gene a)-related kinase 1	NEK1	0.27	0.0000
219014_at	placenta-specific 8	PLAC8	0.44	0.0000
203123_s_at	solute carrier family 11 (proton-coupled divalent metal ion transporters), member 2	SLC11A2	0.34	0.0000
212568_s_at	dihydrolipoamide S-acetyltransferase	DLAT	0.46	0.0000
201046_s_at	RAD23 homolog A (S. cerevisiae)	RAD23A	0.40	0.0000
213926_s_at	ArfGAP with FG repeats 1	AGFG1	0.48	0.0000
241763_s_at	F-box protein 32	FBXO32	0.11	0.0000
218472_s_at	pelota homolog (Drosophila)	PELO	0.48	0.0000
225942_at	neurolysin (metallopeptidase M3 family)	NLN	0.29	0.0000
218112_at	mitochondrial ribosomal protein S34	MRPS34	0.44	0.0000
218970_s_at	cutC copper transporter homolog (E. coli)	CUTC	0.42	0.0000
216913_s_at	ribosomal RNA processing 12 homolog (S. cerevisiae)	RRP12	0.42	0.0000
220688_s_at	mRNA turnover 4 homolog (S. cerevisiae)	MRTO4	0.41	0.0000
221805_at	neurofilament, light polypeptide	NEFL	0.49	0.0000
202643_s_at	tumor necrosis factor, alpha-induced protein 3	TNFAIP3	0.26	0.0000
205135_s_at	nuclear fragile X mental retardation protein interacting protein 1	NUFIP1	0.38	0.0000
210517_s_at	A kinase (PRKA) anchor protein 12	AKAP12	0.40	0.0000
229888_at	chromosome 12 open reading frame 60	C12orf60	0.21	0.0000
205921_s_at	solute carrier family 6 (neurotransmitter transporter, taurine), member 6	SLC6A6	0.15	0.0000
203324_s_at	caveolin 2	CAV2	0.46	0.0000
239001_at	Microsomal glutathione S-transferase 1	MGST1	0.27	0.0000
201527_at	ATPase, H+ transporting, lysosomal 14kDa, V1 subunit F	ATP6V1F	0.48	0.0000
219960_s_at	ubiquitin carboxyl-terminal hydrolase L5	UCHL5	0.46	0.0000
223225_s_at	SEH1-like (S. cerevisiae)	SEH1L	0.31	0.0000
224204_x_at	aryl hydrocarbon receptor nuclear translocator-like 2	ARNTL2	0.22	0.0000
65517_at	adaptor-related protein complex 1, mu 2 subunit	AP1M2	0.12	0.0000
208691_at	transferrin receptor (p90, CD71)	TFRC	0.40	0.0000
221711_s_at	BRISC and BRCA1 A complex member 1	BABAM1	0.44	0.0000
219245_s_at	2-oxoglutarate and iron-dependent oxygenase domain containing 2	OGFOD2	0.33	0.0000
220658_s_at	aryl hydrocarbon receptor nuclear translocator-like 2	ARNTL2	0.18	0.0000
201039_s_at	RAD23 homolog A (S. cerevisiae)	RAD23A	0.31	0.0000
211814_s_at	cyclin E2	CCNE2	0.37	0.0000
203124_s_at	solute carrier family 11 (proton-coupled divalent metal ion transporters), member 2	SLC11A2	0.36	0.0000
208676_s_at	proliferation-associated 2G4, 38kDa	PA2G4	0.46	0.0000
201939_at	polo-like kinase 2	PLK2	0.42	0.0000
221801_x_at	neurofilament, light polypeptide	NEFL	0.48	0.0000
1555229_a_at	complement component 1, s subcomponent	C1S	0.37	0.0000
224879_at	chromosome 9 open reading frame 123	C9orf123	0.49	0.0000
212919_at	DCP2 decapping enzyme homolog (S. cerevisiae)	DCP2	0.46	0.0000
231775_at	tumor necrosis factor receptor superfamily, member 10a	TNFRSF10A	0.28	0.0000
243779_at	UDP-N-acetyl-alpha-D-galactosamine:polypeptide N-acetylgalactosaminyltransferase 13 (GalNAc-T13)	GALNT13	0.46	0.0000
228810_at	cyclin Y-like 1	CCNYL1	0.22	0.0000
226206_at	v-maf musculoaponeurotic fibrosarcoma oncogene homolog K (avian)	MAFK	0.37	0.0000
234976_x_at	methylenetetrahydrofolate dehydrogenase (NADP+ dependent) 2, methenyltetrahydrofolate cyclohydrolase	MTHFD2	0.43	0.0000
221009_s_at	angiopoietin-like 4	ANGPTL4	0.25	0.0000
225638_at	chromosome 1 open reading frame 31	C1orf31	0.47	0.0000
208972_s_at	ATP synthase, H+ transporting, mitochondrial Fo complex, subunit C1 (subunit	ATP5G1	0.48	0.0000

Probeset ID	Gene title	Gene symbol	Fold change	FDR (%)
	9)			
208815_x_at	heat shock 70kDa protein 4	HSPA4	0.48	0.0000
204610_s_at	coiled-coil domain containing 85B	CCDC85B	0.42	0.0000
218481_at	exosome component 5	EXOSC5	0.44	0.0000
226901_at	chromosome 17 open reading frame 58	C17orf58	0.44	0.0000
205080_at	retinoic acid receptor, beta	RARB	0.36	0.0000
203622_s_at	partner of NOB1 homolog (S. cerevisiae)	PNO1	0.50	0.0000
203176_s_at	transcription factor A, mitochondrial	TFAM	0.43	0.0000
209100_at	interferon-related developmental regulator 2	IFRD2	0.43	0.0000
225682_s_at	polymerase (RNA) III (DNA directed) polypeptide H (22.9kD)	POLR3H	0.49	0.0000
203702_s_at	tubulin tyrosine ligase-like family, member 4	TTLL4	0.41	0.0000
203095_at	mitochondrial translational initiation factor 2	MTIF2	0.46	0.0000
203910_at	Rho GTPase activating protein 29	ARHGAP29	0.40	0.0000
210538_s_at	baculoviral IAP repeat containing 3	BIRC3	0.33	0.0000
225868_at	tripartite motif containing 47	TRIM47	0.38	0.0000
201797_s_at	valyl-tRNA synthetase	VARS	0.25	0.0000
218558_s_at	mitochondrial ribosomal protein L39	MRPL39	0.46	0.0000
208230_s_at	neuregulin 1	NRG1	0.42	0.0000
209433_s_at	phosphoribosyl pyrophosphate amidotransferase	PPAT	0.45	0.0000
209486_at	UTP3, small subunit (SSU) processome component, homolog (S. cerevisiae)	UTP3	0.48	0.0000
226089_at	RAB, member of RAS oncogene family-like 3	RABL3	0.40	0.0000
206795_at	coagulation factor II (thrombin) receptor-like 2	F2RL2	0.07	0.0000
224996_at	aspartate beta-hydroxylase	ASPH	0.49	0.0000
219295_s_at	procollagen C-endopeptidase enhancer 2	PCOLCE2	0.36	0.0000
226350_at	choroideremia-like (Rab escort protein 2)	CHML	0.45	0.0000
201323_at	EBNA1 binding protein 2	EBNA1BP2	0.48	0.0000
227037_at	phospholipase D family, member 6	PLD6	0.17	0.0000
207536_s_at	tumor necrosis factor receptor superfamily, member 9	TNFRSF9	0.10	0.0000
242560_at	Fanconi anemia, complementation group D2	FANCD2	0.42	0.0000
211071_s_at	myeloid/lymphoid or mixed-lineage leukemia (trithorax homolog, Drosophila); translocated to, 11	MLLT11	0.45	0.0000
229908_s_at	unkempt homolog (Drosophila)-like	UNKL	0.20	0.0000
236273_at	neuroblastoma breakpoint family, member 1	NBPF1	0.28	0.0000
218512_at	WD repeat domain 12	WDR12	0.46	0.0000
213554_s_at	CDV3 homolog (mouse)	CDV3	0.45	0.0000
203867_s_at	notchless homolog 1 (Drosophila)	NLE1	0.45	0.0000
227530_at	A kinase (PRKA) anchor protein 12	AKAP12	0.46	0.0000
222483_at	EF-hand domain family, member D2	EFHD2	0.29	0.0000
202805_s_at	ATP-binding cassette, sub-family C (CFTR/MRP), member 1	ABCC1	0.42	0.0000
209592_s_at	DDB1 and CUL4 associated factor 7	DCAF7	0.47	0.0000
223241_at	sorting nexin 8	SNX8	0.26	0.0000
202431_s_at	v-myc myelocytomatosis viral oncogene homolog (avian)	MYC	0.48	0.0000
209233_at	EMG1 nucleolar protein homolog (S. cerevisiae)	EMG1	0.42	0.0000
217833_at	synaptotagmin binding, cytoplasmic RNA interacting protein	SYNCRIP	0.38	0.0000
1555788_a_at	tribbles homolog 3 (Drosophila)	TRIB3	0.49	0.0000
214205_x_at	glutaredoxin 3	GLRX3	0.47	0.0000
218398_at	mitochondrial ribosomal protein S30	MRPS30	0.42	0.0000
223452_s_at	atlastin GTPase 3	ATL3	0.42	0.0000
226479_at	kelch repeat and BTB (POZ) domain containing 6	KBTBD6	0.42	0.0000
221802_s_at	KIAA1598	KIAA1598	0.38	0.0000
1553962_s_at	ras homolog family member B	RHOB	0.48	0.0000
209457_at	dual specificity phosphatase 5	DUSP5	0.46	0.0000
225492_at	transmembrane protein 33	TMEM33	0.50	0.0000
1555679_a_at	reticulon 4 interacting protein 1	RTN4IP1	0.49	0.0000
224480_s_at	1-acylglycerol-3-phosphate O-acyltransferase 9	AGPAT9	0.24	0.0000
224738_x_at	ribosomal protein L7-like 1	RPL7L1	0.48	0.0000

Probeset ID	Gene title	Gene symbol	Fold change	FDR (%)
211162_x_at	stearoyl-CoA desaturase (delta-9-desaturase)	SCD	0.40	0.0000
224833_at	v-ets erythroblastosis virus E26 oncogene homolog 1 (avian)	ETS1	0.32	0.0000
206561_s_at	aldo-keto reductase family 1, member B10 (aldose reductase)	AKR1B10	0.32	0.0000
224461_s_at	apoptosis-inducing factor, mitochondrion-associated, 2	AIFM2	0.30	0.0000
222479_s_at	dynein, cytoplasmic 1, light intermediate chain 1	DYNC1LI1	0.49	0.0000
210048_at	N-ethylmaleimide-sensitive factor attachment protein, gamma	NAPG	0.36	0.0000
203947_at	cleavage stimulation factor, 3' pre-RNA, subunit 3, 77kDa	CSTF3	0.43	0.0000
228754_at	solute carrier family 6 (neurotransmitter transporter, taurine), member 6	SLC6A6	0.46	0.0000
219066_at	phosphopantothenoylcysteine decarboxylase	PPCDC	0.23	0.0000
227034_at	sosondowah ankyrin repeat domain family member C	SOWAHC	0.43	0.0000
201468_s_at	NAD(P)H dehydrogenase, quinone 1	NQO1	0.45	0.0000
218027_at	mitochondrial ribosomal protein L15	MRPL15	0.46	0.0000
223461_at	TBC1 domain family, member 7	TBC1D7	0.44	0.0000
1554469_at	zinc finger and BTB domain containing 44	ZBTB44	0.42	0.0000
209029_at	COP9 constitutive photomorphogenic homolog subunit 7A (Arabidopsis)	COPS7A	0.48	0.0000
219071_x_at	family with sequence similarity 203, member A	FAM203A	0.46	0.0000
33304_at	interferon stimulated exonuclease gene 20kDa	ISG20	0.27	0.0000
206343_s_at	neuregulin 1	NRG1	0.35	0.0000
225930_at	NFKB inhibitor interacting Ras-like 1	NKIRAS1	0.38	0.0000
223532_at	ankyrin repeat domain 39	ANKRD39	0.27	0.0000
202054_s_at	aldehyde dehydrogenase 3 family, member A2	ALDH3A2	0.48	0.0000
230466_s_at	Ras association (RalGDS/AF-6) domain family member 3	RASSF3	0.27	0.0000
218774_at	decapping enzyme, scavenger	DCPS	0.38	0.0000
213457_at	malignant fibrous histiocytoma amplified sequence 1	MFHAS1	0.46	0.0000
230298_at	metallo-beta-lactamase domain containing 2	MBLAC2	0.29	0.0000
1554538_at	ras homolog family member F (in filopodia)	RHOF	0.24	0.0000
1555355_a_at	v-ets erythroblastosis virus E26 oncogene homolog 1 (avian)	ETS1	0.34	0.0000
221916_at	neurofilament, light polypeptide	NEFL	0.47	0.0000
204616_at	ubiquitin carboxyl-terminal esterase L3 (ubiquitin thiolesterase)	UCHL3	0.49	0.0000
214695_at	ubiquitin associated protein 2-like	UBAP2L	0.45	0.0000
210201_x_at	bridging integrator 1	BIN1	0.39	0.0000
232269_x_at	meteorin, glial cell differentiation regulator	METRN	0.41	0.0000
204435_at	nucleoporin like 1	NUPL1	0.49	0.0000
225520_at	methylenetetrahydrofolate dehydrogenase (NADP+ dependent) 1-like	MTHFD1L	0.49	0.0000
214045_at	lipoic acid synthetase	LIAS	0.26	0.0000
205047_s_at	asparagine synthetase (glutamine-hydrolyzing)	ASNS	0.42	0.0000
1554539_a_at	ras homolog family member F (in filopodia)	RHOF	0.43	0.0000
210202_s_at	bridging integrator 1	BIN1	0.35	0.0000
204005_s_at	PRKC, apoptosis, WT1, regulator	PAWR	0.42	0.0000
202847_at	phosphoenolpyruvate carboxykinase 2 (mitochondrial)	PCK2	0.48	0.0000
209882_at	Ras-like without CAAX 1	RIT1	0.39	0.0000
225195_at	DPH3, KTI11 homolog (S. cerevisiae)	DPH3	0.48	0.0000
221534_at	chromosome 11 open reading frame 68	C11orf68	0.42	0.0000
218653_at	solute carrier family 25 (mitochondrial carrier; ornithine transporter) member 15	SLC25A15	0.39	0.0000
201466_s_at	jun proto-oncogene	JUN	0.42	0.0000
209017_s_at	lon peptidase 1, mitochondrial	LONP1	0.40	0.0000
243011_at	neurexophilin and PC-esterase domain family, member 3	NXPE3	0.34	0.0000
203814_s_at	NAD(P)H dehydrogenase, quinone 2	NQO2	0.42	0.0000
201890_at	ribonucleotide reductase M2	RRM2	0.48	0.0000
202804_at	ATP-binding cassette, sub-family C (CFTR/MRP), member 1	ABCC1	0.47	0.0000
242283_at	dynein, axonemal, heavy chain 14	DNAH14	0.45	0.0000
204224_s_at	GTP cyclohydrolase 1	GCH1	0.37	0.0000
223308_s_at	WD repeat domain 5	WDR5	0.50	0.0000
218721_s_at	chromosome 1 open reading frame 27	C1orf27	0.47	0.0000
201289_at	cysteine-rich, angiogenic inducer, 61	CYR61	0.42	0.0000

Probeset ID	Gene title	Gene symbol	Fold change	FDR (%)
223249_at	claudin 12	CLDN12	0.37	0.0000
209773_s_at	ribonucleotide reductase M2	RRM2	0.49	0.0000
211016_x_at	heat shock 70kDa protein 4	HSPA4	0.44	0.0000
227718_at	purine-rich element binding protein B	PURB	0.44	0.0000
206106_at	mitogen-activated protein kinase 12	MAPK12	0.36	0.0000
1564651_at	chromosome 6 open reading frame 228	C6orf228	0.45	0.0000
220239_at	kelch-like 7 (Drosophila)	KLHL7	0.35	0.0000
223586_at	aryl hydrocarbon receptor nuclear translocator-like 2	ARNTL2	0.38	0.0000
227296_at	major facilitator superfamily domain containing 3	MFSD3	0.49	0.0000
218404_at	sorting nexin 10	SNX10	0.44	0.0000
223181_at	translocase of inner mitochondrial membrane 21 homolog (yeast)	TIMM21	0.50	0.0000
208711_s_at	cyclin D1	CCND1	0.45	0.0000
224185_at	WD repeat containing, antisense to TP53	WRAP53	0.29	0.0000
210896_s_at	aspartate beta-hydroxylase	ASPH	0.50	0.0000
1555834_at	Ubiquitin carboxyl-terminal esterase L1 (ubiquitin thiolesterase)	UCHL1	0.19	0.0000
1568574_x_at	Secreted phosphoprotein 1	SPP1	0.07	0.0000
210544_s_at	aldehyde dehydrogenase 3 family, member A2	ALDH3A2	0.48	0.0000
217965_s_at	SAP30 binding protein	SAP30BP	0.49	0.0000
205767_at	epiregulin	EREG	0.38	0.0000
225419_at	M-phase specific PLK1 interacting protein	MPLKIP	0.50	0.0000
223145_s_at	akirin 2	AKIRIN2	0.48	0.0000
231849_at	keratin 80	KRT80	0.41	0.0000
209743_s_at	itchy E3 ubiquitin protein ligase	ITCH	0.41	0.0000
219006_at	NADH dehydrogenase (ubiquinone) complex I, assembly factor 4	NDUFAF4	0.45	0.0000
235258_at	DCP2 decapping enzyme homolog (S. cerevisiae)	DCP2	0.28	0.0000
226285_at	cell cycle associated protein 1	CAPRIN1	0.23	0.0000
207980_s_at	Cbp/p300-interacting transactivator, with Glu/Asp-rich carboxy-terminal domain, 2	CITED2	0.48	0.0000
212445_s_at	neural precursor cell expressed, developmentally down-regulated 4-like, E3 ubiquitin protein ligase	NEDD4L	0.45	0.0000
211015_s_at	heat shock 70kDa protein 4	HSPA4	0.49	0.0000
221712_s_at	WD repeat domain 74	WDR74	0.43	0.0000
210253_at	HIV-1 Tat interactive protein 2, 30kDa	HTATIP2	0.28	0.0000
233085_s_at	nucleic acid binding protein 1	NABP1	0.43	0.0000
223689_at	insulin-like growth factor 2 mRNA binding protein 1	IGF2BP1	0.38	0.0000
218581_at	abhydrolase domain containing 4	ABHD4	0.46	0.0000
218936_s_at	coiled-coil domain containing 59	CCDC59	0.47	0.0000
202776_at	deoxynucleotidyltransferase, terminal, interacting protein 2	DNTTIP2	0.46	0.0000
226435_at	papilin, proteoglycan-like sulfated glycoprotein	PAPLN	0.16	0.0000
208967_s_at	adenylate kinase 2	AK2	0.43	0.0000
1565951_s_at	choroideremia-like (Rab escort protein 2)	CHML	0.49	0.0000
201535_at	ubiquitin-like 3	UBL3	0.46	0.0000
218480_at	ATP/GTP binding protein-like 5	AGBL5	0.47	0.0000
242470_at	EP300 interacting inhibitor of differentiation 2B	EID2B	0.34	0.0000
212619_at	transmembrane protein 194A	TMEM194A	0.49	0.0000
207535_s_at	nuclear factor of kappa light polypeptide gene enhancer in B-cells 2 (p49/p100)	NFKB2	0.16	0.0000
214437_s_at	serine hydroxymethyltransferase 2 (mitochondrial)	SHMT2	0.33	0.0000
238574_at	solute carrier family 25, member 51	SLC25A51	0.40	0.0000
212590_at	related RAS viral (r-ras) oncogene homolog 2	RRAS2	0.47	0.0000
229113_s_at	chromosome 1 open reading frame 86	C1orf86	0.49	0.0000
201534_s_at	ubiquitin-like 3	UBL3	0.47	0.0000
214435_x_at	v-ral simian leukemia viral oncogene homolog A (ras related)	RALA	0.46	0.0000
230499_at	baculoviral IAP repeat containing 3	BIRC3	0.41	0.0000
208541_x_at	transcription factor A, mitochondrial	TFAM	0.42	0.0000
1558292_s_at	phosphatidylinositol glycan anchor biosynthesis, class W	PIGW	0.36	0.0000

Probeset ID	Gene title	Gene symbol	Fold change	FDR (%)
209797_at	canopy 2 homolog (zebrafish)	CNPY2	0.36	0.0000
219928_s_at	calcium binding tyrosine-(Y)-phosphorylation regulated	CABYR	0.46	0.0000
209434_s_at	phosphoribosyl pyrophosphate amidotransferase	PPAT	0.38	0.0000
244293_at	ubiquinol-cytochrome c reductase binding protein	UQCRB	0.49	0.0000
214960_at	apoptosis inhibitor 5	API5	0.39	0.0000
210570_x_at	mitogen-activated protein kinase 9	MAPK9	0.36	0.0000
218156_s_at	TSR1, 20S rRNA accumulation, homolog (S. cerevisiae)	TSR1	0.45	0.0000
205205_at	v-rel reticuloendotheliosis viral oncogene homolog B	RELB	0.34	0.0000
209461_x_at	WD repeat domain 18	WDR18	0.47	0.0000
213897_s_at	mitochondrial ribosomal protein L23	MRPL23	0.49	0.0000
219770_at	glycosyltransferase-like domain containing 1	GTDC1	0.27	0.0000
200849_s_at	adenosylhomocysteinase-like 1	AHCYL1	0.41	0.0000
211708_s_at	stearoyl-CoA desaturase (delta-9-desaturase)	SCD	0.40	0.0000
1555972_s_at	F-box protein 28	FBXO28	0.48	0.0000
205264_at	CD3e molecule, epsilon associated protein	CD3EAP	0.40	0.0000
201422_at	interferon, gamma-inducible protein 30	IFI30	0.40	0.0000
229551_x_at	zinc finger protein 367	ZNF367	0.47	0.0000
201563_at	sorbitol dehydrogenase	SORD	0.42	0.0000
1554014_at	chromodomain helicase DNA binding protein 2	CHD2	0.35	0.0000
206515_at	cytochrome P450, family 4, subfamily F, polypeptide 3	CYP4F3	0.07	0.0000
200637_s_at	protein tyrosine phosphatase, receptor type, F	PTPRF	0.47	0.0000
202167_s_at	MMS19 nucleotide excision repair homolog (S. cerevisiae)	MMS19	0.43	0.0000
226882_x_at	WD repeat domain 4	WDR4	0.50	0.0000
211668_s_at	plasminogen activator, urokinase	PLAU	0.45	0.0000
204802_at	Ras-related associated with diabetes	RRAD	0.09	0.0000
203705_s_at	frizzled family receptor 7	FZD7	0.44	0.0000
201937_s_at	aspartyl aminopeptidase	DNPEP	0.42	0.0000
219863_at	HECT and RLD domain containing E3 ubiquitin protein ligase 5	HERC5	0.36	0.0000
238585_at	glycosyltransferase-like domain containing 1	GTDC1	0.24	0.0000
223682_s_at	eukaryotic translation initiation factor 1A domain containing	EIF1AD	0.37	0.0000
221931_s_at	SEH1-like (S. cerevisiae)	SEH1L	0.49	0.0000
208864_s_at	thioredoxin	TXN	0.48	0.0000
205129_at	nucleophosmin/nucleoplasmin 3	NPM3	0.37	0.0000
219177_at	BRX1, biogenesis of ribosomes, homolog (S. cerevisiae)	BRIX1	0.44	0.0000
206550_s_at	nucleoporin 155kDa	NUP155	0.37	0.0000
202144_s_at	adenylosuccinate lyase	ADSL	0.46	0.0000
223413_s_at	Ly1 antibody reactive homolog (mouse)	LYAR	0.40	0.0000
202802_at	deoxyhypusine synthase	DHPS	0.39	0.0000
223368_s_at	N-terminal Xaa-Pro-Lys N-methyltransferase 1	NTMT1	0.49	0.0000
234472_at	UDP-N-acetyl-alpha-D-galactosamine:polypeptide N-acetylgalactosaminyltransferase 13 (GalNAc-T13)	GALNT13	0.36	0.0000
218886_at	PAK1 interacting protein 1	PAK1IP1	0.49	0.0000
203162_s_at	katanin p80 (WD repeat containing) subunit B 1	KATNB1	0.49	0.0000
209418_s_at	THO complex 5	THOC5	0.41	0.0000
210008_s_at	mitochondrial ribosomal protein S12	MRPS12	0.46	0.0000
218170_at	isochorismatase domain containing 1	ISOC1	0.49	0.0000
204218_at	anaphase promoting complex subunit 15	ANAPC15	0.47	0.0000
211686_s_at	MAK16 homolog (S. cerevisiae)	MAK16	0.47	0.0000
223743_s_at	mitochondrial ribosomal protein L4	MRPL4	0.47	0.0000
235911_at	antigen p97 (melanoma associated) identified by monoclonal antibodies 133.2 and 96.5	MFI2	0.35	0.0000
209891_at	SPC25, NDC80 kinetochore complex component, homolog (S. cerevisiae)	SPC25	0.46	0.0000
210802_s_at	DIM1 dimethyladenosine transferase 1 homolog (S. cerevisiae)	DIMT1	0.49	0.0000
238332_at	ankyrin repeat domain 29	ANKRD29	0.30	0.0000
204608_at	argininosuccinate lyase	ASL	0.26	0.0000
203192_at	ATP-binding cassette, sub-family B (MDR/TAP), member 6	ABCB6	0.14	0.0000

Probeset ID	Gene title	Gene symbol	Fold change	FDR (%)
211020_at	glucosaminyl (N-acetyl) transferase 2, I-branching enzyme (I blood group)	GCNT2	0.45	0.0000
226876_at	family with sequence similarity 101, member B	FAM101B	0.11	0.0000
219634_at	carbohydrate (chondroitin 4) sulfotransferase 11	CHST11	0.44	0.0000
202686_s_at	AXL receptor tyrosine kinase	AXL	0.49	0.0000
206961_s_at	mediator complex subunit 20	MED20	0.42	0.0000
204521_at	family with sequence similarity 216, member A	FAM216A	0.30	0.0000
203323_at	caveolin 2	CAV2	0.47	0.0000
226258_at	antagonist of mitotic exit network 1 homolog (S. cerevisiae)	AMN1	0.17	0.0000
225103_at	mitochondrial ribosomal protein L38	MRPL38	0.46	0.0000
204971_at	cystatin A (stefin A)	CSTA	0.39	0.0000
242260_at	Matrin 3	MATR3	0.45	0.0000
218566_s_at	cysteine and histidine-rich domain (CHORD) containing 1	CHORDC1	0.40	0.0000
203218_at	mitogen-activated protein kinase 9	MAPK9	0.39	0.0000
202884_s_at	protein phosphatase 2, regulatory subunit A, beta	PPP2R1B	0.48	0.0000
223427_s_at	erythrocyte membrane protein band 4.1 like 4B	EPB41L4B	0.49	0.0000
231914_at	nudix (nucleoside diphosphate linked moiety X)-type motif 14	NUDT14	0.44	0.0000
55081_at	MICAL-like 1	MICALL1	0.43	0.0000
203782_s_at	polymerase (RNA) mitochondrial (DNA directed)	POLRMT	0.40	0.0000
222825_at	OTU domain containing 6B	OTUD6B	0.38	0.0000
236224_at	Ras-like without CAAX 1	RIT1	0.47	0.0000
224535_s_at	mitochondrial ribosomal protein 63	MRP63	0.49	0.0000
229181_s_at	HAUS augmin-like complex, subunit 2	HAUS2	0.47	0.0000
203706_s_at	frizzled family receptor 7	FZD7	0.49	0.0000
218305_at	importin 4	IPO4	0.41	0.0000
38703_at	aspartyl aminopeptidase	DNPEP	0.48	0.0000
219496_at	sosondowah ankyrin repeat domain family member C	SOWAHC	0.35	0.0000
212434_at	GrpE-like 1, mitochondrial (E. coli)	GRPEL1	0.46	0.0000
206307_s_at	forkhead box D1	FOXD1	0.40	0.0000
223414_s_at	Ly1 antibody reactive homolog (mouse)	LYAR	0.49	0.0000
201562_s_at	sorbitol dehydrogenase	SORD	0.36	0.0000
213615_at	Lysophosphatidylcholine acyltransferase 3	LPCAT3	0.44	0.0000
203573_s_at	Rab geranylgeranyltransferase, alpha subunit	RABGGTA	0.43	0.0000
204565_at	acyl-CoA thioesterase 13	ACOT13	0.40	0.0000
224521_s_at	coiled-coil domain containing 77	CCDC77	0.46	0.0000
227214_at	golgi-associated PDZ and coiled-coil motif containing	GOPC	0.46	0.0000
214091_s_at	glutathione peroxidase 3 (plasma)	GPX3	0.15	0.0000
205479_s_at	plasminogen activator, urokinase	PLAU	0.43	0.0000
223143_s_at	akirin 2	AKIRIN2	0.48	0.0000
225803_at	F-box protein 32	FBXO32	0.39	0.0000
223785_at	Fanconi anemia, complementation group I	FANCI	0.47	0.0000
218479_s_at	exportin 4	XPO4	0.15	0.0000
227818_at	centrosomal protein 85kDa	CEP85	0.46	0.0000
229075_at	spermatogenesis associated 5	SPATA5	0.28	0.0000
220651_s_at	minichromosome maintenance complex component 10	MCM10	0.48	0.0000
208478_s_at	BCL2-associated X protein	BAX	0.34	0.0000
233192_s_at	RUN and FYVE domain containing 2	RUFY2	0.29	0.0000
226289_at	cell cycle associated protein 1	CAPRIN1	0.25	0.0000
235011_at	mitogen-activated protein kinase kinase kinase 2	MAP3K2	0.37	0.0000
212603_at	mitochondrial ribosomal protein S31	MRPS31	0.49	0.0000
1554085_at	DEAD (Asp-Glu-Ala-Asp) box polypeptide 51	DDX51	0.49	0.0000
220370_s_at	ubiquitin specific peptidase 36	USP36	0.36	0.0000
210732_s_at	lectin, galactoside-binding, soluble, 8	LGALS8	0.40	0.0000
238121_at	glycerol kinase 5 (putative)	GK5	0.47	0.0000
210811_s_at	DEAD (Asp-Glu-Ala-Asp) box polypeptide 49	DDX49	0.46	0.0000
1554082_a_at	nucleolar protein 9	NOL9	0.43	0.0000

Probeset ID	Gene title	Gene symbol	Fold change	FDR (%)
204657_s_at	Src homology 2 domain containing adaptor protein B	SHB	0.38	0.0000
229970_at	kelch repeat and BTB (POZ) domain containing 7	KBTBD7	0.33	0.0000
200850_s_at	adenosylhomocysteinase-like 1	AHCYL1	0.50	0.0000
214310_s_at	zinc finger protein-like 1	ZFPL1	0.46	0.0000
226483_at	transmembrane protein 68	TMEM68	0.49	0.0000
1556021_at	G protein-coupled receptor 180	GPR180	0.37	0.0000
203632_s_at	G protein-coupled receptor, family C, group 5, member B	GPRC5B	0.21	0.0000
231779_at	interleukin-1 receptor-associated kinase 2	IRAK2	0.42	0.0000
1554555_a_at	SET domain containing 6	SETD6	0.36	0.0000
212635_at	transportin 1	TNPO1	0.47	0.0000
212024_x_at	flightless I homolog (Drosophila)	FLII	0.47	0.0000
243176_at	ADP-ribosylation factor-like 5A	ARL5A	0.35	0.0000
216397_s_at	block of proliferation 1	BOP1	0.48	0.0000
33778_at	TBC1 domain family, member 22A	TBC1D22A	0.48	0.0000
205748_s_at	ring finger protein 126	RNF126	0.48	0.0000
226231_at	PRKC, apoptosis, WT1, regulator	PAWR	0.42	0.0000
205103_at	chromosome 1 open reading frame 61	C1orf61	0.31	0.0000
210187_at	FK506 binding protein 1A, 12kDa	FKBP1A	0.48	0.0000
215711_s_at	WEE1 homolog (S. pombe)	WEE1	0.49	0.0000
210764_s_at	cysteine-rich, angiogenic inducer, 61	CYR61	0.32	0.0000
202756_s_at	glypican 1	GPC1	0.44	0.0000
210250_x_at	adenylosuccinate lyase	ADSL	0.47	0.0000
205214_at	serine/threonine kinase 17b	STK17B	0.35	0.0000
202873_at	ATPase, H+ transporting, lysosomal 42kDa, V1 subunit C1	ATP6V1C1	0.23	0.0000
221586_s_at	E2F transcription factor 5, p130-binding	E2F5	0.45	0.0000
239329_at	RAB21, member RAS oncogene family	RAB21	0.41	0.0000
205770_at	glutathione reductase	GSR	0.33	0.0000
223151_at	DCN1, defective in cullin neddylation 1, domain containing 5 (S. cerevisiae)	DCUN1D5	0.49	0.0000
210567_s_at	S-phase kinase-associated protein 2, E3 ubiquitin protein ligase	SKP2	0.48	0.0000
223159_s_at	NIMA (never in mitosis gene a)-related kinase 6	NEK6	0.48	0.0000
222151_s_at	centrosomal protein 63kDa	CEP63	0.48	0.0000
213607_x_at	NAD kinase	NADK	0.50	0.0000
218253_s_at	eukaryotic translation initiation factor 2D	EIF2D	0.49	0.0000
1558924_s_at	CAP-GLY domain containing linker protein 1	CLIP1	0.50	0.0000
208918_s_at	NAD kinase	NADK	0.48	0.0000
203418_at	cyclin A2	CCNA2	0.50	0.0000
218758_s_at	ribosomal RNA processing 1 homolog (S. cerevisiae)	RRP1	0.47	0.0000
209509_s_at	dolichyl-phosphate (UDP-N-acetylglucosamine) N-acetylglucosaminephosphotransferase 1 (GlcNAc-1-P transferase)	DPAGT1	0.37	0.0000
212604_at	mitochondrial ribosomal protein S31	MRPS31	0.48	0.0000
222872_x_at	nucleic acid binding protein 1	NABP1	0.47	0.0000
214727_at	breast cancer 2, early onset	BRCA2	0.42	0.0000
225448_at	N-ethylmaleimide-sensitive factor attachment protein, gamma	NAPG	0.49	0.0000
203564_at	Fanconi anemia, complementation group G	FANCG	0.48	0.0000
214095_at	serine hydroxymethyltransferase 2 (mitochondrial)	SHMT2	0.17	0.0000
37577_at	Rho GTPase activating protein 19	ARHGAP19	0.37	0.0000
228050_at	UTP15, U3 small nucleolar ribonucleoprotein, homolog (S. cerevisiae)	UTP15	0.49	0.0000
200848_at	adenosylhomocysteinase-like 1	AHCYL1	0.42	0.0000
210367_s_at	prostaglandin E synthase	PTGES	0.49	0.0000
1553947_at	exosome component 6	EXOSC6	0.45	0.0000
227529_s_at	A kinase (PRKA) anchor protein 12	AKAP12	0.44	0.0000
238475_at	asparagine-linked glycosylation 10, alpha-1,2-glucosyltransferase homolog B (yeast)	ALG10B	0.39	0.0000
204472_at	GTP binding protein overexpressed in skeletal muscle	GEM	0.40	0.0000
205034_at	cyclin E2	CCNE2	0.39	0.0000
219944_at	CAP-GLY domain containing linker protein family, member 4	CLIP4	0.42	0.0000

Probeset ID	Gene title	Gene symbol	Fold change	FDR (%)
219334_s_at	nucleic acid binding protein 1	NABP1	0.48	0.0000
202644_s_at	tumor necrosis factor, alpha-induced protein 3	TNFAIP3	0.36	0.0000
233748_x_at	protein kinase, AMP-activated, gamma 2 non-catalytic subunit	PRKAG2	0.37	0.0000
206919_at	ELK4, ETS-domain protein (SRF accessory protein 1)	ELK4	0.41	0.0000
227740_at	U2AF homology motif (UHM) kinase 1	UHMK1	0.49	0.0000
204416_x_at	apolipoprotein C-I	APOC1	0.41	0.0000
218996_at	TCF3 (E2A) fusion partner (in childhood Leukemia)	TFPT	0.47	0.0000
223700_at	meiotic nuclear divisions 1 homolog (S. cerevisiae)	MND1	0.48	0.0000
207657_x_at	transportin 1	TNPO1	0.45	0.0000
218723_s_at	regulator of cell cycle	RGCC	0.17	0.0000
202672_s_at	activating transcription factor 3	ATF3	0.44	0.0000
236518_at	KIAA1984	KIAA1984	0.40	0.0000
205590_at	RAS guanyl releasing protein 1 (calcium and DAG-regulated)	RASGRP1	0.15	0.0000
224509_s_at	reticulon 4 interacting protein 1	RTN4IP1	0.50	0.0000
225827_at	eukaryotic translation initiation factor 2C, 2	EIF2C2	0.50	0.0000
219825_at	cytochrome P450, family 26, subfamily B, polypeptide 1	CYP26B1	0.50	0.0000
216360_x_at	ribosomal RNA processing 12 homolog (S. cerevisiae)	RRP12	0.39	0.0000
230129_at	phosphoseryl-tRNA kinase	PSTK	0.40	0.0000
206376_at	solute carrier family 6 (neutral amino acid transporter), member 15	SLC6A15	0.41	0.0000
228543_at	PET117 homolog (S. cerevisiae)	PET117	0.44	0.0000
206308_at	tRNA aspartic acid methyltransferase 1	TRDMT1	0.45	0.0000
202679_at	Niemann-Pick disease, type C1	NPC1	0.50	0.0000
205196_s_at	adaptor-related protein complex 1, sigma 1 subunit	AP1S1	0.42	0.0000
223365_at	DEAH (Asp-Glu-Ala-His) box polypeptide 37	DHX37	0.46	0.0000
229349_at	lin-28 homolog B (C. elegans)	LIN28B	0.47	0.0000
219480_at	snail homolog 1 (Drosophila)	SNAI1	0.15	0.0000
212911_at	DnaJ (Hsp40) homolog, subfamily C, member 16	DNAJC16	0.38	0.0000
212828_at	synaptojanin 2	SYNJ2	0.48	0.0000
205876_at	leukemia inhibitory factor receptor alpha	LIFR	0.45	0.0000
221987_s_at	TSR1, 20S rRNA accumulation, homolog (S. cerevisiae)	TSR1	0.39	0.0000
1558163_at	peroxisomal biogenesis factor 13	PEX13	0.29	0.0000
203626_s_at	S-phase kinase-associated protein 2, E3 ubiquitin protein ligase	SKP2	0.49	0.0000
219475_at	oxidative stress induced growth inhibitor 1	OSGIN1	0.03	0.0000
205195_at	adaptor-related protein complex 1, sigma 1 subunit	AP1S1	0.46	0.0000
243606_at	neurexophilin and PC-esterase domain family, member 3	NXPE3	0.45	0.0000
235165_at	par-6 partitioning defective 6 homolog beta (C. elegans)	PARD6B	0.41	0.0000
209226_s_at	transportin 1	TNPO1	0.44	0.0000
226839_at	nuclear receptor 2C2-associated protein	NR2C2AP	0.44	0.0000
228107_at	uncharacterized LOC100127983	LOC1001279 83	0.46	0.0000
238662_at	ATP binding domain 4	ATPBD4	0.37	0.0000
231969_at	storkhead box 2	STOX2	0.33	0.0000
217540_at	neurexophilin and PC-esterase domain family, member 3	NXPE3	0.38	0.0000
211767_at	GINS complex subunit 4 (Sld5 homolog)	GINS4	0.36	0.0000
218860_at	nucleolar complex associated 4 homolog (S. cerevisiae)	NOC4L	0.47	0.0000
214161_at	Oxidative stress induced growth inhibitor family member 2	OSGIN2	0.27	0.0000
231871_at	G protein-coupled receptor 180	GPR180	0.49	0.0000
227266_s_at	FYN binding protein	FYB	0.39	0.0000
212432_at	GrpE-like 1, mitochondrial (E. coli)	GRPEL1	0.48	0.0000
209753_s_at	thymopoietin	TMPO	0.49	0.0000
224469_s_at	inverted formin, FH2 and WH2 domain containing	INF2	0.17	0.0000
1566257_at	G protein-coupled receptor 180	GPR180	0.30	0.0000
220253_s_at	low density lipoprotein receptor-related protein 12	LRP12	0.49	0.0000
209744_x_at	itchy E3 ubiquitin protein ligase	ITCH	0.48	0.0000
225056_at	signal-induced proliferation-associated 1 like 2	SIPA1L2	0.50	0.0000
1557257_at	B-cell CLL/lymphoma 10	BCL10	0.44	0.0000

Probeset ID	Gene title	Gene symbol	Fold change	FDR (%)
218756_s_at	dehydrogenase/reductase (SDR family) member 11	DHRS11	0.38	0.0000
204279_at	proteasome (prosome, macropain) subunit, beta type, 9 (large multifunctional peptidase 2)	PSMB9	0.31	0.0000
1554080_at	RCD1 required for cell differentiation1 homolog (S. pombe)	RQCD1	0.45	0.0000
203422_at	polymerase (DNA directed), delta 1, catalytic subunit	POLD1	0.39	0.0000
222847_s_at	egl nine homolog 3 (C. elegans)	EGLN3	0.41	0.0000
228931_at	coenzyme Q4 homolog (S. cerevisiae)	COQ4	0.41	0.0000
225980_at	chromosome 14 open reading frame 43	C14orf43	0.47	0.0000
206066_s_at	RAD51 homolog C (S. cerevisiae)	RAD51C	0.48	0.0000
200961_at	selenophosphate synthetase 2	SEPHS2	0.47	0.0000
231920_s_at	casein kinase 1, gamma 1	CSNK1G1	0.47	0.0000
1554980_a_at	activating transcription factor 3	ATF3	0.47	0.0000
233587_s_at	signal-induced proliferation-associated 1 like 2	SIPA1L2	0.40	0.0000
240806_at	Ribosomal protein L15	RPL15	0.40	0.0000
211124_s_at	KIT ligand	KITLG	0.47	0.0000
204995_at	cyclin-dependent kinase 5, regulatory subunit 1 (p35)	CDK5R1	0.46	0.0000
222029_x_at	prefoldin subunit 6	PFDN6	0.49	0.0000
203387_s_at	TBC1 domain family, member 4	TBC1D4	0.48	0.0000
205100_at	glutamine-fructose-6-phosphate transaminase 2	GFPT2	0.29	0.0000
218486_at	Kruppel-like factor 11	KLF11	0.47	0.0000
203577_at	general transcription factor IIH, polypeptide 4, 52kDa	GTF2H4	0.34	0.0000
209635_at	adaptor-related protein complex 1, sigma 1 subunit	AP1S1	0.49	0.0000
39835_at	SET binding factor 1	SBF1	0.49	0.0000
209489_at	CUGBP, Elav-like family member 1	CELF1	0.46	0.0000
208170_s_at	tripartite motif containing 31	TRIM31	0.36	0.0000
227197_at	Rho guanine nucleotide exchange factor (GEF) 26	ARHGEF26	0.40	0.0000
205403_at	interleukin 1 receptor, type II	IL1R2	0.24	0.0000
218602_s_at	HAUS augmin-like complex, subunit 6	HAUS6	0.50	0.0000
210015_s_at	microtubule-associated protein 2	MAP2	0.36	0.0000
208433_s_at	low density lipoprotein receptor-related protein 8, apolipoprotein e receptor	LRP8	0.49	0.0000
214649_s_at	myotubularin related protein 2	MTMR2	0.48	0.0000
224229_s_at	v-akt murine thymoma viral oncogene homolog 3 (protein kinase B, gamma)	AKT3	0.50	0.0000
204803_s_at	Ras-related associated with diabetes	RRAD	0.14	0.0000
222065_s_at	flightless I homolog (Drosophila)	FLII	0.47	0.0000
238012_at	Dipeptidyl-peptidase 7	DPP7	0.42	0.0000
204330_s_at	mitochondrial ribosomal protein S12	MRPS12	0.43	0.0000
219513_s_at	SH2 domain containing 3A	SH2D3A	0.41	0.0000
209428_s_at	zinc finger protein-like 1	ZFPL1	0.38	0.0000
232746_at	Chemokine (C-X-C motif) receptor 7	CXCR7	0.18	0.0000
220934_s_at	transmembrane protein 223	TMEM223	0.46	0.0000
211833_s_at	BCL2-associated X protein	BAX	0.34	0.0000
226152_at	tetratricopeptide repeat domain 7B	TTC7B	0.28	0.0000
205808_at	aspartate beta-hydroxylase	ASPH	0.22	0.0000
239913_at	solute carrier family 10 (sodium/bile acid cotransporter family), member 4	SLC10A4	0.23	0.0000
222343_at	BCL2-like 11 (apoptosis facilitator)	BCL2L11	0.31	0.0000
219613_s_at	sirtuin 6	SIRT6	0.48	0.0000
222705_s_at	solute carrier family 25 (mitochondrial carrier; ornithine transporter) member 15	SLC25A15	0.21	0.0000
227161_at	nucleolar protein with MIF4G domain 1	NOM1	0.46	0.0000
214816_x_at	chromosome 19 open reading frame 40	C19orf40	0.45	0.0000
1553111_a_at	kelch repeat and BTB (POZ) domain containing 6	KBTBD6	0.38	0.0000
207388_s_at	prostaglandin E synthase	PTGES	0.48	0.0000
214711_at	glutamyl-tRNA(Gln) amidotransferase, subunit C homolog (bacterial)	GATC	0.36	0.0000
214240_at	galanin prepropeptide	GAL	0.34	0.0000
1555136_at	FYVE, RhoGEF and PH domain containing 6	FGD6	0.48	0.0000
207446_at	toll-like receptor 6	TLR6	0.28	0.0000

Probeset ID	Gene title	Gene symbol	Fold change	FDR (%)
235907_at	transmembrane protein 33	TMEM33	0.39	0.0000
202818_s_at	transcription elongation factor B (SIII), polypeptide 3 (110kDa, elongin A)	TCEB3	0.50	0.0000
222765_x_at	ESF1, nucleolar pre-rRNA processing protein, homolog (S. cerevisiae)	ESF1	0.48	0.0000
220473_s_at	zinc finger, CCHC domain containing 4	ZCCHC4	0.43	0.0000
224846_at	SH3KBP1 binding protein 1	SHKBP1	0.50	0.0000
210380_s_at	calcium channel, voltage-dependent, T type, alpha 1G subunit	CACNA1G	0.31	0.0000
229534_at	acyl-CoA thioesterase 4	ACOT4	0.37	0.0000
210007_s_at	glycerol-3-phosphate dehydrogenase 2 (mitochondrial)	GPD2	0.28	0.0000
222931_s_at	threonine synthase-like 1 (S. cerevisiae)	THNSL1	0.40	0.0000
218688_at	dihydroxyacetone kinase 2 homolog (S. cerevisiae)	DAK	0.47	0.0000
236223_s_at	Ras-like without CAAX 1	RIT1	0.47	0.0000
217094_s_at	itchy E3 ubiquitin protein ligase	ITCH	0.47	0.0000
228941_at	asparagine-linked glycosylation 10, alpha-1,2-glucosyltransferase homolog B (yeast)	ALG10B	0.38	0.0000
213370_s_at	Scm-like with four mbt domains 1	SFMBT1	0.50	0.0000
231894_at	seryl-tRNA synthetase	SARS	0.33	0.0000
212025_s_at	flightless I homolog (Drosophila)	FLII	0.42	0.0000
211795_s_at	FYN binding protein	FYB	0.34	0.0000
242900_at	asparagine-linked glycosylation 10, alpha-1,2-glucosyltransferase homolog B (yeast)	ALG10B	0.46	0.0000
1554119_at	chromosome 16 open reading frame 57	C16orf57	0.34	0.0000
213632_at	dihydroorotate dehydrogenase (quinone)	DHODH	0.41	0.0000
211091_s_at	neurofibromin 2 (merlin)	NF2	0.30	0.0000
210112_at	Hermansky-Pudlak syndrome 1	HPS1	0.39	0.0000
219551_at	ELL associated factor 2	EAF2	0.46	0.0000
47069_at	proline rich 5 (renal)	PRR5	0.46	0.0000
203919_at	transcription elongation factor A (SII), 2	TCEA2	0.50	0.0000
221189_s_at	threonyl-tRNA synthetase 2, mitochondrial (putative)	TARS2	0.49	0.0074
226762_at	purine-rich element binding protein B	PURB	0.43	0.0074
203890_s_at	death-associated protein kinase 3	DAPK3	0.47	0.0074
234932_s_at	CUB domain containing protein 1	CDCP1	0.31	0.0074
1568627_at	SMEK homolog 2, suppressor of mek1 (Dictyostelium)	SMEK2	0.48	0.0074
220198_s_at	eukaryotic translation initiation factor 5A2	EIF5A2	0.42	0.0074
227700_x_at	ATPase family, AAA domain containing 3A	ATAD3A	0.46	0.0074
235498_at	leucine-rich repeats and IQ motif containing 3	LRRIQ3	0.42	0.0074
225807_at	ajuba LIM protein	AJUBA	0.35	0.0074
221530_s_at	basic helix-loop-helix family, member e41	BHLHE41	0.43	0.0074
222558_at	regulation of nuclear pre-mRNA domain containing 1A	RPRD1A	0.48	0.0074
225142_at	jumonji C domain containing histone demethylase 1 homolog D (S. cerevisiae)	JHDM1D	0.49	0.0074
201284_s_at	N-acylaminoacyl-peptide hydrolase	APEH	0.45	0.0074
220266_s_at	Kruppel-like factor 4 (gut)	KLF4	0.45	0.0074
227215_at	golgi-associated PDZ and coiled-coil motif containing	GOPC	0.49	0.0074
235707_at	chromosome 6 open reading frame 228	C6orf228	0.40	0.0074
216598_s_at	chemokine (C-C motif) ligand 2	CCL2	0.47	0.0074
1564166_s_at	LOC100289561-PRKRIP1 readthrough	LOC100630923	0.25	0.0074
225571_at	leukemia inhibitory factor receptor alpha	LIFR	0.27	0.0074
1569906_s_at	PHD finger protein 20	PHF20	0.49	0.0074
230747_s_at	tetratricopeptide repeat domain 39C	TTC39C	0.46	0.0074
205993_s_at	T-box 2	TBX2	0.49	0.0074
235030_at	neurexophilin and PC-esterase domain family, member 3	NXPE3	0.23	0.0074
205085_at	origin recognition complex, subunit 1	ORC1	0.37	0.0074
230560_at	syntaxin binding protein 6 (amisyn)	STXBP6	0.47	0.0074
205822_s_at	3-hydroxy-3-methylglutaryl-CoA synthase 1 (soluble)	HMGCS1	0.43	0.0074
235783_at	mRNA turnover 4 homolog (S. cerevisiae)	MRTO4	0.37	0.0074
242251_at	spermatogenesis associated 5	SPATA5	0.24	0.0074

Probeset ID	Gene title	Gene symbol	Fold change	FDR (%)
214820_at	bromodomain and WD repeat domain containing 1	BRWD1	0.46	0.0074
229538_s_at	IQ motif containing GTPase activating protein 3	IQGAP3	0.50	0.0074
235003_at	U2AF homology motif (UHM) kinase 1	UHMK1	0.33	0.0074
206359_at	suppressor of cytokine signaling 3	SOCS3	0.37	0.0074
242639_at	NMDA receptor regulated 2	NARG2	0.43	0.0074
204991_s_at	neurofibromin 2 (merlin)	NF2	0.50	0.0074
214073_at	cortactin	CTTN	0.41	0.0074
213844_at	homeobox A5	HOXA5	0.49	0.0074
210612_s_at	synaptojanin 2	SYNJ2	0.42	0.0074
217620_s_at	phosphoinositide-3-kinase, catalytic, beta polypeptide	PIK3CB	0.48	0.0074
220283_at	HHIP-like 2	HHIPL2	0.47	0.0074
226767_s_at	fumarylacetoacetate hydrolase domain containing 1	FAHD1	0.46	0.0074
1553338_at	SDE2 telomere maintenance homolog (S. pombe)	SDE2	0.44	0.0074
232263_at	solute carrier family 6 (neutral amino acid transporter), member 15	SLC6A15	0.31	0.0074
216226_at	TAF4b RNA polymerase II, TATA box binding protein (TBP)-associated factor, 105kDa	TAF4B	0.46	0.0164
204525_at	PHD finger protein 14	PHF14	0.43	0.0164
227247_at	pleckstrin homology domain containing, family A (phosphoinositide binding specific) member 8	PLEKHA8	0.41	0.0164
1552790_a_at	SEC62 homolog (S. cerevisiae)	SEC62	0.31	0.0164
205873_at	phosphatidylinositol glycan anchor biosynthesis, class L	PIGL	0.48	0.0164
217124_at	IQ motif containing E	IQCE	0.38	0.0164
203879_at	phosphoinositide-3-kinase, catalytic, delta polypeptide	PIK3CD	0.38	0.0164
234347_s_at	density-regulated protein	DENR	0.45	0.0164
1570153_at	coiled-coil domain containing 169	CCDC169	0.44	0.0164
219232_s_at	egl nine homolog 3 (C. elegans)	EGLN3	0.50	0.0164
208216_at	distal-less homeobox 4	DLX4	0.43	0.0164
228287_at	inhibitor of growth family, member 5	ING5	0.50	0.0164
226487_at	family with sequence similarity 222, member A	FAM222A	0.49	0.0164
241393_at	intracisternal A particle-promoted polypeptide	IPP	0.48	0.0480
225328_at	F-box protein 32	FBXO32	0.37	0.0480
229309_at	adrenoceptor beta 1	ADRB1	0.48	0.0480
222019_at	prefoldin subunit 6	PFDN6	0.50	0.0480
216962_at	RPA interacting protein	RPAIN	0.45	0.0480
233903_s_at	Rho guanine nucleotide exchange factor (GEF) 26	ARHGEF26	0.49	0.0480
1553292_s_at	uncharacterized serine/threonine-protein kinase SgK494	SGK494	0.39	0.0480
229090_at	ZEB1 antisense RNA 1 (non-protein coding)	ZEB1-AS1	0.39	0.0480
237367_x_at	CASP8 and FADD-like apoptosis regulator	CFLAR	0.36	0.1147
230427_s_at	BCL2-associated athanogene 5	BAG5	0.41	0.1147

Table S2. The common differentially expressed genes between normal and tumor tissues in the TWN and ESP cohorts

Probeset ID	Gene title	Gene symbol	ESP (GSE18842) Fold change	ESP (GSE18842) Adjusted P	TWN (GSE19804) Fold change	TWN (GSE19804) Adjusted P
1552309_a_at	nexilin (F actin binding protein)	NEXN	0.19	3.70E-13	0.26	1.81E-09
1552398_a_at	C-type lectin domain family 12, member A	CLEC12A	0.11	6.49E-18	0.16	1.14E-11
1552797_s_at	prominin 2	PROM2	7.17	1.22E-10	17.03	1.38E-21
1552921_a_at	fidgetin-like 1	FIGNL1	5.32	7.68E-18	2.07	2.10E-06
1553043_a_at	CD300 molecule-like family member f	CD300LF	0.11	2.17E-14	0.38	5.70E-07
1553102_a_at	coiled-coil domain containing 69	CCDC69	0.20	7.64E-16	0.34	5.84E-11
1553105_s_at	desmoglein 2	DSG2	9.98	9.73E-18	2.45	3.53E-05
1553171_x_at	leucine rich repeat neuronal 4	LRRN4	0.17	4.28E-08	0.41	6.81E-04
1553185_at	RAS and EF-hand domain containing	RASEF	5.58	1.75E-11	2.93	4.83E-11
1553186_x_at	RAS and EF-hand domain containing	RASEF	5.75	1.55E-11	2.88	4.77E-10
1553297_a_at	colony stimulating factor 3 receptor (granulocyte)	CSF3R	0.21	2.71E-11	0.42	5.23E-09
1553681_a_at	perforin 1 (pore forming protein)	PRF1	0.45	1.14E-06	0.25	2.17E-11
1553928_at	ELMO/CED-12 domain containing 2	ELMOD2	2.05	4.35E-10	2.05	1.27E-08
1554008_at	oncostatin M receptor	OSMR	4.68	8.15E-12	2.31	1.78E-07
1554079_at	UDP-N-acetyl-alpha-D-galactosamine:polypeptide N-acetylgalactosaminyltransferase-like 4	GALNTL4	0.41	2.74E-08	0.31	1.81E-13
1554240_a_at	integrin, alpha L (antigen CD11A (p180), lymphocyte function-associated antigen 1; alpha polypeptide)	ITGAL	0.24	3.14E-11	0.35	8.51E-09
1554271_a_at	centromere protein L	CENPL	5.69	3.01E-17	2.42	2.74E-08
1554452_a_at	hypoxia inducible lipid droplet-associated	HILPDA	5.06	2.28E-12	2.08	7.21E-06
1554503_a_at	osteoclast associated, immunoglobulin-like receptor	OSCAR	0.19	9.91E-17	0.38	8.99E-11
1554533_at	complement component 2	C2	0.27	4.25E-13	0.36	8.93E-10
1554547_at	family with sequence similarity 13, member C	FAM13C	0.17	3.56E-16	0.21	1.76E-14
1554648_a_at	dual oxidase maturation factor 1	DUOXA1	0.26	5.21E-13	0.36	9.21E-11
1554679_a_at	lysosomal protein transmembrane 4 beta	LAPTM4B	5.10	6.52E-13	2.56	3.99E-05
1554690_a_at	transforming, acidic coiled-coil containing protein 1	TACC1	0.37	4.31E-08	0.27	8.18E-16
1554696_s_at	thymidylate synthetase	TYMS	11.71	1.50E-19	3.45	6.66E-10
1554899_s_at	Fc fragment of IgE, high affinity I, receptor for; gamma polypeptide	FCER1G	0.26	7.56E-11	0.44	9.01E-06
1554921_a_at	sciellin	SCEL	0.19	9.62E-10	0.22	4.78E-09
1554930_a_at	fucosyltransferase 8 (alpha (1,6) fucosyltransferase)	FUT8	2.07	2.20E-08	2.73	6.66E-09
1554997_a_at	prostaglandin-endoperoxide synthase 2 (prostaglandin G/H synthase and cyclooxygenase)	PTGS2	0.23	1.51E-05	0.14	3.11E-09
1555137_a_at	FYVE, RhoGEF and PH domain containing 6	FGD6	2.90	1.80E-08	2.00	4.73E-06
1555270_a_at	Wolfram syndrome 1 (wolframin)	WFS1	0.32	5.19E-12	0.27	3.69E-16
1555274_a_at	ethanolaminephosphotransferase 1 (CDP-ethanolamine-specific)	EPT1	8.50	1.69E-18	2.13	1.47E-07
1555310_a_at	p21 protein (Cdc42/Rac)-activated kinase 6	PAK6	4.39	4.39E-12	4.16	5.29E-15
1555411_a_at	cyclin L1	CCNL1	0.46	3.52E-10	0.42	4.11E-09
1555497_a_at	cytochrome P450, family 4, subfamily B, polypeptide 1	CYP4B1	0.02	9.98E-18	0.16	1.23E-07
1555579_s_at	protein tyrosine phosphatase, receptor type, M	PTPRM	0.18	1.48E-16	0.31	1.08E-15
1555638_a_at	SAM domain, SH3 domain and nuclear localization signals 1	SAMSN1	0.41	1.61E-06	0.47	4.46E-05
1555728_a_at	membrane-spanning 4-domains, subfamily A, member 4A	MS4A4A	0.28	7.51E-09	0.44	2.92E-04
1555758_a_at	cyclin-dependent kinase inhibitor 3	CDKN3	21.30	1.10E-20	5.22	2.18E-09
1555759_a_at	chemokine (C-C motif) ligand 5	CCL5	0.42	3.29E-05	0.42	1.37E-05
1555800_at	zinc finger protein 385B	ZNF385B	0.06	1.00E-16	0.19	7.95E-07

Probeset ID	Gene title	Gene symbol	ESP (GSE18842) Fold change	ESP (GSE18842) Adjusted P	TWN (GSE19804) Fold change	TWN (GSE19804) Adjusted P
1555801_s_at	zinc finger protein 385B	ZNF385B	0.05	1.66E-16	0.19	7.17E-07
1555832_s_at	Kruppel-like factor 6	KLF6	0.24	2.54E-17	0.32	1.27E-15
1556037_s_at	hedgehog interacting protein	HHIP	0.06	7.30E-17	0.11	2.59E-13
1556325_at	filamin A interacting protein 1	FILIP1	0.12	4.29E-15	0.11	6.87E-16
1556579_s_at	immunoglobulin superfamily, member 10	IGSF10	0.10	7.46E-18	0.05	3.23E-21
1557938_s_at	polymerase I and transcript release factor	PTRF	0.45	1.27E-07	0.28	3.64E-12
1557961_s_at	uncharacterized LOC100127983	LOC100127983	0.41	1.39E-07	0.31	1.00E-13
1558034_s_at	ceruloplasmin (ferroxidase)	CP	3.54	1.55E-04	8.51	1.76E-11
1558397_at	platelet/endothelial cell adhesion molecule 1	PECAM1	0.17	1.83E-16	0.16	7.40E-19
1559883_s_at	SAM domain and HD domain 1	SAMHD1	0.48	7.35E-04	0.46	6.52E-05
1560359_at	Pelota homolog (Drosophila)	PELO	0.22	1.54E-14	0.17	1.36E-14
1560396_at	kelch-like 6 (Drosophila)	KLHL6	0.22	3.99E-11	0.44	9.84E-05
1560562_a_at	zinc finger protein 677	ZNF677	0.32	5.14E-08	0.48	1.99E-08
1560916_a_at	dpy-19-like 1 (C. elegans)	DPY19L1	2.92	5.02E-10	3.25	7.64E-12
1564796_at	epithelial membrane protein 1	EMP1	0.14	2.51E-11	0.16	1.78E-12
1568678_s_at	FGFR1 oncogene partner	FGFR1OP	2.71	4.02E-14	2.07	8.29E-11
1569110_x_at	programmed cell death 6 pseudogene	LOC728613	2.63	1.65E-09	2.18	1.43E-10
1598_g_at	growth arrest-specific 6	GAS6	0.19	2.97E-16	0.35	5.75E-11
160020_at	matrix metallopeptidase 14 (membrane-inserted)	MMP14	3.56	8.35E-15	2.23	8.76E-08
200606_at	desmoplakin	DSP	25.77	1.07E-18	2.31	2.27E-05
200644_at	MARCKS-like 1	MARCKSL1	2.53	2.26E-11	2.18	1.35E-08
200700_s_at	KDEL (Lys-Asp-Glu-Leu) endoplasmic reticulum protein retention receptor 2	KDELR2	3.08	1.48E-18	2.56	5.47E-15
200762_at	dihydropyrimidinase-like 2	DPYSL2	0.21	1.06E-17	0.43	3.64E-12
200795_at	SPARC-like 1 (hevin)	SPARCL1	0.26	1.32E-12	0.35	6.44E-12
200904_at	major histocompatibility complex, class I, E	HLA-E	0.30	1.49E-11	0.48	9.20E-14
200911_s_at	transforming, acidic coiled-coil containing protein 1	TACC1	0.34	9.75E-12	0.37	1.23E-17
200928_s_at	RAB14, member RAS oncogene family	RAB14	0.44	1.53E-12	0.49	1.42E-09
200986_at	serpin peptidase inhibitor, clade G (C1 inhibitor), member 1	SERPING1	0.33	4.56E-12	0.41	4.04E-11
201013_s_at	phosphoribosylaminoimidazole carboxylase, phosphoribosylaminoimidazole succinocarboxamide synthetase	PAICS	5.22	1.32E-20	2.65	3.07E-15
201014_s_at	phosphoribosylaminoimidazole carboxylase, phosphoribosylaminoimidazole succinocarboxamide synthetase	PAICS	7.03	4.81E-20	2.66	8.56E-13
201041_s_at	dual specificity phosphatase 1	DUSP1	0.20	1.02E-18	0.39	4.03E-10
201042_at	transglutaminase 2 (C polypeptide, protein-glutamine-gamma-glutamyltransferase)	TGM2	0.27	3.85E-08	0.43	4.90E-10
201044_x_at	dual specificity phosphatase 1	DUSP1	0.25	1.92E-10	0.32	5.12E-08
201058_s_at	myosin, light chain 9, regulatory	MYL9	0.31	1.52E-09	0.34	8.13E-12
201088_at	karyopherin alpha 2 (RAG cohort 1, importin alpha 1)	KPNA2	3.31	1.86E-18	2.02	2.95E-11
201131_s_at	cadherin 1, type 1, E-cadherin (epithelial)	CDH1	2.45	1.33E-07	2.31	7.88E-14
201147_s_at	TIMP metallopeptidase inhibitor 3	TIMP3	0.46	1.27E-05	0.37	4.88E-10
201148_s_at	TIMP metallopeptidase inhibitor 3	TIMP3	0.31	1.92E-09	0.31	5.58E-13
201150_s_at	TIMP metallopeptidase inhibitor 3	TIMP3	0.27	1.14E-10	0.36	3.76E-12
201195_s_at	solute carrier family 7 (amino acid transporter light chain, L system), member 5	SLC7A5	7.43	1.99E-15	2.59	6.62E-07
201231_s_at	enolase 1, (alpha)	ENO1	2.76	1.22E-20	2.17	2.86E-13
201236_s_at	BTG family, member 2	BTG2	0.27	2.88E-13	0.41	1.51E-11
201250_s_at	solute carrier family 2 (facilitated glucose transporter), member 1	SLC2A1	54.91	4.68E-21	4.56	6.80E-10
201286_at	syndecan 1	SDC1	2.93	9.46E-10	2.18	8.77E-08

Probeset ID	Gene title	Gene symbol	ESP (GSE18842)		TWN (GSE19804)	
			Fold change	Adjusted P	Fold change	Adjusted P
201287_s_at	syndecan 1	SDC1	4.25	3.07E-14	2.44	4.45E-12
201289_at	cysteine-rich, angiogenic inducer, 61	CYR61	0.36	6.29E-08	0.37	1.36E-09
201291_s_at	topoisomerase (DNA) II alpha 170kDa	TOP2A	66.71	2.39E-25	11.95	2.47E-14
201292_at	topoisomerase (DNA) II alpha 170kDa	TOP2A	49.84	7.90E-25	13.97	7.19E-17
201328_at	v-ets erythroblastosis virus E26 oncogene homolog 2 (avian)	ETS2	0.34	8.54E-11	0.44	1.76E-09
201348_at	glutathione peroxidase 3 (plasma)	GPX3	0.09	6.35E-18	0.27	2.86E-13
201387_s_at	ubiquitin carboxyl-terminal esterase L1 (ubiquitin thiolesterase)	UCHL1	20.14	9.63E-13	2.51	2.51E-06
201416_at	SRY (sex determining region Y)-box 4	SOX4	2.96	4.24E-13	2.58	2.08E-13
201417_at	SRY (sex determining region Y)-box 4	SOX4	2.83	6.87E-13	2.25	1.06E-11
201418_s_at	SRY (sex determining region Y)-box 4	SOX4	2.79	2.95E-12	2.40	1.46E-09
201432_at	catalase	CAT	0.25	4.28E-20	0.45	6.60E-10
201466_s_at	jun proto-oncogene	JUN	0.38	8.94E-11	0.45	1.26E-10
201467_s_at	NAD(P)H dehydrogenase, quinone 1	NQO1	7.64	5.11E-12	5.05	3.52E-12
201468_s_at	NAD(P)H dehydrogenase, quinone 1	NQO1	3.22	3.20E-06	4.11	7.55E-12
201473_at	jun B proto-oncogene	JUNB	0.31	2.84E-12	0.39	8.41E-12
201496_x_at	myosin, heavy chain 11, smooth muscle	MYH11	0.21	4.85E-09	0.30	1.38E-08
201497_x_at	myosin, heavy chain 11, smooth muscle	MYH11	0.17	7.93E-10	0.38	3.68E-08
201531_at	zinc finger protein 36, C3H type, homolog (mouse)	ZFP36	0.16	4.13E-17	0.29	1.37E-12
201548_s_at	lysine (K)-specific demethylase 5B	KDM5B	3.80	1.65E-21	2.10	6.22E-11
201563_at	sorbitol dehydrogenase	SORD	4.66	1.55E-14	3.05	1.10E-18
201566_x_at	inhibitor of DNA binding 2, dominant negative helix-loop-helix protein	ID2	0.29	1.28E-17	0.49	2.87E-10
201578_at	podocalyxin-like	PODXL	0.33	4.13E-11	0.38	5.65E-16
201579_at	FAT tumor suppressor homolog 1 (Drosophila)	FAT1	3.49	6.00E-13	2.23	2.00E-10
201609_x_at	isoprenylcysteine carboxyl methyltransferase	ICMT	2.01	3.86E-13	2.11	1.71E-12
201645_at	tenascin C	TNC	4.58	8.68E-10	2.63	3.05E-06
201693_s_at	early growth response 1	EGR1	0.18	1.72E-12	0.24	5.65E-11
201694_s_at	early growth response 1	EGR1	0.33	1.45E-10	0.38	3.70E-12
201709_s_at	nipsnap homolog 1 (C. elegans)	NIPSNAP1	3.39	8.36E-17	2.16	2.43E-14
201719_s_at	erythrocyte membrane protein band 4.1-like 2	EPB41L2	0.49	4.03E-09	0.42	1.32E-11
201797_s_at	valyl-tRNA synthetase	VARS	2.84	1.21E-11	2.27	1.64E-12
201809_s_at	endoglin	ENG	0.30	5.01E-13	0.45	2.11E-12
201810_s_at	SH3-domain binding protein 5 (BTK-associated)	SH3BP5	0.26	1.08E-14	0.32	5.63E-14
201811_x_at	SH3-domain binding protein 5 (BTK-associated)	SH3BP5	0.29	2.05E-15	0.41	5.60E-15
201830_s_at	neuroepithelial cell transforming 1	NET1	3.50	3.34E-12	2.05	4.87E-08
201842_s_at	EGF containing fibulin-like extracellular matrix protein 1	EFEMP1	0.30	2.70E-11	0.32	1.56E-10
201843_s_at	EGF containing fibulin-like extracellular matrix protein 1	EFEMP1	0.18	5.51E-13	0.25	8.94E-12
201852_x_at	collagen, type III, alpha 1	COL3A1	7.06	6.13E-14	4.11	3.22E-12
201858_s_at	serglycin	SRGN	0.35	6.71E-09	0.35	6.67E-06
201884_at	carcinoembryonic antigen-related cell adhesion molecule 5	CEACAM5	10.91	7.02E-09	11.27	3.65E-08
201890_at	ribonucleotide reductase M2	RRM2	26.56	3.93E-22	6.12	3.11E-12
201897_s_at	CDC28 protein kinase regulatory subunit 1B	CKS1B	4.24	1.15E-18	2.05	1.42E-09
201925_s_at	CD55 molecule, decay accelerating factor for complement (Cromer blood group)	CD55	0.20	2.03E-13	0.44	3.70E-10
201983_s_at	epidermal growth factor receptor	EGFR	2.20	1.17E-07	2.18	1.01E-06
201984_s_at	epidermal growth factor receptor	EGFR	2.97	2.50E-10	2.78	9.30E-08
202005_at	suppression of tumorigenicity 14 (colon carcinoma)	ST14	2.53	2.29E-11	2.91	8.21E-18
202014_at	protein phosphatase 1, regulatory subunit 15A	PPP1R15A	0.19	1.91E-15	0.19	8.53E-18
202067_s_at	low density lipoprotein receptor	LDLR	0.44	2.41E-06	0.26	1.06E-10
202068_s_at	low density lipoprotein receptor	LDLR	0.39	3.58E-08	0.26	7.30E-12

Probeset ID	Gene title	Gene symbol	ESP (GSE18842) Fold change	ESP (GSE18842) Adjusted P	TWN (GSE19804) Fold change	TWN (GSE19804) Adjusted P
202073_at	optineurin	OPTN	0.44	4.62E-09	0.47	1.54E-10
202081_at	immediate early response 2	IER2	0.39	1.24E-11	0.47	4.31E-11
202085_at	tight junction protein 2 (zona occludens 2)	TJP2	0.41	9.39E-07	0.42	1.30E-13
202107_s_at	minichromosome maintenance complex component 2	MCM2	12.47	2.43E-21	3.36	2.75E-12
202112_at	von Willebrand factor	VWF	0.20	3.50E-12	0.21	3.53E-16
202148_s_at	pyrroline-5-carboxylate reductase 1	PYCR1	5.65	2.03E-18	4.26	1.64E-20
202149_at	neural precursor cell expressed, developmentally down-regulated 9	NEDD9	0.21	7.30E-17	0.34	3.05E-11
202150_s_at	neural precursor cell expressed, developmentally down-regulated 9	NEDD9	0.31	3.45E-12	0.29	9.07E-13
202156_s_at	CUGBP, Elav-like family member 2	CELF2	0.21	7.35E-13	0.47	1.18E-07
202157_s_at	CUGBP, Elav-like family member 2	CELF2	0.27	9.69E-12	0.49	1.56E-07
202177_at	growth arrest-specific 6	GAS6	0.19	6.54E-16	0.38	6.75E-11
202183_s_at	kinesin family member 22	KIF22	2.08	1.61E-06	2.08	9.69E-08
202200_s_at	SRSF protein kinase 1	SRPK1	2.41	1.24E-14	2.16	5.94E-16
202202_s_at	laminin, alpha 4	LAMA4	0.49	5.53E-07	0.39	1.77E-11
202274_at	actin, gamma 2, smooth muscle, enteric	ACTG2	0.48	2.81E-04	0.44	1.92E-06
202341_s_at	tripartite motif containing 2	TRIM2	3.37	1.82E-12	3.86	4.98E-16
202342_s_at	tripartite motif containing 2	TRIM2	2.87	3.70E-13	3.08	3.34E-14
202357_s_at	complement factor B	CFB	2.15	2.62E-03	3.01	5.42E-08
202388_at	regulator of G-protein signaling 2, 24kDa	RGS2	0.26	3.49E-13	0.30	3.76E-13
202404_s_at	collagen, type I, alpha 2	COL1A2	7.60	3.72E-16	4.33	1.59E-14
202468_s_at	catenin (cadherin-associated protein), alpha-like 1	CTNNAL1	0.35	2.46E-11	0.35	2.18E-15
202487_s_at	H2A histone family, member V	H2AFV	2.87	8.84E-20	2.11	6.24E-13
202497_x_at	solute carrier family 2 (facilitated glucose transporter), member 3	SLC2A3	0.41	1.12E-06	0.28	1.08E-12
202498_s_at	solute carrier family 2 (facilitated glucose transporter), member 3	SLC2A3	0.43	2.02E-05	0.24	2.48E-13
202499_s_at	solute carrier family 2 (facilitated glucose transporter), member 3	SLC2A3	0.33	3.67E-08	0.30	1.01E-11
202503_s_at	KIAA0101	KIAA0101	20.95	1.67E-20	7.65	2.10E-17
202524_s_at	sparc/osteonectin, cwcv and kazal-like domains proteoglycan (testican) 2	SPOCK2	0.10	9.14E-16	0.08	2.63E-21
202531_at	interferon regulatory factor 1	IRF1	0.32	1.79E-08	0.38	6.17E-11
202551_s_at	cysteine rich transmembrane BMP regulator 1 (chordin-like)	CRIM1	0.34	6.64E-13	0.34	3.38E-15
202552_s_at	cysteine rich transmembrane BMP regulator 1 (chordin-like)	CRIM1	0.33	8.44E-15	0.40	5.60E-15
202589_at	thymidylate synthetase	TYMS	7.98	2.46E-17	4.19	2.58E-11
202619_s_at	procollagen-lysine, 2-oxoglutarate 5-dioxygenase 2	PLOD2	7.17	2.51E-19	2.37	8.60E-07
202620_s_at	procollagen-lysine, 2-oxoglutarate 5-dioxygenase 2	PLOD2	6.14	1.00E-18	2.48	4.93E-09
202637_s_at	intercellular adhesion molecule 1	ICAM1	0.22	2.71E-09	0.38	1.11E-07
202638_s_at	intercellular adhesion molecule 1	ICAM1	0.16	5.86E-10	0.32	5.11E-08
202643_s_at	tumor necrosis factor, alpha-induced protein 3	TNFAIP3	0.44	6.60E-05	0.27	1.49E-10
202644_s_at	tumor necrosis factor, alpha-induced protein 3	TNFAIP3	0.41	3.86E-06	0.37	1.11E-10
202672_s_at	activating transcription factor 3	ATF3	0.23	2.73E-10	0.18	9.11E-12
202674_s_at	LIM domain 7	LMO7	0.10	1.44E-19	0.15	1.38E-13
202705_at	cyclin B2	CCNB2	29.20	1.92E-21	6.70	4.27E-14
202718_at	insulin-like growth factor binding protein 2, 36kDa	IGFBP2	8.65	3.35E-12	2.11	2.59E-04
202721_s_at	glutamine--fructose-6-phosphate transaminase 1	GFPT1	3.66	4.00E-14	2.65	1.15E-09
202722_s_at	glutamine--fructose-6-phosphate transaminase 1	GFPT1	2.57	1.56E-13	2.61	2.05E-13
202732_at	protein kinase (cAMP-dependent, catalytic) inhibitor gamma	PKIG	0.27	4.67E-13	0.30	1.10E-14
202768_at	FBJ murine osteosarcoma viral oncogene	FOSB	0.04	3.86E-14	0.07	6.23E-12

Probeset ID	Gene title	Gene symbol	ESP (GSE18842) Fold change	ESP (GSE18842) Adjusted P	TWN (GSE19804) Fold change	TWN (GSE19804) Adjusted P
	homolog B					
202794_at	inositol polyphosphate-1-phosphatase	INPP1	0.48	3.41E-07	0.47	9.35E-12
202796_at	synaptopodin	SYNPO	0.30	1.19E-13	0.43	3.17E-10
202861_at	period homolog 1 (Drosophila)	PER1	0.35	5.05E-09	0.39	5.45E-09
202877_s_at	CD93 molecule	CD93	0.22	3.88E-12	0.16	1.22E-14
202878_s_at	CD93 molecule	CD93	0.21	1.16E-12	0.23	2.89E-16
202897_at	signal-regulatory protein alpha	SIRPA	0.40	8.35E-09	0.45	8.68E-08
202908_at	Wolfram syndrome 1 (wolframin)	WFS1	0.30	8.23E-13	0.33	1.95E-14
202910_s_at	CD97 molecule	CD97	0.19	7.35E-15	0.37	1.37E-14
202917_s_at	S100 calcium binding protein A8	S100A8	0.45	4.15E-03	0.18	3.74E-10
202920_at	ankyrin 2, neuronal	ANK2	0.13	1.80E-13	0.24	3.70E-10
202947_s_at	glycophorin C (Gerbich blood group)	GYPC	0.22	3.14E-16	0.37	3.81E-15
202949_s_at	four and a half LIM domains 2	FHL2	2.77	9.57E-09	3.02	3.73E-10
202953_at	complement component 1, q subcomponent, B chain	C1QB	0.21	3.51E-12	0.49	2.18E-05
202954_at	ubiquitin-conjugating enzyme E2C	UBE2C	31.73	5.03E-22	6.00	2.55E-14
202992_at	complement component 7	C7	0.05	1.14E-14	0.19	1.36E-09
202994_s_at	fibulin 1	FBLN1	0.35	5.22E-09	0.35	3.48E-09
202995_s_at	fibulin 1	FBLN1	0.39	7.02E-07	0.30	4.08E-08
203002_at	angiomotin like 2	AMOTL2	0.46	7.99E-09	0.43	1.95E-14
203021_at	secretory leukocyte peptidase inhibitor	SLPI	0.21	4.82E-11	0.36	3.20E-04
203022_at	ribonuclease H2, subunit A	RNASEH2 A	5.45	1.29E-16	2.76	3.24E-11
203029_s_at	protein tyrosine phosphatase, receptor type, N polypeptide 2	PTPRN2	0.19	8.54E-12	0.32	2.83E-09
203035_s_at	protein inhibitor of activated STAT, 3	PIAS3	2.15	1.10E-10	2.15	4.86E-15
203040_s_at	hydroxymethylbilane synthase	HMBS	2.43	5.91E-15	2.43	1.77E-17
203046_s_at	timeless homolog (Drosophila)	TIMELESS	5.70	4.66E-18	2.27	7.20E-11
203058_s_at	3'-phosphoadenosine 5'-phosphosulfate synthase 2	PAPSS2	0.22	2.02E-14	0.29	8.47E-15
203059_s_at	3'-phosphoadenosine 5'-phosphosulfate synthase 2	PAPSS2	0.30	1.01E-12	0.32	2.31E-12
203060_s_at	3'-phosphoadenosine 5'-phosphosulfate synthase 2	PAPSS2	0.24	1.41E-14	0.37	3.81E-15
203063_at	protein phosphatase, Mg2+/Mn2+ dependent, 1F	PPM1F	0.47	3.15E-10	0.32	6.01E-17
203065_s_at	caveolin 1, caveolae protein, 22kDa	CAV1	0.10	8.92E-19	0.10	1.00E-16
203083_at	thrombospondin 2	THBS2	9.93	3.77E-14	6.76	4.32E-15
203088_at	fibulin 5	FBLN5	0.14	1.08E-17	0.28	5.27E-11
203108_at	G protein-coupled receptor, family C, group 5, member A	GPRC5A	0.13	6.12E-13	0.29	8.11E-08
203143_s_at	KIAA0040	KIAA0040	0.27	4.49E-15	0.36	2.78E-14
203145_at	sperm associated antigen 5	SPAG5	13.21	1.80E-21	2.80	1.85E-09
203185_at	Ras association (RalGDS/AF-6) domain family member 2	RASSF2	0.20	8.78E-13	0.32	5.14E-12
203186_s_at	S100 calcium binding protein A4	S100A4	0.19	1.35E-14	0.40	1.98E-12
203213_at	cyclin-dependent kinase 1	CDK1	9.79	1.41E-20	2.93	1.70E-11
203214_x_at	cyclin-dependent kinase 1	CDK1	21.40	7.08E-25	3.45	5.82E-09
203276_at	lamin B1	LMNB1	2.70	4.88E-12	2.45	1.77E-07
203287_at	ladinin 1	LAD1	5.74	4.49E-13	4.82	1.12E-14
203323_at	caveolin 2	CAV2	0.12	1.09E-18	0.14	8.07E-14
203324_s_at	caveolin 2	CAV2	0.26	2.38E-12	0.24	9.67E-12
203325_s_at	collagen, type V, alpha 1	COL5A1	4.04	2.20E-11	2.71	2.10E-10
203329_at	protein tyrosine phosphatase, receptor type, M	PTPRM	0.24	2.09E-15	0.37	6.64E-15
203362_s_at	MAD2 mitotic arrest deficient-like 1 (yeast)	MAD2L1	20.44	1.97E-23	3.92	1.02E-09
203372_s_at	suppressor of cytokine signaling 2	SOCS2	0.20	1.57E-08	0.11	6.34E-14
203373_at	suppressor of cytokine signaling 2	SOCS2	0.19	1.26E-11	0.16	7.01E-16
203397_s_at	UDP-N-acetyl-alpha-D-	GALNT3	2.27	6.81E-06	2.46	5.15E-09

Probeset ID	Gene title	Gene symbol	ESP (GSE18842) Fold change	ESP (GSE18842) Adjusted P	TWN (GSE19804) Fold change	TWN (GSE19804) Adjusted P
	galactosamine:polypeptide N-acetylgalactosaminyltransferase 3 (GalNAc-T3)					
203417_at	microfibrillar-associated protein 2	MFAP2	4.40	1.13E-12	2.83	7.31E-09
203434_s_at	membrane metallo-endopeptidase	MME	0.19	2.26E-08	0.08	3.06E-17
203471_s_at	pleckstrin	PLEK	0.38	2.44E-06	0.46	1.82E-05
203476_at	trophoblast glycoprotein	TPBG	4.90	1.52E-19	3.27	3.43E-16
203485_at	reticulon 1	RTN1	0.22	1.82E-10	0.32	1.55E-09
203517_at	metaxin 2	MTX2	2.23	3.81E-16	2.00	7.86E-16
203542_s_at	Kruppel-like factor 9	KLF9	0.23	4.36E-14	0.27	1.85E-14
203543_s_at	Kruppel-like factor 9	KLF9	0.29	2.06E-12	0.26	1.32E-14
203548_s_at	lipoprotein lipase	LPL	0.05	1.02E-18	0.19	1.40E-11
203549_s_at	lipoprotein lipase	LPL	0.07	3.37E-18	0.21	1.33E-12
203554_x_at	pituitary tumor-transforming 1	PTTG1	9.17	1.00E-19	3.79	3.09E-13
203562_at	fasciculation and elongation protein zeta 1 (zygin I)	FEZ1	0.45	9.05E-06	0.21	1.61E-12
203570_at	lysyl oxidase-like 1	LOXL1	2.95	1.85E-11	2.24	1.28E-08
203571_s_at	chromosome 10 open reading frame 116	C10orf116	0.07	1.57E-17	0.10	3.82E-19
203591_s_at	colony stimulating factor 3 receptor (granulocyte)	CSF3R	0.17	1.71E-11	0.22	2.01E-12
203592_s_at	follistatin-like 3 (secreted glycoprotein)	FSTL3	0.23	9.30E-11	0.28	2.73E-14
203603_s_at	zinc finger E-box binding homeobox 2	ZEB2	0.24	7.84E-12	0.38	2.33E-11
203615_x_at	sulfotransferase family, cytosolic, 1A, phenol-preferring, member 1	SULT1A1	0.23	6.45E-15	0.49	2.12E-13
203661_s_at	tropomodulin 1	TMOD1	0.18	2.01E-16	0.22	1.53E-14
203666_at	chemokine (C-X-C motif) ligand 12	CXCL12	0.40	3.78E-07	0.41	7.45E-07
203680_at	protein kinase, cAMP-dependent, regulatory, type II, beta	PRKAR2B	0.41	6.16E-08	0.41	3.66E-07
203710_at	inositol 1,4,5-trisphosphate receptor, type 1	ITPR1	0.46	2.48E-10	0.46	2.00E-12
203718_at	patatin-like phospholipase domain containing 6	PNPLA6	0.27	2.84E-20	0.44	7.63E-15
203729_at	epithelial membrane protein 3	EMP3	0.33	1.27E-09	0.50	2.37E-09
203751_x_at	jun D proto-oncogene	JUND	0.34	2.93E-10	0.34	5.91E-12
203760_s_at	Src-like-adaptor	SLA	0.24	3.88E-12	0.46	3.05E-08
203764_at	discs, large (Drosophila) homolog-associated protein 5	DLGAP5	41.46	7.38E-26	7.51	7.01E-12
203766_s_at	leiomodin 1 (smooth muscle)	LMOD1	0.21	2.38E-14	0.30	3.66E-10
203775_at	solute carrier family 25 (aspartate/glutamate carrier), member 13	SLC25A13	2.01	9.19E-11	2.09	5.31E-13
203779_s_at	myelin protein zero-like 2	MPZL2	2.03	1.12E-04	2.09	1.22E-04
203810_at	DnaJ (Hsp40) homolog, subfamily B, member 4	DNAJB4	0.42	5.22E-09	0.35	5.51E-13
203813_s_at	slit homolog 3 (Drosophila)	SLIT3	0.36	1.05E-08	0.36	1.98E-12
203820_s_at	insulin-like growth factor 2 mRNA binding protein 3	IGF2BP3	32.18	7.58E-17	7.52	1.05E-08
203821_at	heparin-binding EGF-like growth factor	HBEGF	0.18	1.51E-11	0.15	2.12E-16
203828_s_at	interleukin 32	IL32	0.47	1.94E-04	0.50	3.10E-06
203835_at	leucine rich repeat containing 32	LRRC32	0.15	8.25E-14	0.17	4.16E-15
203845_at	K(lysine) acetyltransferase 2B	KAT2B	0.27	6.34E-16	0.49	6.07E-12
203851_at	insulin-like growth factor binding protein 6	IGFBP6	0.38	1.91E-05	0.42	3.01E-07
203865_s_at	adenosine deaminase, RNA-specific, B1	ADARB1	0.13	1.02E-17	0.19	4.62E-17
203887_s_at	thrombomodulin	THBD	0.25	1.70E-09	0.18	1.96E-17
203888_at	thrombomodulin	THBD	0.26	3.60E-09	0.18	1.17E-16
203895_at	phospholipase C, beta 4	PLCB4	0.36	2.40E-05	0.16	4.72E-11
203910_at	Rho GTPase activating protein 29	ARHGAP29	0.20	3.38E-13	0.32	5.63E-16
203913_s_at	hydroxyprostaglandin dehydrogenase 15-(NAD)	HPGD	0.10	3.58E-15	0.50	1.61E-03
203914_x_at	hydroxyprostaglandin dehydrogenase 15-(NAD)	HPGD	0.06	4.47E-16	0.37	4.38E-06
203934_at	kinase insert domain receptor (a type III receptor tyrosine kinase)	KDR	0.38	2.05E-05	0.32	4.13E-12
203936_s_at	matrix metallopeptidase 9 (gelatinase B, 92kDa	MMP9	5.83	3.22E-10	4.27	5.36E-07

Probeset ID	Gene title	Gene symbol	ESP (GSE18842) Fold change	Adjusted P	TWN (GSE19804) Fold change	Adjusted P
	gelatinase, 92kDa type IV collagenase)					
203946_s_at	arginase, type II	ARG2	4.06	2.13E-07	2.25	3.38E-04
203951_at	calponin 1, basic, smooth muscle	CNN1	0.30	8.36E-09	0.29	1.42E-09
203961_at	nebulette	NEBL	0.18	1.02E-11	0.28	1.93E-09
203962_s_at	nebulette	NEBL	0.32	1.09E-07	0.28	1.71E-08
203968_s_at	cell division cycle 6 homolog (S. cerevisiae)	CDC6	9.65	1.05E-18	2.36	3.29E-07
203973_s_at	CCAAT/enhancer binding protein (C/EBP), delta	CEBPD	0.46	2.78E-11	0.49	1.64E-10
203979_at	cytochrome P450, family 27, subfamily A, polypeptide 1	CYP27A1	0.15	1.08E-16	0.49	1.04E-07
203980_at	fatty acid binding protein 4, adipocyte	FABP4	0.01	5.41E-17	0.03	5.98E-17
204007_at	Fc fragment of IgG, low affinity IIIb, receptor (CD16b)	FCGR3B	0.26	1.77E-10	0.24	1.80E-12
204017_at	KDEL (Lys-Asp-Glu-Leu) endoplasmic reticulum protein retention receptor 3	KDELR3	3.71	1.88E-11	4.66	7.40E-19
204023_at	replication factor C (activator 1) 4, 37kDa	RFC4	8.53	7.42E-19	2.21	3.21E-08
204026_s_at	ZW10 interactor	ZWINT	9.86	2.13E-19	5.22	1.65E-16
204027_s_at	methyltransferase like 1	METTL1	2.16	1.57E-10	2.20	6.33E-11
204033_at	thyroid hormone receptor interactor 13	TRIP13	18.25	2.16E-20	2.64	3.41E-06
204042_at	WAS protein family, member 3	WASF3	0.26	1.32E-10	0.21	4.96E-14
204048_s_at	phosphatase and actin regulator 2	PHACTR2	0.32	1.09E-14	0.43	1.22E-11
204049_s_at	phosphatase and actin regulator 2	PHACTR2	0.28	4.28E-15	0.43	1.14E-10
204063_s_at	unc-51-like kinase 2 (C. elegans)	ULK2	0.40	5.55E-16	0.44	7.96E-13
204069_at	Meis homeobox 1	MEIS1	0.46	1.12E-10	0.39	1.27E-10
204072_s_at	furry homolog (Drosophila)	FRY	0.10	6.04E-21	0.27	4.38E-14
204073_s_at	chromosome 11 open reading frame 9	C11orf9	0.09	1.09E-17	0.14	5.00E-15
204078_at	leprecan-like 4	LEPREL4	7.19	1.67E-16	3.17	3.95E-14
204081_at	neurogranin (protein kinase C substrate, RC3)	NRGN	0.16	7.15E-13	0.43	1.33E-06
204103_at	chemokine (C-C motif) ligand 4	CCL4	0.31	1.40E-06	0.26	1.69E-09
204115_at	guanine nucleotide binding protein (G protein), gamma 11	GNG11	0.13	9.27E-16	0.21	6.80E-17
204122_at	TYRO protein tyrosine kinase binding protein	TYROBP	0.19	4.85E-15	0.49	2.54E-07
204146_at	RAD51 associated protein 1	RAD51AP1	6.68	2.28E-17	2.79	8.89E-09
204153_s_at	MFNG O-fucosylpeptide 3-beta-N-acetylglucosaminyltransferase	MFNG	0.16	1.66E-18	0.32	4.22E-15
204154_at	cysteine dioxygenase, type I	CDO1	0.08	6.76E-18	0.10	9.52E-16
204162_at	NDC80 kinetochore complex component homolog (S. cerevisiae)	NDC80	13.33	4.45E-21	3.33	6.30E-10
204174_at	arachidonate 5-lipoxygenase-activating protein	ALOX5AP	0.17	5.53E-13	0.38	1.11E-06
204192_at	CD37 molecule	CD37	0.19	9.77E-12	0.42	2.44E-06
204204_at	solute carrier family 31 (copper transporters), member 2	SLC31A2	0.24	8.79E-14	0.40	3.21E-08
204220_at	glia maturation factor, gamma	GMFG	0.17	1.25E-16	0.31	1.08E-13
204223_at	proline/arginine-rich end leucine-rich repeat protein	PRELP	0.22	3.07E-10	0.32	6.95E-11
204236_at	Friend leukemia virus integration 1	FLI1	0.24	9.47E-16	0.29	2.08E-12
204249_s_at	LIM domain only 2 (rhombotin-like 1)	LMO2	0.21	6.62E-18	0.31	1.52E-14
204257_at	fatty acid desaturase 3	FADS3	0.39	2.47E-09	0.47	1.08E-11
204268_at	S100 calcium binding protein A2	S100A2	77.60	7.65E-15	6.02	1.95E-08
204271_s_at	endothelin receptor type B	EDNRB	0.08	1.75E-21	0.09	3.81E-20
204273_at	endothelin receptor type B	EDNRB	0.06	5.40E-21	0.08	7.84E-19
204303_s_at	CBP80/20-dependent translation initiation factor	CTIF	0.46	2.58E-15	0.42	9.86E-16
204326_x_at	metallothionein 1X	MT1X	0.37	3.67E-07	0.49	6.49E-08
204343_at	ATP-binding cassette, sub-family A (ABC1), member 3	ABCA3	0.06	2.31E-17	0.20	7.96E-11
204364_s_at	receptor accessory protein 1	REEP1	0.18	8.40E-12	0.20	9.94E-14
204368_at	solute carrier organic anion transporter family, member 2A1	SLCO2A1	0.16	1.91E-13	0.28	3.98E-12

Probeset ID	Gene title	Gene symbol	ESP (GSE18842) Fold change	Adjusted P	TWN (GSE19804) Fold change	Adjusted P
204396_s_at	G protein-coupled receptor kinase 5	GRK5	0.16	6.28E-19	0.12	1.38E-21
204422_s_at	fibroblast growth factor 2 (basic)	FGF2	0.24	6.18E-15	0.14	1.95E-17
204438_at	mannose receptor, C type 1	MRC1	0.15	3.77E-13	0.42	1.54E-06
204440_at	CD83 molecule	CD83	0.19	9.50E-16	0.45	1.14E-09
204445_s_at	arachidonate 5-lipoxygenase	ALOX5	0.18	1.17E-15	0.44	1.82E-08
204446_s_at	arachidonate 5-lipoxygenase	ALOX5	0.09	2.83E-16	0.41	6.87E-10
204468_s_at	tyrosine kinase with immunoglobulin-like and EGF-like domains 1	TIE1	0.15	3.95E-16	0.18	5.19E-17
204469_at	protein tyrosine phosphatase, receptor-type, Z polypeptide 1	PTPRZ1	18.05	7.00E-09	2.44	1.63E-04
204475_at	matrix metallopeptidase 1 (interstitial collagenase)	MMP1	75.00	2.41E-14	21.31	5.42E-11
204482_at	claudin 5	CLDN5	0.15	4.02E-16	0.14	7.40E-19
204491_at	phosphodiesterase 4D, cAMP-specific	PDE4D	0.43	3.87E-11	0.48	2.98E-08
204500_s_at	ATP/GTP binding protein 1	AGTPBP1	0.33	8.15E-16	0.37	7.40E-19
204513_s_at	engulfment and cell motility 1	ELMO1	0.36	1.65E-10	0.34	3.96E-14
204519_s_at	plasmolipin	PLLP	0.14	2.23E-13	0.32	5.32E-08
204563_at	selectin L	SELL	0.26	8.05E-08	0.38	9.28E-06
204570_at	cytochrome c oxidase subunit VIIa polypeptide 1 (muscle)	COX7A1	0.16	7.30E-19	0.26	1.65E-14
204575_s_at	matrix metallopeptidase 19	MMP19	0.17	7.96E-12	0.33	3.17E-10
204580_at	matrix metallopeptidase 12 (macrophage elastase)	MMP12	139.48	7.44E-18	22.32	8.80E-14
204588_s_at	solute carrier family 7 (amino acid transporter light chain, y+L system), member 7	SLC7A7	0.22	1.62E-12	0.45	6.12E-09
204606_at	chemokine (C-C motif) ligand 21	CCL21	0.42	1.75E-04	0.25	4.02E-08
204619_s_at	versican	VCAN	2.90	3.03E-07	2.48	1.52E-08
204620_s_at	versican	VCAN	2.55	4.02E-07	2.07	1.37E-06
204621_s_at	nuclear receptor subfamily 4, group A, member 2	NR4A2	0.12	5.02E-14	0.21	1.71E-11
204622_x_at	nuclear receptor subfamily 4, group A, member 2	NR4A2	0.11	1.77E-14	0.18	3.39E-12
204641_at	NIMA (never in mitosis gene a)-related kinase 2	NEK2	28.56	9.34E-28	9.66	9.74E-14
204642_at	sphingosine-1-phosphate receptor 1	S1PR1	0.18	4.77E-16	0.16	1.40E-18
204661_at	CD52 molecule	CD52	0.09	4.43E-18	0.35	2.90E-09
204675_at	steroid-5-alpha-reductase, alpha polypeptide 1 (3-oxo-5 alpha-steroid delta 4-dehydrogenase alpha 1)	SRD5A1	11.61	2.05E-19	6.87	5.22E-17
204677_at	cadherin 5, type 2 (vascular endothelium)	CDH5	0.13	2.92E-16	0.14	1.60E-18
204678_s_at	potassium channel, subfamily K, member 1	KCNK1	3.50	5.54E-11	2.79	2.46E-06
204679_at	potassium channel, subfamily K, member 1	KCNK1	3.13	7.43E-11	2.52	1.05E-06
204681_s_at	Rap guanine nucleotide exchange factor (GEF) 5	RAPGEF5	0.38	9.72E-10	0.46	6.22E-13
204683_at	intercellular adhesion molecule 2	ICAM2	0.18	1.00E-17	0.26	5.10E-16
204688_at	sarcoglycan, epsilon	SGCE	0.45	4.99E-06	0.36	2.91E-10
204702_s_at	nuclear factor (erythroid-derived 2)-like 3	NFE2L3	3.26	8.17E-11	2.29	9.30E-08
204712_at	WNT inhibitory factor 1	WIF1	0.04	2.51E-12	0.03	1.97E-12
204719_at	ATP-binding cassette, sub-family A (ABC1), member 8	ABCA8	0.03	1.95E-20	0.09	4.96E-13
204730_at	regulating synaptic membrane exocytosis 3	RIMS3	0.35	8.98E-09	0.47	6.09E-06
204731_at	transforming growth factor, beta receptor III	TGFBR3	0.18	5.58E-11	0.14	3.34E-16
204745_x_at	metallothionein 1G	MT1G	0.22	5.18E-10	0.48	1.25E-07
204748_at	prostaglandin-endoperoxide synthase 2 (prostaglandin G/H synthase and cyclooxygenase)	PTGS2	0.26	1.05E-05	0.20	8.46E-10
204753_s_at	hepatic leukemia factor	HLF	0.32	5.07E-06	0.43	1.62E-05
204755_x_at	hepatic leukemia factor	HLF	0.20	2.20E-11	0.38	1.66E-06
204767_s_at	flap structure-specific endonuclease 1	FEN1	5.46	1.53E-22	2.53	4.60E-12
204768_s_at	flap structure-specific endonuclease 1	FEN1	8.11	1.05E-21	2.72	9.56E-10
204773_at	interleukin 11 receptor, alpha	IL11RA	0.31	3.86E-13	0.38	4.37E-13

Probeset ID	Gene title	Gene symbol	ESP (GSE18842) Fold change	Adjusted P	TWN (GSE19804) Fold change	Adjusted P
204777_s_at	mal, T-cell differentiation protein	MAL	0.25	3.32E-08	0.22	8.62E-14
204779_s_at	homeobox B7	HOXB7	3.70	1.24E-08	2.75	1.61E-06
204780_s_at	Fas (TNF receptor superfamily, member 6)	FAS	0.47	7.97E-08	0.46	1.19E-07
204781_s_at	Fas (TNF receptor superfamily, member 6)	FAS	0.38	1.99E-09	0.46	7.27E-11
204790_at	SMAD family member 7	SMAD7	0.44	2.63E-10	0.48	3.66E-10
204796_at	echinoderm microtubule associated protein like 1	EML1	0.36	6.37E-08	0.21	3.57E-14
204797_s_at	echinoderm microtubule associated protein like 1	EML1	0.50	2.06E-04	0.25	5.38E-11
204802_at	Ras-related associated with diabetes	RRAD	0.11	6.86E-11	0.42	1.03E-04
204803_s_at	Ras-related associated with diabetes	RRAD	0.16	9.71E-11	0.49	4.87E-04
204811_s_at	calcium channel, voltage-dependent, alpha 2/delta subunit 2	CACNA2D2	0.04	3.19E-19	0.26	3.97E-07
204822_at	TTK protein kinase	TTK	26.74	9.52E-25	5.69	4.75E-11
204846_at	ceruloplasmin (ferroxidase)	CP	5.63	1.14E-05	9.88	3.22E-10
204875_s_at	GDP-mannose 4,6-dehydratase	GMDS	2.15	3.96E-08	2.58	1.11E-11
204882_at	Rho GTPase activating protein 25	ARHGAP25	0.29	1.95E-12	0.36	4.53E-09
204894_s_at	amine oxidase, copper containing 3 (vascular adhesion protein 1)	AOC3	0.09	7.43E-16	0.17	7.64E-13
204897_at	prostaglandin E receptor 4 (subtype EP4)	PTGER4	0.34	4.14E-11	0.33	3.73E-13
204929_s_at	vesicle-associated membrane protein 5 (myobrevin)	VAMP5	0.31	5.83E-09	0.37	2.14E-10
204931_at	transcription factor 21	TCF21	0.03	5.34E-23	0.08	8.12E-15
204939_s_at	phospholamban	PLN	0.20	2.90E-07	0.49	4.99E-03
204959_at	myeloid cell nuclear differentiation antigen	MNDA	0.15	4.27E-14	0.30	2.04E-10
204975_at	epithelial membrane protein 2	EMP2	0.18	1.71E-15	0.22	7.00E-15
204990_s_at	integrin, beta 4	ITGB4	5.81	6.97E-11	2.21	8.39E-07
205003_at	dedicator of cytokinesis 4	DOCK4	0.19	3.59E-19	0.30	1.68E-15
205013_s_at	adenosine A2a receptor	ADORA2A	0.47	2.45E-10	0.49	3.09E-11
205027_s_at	mitogen-activated protein kinase kinase kinase 8	MAP3K8	0.28	1.59E-09	0.24	8.47E-16
205034_at	cyclin E2	CCNE2	8.08	2.11E-20	3.02	2.37E-09
205048_s_at	phosphoserine phosphatase	PSPH	3.65	9.33E-12	2.74	1.01E-07
205051_s_at	v-kit Hardy-Zuckerman 4 feline sarcoma viral oncogene homolog	KIT	0.23	1.18E-08	0.42	2.77E-06
205079_s_at	multiple PDZ domain protein	MPDZ	0.45	1.01E-07	0.28	2.34E-13
205081_at	cysteine-rich protein 1 (intestinal)	CRIP1	0.12	4.12E-12	0.45	3.43E-07
205083_at	aldehyde oxidase 1	AOX1	0.13	3.17E-16	0.15	6.62E-15
205111_s_at	phospholipase C, epsilon 1	PLCE1	0.34	6.57E-12	0.37	7.57E-12
205112_at	phospholipase C, epsilon 1	PLCE1	0.36	7.70E-11	0.31	4.17E-13
205119_s_at	formyl peptide receptor 1	FPR1	0.19	2.25E-11	0.23	2.42E-13
205129_at	nucleophosmin/nucleoplasmin 3	NPM3	4.65	1.33E-13	2.99	8.92E-13
205159_at	colony stimulating factor 2 receptor, beta, low-affinity (granulocyte-macrophage)	CSF2RB	0.33	6.77E-08	0.33	2.52E-08
205193_at	v-maf musculoaponeurotic fibrosarcoma oncogene homolog F (avian)	MAFF	0.32	9.62E-10	0.25	5.04E-13
205194_at	phosphoserine phosphatase	PSPH	4.65	8.07E-15	3.52	4.58E-12
205207_at	interleukin 6 (interferon, beta 2)	IL6	0.06	6.76E-11	0.04	5.90E-14
205236_x_at	superoxide dismutase 3, extracellular	SOD3	0.27	1.22E-11	0.41	3.26E-11
205237_at	ficolin (collagen/fibrinogen domain containing) 1	FCN1	0.09	1.47E-15	0.13	7.34E-12
205242_at	chemokine (C-X-C motif) ligand 13	CXCL13	10.55	3.96E-09	8.47	3.37E-07
205247_at	notch 4	NOTCH4	0.26	7.48E-17	0.28	1.01E-18
205249_at	early growth response 2	EGR2	0.36	5.00E-07	0.42	5.60E-10
205261_at	progastricsin (pepsinogen C)	PGC	0.03	3.37E-14	0.14	1.02E-06
205267_at	POU class 2 associating factor 1	POU2AF1	2.18	4.73E-03	3.65	4.98E-07
205277_at	PR domain containing 2, with ZNF domain	PRDM2	0.49	1.14E-09	0.42	8.72E-11
205282_at	low density lipoprotein receptor-related protein 8, apolipoprotein e receptor	LRP8	4.94	1.57E-11	2.22	9.49E-07
205286_at	transcription factor AP-2 gamma (activating	TFAP2C	4.80	8.29E-12	2.75	4.69E-09

Probeset ID	Gene title	Gene symbol	ESP (GSE18842) Fold change	Adjusted P	TWN (GSE19804) Fold change	Adjusted P
	enhancer binding protein 2 gamma)					
205289_at	bone morphogenetic protein 2	BMP2	0.18	1.39E-10	0.20	4.97E-12
205290_s_at	bone morphogenetic protein 2	BMP2	0.17	8.92E-10	0.17	2.83E-12
205303_at	potassium inwardly-rectifying channel, subfamily J, member 8	KCNJ8	0.34	2.28E-10	0.25	3.01E-15
205304_s_at	potassium inwardly-rectifying channel, subfamily J, member 8	KCNJ8	0.38	1.14E-08	0.26	3.19E-14
205306_x_at	kynurenine 3-monooxygenase (kynurenine 3-hydroxylase)	KMO	0.15	8.09E-12	0.38	2.19E-05
205316_at	solute carrier family 15 (H+/peptide transporter), member 2	SLC15A2	0.22	4.83E-09	0.49	1.73E-04
205326_at	receptor (G protein-coupled) activity modifying protein 3	RAMP3	0.10	4.41E-20	0.09	2.64E-19
205328_at	claudin 10	CLDN10	6.34	3.12E-08	2.61	6.52E-04
205339_at	SCL/TAL1 interrupting locus	STIL	5.90	2.45E-18	3.92	1.27E-13
205351_at	gamma-glutamyl carboxylase	GGCX	2.07	9.43E-09	2.35	2.01E-10
205382_s_at	complement factor D (adipsin)	CFD	0.08	3.31E-19	0.15	6.00E-15
205404_at	hydroxysteroid (11-beta) dehydrogenase 1	HSD11B1	0.40	2.04E-05	0.35	3.65E-07
205407_at	reversion-inducing-cysteine-rich protein with kazal motifs	RECK	0.24	4.22E-14	0.28	3.00E-13
205414_s_at	Rho GTPase activating protein 44	ARHGAP4 4	0.15	1.99E-21	0.37	6.68E-10
205431_s_at	bone morphogenetic protein 5	BMP5	0.23	1.65E-09	0.37	9.68E-07
205433_at	butyrylcholinesterase	BCHE	0.23	1.35E-06	0.09	8.39E-12
205442_at	microfibrillar-associated protein 3-like	MFAP3L	0.26	7.86E-09	0.20	9.26E-12
205449_at	SAC3 domain containing 1	SAC3D1	3.07	8.27E-15	2.04	1.29E-12
205462_s_at	hippocalcin-like 1	HPCAL1	0.30	5.87E-13	0.40	2.87E-10
205464_at	sodium channel, non-voltage-gated 1, beta subunit	SCNN1B	0.24	8.75E-10	0.36	1.97E-06
205471_s_at	dachshund homolog 1 (Drosophila)	DACH1	0.15	6.15E-13	0.13	7.36E-16
205479_s_at	plasminogen activator, urokinase	PLAU	4.94	5.96E-11	3.47	8.75E-10
205488_at	granzyme A (granzyme 1, cytotoxic T-lymphocyte-associated serine esterase 3)	GZMA	0.50	3.51E-04	0.38	2.66E-06
205495_s_at	granulysin	GNLY	0.21	2.35E-08	0.09	1.15E-13
205529_s_at	runt-related transcription factor 1; translocated to, 1 (cyclin D-related)	RUNX1T1	0.28	3.35E-08	0.34	5.35E-07
205542_at	six transmembrane epithelial antigen of the prostate 1	STEAP1	6.79	3.25E-14	2.35	8.49E-05
205554_s_at	deoxyribonuclease I-like 3	DNASE1L3	0.09	1.21E-13	0.11	5.94E-16
205568_at	aquaporin 9	AQP9	0.24	1.91E-09	0.23	2.15E-10
205569_at	lysosomal-associated membrane protein 3	LAMP3	0.11	8.55E-15	0.20	5.85E-14
205608_s_at	angiopoietin 1	ANGPT1	0.14	9.44E-16	0.15	8.62E-14
205609_at	angiopoietin 1	ANGPT1	0.09	1.92E-16	0.19	3.55E-10
205612_at	multimerin 1	MMRN1	0.12	1.30E-14	0.11	6.92E-13
205624_at	carboxypeptidase A3 (mast cell)	CPA3	0.14	1.69E-10	0.33	1.25E-05
205632_s_at	phosphatidylinositol-4-phosphate 5-kinase, type I, beta	PIP5K1B	0.09	6.02E-18	0.13	1.19E-15
205640_at	aldehyde dehydrogenase 3 family, member B1	ALDH3B1	0.14	3.59E-12	0.38	3.63E-11
205648_at	wingless-type MMTV integration site family member 2	WNT2	0.34	1.20E-06	0.47	2.84E-06
205651_x_at	Rap guanine nucleotide exchange factor (GEF) 4	RAPGEF4	0.25	1.34E-14	0.18	2.48E-18
205654_at	complement component 4 binding protein, alpha	C4BPA	0.04	1.26E-10	0.40	3.78E-03
205656_at	protocadherin 17	PCDH17	0.37	3.80E-09	0.23	1.69E-13
205660_at	2'-5'-oligoadenylate synthetase-like	OASL	0.26	1.99E-08	0.29	1.92E-08
205681_at	BCL2-related protein A1	BCL2A1	0.26	5.48E-08	0.34	1.84E-07
205683_x_at	tryptase alpha/beta 1	TPSAB1	0.14	8.96E-12	0.33	5.17E-07
205687_at	ubiquitin family domain containing 1	UBFD1	3.07	8.47E-17	2.66	1.25E-12
205700_at	hydroxysteroid (17-beta) dehydrogenase 6 homolog (mouse)	HSD17B6	0.07	2.98E-17	0.23	1.33E-05

Probeset ID	Gene title	Gene symbol	ESP (GSE18842) Fold change	ESP (GSE18842) Adjusted P	TWN (GSE19804) Fold change	TWN (GSE19804) Adjusted P
205715_at	bone marrow stromal cell antigen 1	BST1	0.31	1.79E-10	0.37	7.00E-12
205725_at	secretoglobin, family 1A, member 1 (uteroglobin)	SCGB1A1	0.04	4.44E-08	0.04	1.16E-09
205733_at	Bloom syndrome, RecQ helicase-like	BLM	6.09	1.66E-16	2.14	8.16E-07
205739_x_at	zinc finger protein 107	ZNF107	2.34	2.08E-08	2.27	6.29E-08
205750_at	biphenyl hydrolase-like (serine hydrolase)	BPHL	2.11	7.40E-09	2.12	5.84E-12
205752_s_at	glutathione S-transferase mu 5	GSTM5	0.25	9.67E-17	0.41	4.27E-14
205761_s_at	dihydrouridine synthase 4-like (S. cerevisiae)	DUS4L	3.20	2.29E-15	2.02	1.14E-09
205782_at	fibroblast growth factor 7	FGF7	0.35	1.53E-09	0.35	3.49E-08
205805_s_at	receptor tyrosine kinase-like orphan receptor 1	ROR1	0.24	1.12E-11	0.45	1.33E-10
205819_at	macrophage receptor with collagenous structure	MARCO	0.04	2.61E-16	0.12	2.57E-12
205846_at	protein tyrosine phosphatase, receptor type, B	PTPRB	0.11	5.55E-16	0.11	2.52E-17
205857_at	solute carrier family 18 (vesicular monoamine), member 2	SLC18A2	0.23	1.25E-15	0.48	3.68E-06
205863_at	S100 calcium binding protein A12	S100A12	0.10	9.49E-11	0.06	3.71E-13
205866_at	ficolin (collagen/fibrinogen domain containing) 3 (Hakata antigen)	FCN3	0.03	4.84E-19	0.04	8.34E-16
205876_at	leukemia inhibitory factor receptor alpha	LIFR	0.36	5.39E-11	0.27	3.50E-12
205883_at	zinc finger and BTB domain containing 16	ZBTB16	0.06	1.14E-15	0.11	2.63E-14
205898_at	chemokine (C-X3-C motif) receptor 1	CX3CR1	0.08	6.61E-16	0.24	7.97E-10
205909_at	polymerase (DNA directed), epsilon 2, accessory subunit	POLE2	6.48	2.45E-16	2.46	3.66E-09
205934_at	phospholipase C-like 1	PLCL1	0.40	1.89E-09	0.28	3.12E-15
205935_at	forkhead box F1	FOXF1	0.12	1.36E-21	0.14	7.03E-17
205941_s_at	collagen, type X, alpha 1	COL10A1	26.71	9.42E-16	72.38	7.71E-22
205943_at	tryptophan 2,3-dioxygenase	TDO2	3.33	2.53E-05	3.49	6.38E-07
205952_at	potassium channel, subfamily K, member 3	KCNK3	0.05	4.38E-18	0.05	1.21E-17
205978_at	klotho	KL	0.14	7.49E-18	0.10	2.71E-19
205982_x_at	surfactant protein C	SFTPC	0.00	7.33E-16	0.04	4.17E-09
205992_s_at	interleukin 15	IL15	0.36	1.79E-06	0.48	6.87E-07
205993_s_at	T-box 2	TBX2	0.33	1.20E-11	0.33	5.47E-13
205997_at	ADAM metallopeptidase domain 28	ADAM28	2.33	2.50E-06	4.09	1.64E-08
206049_at	selectin P (granule membrane protein 140kDa, antigen CD62)	SELP	0.13	4.00E-12	0.18	4.86E-14
206068_s_at	acyl-CoA dehydrogenase, long chain	ACADL	0.06	1.13E-19	0.10	6.06E-15
206074_s_at	high mobility group AT-hook 1	HMGA1	5.92	2.54E-15	2.36	3.11E-11
206101_at	extracellular matrix protein 2, female organ and adipocyte specific	ECM2	0.21	9.64E-13	0.36	1.64E-07
206102_at	GINS complex subunit 1 (Psf1 homolog)	GINS1	23.95	1.38E-23	8.71	7.89E-15
206115_at	early growth response 3	EGR3	0.21	4.29E-10	0.22	1.46E-11
206134_at	ADAM-like, decysin 1	ADAMDEC 1	9.32	1.69E-10	6.23	7.12E-07
206157_at	pentraxin 3, long	PTX3	0.20	5.25E-06	0.19	4.73E-08
206170_at	adrenoceptor beta 2, surface	ADRB2	0.10	2.83E-16	0.13	5.97E-16
206191_at	ectonucleoside triphosphate diphosphohydrolase 3	ENTPD3	2.18	9.29E-06	2.73	1.94E-06
206197_at	NME/NM23 family member 5	NME5	0.22	1.16E-09	0.47	5.97E-05
206201_s_at	mesenchyme homeobox 2	MEOX2	0.32	7.89E-06	0.42	2.96E-04
206209_s_at	carbonic anhydrase IV	CA4	0.02	1.06E-22	0.01	1.08E-22
206211_at	selectin E	SELE	0.04	1.62E-13	0.14	1.11E-08
206227_at	cartilage intermediate layer protein, nucleotide pyrophosphohydrolase	CILP	2.55	5.82E-03	5.30	2.33E-06
206236_at	G protein-coupled receptor 4	GPR4	0.48	2.78E-06	0.31	3.40E-15
206283_s_at	T-cell acute lymphocytic leukemia 1	TAL1	0.14	1.82E-18	0.09	5.89E-21
206311_s_at	phospholipase A2, group IB (pancreas)	PLA2G1B	0.04	2.93E-22	0.18	2.96E-06
206316_s_at	kinetochore associated 1	KNTC1	4.64	2.77E-20	2.36	3.56E-08
206331_at	calcitonin receptor-like	CALCRL	0.20	2.26E-14	0.17	1.71E-12
206359_at	suppressor of cytokine signaling 3	SOCS3	0.25	5.07E-12	0.25	3.95E-13

Probeset ID	Gene title	Gene symbol	ESP (GSE18842) Fold change	ESP (GSE18842) Adjusted P	TWN (GSE19804) Fold change	TWN (GSE19804) Adjusted P
206364_at	kinesin family member 14	KIF14	18.05	2.67E-29	3.82	2.28E-10
206374_at	dual specificity phosphatase 8	DUSP8	0.41	4.88E-11	0.41	1.63E-11
206377_at	forkhead box F2	FOXF2	0.41	2.79E-06	0.33	9.61E-08
206432_at	hyaluronan synthase 2	HAS2	0.44	1.74E-04	0.40	5.37E-05
206453_s_at	NDRG family member 2	NDRG2	0.35	7.06E-09	0.32	1.42E-17
206481_s_at	LIM domain binding 2	LDB2	0.10	1.65E-21	0.15	3.87E-18
206488_s_at	CD36 molecule (thrombospondin receptor)	CD36	0.06	3.81E-17	0.10	1.03E-15
206496_at	flavin containing monooxygenase 3	FMO3	0.34	1.53E-06	0.36	9.02E-08
206499_s_at	regulator of chromosome condensation 1	RCC1	2.99	1.03E-12	3.79	5.25E-16
206595_at	cystatin E/M	CST6	0.16	6.44E-10	0.30	5.13E-09
206600_s_at	solute carrier family 16, member 5 (monocarboxylic acid transporter 6)	SLC16A5	0.28	2.11E-11	0.46	1.14E-08
206618_at	interleukin 18 receptor 1	IL18R1	0.32	4.50E-11	0.17	5.93E-16
206631_at	prostaglandin E receptor 2 (subtype EP2), 53kDa	PTGER2	0.40	1.44E-08	0.47	3.21E-08
206637_at	purinergic receptor P2Y, G-protein coupled, 14	P2RY14	0.18	4.84E-15	0.22	8.18E-14
206701_x_at	endothelin receptor type B	EDNRB	0.07	1.32E-20	0.05	1.64E-20
206702_at	TEK tyrosine kinase, endothelial	TEK	0.08	1.99E-21	0.10	6.25E-20
206707_x_at	family with sequence similarity 65, member B	FAM65B	0.32	3.18E-08	0.27	1.13E-09
206710_s_at	erythrocyte membrane protein band 4.1-like 3	EPB41L3	0.39	5.16E-09	0.29	2.61E-08
206715_at	transcription factor EC	TFEC	0.23	6.62E-10	0.47	3.51E-04
206747_at	G protein regulated inducer of neurite outgrowth 2	GPRIN2	0.38	2.62E-12	0.37	2.15E-13
206767_at	RNA binding motif, single stranded interacting protein 3	RBMS3	0.39	3.35E-11	0.40	1.07E-09
206855_s_at	hyaluronoglucosaminidase 2	HYAL2	0.32	8.00E-17	0.36	5.40E-16
206857_s_at	FK506 binding protein 1B, 12.6 kDa	FKBP1B	0.41	8.21E-08	0.34	8.79E-12
206858_s_at	homeobox C6	HOXC6	3.12	9.28E-07	2.33	2.95E-03
206874_s_at	STE20-like kinase	SLK	0.40	2.53E-12	0.47	6.83E-15
206884_s_at	sciellin	SCEL	0.12	5.39E-10	0.15	5.07E-09
206932_at	cholesterol 25-hydroxylase	CH25H	0.24	1.38E-08	0.29	1.32E-08
206949_s_at	RUN and SH3 domain containing 1	RUSC1	2.23	5.59E-16	2.09	8.47E-14
206953_s_at	latrophilin 2	LPHN2	0.34	6.11E-10	0.17	3.69E-16
206995_x_at	scavenger receptor class F, member 1	SCARF1	0.35	3.70E-12	0.43	6.81E-10
207002_s_at	pleiomorphic adenoma gene-like 1	PLAGL1	0.38	5.65E-09	0.45	5.11E-10
207008_at	chemokine (C-X-C motif) receptor 2	CXCR2	0.12	7.01E-13	0.10	7.80E-15
207069_s_at	SMAD family member 6	SMAD6	0.15	8.92E-15	0.26	3.82E-13
207122_x_at	sulfotransferase family, cytosolic, 1A, phenol-preferring, member 2	SULT1A2	0.26	7.28E-15	0.46	1.10E-11
207134_x_at	tryptase beta 2 (gene/pseudogene)	TPSB2	0.14	9.33E-12	0.35	1.32E-06
207165_at	hyaluronan-mediated motility receptor (RHAMM)	HMMR	12.97	2.59E-21	9.33	2.91E-14
207194_s_at	intercellular adhesion molecule 4 (Landsteiner-Wiener blood group)	ICAM4	0.12	1.54E-13	0.42	2.70E-04
207238_s_at	protein tyrosine phosphatase, receptor type, C	PTPRC	0.40	2.68E-05	0.38	1.97E-05
207265_s_at	KDEL (Lys-Asp-Glu-Leu) endoplasmic reticulum protein retention receptor 3	KDELR3	5.39	2.58E-14	4.46	1.88E-12
207426_s_at	tumor necrosis factor (ligand) superfamily, member 4	TNFSF4	3.03	3.79E-10	2.88	2.88E-09
207526_s_at	interleukin 1 receptor-like 1	IL1RL1	0.11	2.39E-08	0.05	1.33E-12
207542_s_at	aquaporin 1 (Colton blood group)	AQP1	0.13	5.53E-13	0.17	2.93E-10
207574_s_at	growth arrest and DNA-damage-inducible, beta	GADD45B	0.12	2.80E-17	0.27	1.35E-13
207697_x_at	leukocyte immunoglobulin-like receptor, subfamily B (with TM and ITIM domains), member 2	LILRB2	0.40	5.97E-08	0.45	8.08E-08
207714_s_at	serpin peptidase inhibitor, clade H (heat shock protein 47), member 1, (collagen binding protein 1)	SERPINH1	2.55	1.19E-11	2.14	7.70E-12
207741_x_at	tryptase alpha/beta 1	TPSAB1	0.16	3.83E-12	0.36	6.76E-07

Probeset ID	Gene title	Gene symbol	ESP (GSE18842) Fold change	ESP (GSE18842) Adjusted P	TWN (GSE19804) Fold change	TWN (GSE19804) Adjusted P
207761_s_at	methyltransferase like 7A	METTL7A	0.28	8.89E-12	0.36	4.15E-14
207808_s_at	protein S (alpha)	PROS1	0.31	4.80E-13	0.35	2.26E-12
207826_s_at	inhibitor of DNA binding 3, dominant negative helix-loop-helix protein	ID3	0.41	4.28E-08	0.38	3.48E-10
207850_at	chemokine (C-X-C motif) ligand 3	CXCL3	0.10	2.88E-10	0.14	4.72E-13
207857_at	leukocyte immunoglobulin-like receptor, subfamily A (with TM domain), member 2	LILRA2	0.13	8.36E-17	0.25	1.83E-12
207957_s_at	protein kinase C, beta	PRKCB	0.26	6.44E-12	0.23	2.22E-12
207977_s_at	dermatopontin	DPT	0.45	1.86E-04	0.28	9.27E-10
207980_s_at	Cbp/p300-interacting transactivator, with Glu/Asp-rich carboxy-terminal domain, 2	CITED2	0.29	3.36E-13	0.47	3.74E-05
208018_s_at	hemopoietic cell kinase	HCK	0.30	3.91E-10	0.46	1.45E-08
208029_s_at	lysosomal protein transmembrane 4 beta	LAPTM4B	3.17	3.17E-11	2.55	4.33E-10
208051_s_at	poly(A) binding protein interacting protein 1	PAIP1	2.27	3.40E-07	2.10	1.00E-13
208056_s_at	core-binding factor, runt domain, alpha subunit 2; translocated to, 3	CBFA2T3	0.33	4.12E-13	0.21	5.73E-21
208078_s_at	salt-inducible kinase 1	SIK1	0.35	1.80E-07	0.27	1.22E-10
208079_s_at	aurora kinase A	AURKA	14.35	5.29E-22	4.99	2.24E-14
208103_s_at	acidic (leucine-rich) nuclear phosphoprotein 32 family, member E	ANP32E	5.58	1.70E-20	2.88	7.56E-13
208131_s_at	prostaglandin I2 (prostacyclin) synthase	PTGIS	0.31	4.16E-05	0.29	4.59E-07
208190_s_at	lipolysis stimulated lipoprotein receptor	LSR	2.46	2.14E-09	2.46	2.17E-13
208206_s_at	RAS guanyl releasing protein 2 (calcium and DAG-regulated)	RASGRP2	0.24	3.04E-19	0.37	4.00E-14
208335_s_at	Duffy blood group, chemokine receptor	DARC	0.04	1.13E-13	0.09	3.87E-13
208370_s_at	regulator of calcineurin 1	RCAN1	0.44	2.01E-07	0.41	2.80E-12
208423_s_at	macrophage scavenger receptor 1	MSR1	0.25	1.56E-09	0.38	2.06E-05
208438_s_at	Gardner-Rasheed feline sarcoma viral (v-fgr) oncogene homolog	FGR	0.10	4.73E-17	0.23	6.36E-13
208510_s_at	peroxisome proliferator-activated receptor gamma	PPARG	0.17	6.88E-14	0.31	1.70E-08
208546_x_at	histone cluster 1, H2bh	HIST1H2BH	3.17	7.86E-11	2.79	4.67E-12
208581_x_at	metallothionein 1X	MT1X	0.31	1.25E-08	0.47	2.66E-09
208650_s_at	CD24 molecule	CD24	6.49	4.47E-11	4.82	4.86E-10
208651_x_at	CD24 molecule	CD24	3.66	5.81E-09	3.49	3.17E-09
208658_at	protein disulfide isomerase family A, member 4	PDIA4	3.20	3.83E-15	3.28	4.26E-16
208740_at	Sin3A-associated protein, 18kDa	SAP18	0.34	1.11E-15	0.45	1.45E-15
208741_at	Sin3A-associated protein, 18kDa	SAP18	0.30	4.09E-17	0.46	3.14E-13
208767_s_at	lysosomal protein transmembrane 4 beta	LAPTM4B	3.87	1.68E-12	2.49	1.24E-08
208789_at	polymerase I and transcript release factor	PTRF	0.39	9.64E-12	0.30	2.42E-15
208790_s_at	polymerase I and transcript release factor	PTRF	0.43	4.06E-10	0.22	6.87E-16
208837_at	transmembrane emp24 protein transport domain containing 3	TMED3	2.36	1.18E-13	2.56	2.09E-18
208850_s_at	Thy-1 cell surface antigen	THY1	3.27	1.59E-08	3.75	3.04E-08
208851_s_at	Thy-1 cell surface antigen	THY1	3.43	6.90E-09	3.39	4.52E-10
208869_s_at	GABA(A) receptor-associated protein like 1	GABARAPL1	0.46	6.10E-11	0.37	1.41E-16
208885_at	lymphocyte cytosolic protein 1 (L-plastin)	LCP1	0.37	7.39E-08	0.49	2.54E-06
208886_at	H1 histone family, member 0	H1F0	2.83	1.54E-13	2.14	3.43E-12
208937_s_at	inhibitor of DNA binding 1, dominant negative helix-loop-helix protein	ID1	0.41	1.21E-06	0.29	2.32E-09
208944_at	transforming growth factor, beta receptor II (70/80kDa)	TGFBR2	0.21	2.82E-15	0.40	2.28E-12
208960_s_at	Kruppel-like factor 6	KLF6	0.32	1.98E-10	0.24	1.87E-14
208961_s_at	Kruppel-like factor 6	KLF6	0.28	1.41E-14	0.27	1.80E-16
209002_s_at	calcium binding and coiled-coil domain 1	CALCOCO1	0.35	1.18E-18	0.49	3.26E-13
209031_at	cell adhesion molecule 1	CADM1	0.21	1.76E-11	0.42	1.71E-07

Probeset ID	Gene title	Gene symbol	ESP (GSE18842) Fold change	ESP (GSE18842) Adjusted P	TWN (GSE19804) Fold change	TWN (GSE19804) Adjusted P
209032_s_at	cell adhesion molecule 1	CADM1	0.16	6.18E-13	0.37	1.71E-07
209035_at	midkine (neurite growth-promoting factor 2)	MDK	4.13	1.07E-13	4.24	1.52E-14
209047_at	aquaporin 1 (Colton blood group)	AQP1	0.11	4.34E-16	0.22	1.37E-10
209064_x_at	poly(A) binding protein interacting protein 1	PAIP1	2.17	8.31E-08	2.04	3.64E-14
209116_x_at	hemoglobin, beta	HBB	0.06	2.70E-18	0.12	2.47E-13
209172_s_at	centromere protein F, 350/400kDa (mitosin)	CENPF	4.92	1.93E-13	2.56	6.34E-08
209182_s_at	chromosome 10 open reading frame 10	C10orf10	0.39	1.92E-06	0.27	5.06E-12
209183_s_at	chromosome 10 open reading frame 10	C10orf10	0.24	6.11E-10	0.31	7.93E-13
209186_at	ATPase, Ca++ transporting, cardiac muscle, slow twitch 2	ATP2A2	3.91	1.26E-19	2.07	1.76E-15
209189_at	FBJ murine osteosarcoma viral oncogene homolog	FOS	0.17	5.21E-12	0.28	8.14E-10
209209_s_at	fermitin family member 2	FERMT2	0.41	2.07E-07	0.30	1.02E-08
209210_s_at	fermitin family member 2	FERMT2	0.30	1.27E-11	0.37	2.52E-13
209242_at	paternally expressed 3	PEG3	0.29	4.27E-12	0.35	1.07E-07
209267_s_at	solute carrier family 39 (zinc transporter), member 8	SLC39A8	0.10	9.08E-19	0.31	2.22E-12
209291_at	inhibitor of DNA binding 4, dominant negative helix-loop-helix protein	ID4	0.18	2.48E-14	0.30	2.42E-10
209292_at	inhibitor of DNA binding 4, dominant negative helix-loop-helix protein	ID4	0.07	6.63E-18	0.22	1.09E-10
209293_x_at	inhibitor of DNA binding 4, dominant negative helix-loop-helix protein	ID4	0.40	5.73E-10	0.43	5.20E-11
209301_at	carbonic anhydrase II	CA2	0.20	1.11E-11	0.37	6.04E-05
209304_x_at	growth arrest and DNA-damage-inducible, beta	GADD45B	0.13	7.00E-17	0.28	1.09E-13
209305_s_at	growth arrest and DNA-damage-inducible, beta	GADD45B	0.16	2.01E-12	0.24	9.75E-11
209318_x_at	pleiomorphic adenoma gene-like 1	PLAGL1	0.42	8.34E-09	0.42	2.63E-08
209324_s_at	regulator of G-protein signaling 16	RGS16	0.33	3.12E-07	0.45	2.04E-04
209325_s_at	regulator of G-protein signaling 16	RGS16	0.41	3.58E-07	0.48	6.34E-05
209335_at	decorin	DCN	0.28	1.93E-09	0.45	3.34E-07
209355_s_at	phosphatidic acid phosphatase type 2B	PPAP2B	0.38	9.31E-10	0.25	4.19E-14
209365_s_at	extracellular matrix protein 1	ECM1	2.20	1.17E-04	3.14	3.63E-10
209369_at	annexin A3	ANXA3	0.11	6.45E-13	0.23	3.25E-10
209433_s_at	phosphoribosyl pyrophosphate amidotransferase	PPAT	7.10	2.13E-17	3.31	1.35E-13
209434_s_at	phosphoribosyl pyrophosphate amidotransferase	PPAT	7.01	1.25E-21	2.78	1.05E-14
209447_at	spectrin repeat containing, nuclear envelope 1	SYNE1	0.19	1.19E-12	0.45	1.71E-07
209470_s_at	glycoprotein M6A	GPM6A	0.02	1.14E-26	0.02	1.15E-20
209481_at	SNF related kinase	SNRK	0.38	9.69E-14	0.39	1.16E-16
209493_at	PDZ domain containing 2	PDZD2	0.15	7.68E-11	0.18	1.15E-11
209496_at	retinoic acid receptor responder (tazarotene induced) 2	RARRES2	0.37	1.17E-06	0.37	7.98E-11
209498_at	carcinoembryonic antigen-related cell adhesion molecule 1 (biliary glycoprotein)	CEACAM1	2.74	3.71E-05	5.89	7.37E-11
209508_x_at	CASP8 and FADD-like apoptosis regulator	CFLAR	0.32	2.62E-11	0.49	5.18E-11
209529_at	phosphatidic acid phosphatase type 2C	PPAP2C	10.83	1.31E-17	7.69	1.31E-18
209543_s_at	CD34 molecule	CD34	0.30	5.94E-14	0.30	3.87E-12
209555_s_at	CD36 molecule (thrombospondin receptor)	CD36	0.07	5.35E-17	0.09	8.67E-16
209610_s_at	solute carrier family 1 (glutamate/neutral amino acid transporter), member 4	SLC1A4	5.21	1.44E-15	2.68	1.44E-13
209612_s_at	alcohol dehydrogenase 1B (class I), beta polypeptide	ADH1B	0.01	1.11E-20	0.05	3.93E-13
209613_s_at	alcohol dehydrogenase 1B (class I), beta polypeptide	ADH1B	0.01	1.65E-20	0.06	3.05E-12
209621_s_at	PDZ and LIM domain 3	PDLIM3	0.46	4.26E-06	0.42	5.30E-05
209652_s_at	placental growth factor	PGF	5.20	3.99E-12	2.18	7.24E-05
209676_at	tissue factor pathway inhibitor (lipoprotein-associated coagulation inhibitor)	TFPI	0.26	3.55E-08	0.48	4.69E-05
209683_at	family with sequence similarity 49, member A	FAM49A	0.31	1.29E-12	0.33	9.17E-15

Probeset ID	Gene title	Gene symbol	ESP (GSE18842) Fold change	ESP (GSE18842) Adjusted P	TWN (GSE19804) Fold change	TWN (GSE19804) Adjusted P
209685_s_at	protein kinase C, beta	PRKCB	0.33	5.61E-10	0.37	2.19E-08
209687_at	chemokine (C-X-C motif) ligand 12	CXCL12	0.22	1.75E-10	0.30	7.07E-09
209709_s_at	hyaluronan-mediated motility receptor (RHAMM)	HMMR	12.24	9.73E-21	3.70	7.86E-10
209710_at	GATA binding protein 2	GATA2	0.16	4.91E-20	0.24	2.68E-14
209714_s_at	cyclin-dependent kinase inhibitor 3	CDKN3	16.71	9.10E-21	6.21	2.03E-11
209723_at	serpin peptidase inhibitor, clade B (ovalbumin), member 9	SERPINB9	0.38	1.15E-08	0.45	1.84E-09
209732_at	C-type lectin domain family 2, member B	CLEC2B	0.46	1.42E-07	0.50	2.22E-07
209735_at	ATP-binding cassette, sub-family G (WHITE), member 2	ABCG2	0.17	1.95E-14	0.27	1.34E-12
209771_x_at	CD24 molecule	CD24	3.37	1.48E-08	3.39	2.72E-10
209772_s_at	CD24 molecule	CD24	8.06	6.48E-12	4.41	2.19E-10
209773_s_at	ribonucleotide reductase M2	RRM2	34.10	3.18E-19	10.29	1.73E-13
209774_x_at	chemokine (C-X-C motif) ligand 2	CXCL2	0.05	3.22E-13	0.15	3.10E-12
209795_at	CD69 molecule	CD69	0.11	8.79E-15	0.24	1.14E-09
209803_s_at	pleckstrin homology-like domain, family A, member 2	PHLDA2	2.01	1.04E-04	3.26	2.39E-08
209810_at	surfactant protein B	SFTPB	0.08	1.22E-06	0.37	3.92E-03
209821_at	interleukin 33	IL33	0.09	2.45E-15	0.18	8.92E-13
209829_at	family with sequence similarity 65, member B	FAM65B	0.26	1.81E-08	0.29	1.42E-08
209841_s_at	leucine rich repeat neuronal 3	LRRN3	0.08	2.01E-16	0.09	3.26E-17
209873_s_at	plakophilin 3	PKP3	4.75	1.21E-12	2.50	9.59E-11
209875_s_at	secreted phosphoprotein 1	SPP1	32.47	2.42E-14	52.52	8.66E-19
209879_at	selectin P ligand	SELPLG	0.22	6.74E-13	0.45	4.96E-11
209894_at	leptin receptor	LEPR	0.10	3.39E-16	0.23	1.34E-15
209897_s_at	slit homolog 2 (Drosophila)	SLIT2	0.17	7.19E-15	0.21	4.66E-15
209911_x_at	histone cluster 1, H2bd	HIST1H2BD	4.21	7.92E-12	4.08	3.10E-12
209921_at	solute carrier family 7 (anionic amino acid transporter light chain, xc- system), member 11	SLC7A11	10.32	4.30E-11	3.36	8.67E-08
209949_at	neutrophil cytosolic factor 2	NCF2	0.19	8.84E-13	0.39	2.49E-06
209960_at	hepatocyte growth factor (hepapoietin A; scatter factor)	HGF	0.22	2.98E-14	0.43	2.95E-07
210002_at	GATA binding protein 6	GATA6	0.11	2.28E-14	0.29	2.39E-09
210004_at	oxidized low density lipoprotein (lectin-like) receptor 1	OLR1	0.06	5.37E-17	0.28	2.22E-07
210038_at	protein kinase C, theta	PRKCQ	0.17	2.82E-15	0.28	2.82E-11
210039_s_at	protein kinase C, theta	PRKCQ	0.35	1.05E-10	0.37	2.18E-09
210046_s_at	isocitrate dehydrogenase 2 (NADP+), mitochondrial	IDH2	2.91	3.85E-14	2.21	2.52E-14
210058_at	mitogen-activated protein kinase 13	MAPK13	2.03	4.13E-06	2.26	1.30E-12
210066_s_at	aquaporin 4	AQP4	0.05	2.64E-17	0.08	1.83E-13
210068_s_at	aquaporin 4	AQP4	0.03	1.22E-15	0.08	6.13E-12
210084_x_at	tryptase alpha/beta 1	TPSAB1	0.16	1.19E-11	0.37	1.10E-06
210095_s_at	insulin-like growth factor binding protein 3	IGFBP3	6.21	1.56E-12	2.75	7.90E-09
210096_at	cytochrome P450, family 4, subfamily B, polypeptide 1	CYP4B1	0.01	8.26E-16	0.18	3.36E-06
210139_s_at	peripheral myelin protein 22	PMP22	0.33	1.56E-12	0.43	4.47E-11
210140_at	cystatin F (leukocystatin)	CST7	0.27	1.27E-10	0.35	1.33E-10
210146_x_at	leukocyte immunoglobulin-like receptor, subfamily B (with TM and ITIM domains), member 2	LILRB2	0.28	2.29E-10	0.19	6.63E-12
210170_at	PDZ and LIM domain 3	PDLIM3	0.35	4.30E-07	0.27	2.18E-11
210222_s_at	reticulon 1	RTN1	0.31	1.04E-10	0.40	1.37E-09
210258_at	regulator of G-protein signaling 13	RGS13	0.10	5.08E-22	0.28	2.06E-08
210272_at	cytochrome P450, family 2, subfamily B, polypeptide 7 pseudogene 1	CYP2B7P1	0.05	7.18E-16	0.40	7.22E-03
210321_at	granzyme H (cathepsin G-like 2, protein h-	GZMH	0.21	6.96E-11	0.18	1.46E-12

Probeset ID	Gene title	Gene symbol	ESP (GSE18842) Fold change	Adjusted P	TWN (GSE19804) Fold change	Adjusted P
	CCPX)					
210347_s_at	B-cell CLL/lymphoma 11A (zinc finger protein)	BCL11A	6.19	1.40E-09	2.10	5.93E-06
210423_s_at	solute carrier family 11 (proton-coupled divalent metal ion transporters), member 1	SLC11A1	0.14	5.78E-16	0.33	1.32E-09
210517_s_at	A kinase (PRKA) anchor protein 12	AKAP12	0.28	7.61E-09	0.16	2.45E-14
210519_s_at	NAD(P)H dehydrogenase, quinone 1	NQO1	6.08	2.06E-11	4.29	4.26E-13
210559_s_at	cyclin-dependent kinase 1	CDK1	20.90	2.33E-23	3.71	3.37E-10
210619_s_at	hyaluronoglucosaminidase 1	HYAL1	0.09	1.91E-13	0.12	6.42E-13
210629_x_at	leukocyte specific transcript 1	LST1	0.17	9.01E-15	0.39	2.05E-10
210657_s_at	septin 4	SEPT4	0.23	1.49E-15	0.37	1.83E-15
210762_s_at	deleted in liver cancer 1	DLC1	0.09	4.46E-19	0.20	1.88E-14
210764_s_at	cysteine-rich, angiogenic inducer, 61	CYR61	0.33	5.63E-07	0.32	5.43E-09
210772_at	formyl peptide receptor 2	FPR2	0.21	2.03E-11	0.13	1.88E-13
210785_s_at	chromosome 1 open reading frame 38	C1orf38	0.28	1.18E-11	0.45	5.86E-08
210786_s_at	Friend leukemia virus integration 1	FLI1	0.21	4.69E-12	0.22	3.08E-11
210797_s_at	2'-5'-oligoadenylate synthetase-like	OASL	0.43	2.06E-07	0.38	7.77E-09
210815_s_at	calcitonin receptor-like	CALCRL	0.21	7.50E-11	0.12	1.33E-11
210869_s_at	melanoma cell adhesion molecule	MCAM	0.47	1.40E-05	0.35	1.04E-09
210873_x_at	apolipoprotein B mRNA editing enzyme, catalytic polypeptide-like 3A	APOBEC3 A	0.41	1.56E-04	0.21	5.24E-09
210906_x_at	aquaporin 4	AQP4	0.03	2.04E-15	0.11	6.96E-11
210944_s_at	calpain 3, (p94)	CAPN3	0.08	2.29E-21	0.46	1.63E-06
210959_s_at	steroid-5-alpha-reductase, alpha polypeptide 1 (3-oxo-5 alpha-steroid delta 4-dehydrogenase alpha 1)	SRD5A1	4.51	1.54E-13	2.64	1.77E-09
211048_s_at	protein disulfide isomerase family A, member 4	PDIA4	3.62	1.32E-16	2.48	1.05E-13
211138_s_at	kynurenine 3-monooxygenase (kynurenine 3-hydroxylase)	KMO	0.17	4.49E-12	0.38	5.57E-06
211161_s_at	collagen, type III, alpha 1	COL3A1	6.12	3.51E-14	4.82	7.64E-16
211317_s_at	CASP8 and FADD-like apoptosis regulator	CFLAR	0.46	1.14E-05	0.48	3.05E-06
211330_s_at	hemochromatosis	HFE	2.02	7.80E-06	2.07	2.58E-06
211339_s_at	IL2-inducible T-cell kinase	ITK	0.44	3.52E-07	0.41	4.21E-06
211343_s_at	collagen, type XIII, alpha 1	COL13A1	0.30	8.71E-09	0.28	2.57E-12
211354_s_at	leptin receptor	LEPR	0.31	2.56E-07	0.26	9.01E-11
211355_x_at	leptin receptor	LEPR	0.24	5.68E-08	0.23	7.18E-12
211356_x_at	leptin receptor	LEPR	0.25	9.22E-07	0.22	1.75E-11
211385_x_at	sulfotransferase family, cytosolic, 1A, phenol-preferring, member 2	SULT1A2	0.24	9.08E-16	0.45	1.41E-12
211434_s_at	chemokine (C-C motif) receptor-like 2	CCRL2	0.17	3.32E-12	0.42	5.21E-08
211548_s_at	hydroxyprostaglandin dehydrogenase 15-(NAD)	HPGD	0.07	1.82E-16	0.35	2.71E-06
211549_s_at	hydroxyprostaglandin dehydrogenase 15-(NAD)	HPGD	0.14	4.43E-14	0.45	4.35E-04
211564_s_at	PDZ and LIM domain 4	PDLIM4	5.44	1.35E-11	2.35	2.53E-05
211571_s_at	versican	VCAN	4.59	2.41E-09	2.25	1.57E-04
211581_x_at	leukocyte specific transcript 1	LST1	0.19	1.37E-14	0.49	9.96E-09
211582_x_at	leukocyte specific transcript 1	LST1	0.17	6.95E-14	0.44	3.57E-09
211663_x_at	prostaglandin D2 synthase 21kDa (brain)	PTGDS	0.15	2.18E-14	0.26	2.02E-09
211668_s_at	plasminogen activator, urokinase	PLAU	6.64	6.40E-12	3.65	1.25E-07
211685_s_at	neurocalcin delta	NCALD	0.33	4.86E-13	0.38	1.26E-09
211696_x_at	hemoglobin, beta	HBB	0.08	1.56E-17	0.15	5.76E-13
211726_s_at	flavin containing monooxygenase 2 (non-functional)	FMO2	0.05	1.88E-19	0.10	4.13E-18
211735_x_at	surfactant protein C	SFTPC	0.00	1.30E-15	0.04	4.86E-09
211748_x_at	prostaglandin D2 synthase 21kDa (brain)	PTGDS	0.15	6.92E-15	0.27	7.17E-10
211762_s_at	karyopherin alpha 2 (RAG cohort 1, importin alpha 1)	KPNA2	5.18	3.98E-20	2.02	3.60E-08
211776_s_at	erythrocyte membrane protein band 4.1-like 3	EPB41L3	0.46	9.66E-08	0.33	5.51E-09
211798_x_at	immunoglobulin lambda joining 3	IGLJ3	2.39	2.91E-03	2.36	2.79E-04

Probeset ID	Gene title	Gene symbol	ESP (GSE18842) Fold change	ESP (GSE18842) Adjusted P	TWN (GSE19804) Fold change	TWN (GSE19804) Adjusted P
211813_x_at	decorin	DCN	0.44	3.20E-05	0.48	2.45E-06
211881_x_at	immunoglobulin lambda joining 3	IGLJ3	2.47	2.06E-03	2.58	1.49E-04
211887_x_at	macrophage scavenger receptor 1	MSR1	0.23	1.67E-10	0.39	2.77E-05
211896_s_at	decorin	DCN	0.40	3.76E-06	0.46	3.46E-06
211922_s_at	catalase	CAT	0.25	2.88E-16	0.39	3.79E-10
211986_at	AHNAK nucleoprotein	AHNAK	0.42	2.93E-09	0.34	7.09E-14
212097_at	caveolin 1, caveolae protein, 22kDa	CAV1	0.11	2.28E-18	0.17	1.60E-15
212099_at	ras homolog family member B	RHOB	0.38	8.53E-11	0.41	3.96E-12
212115_at	hematological and neurological expressed 1-like	HN1L	3.04	1.28E-19	2.59	1.81E-18
212143_s_at	insulin-like growth factor binding protein 3	IGFBP3	8.86	9.46E-13	2.68	3.14E-06
212187_x_at	prostaglandin D2 synthase 21kDa (brain)	PTGDS	0.13	1.38E-14	0.24	1.18E-09
212188_at	potassium channel tetramerisation domain containing 12	KCTD12	0.30	5.77E-13	0.47	9.41E-10
212190_at	serpin peptidase inhibitor, clade E (nexin, plasminogen activator inhibitor type 1), member 2	SERPINE2	6.69	1.58E-13	2.16	8.30E-06
212226_s_at	phosphatidic acid phosphatase type 2B	PPAP2B	0.37	1.09E-12	0.39	2.62E-15
212230_at	phosphatidic acid phosphatase type 2B	PPAP2B	0.36	1.41E-13	0.37	1.38E-13
212288_at	formin binding protein 1	FNBP1	0.46	2.97E-10	0.48	2.04E-09
212308_at	cytoplasmic linker associated protein 2	CLASP2	0.47	1.82E-12	0.49	9.07E-10
212325_at	LIM and calponin homology domains 1	LIMCH1	0.12	1.09E-13	0.28	4.09E-09
212327_at	LIM and calponin homology domains 1	LIMCH1	0.05	7.28E-17	0.25	3.80E-11
212328_at	LIM and calponin homology domains 1	LIMCH1	0.07	2.64E-16	0.27	1.30E-11
212344_at	sulfatase 1	SULF1	19.64	1.62E-17	5.46	7.44E-12
212353_at	sulfatase 1	SULF1	20.81	4.75E-19	6.69	1.82E-13
212354_at	sulfatase 1	SULF1	16.54	1.51E-18	4.93	4.76E-12
212372_at	myosin, heavy chain 10, non-muscle	MYH10	0.34	3.22E-12	0.37	1.86E-11
212427_at	KIAA0368	KIAA0368	0.45	1.29E-12	0.46	7.46E-10
212444_at	G protein-coupled receptor, family C, group 5, member A	GPRC5A	0.09	1.63E-13	0.34	6.91E-07
212448_at	neural precursor cell expressed, developmentally down-regulated 4-like, E3 ubiquitin protein ligase	NEDD4L	0.19	1.80E-15	0.21	3.83E-13
212450_at	SECIS binding protein 2-like	SECISBP2 L	0.25	2.46E-19	0.44	1.34E-14
212451_at	SECIS binding protein 2-like	SECISBP2 L	0.23	1.62E-17	0.35	1.27E-10
212486_s_at	FYN oncogene related to SRC, FGR, YES	FYN	0.34	1.42E-11	0.25	2.05E-15
212488_at	collagen, type V, alpha 1	COL5A1	6.34	1.07E-13	4.41	9.72E-15
212489_at	collagen, type V, alpha 1	COL5A1	6.86	1.12E-14	4.39	3.45E-13
212494_at	tensin like C1 domain containing phosphatase (tensin 2)	TENC1	0.18	2.52E-19	0.45	1.23E-10
212531_at	lipocalin 2	LCN2	3.83	7.80E-04	2.73	3.94E-05
212552_at	hippocalcin-like 1	HPCAL1	0.33	1.04E-14	0.47	3.88E-17
212587_s_at	protein tyrosine phosphatase, receptor type, C	PTPRC	0.34	1.75E-07	0.41	1.82E-06
212588_at	protein tyrosine phosphatase, receptor type, C	PTPRC	0.33	1.97E-07	0.47	2.90E-06
212609_s_at	v-akt murine thymoma viral oncogene homolog 3 (protein kinase B, gamma)	AKT3	0.48	2.14E-08	0.36	4.96E-13
212636_at	QKI, KH domain containing, RNA binding	QKI	0.19	1.51E-16	0.24	3.06E-17
212647_at	related RAS viral (r-ras) oncogene homolog	RRAS	0.29	2.03E-13	0.40	3.12E-15
212665_at	TCDD-inducible poly(ADP-ribose) polymerase	TIPARP	0.39	3.98E-09	0.49	5.49E-07
212680_x_at	protein phosphatase 1, regulatory (inhibitor) subunit 14B	PPP1R14B	3.92	4.08E-19	2.66	8.31E-18
212681_at	erythrocyte membrane protein band 4.1-like 3	EPB41L3	0.40	1.70E-09	0.34	4.06E-09
212713_at	microfibrillar-associated protein 4	MFAP4	0.06	1.46E-15	0.13	4.27E-13
212730_at	synemin, intermediate filament protein	SYNM	0.29	1.72E-11	0.14	1.17E-16
212747_at	ankyrin repeat and sterile alpha motif domain containing 1A	ANKS1A	0.46	2.50E-10	0.45	5.04E-18
212764_at	zinc finger E-box binding homeobox 1	ZEB1	0.43	8.61E-09	0.46	1.04E-08

Probeset ID	Gene title	Gene symbol	ESP (GSE18842)		TWN (GSE19804)	
			Fold change	Adjusted P	Fold change	Adjusted P
212810_s_at	solute carrier family 1 (glutamate/neutral amino acid transporter), member 4	SLC1A4	5.05	2.10E-13	3.14	6.76E-14
212811_x_at	solute carrier family 1 (glutamate/neutral amino acid transporter), member 4	SLC1A4	5.91	6.11E-15	2.85	2.62E-14
212813_at	junctional adhesion molecule 3	JAM3	0.42	2.62E-08	0.37	2.81E-12
212814_at	adenosylhomocysteinase-like 2	AHCYL2	0.27	1.69E-10	0.45	2.11E-05
212822_at	HEG homolog 1 (zebrafish)	HEG1	0.39	5.30E-09	0.24	1.65E-16
212828_at	synaptojanin 2	SYNJ2	2.88	1.28E-10	2.84	6.00E-12
212872_s_at	mediator complex subunit 20	MED20	2.11	2.06E-12	2.10	1.32E-14
212886_at	coiled-coil domain containing 69	CCDC69	0.19	1.40E-15	0.35	7.05E-11
212902_at	SEC24 family, member A (S. cerevisiae)	SEC24A	2.28	4.27E-11	2.39	1.77E-11
212912_at	ribosomal protein S6 kinase, 90kDa, polypeptide 2	RPS6KA2	0.20	1.41E-13	0.47	8.51E-09
212914_at	chromobox homolog 7	CBX7	0.23	5.16E-16	0.37	2.26E-13
212915_at	PDZ domain containing ring finger 3	PDZRN3	0.47	2.02E-07	0.45	2.31E-07
212923_s_at	PX domain containing 1	PXDC1	0.39	8.51E-14	0.43	7.88E-14
212942_s_at	KIAA1199	KIAA1199	5.25	1.42E-09	3.35	2.61E-06
212993_at	NACC family member 2, BEN and BTB (POZ) domain containing	NACC2	0.41	2.12E-12	0.47	2.08E-14
213006_at	CCAAT/enhancer binding protein (C/EBP), delta	CEBPD	0.20	4.72E-12	0.18	2.80E-12
213007_at	Fanconi anemia, complementation group I	FANCI	10.96	2.15E-20	3.14	6.77E-12
213058_at	tetratricopeptide repeat domain 28	TTC28	0.48	4.09E-08	0.36	1.21E-14
213060_s_at	chitinase 3-like 2	CHI3L2	0.07	1.48E-18	0.25	1.25E-08
213067_at	myosin, heavy chain 10, non-muscle	MYH10	0.37	9.75E-08	0.29	2.06E-11
213068_at	dermatopontin	DPT	0.30	1.71E-05	0.19	1.64E-09
213069_at	HEG homolog 1 (zebrafish)	HEG1	0.39	3.97E-09	0.28	2.78E-14
213071_at	dermatopontin	DPT	0.26	1.86E-06	0.22	3.75E-11
213076_at	inositol-trisphosphate 3-kinase C	ITPKC	0.47	4.53E-05	0.43	3.07E-08
213094_at	G protein-coupled receptor 126	GPR126	0.24	3.56E-08	0.32	8.61E-10
213103_at	StAR-related lipid transfer (START) domain containing 13	STARD13	0.16	9.62E-19	0.19	2.18E-20
213116_at	NIMA (never in mitosis gene a)-related kinase 3	NEK3	0.41	9.48E-12	0.46	7.38E-09
213138_at	AT rich interactive domain 5A (MRF1-like)	ARID5A	0.44	2.83E-09	0.39	1.69E-11
213146_at	lysine (K)-specific demethylase 6B	KDM6B	0.30	5.74E-10	0.42	1.05E-07
213160_at	dedicator of cytokinesis 2	DOCK2	0.30	1.79E-10	0.48	9.89E-07
213169_at	sema domain, seven thrombospondin repeats (type 1 and type 1-like), transmembrane domain (TM) and short cytoplasmic domain, (semaphorin) 5A	SEMA5A	0.18	4.06E-13	0.16	1.29E-16
213182_x_at	cyclin-dependent kinase inhibitor 1C (p57, Kip2)	CDKN1C	0.35	4.59E-08	0.35	9.05E-12
213221_s_at	salt-inducible kinase 2	SIK2	0.44	1.57E-11	0.36	5.62E-19
213226_at	cyclin A2	CCNA2	10.65	1.45E-20	3.51	7.37E-11
213236_at	SAM and SH3 domain containing 1	SASH1	0.34	1.58E-14	0.29	2.48E-18
213237_at	chromosome 16 open reading frame 88	C16orf88	4.25	4.12E-17	2.60	4.63E-17
213278_at	myotubularin related protein 9	MTMR9	0.44	1.21E-14	0.48	5.53E-13
213306_at	multiple PDZ domain protein	MPDZ	0.46	8.61E-10	0.37	4.70E-13
213307_at	SH3 and multiple ankyrin repeat domains 2	SHANK2	0.24	1.95E-14	0.39	2.84E-06
213316_at	KIAA1462	KIAA1462	0.20	1.24E-12	0.13	6.33E-18
213317_at	chloride intracellular channel 5	CLIC5	0.01	1.31E-21	0.05	6.65E-16
213348_at	cyclin-dependent kinase inhibitor 1C (p57, Kip2)	CDKN1C	0.28	2.36E-10	0.35	4.62E-11
213364_s_at	sorting nexin 1	SNX1	0.31	5.47E-15	0.37	1.34E-13
213375_s_at	NEDD4 binding protein 2-like 1	N4BP2L1	0.19	2.25E-16	0.49	7.86E-11
213427_at	ribonuclease P/MRP 40kDa subunit	RPP40	3.20	7.33E-13	2.17	1.78E-08
213434_at	syntaxin 2	STX2	0.45	6.47E-11	0.40	2.90E-11
213506_at	coagulation factor II (thrombin) receptor-like 1	F2RL1	3.67	1.44E-09	3.29	3.28E-13
213524_s_at	G0/G1switch 2	G0S2	0.25	2.34E-07	0.35	1.69E-08
213541_s_at	v-ets erythroblastosis virus E26 oncogene	ERG	0.19	2.14E-16	0.15	1.74E-13

Probeset ID	Gene title	Gene symbol	ESP (GSE18842) Fold change	Adjusted P	TWN (GSE19804) Fold change	Adjusted P
	homolog (avian)					
213618_at	ArfGAP with RhoGAP domain, ankyrin repeat and PH domain 2	ARAP2	0.31	7.68E-11	0.38	6.23E-16
213620_s_at	intercellular adhesion molecule 2	ICAM2	0.16	3.16E-17	0.29	9.87E-18
213664_at	solute carrier family 1 (neuronal/epithelial high affinity glutamate transporter, system Xag), member 1	SLC1A1	0.09	1.37E-15	0.17	1.10E-11
213668_s_at	SRY (sex determining region Y)-box 4	SOX4	3.97	8.53E-13	3.06	1.49E-09
213675_at	parvin, alpha	PARVA	0.40	6.52E-12	0.46	6.08E-11
213689_x_at	family with sequence similarity 69, member A	FAM69A	2.21	2.63E-08	2.63	1.30E-13
213715_s_at	KN motif and ankyrin repeat domains 3	KANK3	0.08	8.39E-23	0.08	2.76E-21
213733_at	myosin IF	MYO1F	0.25	3.96E-13	0.46	1.40E-09
213805_at	abhydrolase domain containing 5	ABHD5	0.46	1.35E-08	0.48	7.49E-10
213817_at	interleukin-1 receptor-associated kinase 3	IRAK3	0.24	4.23E-10	0.38	7.31E-06
213844_at	homeobox A5	HOXA5	0.19	2.56E-13	0.22	3.19E-13
213869_x_at	Thy-1 cell surface antigen	THY1	3.49	7.53E-09	3.85	5.71E-11
213894_at	thrombospondin, type I, domain containing 7A	THSD7A	0.36	1.21E-06	0.43	2.15E-06
213895_at	epithelial membrane protein 1	EMP1	0.22	2.83E-09	0.12	1.58E-14
213900_at	family with sequence similarity 189, member A2	FAM189A2	0.04	1.59E-21	0.13	3.22E-16
213909_at	leucine rich repeat containing 15	LRRC15	8.60	4.07E-10	8.99	9.35E-11
213915_at	natural killer cell group 7 sequence	NKG7	0.22	1.05E-09	0.13	1.34E-13
213935_at	abhydrolase domain containing 5	ABHD5	0.37	1.25E-13	0.48	2.80E-12
213936_x_at	surfactant protein B	SFTPB	0.05	2.67E-10	0.26	9.90E-05
213943_at	twist homolog 1 (Drosophila)	TWIST1	7.61	1.02E-11	3.43	3.59E-06
213974_at	ADAMTS-like 3	ADAMTSL3	0.14	1.98E-14	0.09	4.11E-17
214039_s_at	lysosomal protein transmembrane 4 beta	LAPTM4B	2.42	6.06E-11	2.34	2.83E-13
214043_at	protein tyrosine phosphatase, receptor type, D	PTPRD	0.49	4.39E-04	0.35	1.61E-07
214054_at	docking protein 2, 56kDa	DOK2	0.19	1.48E-18	0.47	7.35E-10
214074_s_at	cortactin	CTTN	5.39	1.34E-11	2.40	3.62E-10
214084_x_at	neutrophil cytosolic factor 1C pseudogene	NCF1C	0.29	5.07E-10	0.49	1.47E-06
214088_s_at	fucosyltransferase 3 (galactoside 3(4)-L-fucosyltransferase, Lewis blood group)	FUT3	4.40	4.48E-10	5.97	3.55E-16
214091_s_at	glutathione peroxidase 3 (plasma)	GPX3	0.08	3.16E-19	0.24	9.74E-14
214096_s_at	serine hydroxymethyltransferase 2 (mitochondrial)	SHMT2	4.65	2.59E-20	2.25	2.06E-13
214106_s_at	GDP-mannose 4,6-dehydratase	GMDS	2.06	1.50E-09	2.65	2.38E-10
214135_at	claudin 18	CLDN18	0.01	6.04E-21	0.04	9.54E-13
214146_s_at	pro-platelet basic protein (chemokine (C-X-C motif) ligand 7)	PPBP	0.06	6.42E-13	0.07	7.47E-11
214181_x_at	leukocyte specific transcript 1	LST1	0.17	5.70E-15	0.43	8.65E-09
214193_s_at	digestive organ expansion factor homolog (zebrafish)	DIEXF	2.42	5.01E-15	2.13	8.70E-11
214199_at	surfactant protein D	SFTPD	0.03	1.42E-12	0.15	2.56E-07
214212_x_at	fermitin family member 2	FERMT2	0.40	4.11E-09	0.38	1.70E-10
214264_s_at	EF-hand calcium binding domain 11	EFCAB11	3.26	1.22E-11	2.09	3.15E-08
214265_at	integrin, alpha 8	ITGA8	0.09	4.74E-18	0.21	2.87E-13
214318_s_at	furry homolog (Drosophila)	FRY	0.17	6.57E-16	0.28	1.62E-11
214319_at	furry homolog (Drosophila)	FRY	0.17	8.47E-21	0.40	1.55E-06
214354_x_at	surfactant protein B	SFTPB	0.05	3.64E-11	0.30	4.45E-04
214366_s_at	arachidonate 5-lipoxygenase	ALOX5	0.09	2.46E-16	0.29	5.02E-09
214369_s_at	RAS guanyl releasing protein 2 (calcium and DAG-regulated)	RASGRP2	0.23	1.44E-19	0.47	4.03E-11
214387_x_at	surfactant protein C	SFTPC	0.00	2.61E-15	0.05	9.42E-09
214438_at	H2.0-like homeobox	HLX	0.34	1.50E-09	0.25	5.90E-14
214467_at	G protein-coupled receptor 65	GPR65	0.21	2.82E-13	0.36	3.05E-09
214574_x_at	leukocyte specific transcript 1	LST1	0.17	2.69E-14	0.46	6.01E-09
214581_x_at	tumor necrosis factor receptor superfamily,	TNFRSF21	4.91	2.33E-12	2.15	6.09E-06

Probeset ID	Gene title	Gene symbol	ESP (GSE18842) Fold change	ESP (GSE18842) Adjusted P	TWN (GSE19804) Fold change	TWN (GSE19804) Adjusted P
	member 21					
214582_at	phosphodiesterase 3B, cGMP-inhibited	PDE3B	0.44	4.25E-13	0.31	3.90E-13
214617_at	perforin 1 (pore forming protein)	PRF1	0.31	4.79E-08	0.17	6.26E-13
214710_s_at	cyclin B1	CCNB1	27.86	2.63E-24	5.80	4.71E-13
214724_at	DIX domain containing 1	DIXDC1	0.42	9.17E-10	0.39	2.77E-11
214761_at	zinc finger protein 423	ZNF423	0.40	4.80E-06	0.31	1.22E-13
214770_at	macrophage scavenger receptor 1	MSR1	0.13	1.34E-14	0.39	9.10E-08
214805_at	eukaryotic translation initiation factor 4A1	EIF4A1	0.19	5.35E-13	0.36	1.30E-09
214906_x_at	NEDD4 binding protein 2-like 1	N4BP2L1	0.32	5.96E-17	0.48	1.01E-08
214920_at	thrombospondin, type I, domain containing 7A	THSD7A	0.33	4.28E-08	0.33	1.23E-09
214974_x_at	chemokine (C-X-C motif) ligand 5	CXCL5	0.28	1.86E-04	0.21	6.76E-06
215076_s_at	collagen, type III, alpha 1	COL3A1	4.82	3.85E-14	3.37	7.52E-13
215214_at	Immunoglobulin lambda constant 1 (Mcg marker)	IGLC1	2.28	7.87E-03	2.37	9.82E-04
215299_x_at	sulfotransferase family, cytosolic, 1A, phenol-preferring, member 1	SULT1A1	0.24	1.19E-14	0.49	1.36E-12
215380_s_at	gamma-glutamylcyclotransferase	GGCT	3.75	2.07E-18	2.77	4.28E-18
215382_x_at	tryptase alpha/beta 1	TPSAB1	0.16	7.16E-12	0.36	7.83E-07
215454_x_at	surfactant protein C	SFTPC	0.01	1.98E-22	0.04	6.77E-14
215485_s_at	intercellular adhesion molecule 1	ICAM1	0.33	5.09E-07	0.43	1.75E-06
215617_at	spermatogenesis associated, serine-rich 2-like	SPATS2L	0.47	2.23E-08	0.33	1.23E-10
215633_x_at	leukocyte specific transcript 1	LST1	0.15	1.07E-14	0.38	4.07E-10
215646_s_at	versican	VCAN	4.60	6.78E-10	2.37	1.25E-04
215719_x_at	Fas (TNF receptor superfamily, member 6)	FAS	0.49	2.01E-05	0.39	4.69E-06
215800_at	dual oxidase 1	DUOX1	0.25	6.12E-12	0.44	1.20E-06
215933_s_at	hematopoietically expressed homeobox	HHEX	0.41	2.42E-07	0.46	1.64E-04
215945_s_at	tripartite motif containing 2	TRIM2	3.32	3.86E-13	3.06	5.15E-12
216044_x_at	family with sequence similarity 69, member A	FAM69A	2.20	3.51E-08	2.22	9.41E-11
216080_s_at	fatty acid desaturase 3	FADS3	0.37	2.93E-09	0.33	1.47E-13
216248_s_at	nuclear receptor subfamily 4, group A, member 2	NR4A2	0.11	6.37E-14	0.18	6.23E-12
216379_x_at	CD24 molecule	CD24	3.53	8.50E-09	3.61	2.63E-10
216504_s_at	solute carrier family 39 (zinc transporter), member 8	SLC39A8	0.15	1.70E-16	0.38	1.36E-10
216598_s_at	chemokine (C-C motif) ligand 2	CCL2	0.16	5.38E-09	0.36	1.76E-05
216620_s_at	Rho guanine nucleotide exchange factor (GEF) 10	ARHGEF10	0.29	2.15E-14	0.29	1.48E-18
216623_x_at	TOX high mobility group box family member 3	TOX3	2.42	7.71E-03	16.85	2.98E-16
216894_x_at	cyclin-dependent kinase inhibitor 1C (p57, Kip2)	CDKN1C	0.49	4.50E-07	0.45	3.18E-10
216905_s_at	suppression of tumorigenicity 14 (colon carcinoma)	ST14	3.15	3.18E-10	2.82	9.75E-14
216973_s_at	homeobox B7	HOXB7	3.74	1.61E-08	2.59	3.22E-07
217143_s_at	YME1-like 1 (S. cerevisiae)	YME1L1	0.25	5.09E-09	0.13	6.10E-13
217177_s_at	protein tyrosine phosphatase, receptor type, B	PTPRB	0.18	7.25E-17	0.08	1.89E-21
217197_x_at	NEDD4 binding protein 2-like 1	N4BP2L1	0.30	2.38E-14	0.37	2.27E-10
217227_x_at	immunoglobulin lambda variable 1-44	IGLV1-44	2.63	1.22E-03	2.24	1.75E-03
217232_x_at	hemoglobin, beta	HBB	0.06	1.94E-18	0.13	2.12E-13
217258_x_at	immunoglobulin lambda variable 1-44	IGLV1-44	3.06	3.28E-04	2.83	4.43E-05
217287_s_at	transient receptor potential cation channel, subfamily C, member 6	TRPC6	0.25	2.14E-09	0.30	6.05E-09
217294_s_at	enolase 1, (alpha)	ENO1	6.17	1.03E-12	2.75	1.51E-06
217428_s_at	collagen, type X, alpha 1	COL10A1	23.38	2.52E-16	66.11	3.73E-22
217437_s_at	transforming, acidic coiled-coil containing protein 1	TACC1	0.34	9.84E-09	0.29	1.07E-18
217525_at	olfactomedin-like 1	OLFML1	0.30	2.38E-11	0.25	1.40E-11
217546_at	metallothionein 1M	MT1M	0.02	4.57E-18	0.05	2.07E-15
217678_at	solute carrier family 7 (anionic amino acid transporter light chain, xc- system), member 11	SLC7A11	9.52	6.61E-11	2.50	5.26E-06

Probeset ID	Gene title	Gene symbol	ESP (GSE18842) Fold change	Adjusted P	TWN (GSE19804) Fold change	Adjusted P
217721_at	septin 7	SEPT7	0.45	2.42E-09	0.34	4.63E-17
217736_s_at	eukaryotic translation initiation factor 2-alpha kinase 1	EIF2AK1	2.09	1.94E-17	2.16	3.92E-16
217744_s_at	PERP, TP53 apoptosis effector	PERP	8.52	2.62E-15	2.02	1.15E-04
217755_at	hematological and neurological expressed 1	HN1	4.29	2.68E-17	2.57	1.66E-11
217757_at	alpha-2-macroglobulin	A2M	0.23	3.77E-14	0.49	1.05E-12
217771_at	golgi membrane protein 1	GOLM1	4.41	2.40E-12	5.97	1.84E-21
217781_s_at	zinc finger protein 106 homolog (mouse)	ZFP106	0.45	6.02E-13	0.42	8.70E-16
217785_s_at	YKT6 v-SNARE homolog (S. cerevisiae)	YKT6	3.92	2.30E-15	2.08	6.89E-09
217791_s_at	aldehyde dehydrogenase 18 family, member A1	ALDH18A1	3.83	6.48E-17	2.38	4.67E-13
217809_at	basic leucine zipper and W2 domains 2	BZW2	2.35	5.37E-12	2.40	2.19E-14
217862_at	protein inhibitor of activated STAT, 1	PIAS1	0.31	1.22E-17	0.40	3.58E-11
217867_x_at	beta-site APP-cleaving enzyme 2	BACE2	2.15	6.64E-07	3.39	6.78E-10
217897_at	FXYD domain containing ion transport regulator 6	FXYD6	0.26	5.22E-12	0.24	7.44E-16
217901_at	desmoglein 2	DSG2	5.79	1.86E-18	2.14	1.54E-07
218002_s_at	chemokine (C-X-C motif) ligand 14	CXCL14	24.70	1.83E-10	10.70	2.45E-14
218009_s_at	protein regulator of cytokinesis 1	PRC1	21.88	2.52E-21	4.39	7.78E-11
218039_at	nucleolar and spindle associated protein 1	NUSAP1	13.10	4.28E-20	4.63	4.03E-14
218073_s_at	transmembrane protein 48	TMEM48	5.45	2.84E-17	2.19	1.22E-08
218087_s_at	sorbin and SH3 domain containing 1	SORBS1	0.10	5.14E-17	0.27	4.18E-10
218151_x_at	solute carrier family 52, riboflavin transporter, member 2	SLC52A2	2.34	4.25E-14	2.04	1.70E-13
218252_at	cytoskeleton associated protein 2	CKAP2	4.17	1.36E-14	2.28	2.91E-09
218257_s_at	UDP-glucose glycoprotein glucosyltransferase 1	UGGT1	3.98	4.41E-20	2.42	2.10E-16
218305_at	importin 4	IPO4	3.05	1.45E-13	2.01	4.51E-10
218309_at	calcium/calmodulin-dependent protein kinase II inhibitor 1	CAMK2N1	0.14	1.14E-13	0.27	3.46E-11
218313_s_at	UDP-N-acetyl-alpha-D-galactosamine:polypeptide N-acetylgalactosaminyltransferase 7 (GalNAc-T7)	GALNT7	2.65	2.78E-12	3.32	2.11E-17
218326_s_at	leucine-rich repeat containing G protein-coupled receptor 4	LGR4	8.63	2.21E-22	5.13	1.24E-15
218346_s_at	sestrin 1	SESN1	0.32	1.52E-14	0.47	4.19E-08
218350_s_at	geminin, DNA replication inhibitor	GMNN	3.66	8.02E-16	2.50	3.94E-14
218380_at	uncharacterized LOC728392	LOC728392	0.36	5.08E-09	0.49	1.33E-07
218424_s_at	STEAP family member 3, metalloreductase	STEAP3	2.11	1.19E-06	3.38	1.71E-14
218451_at	CUB domain containing protein 1	CDCP1	3.59	1.16E-09	2.58	3.68E-12
218486_at	Kruppel-like factor 11	KLF11	0.43	1.19E-11	0.42	2.79E-15
218493_at	small nuclear ribonucleoprotein 25kDa (U11/U12)	SNRNP25	2.30	8.73E-11	2.09	7.03E-17
218498_s_at	ERO1-like (S. cerevisiae)	ERO1L	4.35	3.70E-13	3.24	3.57E-13
218510_x_at	family with sequence similarity 134, member B	FAM134B	0.29	6.46E-08	0.29	1.78E-12
218517_at	PHD finger protein 17	PHF17	0.29	6.51E-11	0.49	3.79E-13
218532_s_at	family with sequence similarity 134, member B	FAM134B	0.31	1.92E-07	0.30	1.07E-10
218546_at	chromosome 1 open reading frame 115	C1orf115	0.25	2.84E-13	0.30	1.73E-13
218574_s_at	LIM and cysteine-rich domains 1	LMCD1	0.36	1.78E-09	0.35	1.14E-12
218625_at	neuritin 1	NRN1	0.47	5.68E-03	0.13	1.06E-13
218631_at	arginine vasopressin-induced 1	AVPI1	0.43	4.66E-10	0.50	1.45E-08
218656_s_at	lipoma HMGIC fusion partner	LHFP	0.28	1.20E-18	0.31	8.50E-16
218662_s_at	non-SMC condensin I complex, subunit G	NCAPG	11.00	4.72E-21	4.16	1.47E-13
218663_at	non-SMC condensin I complex, subunit G	NCAPG	13.08	2.68E-22	3.83	3.80E-10
218665_at	frizzled family receptor 4	FZD4	0.25	2.73E-15	0.27	4.65E-17
218678_at	nestin	NES	0.34	1.48E-07	0.25	2.08E-14
218681_s_at	stromal cell-derived factor 2-like 1	SDF2L1	2.36	9.54E-12	2.10	2.21E-14
218704_at	ring finger protein 43	RNF43	2.81	2.19E-09	2.84	8.52E-12

Probeset ID	Gene title	Gene symbol	ESP (GSE18842)		TWN (GSE19804)	
			Fold change	Adjusted P	Fold change	Adjusted P
218711_s_at	serum deprivation response	SDPR	0.08	3.58E-16	0.08	1.02E-15
218717_s_at	leprecan-like 1	LEPREL1	0.30	4.24E-05	0.29	6.46E-08
218723_s_at	regulator of cell cycle	RGCC	0.10	2.39E-25	0.19	2.03E-17
218736_s_at	palmdelphin	PALMD	0.14	9.08E-19	0.20	1.64E-13
218756_s_at	dehydrogenase/reductase (SDR family) member 11	DHRS11	2.03	4.24E-06	2.32	1.61E-11
218764_at	protein kinase C, eta	PRKCH	0.48	1.70E-08	0.39	3.38E-15
218782_s_at	ATPase family, AAA domain containing 2	ATAD2	13.32	3.08E-21	2.38	1.48E-07
218824_at	paraneoplastic Ma antigen family-like 1	PNMAL1	2.27	5.87E-04	3.74	7.31E-09
218826_at	solute carrier family 35, member F2	SLC35F2	2.24	2.38E-06	3.38	1.72E-12
218850_s_at	LIM domains containing 1	LIMD1	0.42	2.88E-10	0.39	2.38E-09
218856_at	tumor necrosis factor receptor superfamily, member 21	TNFRSF21	4.07	8.84E-13	2.34	8.92E-12
218864_at	tensin 1	TNS1	0.40	1.20E-11	0.40	6.89E-13
218876_at	tubulin polymerization-promoting protein family member 3	TPPP3	0.10	5.56E-14	0.26	8.94E-08
218883_s_at	MLF1 interacting protein	MLF1IP	8.62	7.64E-19	4.11	4.49E-13
218897_at	transmembrane protein 177	TMEM177	4.30	4.52E-18	3.26	8.45E-16
218901_at	phospholipid scramblase 4	PLSCR4	0.29	5.16E-14	0.45	4.53E-09
218930_s_at	transmembrane protein 106B	TMEM106B	2.15	1.26E-17	2.98	7.48E-17
218950_at	ArfGAP with RhoGAP domain, ankyrin repeat and PH domain 3	ARAP3	0.32	2.54E-14	0.30	2.09E-18
218974_at	sine oculis binding protein homolog (Drosophila)	SOBP	0.32	3.11E-09	0.41	1.73E-07
218979_at	RMI1, RecQ mediated genome instability 1, homolog (S. cerevisiae)	RMI1	2.74	6.85E-13	2.06	6.18E-12
218982_s_at	mitochondrial ribosomal protein S17	MRPS17	2.77	4.84E-15	2.12	1.06E-09
218999_at	transmembrane protein 140	TMEM140	0.35	7.50E-10	0.49	5.27E-09
219004_s_at	MIS18 kinetochore protein homolog A (S. pombe)	MIS18A	3.50	2.22E-17	2.15	9.21E-11
219010_at	chromosome 1 open reading frame 106	C1orf106	6.99	2.86E-16	5.09	2.03E-12
219014_at	placenta-specific 8	PLAC8	0.23	9.74E-07	0.27	7.65E-10
219049_at	chondroitin sulfate N-acetylgalactosaminyltransferase 1	CSGALNACT1	0.35	2.96E-08	0.46	5.22E-08
219054_at	natriuretic peptide receptor C/guanylate cyclase C (atrionatriuretic peptide receptor C)	NPR3	0.45	4.21E-04	0.28	7.89E-10
219090_at	solute carrier family 24 (sodium/potassium/calcium exchanger), member 3	SLC24A3	0.41	1.18E-05	0.38	2.14E-08
219091_s_at	multimerin 2	MMRN2	0.20	2.62E-12	0.19	6.87E-19
219093_at	phosphotyrosine interaction domain containing 1	PID1	0.17	9.70E-15	0.31	8.22E-10
219105_x_at	origin recognition complex, subunit 6	ORC6	4.68	2.34E-18	3.57	7.16E-12
219115_s_at	interleukin 20 receptor, alpha	IL20RA	0.30	1.94E-07	0.45	2.25E-04
219117_s_at	FK506 binding protein 11, 19 kDa	FKBP11	2.13	8.63E-07	2.59	9.71E-15
219118_at	FK506 binding protein 11, 19 kDa	FKBP11	2.42	3.31E-07	2.61	4.94E-13
219121_s_at	epithelial splicing regulatory protein 1	ESRP1	7.71	4.18E-15	2.65	3.72E-07
219134_at	EGF, latrophilin and seven transmembrane domain containing 1	ELTD1	0.31	1.59E-11	0.32	3.45E-12
219148_at	PDZ binding kinase	PBK	28.08	2.74E-21	3.77	3.97E-08
219165_at	PDZ and LIM domain 2 (mystique)	PDLIM2	0.22	1.27E-18	0.36	2.53E-14
219167_at	RAS-like, family 12	RASL12	0.22	1.60E-12	0.27	3.98E-13
219213_at	junctional adhesion molecule 2	JAM2	0.11	4.02E-18	0.18	1.66E-15
219215_s_at	solute carrier family 39 (zinc transporter), member 4	SLC39A4	2.90	5.22E-11	2.30	2.69E-08
219228_at	zinc finger protein 331	ZNF331	0.25	4.10E-14	0.30	2.23E-10
219230_at	transmembrane protein 100	TMEM100	0.01	8.40E-18	0.04	7.92E-15
219232_s_at	egl nine homolog 3 (C. elegans)	EGLN3	5.66	5.25E-10	3.25	5.52E-07
219243_at	GTPase, IMAP family member 4	GIMAP4	0.21	2.19E-13	0.31	3.24E-12
219250_s_at	fibronectin leucine rich transmembrane protein 3	FLRT3	0.11	8.94E-15	0.23	1.79E-08

Probeset ID	Gene title	Gene symbol	ESP (GSE18842)		TWN (GSE19804)	
			Fold change	Adjusted P	Fold change	Adjusted P
219274_at	tetraspanin 12	TSPAN12	0.16	1.97E-14	0.33	1.04E-10
219282_s_at	transient receptor potential cation channel, subfamily V, member 2	TRPV2	0.20	2.14E-14	0.32	4.12E-14
219295_s_at	procollagen C-endopeptidase enhancer 2	PCOLCE2	0.11	3.93E-11	0.13	1.09E-12
219298_at	enoyl CoA hydratase domain containing 3	ECHDC3	0.48	3.68E-04	0.47	1.10E-05
219315_s_at	transmembrane protein 204	TMEM204	0.23	1.32E-14	0.30	7.64E-15
219371_s_at	Kruppel-like factor 2 (lung)	KLF2	0.13	1.11E-16	0.22	2.25E-14
219388_at	grainyhead-like 2 (Drosophila)	GRHL2	3.02	1.01E-10	2.38	1.03E-12
219412_at	RAB38, member RAS oncogene family	RAB38	3.73	1.11E-10	2.25	1.84E-07
219427_at	FAT tumor suppressor homolog 4 (Drosophila)	FAT4	0.38	4.44E-08	0.44	6.27E-07
219434_at	triggering receptor expressed on myeloid cells 1	TREM1	0.15	1.72E-10	0.40	6.07E-06
219436_s_at	endomucin	EMCN	0.06	3.98E-19	0.09	1.44E-15
219478_at	WAP four-disulfide core domain 1	WFDC1	0.19	3.59E-12	0.33	1.13E-06
219493_at	SHC SH2-domain binding protein 1	SHCBP1	8.73	5.39E-21	4.53	1.96E-12
219497_s_at	B-cell CLL/lymphoma 11A (zinc finger protein)	BCL11A	13.17	3.02E-12	3.47	2.02E-07
219527_at	mitochondrial amidoxime reducing component 2	MARC2	0.27	3.47E-16	0.41	8.38E-09
219529_at	chloride intracellular channel 3	CLIC3	0.09	1.17E-15	0.18	1.30E-12
219534_x_at	cyclin-dependent kinase inhibitor 1C (p57, Kip2)	CDKN1C	0.41	2.07E-07	0.41	3.40E-10
219544_at	bora, aurora kinase A activator	BORA	5.37	3.86E-18	2.63	1.04E-08
219557_s_at	nuclear receptor interacting protein 3	NRIP3	3.75	5.13E-10	2.60	2.51E-07
219563_at	long intergenic non-protein coding RNA 341	LINC00341	0.20	6.44E-13	0.25	6.95E-15
219568_x_at	SRY (sex determining region Y)-box 18	SOX18	0.24	2.93E-12	0.50	2.03E-06
219584_at	phospholipase A1 member A	PLA1A	0.15	2.30E-14	0.27	1.62E-08
219588_s_at	non-SMC condensin II complex, subunit G2	NCAPG2	5.94	4.42E-17	2.05	9.15E-09
219597_s_at	dual oxidase 1	DUOX1	0.11	9.54E-16	0.11	9.33E-13
219602_s_at	piezo-type mechanosensitive ion channel component 2	PIEZO2	0.30	1.71E-10	0.49	1.11E-06
219654_at	protein tyrosine phosphatase-like (proline instead of catalytic arginine), member A	PTPLA	0.29	2.88E-08	0.25	6.57E-10
219656_at	protocadherin 12	PCDH12	0.35	8.82E-11	0.33	5.79E-18
219660_s_at	ATPase, aminophospholipid transporter, class I, type 8A, member 2	ATP8A2	0.33	7.23E-07	0.46	2.81E-05
219681_s_at	RAB11 family interacting protein 1 (class I)	RAB11FIP1	0.29	7.11E-10	0.39	6.48E-12
219682_s_at	T-box 3	TBX3	0.40	6.63E-06	0.18	1.21E-14
219694_at	family with sequence similarity 105, member A	FAM105A	0.21	1.70E-12	0.43	4.88E-10
219737_s_at	protocadherin 9	PCDH9	0.13	1.07E-13	0.24	5.28E-13
219747_at	neuron-derived neurotrophic factor	NDNF	0.09	1.45E-12	0.31	1.87E-06
219761_at	C-type lectin domain family 1, member A	CLEC1A	0.18	2.52E-16	0.19	3.79E-17
219773_at	NADPH oxidase 4	NOX4	5.11	1.33E-11	2.82	1.33E-07
219778_at	zinc finger protein, multitype 2	ZFPM2	0.37	1.82E-07	0.37	9.98E-08
219787_s_at	epithelial cell transforming sequence 2 oncogene	ECT2	11.34	1.26E-19	3.07	8.71E-10
219788_at	paired immunoglobin-like type 2 receptor alpha	PILRA	0.30	7.88E-11	0.37	1.91E-08
219836_at	zinc finger, BED-type containing 2	ZBED2	0.13	2.04E-10	0.07	1.22E-16
219850_s_at	ets homologous factor	EHF	2.86	3.05E-06	2.34	5.97E-06
219866_at	chloride intracellular channel 5	CLIC5	0.02	1.94E-23	0.06	6.52E-19
219869_s_at	solute carrier family 39 (zinc transporter), member 8	SLC39A8	0.10	9.63E-17	0.18	6.24E-11
219874_at	solute carrier family 12 (potassium/chloride transporters), member 8	SLC12A8	5.58	4.18E-16	3.52	1.60E-08
219892_at	transmembrane 6 superfamily member 1	TM6SF1	0.19	1.65E-15	0.37	4.99E-08
219901_at	FYVE, RhoGEF and PH domain containing 6	FGD6	2.96	1.04E-09	2.04	4.13E-08
219908_at	dickkopf 2 homolog (Xenopus laevis)	DKK2	0.19	1.05E-09	0.16	1.67E-12
219911_s_at	solute carrier organic anion transporter family, member 4A1	SLCO4A1	0.30	8.86E-07	0.26	3.07E-07
219956_at	UDP-N-acetyl-alpha-D-galactosamine:polypeptide N-acetylgalactosaminyltransferase 6 (GalNAc-T6)	GALNT6	2.42	2.50E-05	3.15	3.42E-06

Probeset ID	Gene title	Gene symbol	ESP (GSE18842) Fold change	ESP (GSE18842) Adjusted P	TWN (GSE19804) Fold change	TWN (GSE19804) Adjusted P
219957_at	RUN and FYVE domain containing 2	RUFY2	0.20	1.15E-14	0.45	3.07E-07
219959_at	molybdenum cofactor sulfurase	MOCOS	2.34	2.50E-05	2.18	3.93E-06
219970_at	GIPC PDZ domain containing family, member 2	GIPC2	0.21	1.23E-12	0.28	1.75E-10
219976_at	hook homolog 1 (Drosophila)	HOOK1	2.31	1.69E-07	2.50	7.06E-11
219993_at	SRY (sex determining region Y)-box 17	SOX17	0.09	2.45E-18	0.14	6.45E-18
219995_s_at	zinc finger protein 750	ZNF750	9.29	2.07E-09	3.88	1.58E-07
220005_at	purinergic receptor P2Y, G-protein coupled, 13	P2RY13	0.21	1.74E-11	0.32	2.00E-07
220027_s_at	Ras interacting protein 1	RASIP1	0.14	4.45E-15	0.10	2.76E-21
220037_s_at	lymphatic vessel endothelial hyaluronan receptor 1	LYVE1	0.10	3.78E-15	0.07	1.52E-16
220046_s_at	cyclin L1	CCNL1	0.47	4.03E-11	0.50	3.99E-11
220088_at	complement component 5a receptor 1	C5AR1	0.19	1.46E-14	0.26	8.62E-11
220150_s_at	family with sequence similarity 184, member A	FAM184A	0.17	5.52E-18	0.43	6.22E-06
220170_at	four and a half LIM domains 5	FHL5	0.09	2.83E-22	0.12	5.97E-17
220180_at	coiled-coil domain containing 68	CCDC68	0.18	3.55E-15	0.27	1.49E-10
220187_at	STEAP family member 4	STEAP4	0.20	1.75E-08	0.49	4.52E-03
220244_at	long intergenic non-protein coding RNA 312	LINC00312	0.13	1.67E-16	0.16	2.96E-16
220266_s_at	Kruppel-like factor 4 (gut)	KLF4	0.25	2.37E-07	0.08	9.21E-15
220301_at	coiled-coil domain containing 102B	CCDC102B	0.31	4.90E-10	0.28	8.49E-14
220327_at	vestigial like 3 (Drosophila)	VGLL3	0.12	2.52E-16	0.16	2.98E-14
220615_s_at	fatty acyl CoA reductase 2	FAR2	0.25	7.78E-09	0.41	3.11E-07
220750_s_at	leucine proline-enriched proteoglycan (leprecan) 1	LEPRE1	2.51	1.78E-10	2.25	6.81E-13
220765_s_at	LIM and senescent cell antigen-like domains 2	LIMS2	0.11	2.11E-20	0.18	4.38E-20
220979_s_at	ST6 (alpha-N-acetyl-neuraminyl-2,3-beta-galactosyl-1,3)-N-acetylgalactosaminide alpha-2,6-sialyltransferase 5	ST6GALNAC5	0.23	4.91E-10	0.10	1.14E-14
221011_s_at	limb bud and heart development homolog (mouse)	LBH	0.43	2.25E-09	0.46	1.54E-08
221030_s_at	Rho GTPase activating protein 24	ARHGAP24	0.35	1.11E-15	0.42	9.94E-09
221031_s_at	apolipoprotein L domain containing 1	APOLD1	0.23	3.97E-14	0.22	1.21E-13
221060_s_at	toll-like receptor 4	TLR4	0.30	4.11E-09	0.30	6.82E-08
221087_s_at	apolipoprotein L, 3	APOL3	0.26	9.23E-12	0.35	7.68E-15
221204_s_at	cartilage acidic protein 1	CRTAC1	0.13	4.85E-15	0.14	9.06E-16
221245_s_at	frizzled family receptor 5	FZD5	0.43	3.39E-06	0.49	2.34E-06
221276_s_at	syncoilin, intermediate filament protein	SYNC	0.19	6.64E-16	0.36	1.58E-08
221489_s_at	sprouty homolog 4 (Drosophila)	SPRY4	0.27	3.46E-10	0.48	1.74E-05
221541_at	cysteine-rich secretory protein LCCL domain containing 2	CRISPLD2	0.44	1.76E-05	0.41	1.17E-09
221552_at	abhydrolase domain containing 6	ABHD6	0.40	7.89E-10	0.37	1.03E-12
221558_s_at	lymphoid enhancer-binding factor 1	LEF1	3.66	1.31E-11	2.23	2.65E-08
221610_s_at	signal transducing adaptor family member 2	STAP2	2.70	6.39E-10	2.07	1.12E-11
221667_s_at	heat shock 22kDa protein 8	HSPB8	0.23	8.78E-12	0.27	4.15E-10
221675_s_at	choline phosphotransferase 1	CHPT1	0.40	2.59E-11	0.48	1.12E-10
221677_s_at	downstream neighbor of SON	DONSON	3.51	1.25E-17	2.50	7.36E-11
221696_s_at	serine/threonine/tyrosine kinase 1	STYK1	3.38	4.84E-11	2.45	1.01E-06
221729_at	collagen, type V, alpha 2	COL5A2	7.72	4.70E-16	4.13	1.26E-14
221730_at	collagen, type V, alpha 2	COL5A2	7.41	4.85E-15	3.46	7.74E-10
221731_x_at	versican	VCAN	2.53	6.21E-07	2.17	3.25E-07
221747_at	tensin 1	TNS1	0.16	1.85E-17	0.26	1.19E-16
221748_s_at	tensin 1	TNS1	0.18	2.59E-18	0.35	1.00E-13
221841_s_at	Kruppel-like factor 4 (gut)	KLF4	0.27	3.73E-09	0.15	1.81E-14
221867_at	NEDD4 binding protein 1	N4BP1	0.44	3.15E-11	0.49	5.69E-13
221870_at	EH-domain containing 2	EHD2	0.44	6.19E-09	0.45	9.98E-12
221935_s_at	EGF domain-specific O-linked N-acetylglucosamine (GlcNAc) transferase	EOGT	0.50	1.44E-11	0.43	1.66E-12

Probeset ID	Gene title	Gene symbol	ESP (GSE18842) Fold change	Adjusted P	TWN (GSE19804) Fold change	Adjusted P
222036_s_at	minichromosome maintenance complex component 4	MCM4	7.50	1.19E-20	2.67	4.06E-12
222039_at	kinesin family member 18B	KIF18B	8.77	1.40E-21	3.60	6.17E-09
222043_at	clusterin	CLU	0.14	1.95E-15	0.22	4.21E-12
222067_x_at	histone cluster 1, H2bd	HIST1H2B D	3.92	1.12E-13	3.17	2.56E-14
222071_s_at	solute carrier organic anion transporter family, member 4C1	SLCO4C1	0.26	1.61E-08	0.41	4.69E-04
222073_at	collagen, type IV, alpha 3 (Goodpasture antigen)	COL4A3	0.03	6.39E-20	0.27	6.53E-07
222121_at	Rho guanine nucleotide exchange factor (GEF) 26	ARHGEF26	0.25	2.16E-11	0.08	1.92E-19
222155_s_at	solute carrier family 52, riboflavin transporter, member 2	SLC52A2	3.33	5.05E-16	2.38	1.67E-11
222162_s_at	ADAM metallopeptidase with thrombospondin type 1 motif, 1	ADAMTS1	0.23	2.05E-11	0.17	1.05E-12
222218_s_at	paired immunoglobin-like type 2 receptor alpha	PILRA	0.23	1.30E-13	0.38	1.43E-09
222221_x_at	EH-domain containing 1	EHD1	0.48	4.08E-10	0.48	7.09E-14
222317_at	phosphodiesterase 3B, cGMP-inhibited	PDE3B	0.36	1.34E-14	0.27	2.91E-14
222416_at	aldehyde dehydrogenase 18 family, member A1	ALDH18A1	3.28	5.61E-18	2.53	6.25E-20
222453_at	cytochrome b reductase 1	CYBRD1	0.22	1.00E-18	0.35	1.85E-11
222484_s_at	chemokine (C-X-C motif) ligand 14	CXCL14	19.93	6.31E-10	9.18	1.80E-13
222486_s_at	ADAM metallopeptidase with thrombospondin type 1 motif, 1	ADAMTS1	0.31	1.17E-06	0.15	8.51E-13
222520_s_at	intraflagellar transport 57 homolog (Chlamydomonas)	IFT57	0.11	3.29E-23	0.33	9.02E-09
222532_at	signal recognition particle receptor, B subunit	SRPRB	3.09	2.16E-14	2.04	2.12E-13
222549_at	claudin 1	CLDN1	5.15	2.68E-06	3.32	7.00E-08
222571_at	ST6 (alpha-N-acetyl-neuraminyl-2,3-beta-galactosyl-1,3)-N-acetylgalactosaminide alpha-2,6-sialyltransferase 6	ST6GALNA C6	0.33	3.84E-18	0.47	1.37E-12
222581_at	xenotropic and polytropic retrovirus receptor 1	XPR1	3.21	2.32E-12	3.19	3.35E-16
222587_s_at	UDP-N-acetyl-alpha-D-galactosamine:polypeptide N-acetylgalactosaminyltransferase 7 (GalNAc-T7)	GALNT7	3.23	4.95E-13	4.33	2.10E-15
222608_s_at	anillin, actin binding protein	ANLN	44.29	4.28E-26	14.05	2.23E-15
222646_s_at	ERO1-like (S. cerevisiae)	ERO1L	7.31	6.09E-18	3.99	3.38E-14
222717_at	serum deprivation response	SDPR	0.06	7.08E-19	0.09	6.92E-17
222722_at	osteoglycin	OGN	0.06	9.08E-16	0.12	2.91E-11
222740_at	ATPase family, AAA domain containing 2	ATAD2	7.05	6.16E-18	2.29	7.59E-11
222750_s_at	steroid 5 alpha-reductase 3	SRD5A3	2.61	3.10E-08	3.38	8.24E-15
222762_x_at	LIM domains containing 1	LIMD1	0.29	5.20E-17	0.46	8.30E-14
222774_s_at	neuropilin (NRP) and tolloid (TLL)-like 2	NETO2	7.10	4.44E-15	2.02	3.98E-05
222802_at	endothelin 1	EDN1	0.18	8.07E-11	0.18	2.42E-15
222830_at	grainyhead-like 1 (Drosophila)	GRHL1	6.71	1.16E-10	2.24	6.82E-06
222843_at	fidgetin-like 1	FIGNL1	5.01	5.22E-19	3.21	3.73E-10
222848_at	centromere protein K	CENPK	5.85	3.26E-19	3.28	5.51E-10
222853_at	fibronectin leucine rich transmembrane protein 3	FLRT3	0.08	4.99E-14	0.26	2.05E-07
222885_at	endomucin	EMCN	0.06	6.46E-20	0.12	5.51E-17
222891_s_at	B-cell CLL/lymphoma 11A (zinc finger protein)	BCL11A	8.20	1.39E-10	3.40	1.11E-07
222899_at	integrin, alpha 11	ITGA11	3.65	2.36E-10	3.36	1.47E-09
222912_at	arrestin, beta 1	ARRB1	0.11	5.35E-15	0.37	1.33E-13
222931_s_at	threonine synthase-like 1 (S. cerevisiae)	THNSL1	3.72	2.55E-10	2.81	5.00E-12
222934_s_at	C-type lectin domain family 4, member E	CLEC4E	0.26	1.19E-09	0.16	1.35E-12
223062_s_at	phosphoserine aminotransferase 1	PSAT1	41.43	2.02E-20	10.85	1.21E-17
223075_s_at	allograft inflammatory factor 1-like	AIF1L	0.25	2.52E-10	0.27	1.60E-10
223121_s_at	secreted frizzled-related protein 2	SFRP2	3.72	1.24E-05	2.43	6.01E-03
223122_s_at	secreted frizzled-related protein 2	SFRP2	2.27	3.25E-03	2.67	3.40E-03
223172_s_at	mitochondrial fission process 1	MTFP1	2.66	6.41E-12	2.25	2.63E-12

Probeset ID	Gene title	Gene symbol	ESP (GSE18842) Fold change	ESP (GSE18842) Adjusted P	TWN (GSE19804) Fold change	TWN (GSE19804) Adjusted P
223217_s_at	nuclear factor of kappa light polypeptide gene enhancer in B-cells inhibitor, zeta	NFKBIZ	0.22	1.83E-11	0.25	2.22E-10
223218_s_at	nuclear factor of kappa light polypeptide gene enhancer in B-cells inhibitor, zeta	NFKBIZ	0.22	1.11E-13	0.35	1.63E-10
223229_at	ubiquitin-conjugating enzyme E2T (putative)	UBE2T	21.55	7.08E-25	6.63	2.16E-15
223245_at	spermatid perinuclear RNA binding protein	STRBP	2.40	4.49E-09	2.20	2.44E-13
223246_s_at	spermatid perinuclear RNA binding protein	STRBP	3.29	8.18E-10	2.31	3.74E-13
223315_at	netrin 4	NTN4	0.14	3.94E-13	0.38	6.91E-08
223343_at	membrane-spanning 4-domains, subfamily A, member 7	MS4A7	0.10	3.28E-16	0.42	2.96E-08
223344_s_at	membrane-spanning 4-domains, subfamily A, member 7	MS4A7	0.09	3.12E-16	0.32	3.64E-08
223363_at	proteasome (prosome, macropain) assembly chaperone 3	PSMG3	2.44	1.92E-16	2.31	1.73E-14
223380_s_at	LATS, large tumor suppressor, homolog 2 (Drosophila)	LATS2	0.36	1.13E-15	0.49	2.22E-12
223394_at	SERTA domain containing 1	SERTAD1	0.25	1.04E-13	0.37	7.63E-14
223395_at	ABI family, member 3 (NESH) binding protein	ABI3BP	0.07	2.14E-16	0.16	3.24E-13
223449_at	sema domain, transmembrane domain (TM), and cytoplasmic domain, (semaphorin) 6A	SEMA6A	0.42	1.08E-06	0.12	6.25E-20
223452_s_at	atlastin GTPase 3	ATL3	2.82	6.21E-11	2.17	1.20E-08
223484_at	chromosome 15 open reading frame 48	C15orf48	5.69	2.36E-09	2.83	3.72E-07
223492_s_at	leucine rich repeat (in FLII) interacting protein 1	LRRFIP1	0.18	6.94E-19	0.28	5.46E-16
223504_at	DnaJ (Hsp40) homolog, subfamily C, member 27	DNAJC27	0.44	9.02E-13	0.43	7.86E-13
223523_at	transmembrane protein 108	TMEM108	0.22	2.90E-11	0.40	2.51E-07
223623_at	chromosome 2 open reading frame 40	C2orf40	0.03	2.15E-19	0.09	4.16E-13
223805_at	oxysterol binding protein-like 6	OSBPL6	0.50	5.54E-05	0.43	5.07E-06
223809_at	regulator of G-protein signaling 18	RGS18	0.19	2.96E-13	0.25	6.99E-10
223821_s_at	sushi domain containing 4	SUSD4	7.54	1.50E-10	3.82	4.48E-11
224013_s_at	SRY (sex determining region Y)-box 7	SOX7	0.15	3.99E-10	0.07	3.33E-19
224061_at	indolethylamine N-methyltransferase	INMT	0.03	4.62E-23	0.09	1.95E-14
224189_x_at	ets homologous factor	EHF	2.97	2.97E-07	2.04	1.12E-05
224341_x_at	toll-like receptor 4	TLR4	0.26	4.20E-12	0.31	6.06E-13
224342_x_at	BMS1 homolog, ribosome assembly protein (yeast) pseudogene	LOC96610	2.37	8.92E-03	2.24	2.86E-03
224352_s_at	cofilin 2 (muscle)	CFL2	0.25	2.51E-18	0.31	2.42E-17
224358_s_at	membrane-spanning 4-domains, subfamily A, member 7	MS4A7	0.10	4.21E-17	0.28	1.11E-10
224428_s_at	cell division cycle associated 7	CDCA7	21.81	1.54E-21	11.41	1.66E-15
224448_s_at	mitochondrial nucleoid factor 1	MNF1	2.80	1.28E-13	3.07	3.79E-19
224467_s_at	programmed cell death 2-like	PDCD2L	2.70	1.09E-12	2.21	1.72E-11
224480_s_at	1-acylglycerol-3-phosphate O-acyltransferase 9	AGPAT9	0.41	5.56E-06	0.46	7.41E-08
224511_s_at	thioredoxin domain containing 17	TXNDC17	2.52	1.68E-13	2.61	3.88E-17
224606_at	Kruppel-like factor 6	KLF6	0.25	1.15E-17	0.35	1.09E-14
224663_s_at	cofilin 2 (muscle)	CFL2	0.40	1.89E-15	0.40	6.03E-13
224674_at	tweety homolog 3 (Drosophila)	TTYH3	2.58	1.50E-12	2.51	3.31E-09
224780_at	RNA binding motif protein 17	RBM17	0.30	3.22E-12	0.45	9.18E-16
224822_at	deleted in liver cancer 1	DLC1	0.08	2.47E-19	0.19	8.43E-15
224833_at	v-ets erythroblastosis virus E26 oncogene homolog 1 (avian)	ETS1	0.42	2.55E-09	0.42	2.93E-15
224861_at	guanine nucleotide binding protein (G protein), q polypeptide	GNAQ	0.26	1.80E-17	0.44	1.17E-16
224862_at	guanine nucleotide binding protein (G protein), q polypeptide	GNAQ	0.34	1.24E-15	0.49	2.20E-15
224909_s_at	phosphatidylinositol-3,4,5-trisphosphate-dependent Rac exchange factor 1	PREX1	0.36	6.07E-13	0.34	2.19E-13
224920_x_at	myeloid-associated differentiation marker	MYADM	0.21	2.21E-12	0.27	1.10E-14
224925_at	phosphatidylinositol-3,4,5-trisphosphate-dependent Rac exchange factor 1	PREX1	0.29	2.10E-15	0.32	3.34E-14

Probeset ID	Gene title	Gene symbol	ESP (GSE18842) Fold change	Adjusted P	TWN (GSE19804) Fold change	Adjusted P
224937_at	prostaglandin F2 receptor negative regulator	PTGFRN	5.69	6.53E-15	3.19	1.48E-15
224950_at	prostaglandin F2 receptor negative regulator	PTGFRN	6.78	2.69E-16	3.68	3.60E-15
225078_at	epithelial membrane protein 2	EMP2	0.14	2.32E-17	0.21	1.00E-16
225079_at	epithelial membrane protein 2	EMP2	0.19	6.92E-15	0.21	2.03E-15
225093_at	utrophin	UTRN	0.31	1.09E-16	0.49	2.03E-15
225144_at	bone morphogenetic protein receptor, type II (serine/threonine kinase)	BMPR2	0.47	2.51E-10	0.40	5.27E-11
225162_at	SH3 domain containing 19	SH3D19	0.38	4.57E-16	0.45	1.87E-09
225163_at	FERM domain containing 4A	FRMD4A	0.35	1.05E-11	0.33	3.37E-10
225168_at	FERM domain containing 4A	FRMD4A	0.45	2.17E-10	0.46	4.49E-11
225177_at	RAB11 family interacting protein 1 (class I)	RAB11FIP1	0.18	3.58E-13	0.32	2.08E-14
225185_at	muscle RAS oncogene homolog	MRAS	0.38	8.27E-11	0.50	1.53E-08
225200_at	DPH3, KTI11 homolog (S. cerevisiae)	DPH3	0.35	2.65E-15	0.48	2.82E-09
225207_at	pyruvate dehydrogenase kinase, isozyme 4	PDK4	0.05	8.20E-17	0.10	2.87E-16
225212_at	solute carrier family 25 (mitochondrial carrier; phosphate carrier), member 25	SLC25A25	0.39	1.98E-08	0.29	3.87E-16
225237_s_at	musashi homolog 2 (Drosophila)	MSI2	4.42	7.25E-14	3.02	6.01E-12
225240_s_at	musashi homolog 2 (Drosophila)	MSI2	2.69	4.24E-12	2.58	2.73E-18
225299_at	myosin VB	MYO5B	0.33	1.91E-08	0.35	1.19E-08
225314_at	OCIA domain containing 2	OCIAD2	3.47	6.67E-13	4.84	2.65E-20
225316_at	major facilitator superfamily domain containing 2A	MFSD2A	0.26	1.12E-10	0.47	6.40E-05
225328_at	F-box protein 32	FBXO32	7.06	1.92E-16	3.47	7.92E-10
225369_at	endothelial cell adhesion molecule	ESAM	0.14	6.63E-19	0.20	9.87E-18
225373_at	chromosome 10 open reading frame 54	C10orf54	0.32	7.07E-13	0.37	9.87E-18
225380_at	protein kinase domain containing, cytoplasmic homolog (mouse)	PKDCC	0.30	4.30E-08	0.32	2.02E-11
225387_at	tetraspanin 5	TSPAN5	3.41	3.16E-10	2.12	2.54E-09
225426_at	protein phosphatase 6, catalytic subunit	PPP6C	0.49	2.20E-12	0.49	2.07E-13
225474_at	membrane associated guanylate kinase, WW and PDZ domain containing 1	MAGI1	0.26	2.67E-14	0.48	2.55E-07
225485_at	centrosomal protein 41kDa	CEP41	3.81	5.89E-13	2.43	1.90E-10
225504_at	homeobox containing 1	HMBOX1	0.46	1.56E-11	0.37	3.63E-16
225536_at	transmembrane protein 54	TMEM54	2.56	8.17E-10	2.20	3.45E-08
225540_at	microtubule-associated protein 2	MAP2	0.25	6.27E-08	0.30	1.30E-10
225557_at	cysteine-serine-rich nuclear protein 1	CSRNP1	0.10	4.22E-16	0.17	6.76E-16
225571_at	leukemia inhibitory factor receptor alpha	LIFR	0.11	2.59E-16	0.19	4.79E-13
225575_at	leukemia inhibitory factor receptor alpha	LIFR	0.13	7.53E-16	0.25	3.29E-14
225645_at	ets homologous factor	EHF	2.11	4.16E-04	2.32	9.83E-07
225666_at	transmembrane and tetratricopeptide repeat containing 4	TMTC4	2.17	3.60E-09	2.46	1.57E-12
225673_at	myeloid-associated differentiation marker	MYADM	0.22	1.48E-12	0.33	5.59E-14
225681_at	collagen triple helix repeat containing 1	CTHRC1	16.84	1.86E-18	15.09	4.80E-18
225687_at	family with sequence similarity 83, member D	FAM83D	24.65	6.23E-19	3.02	1.24E-07
225720_at	synaptopodin 2	SYNPO2	0.12	8.98E-17	0.16	2.42E-13
225721_at	synaptopodin 2	SYNPO2	0.25	2.33E-15	0.34	2.30E-10
225723_at	coiled-coil domain containing 167	CCDC167	2.47	6.08E-13	2.16	4.49E-13
225750_at	ERO1-like (S. cerevisiae)	ERO1L	3.35	4.79E-14	2.55	4.16E-15
225763_at	RCSD domain containing 1	RCSD1	0.22	3.00E-13	0.35	2.45E-08
225765_at	transportin 1	TNPO1	3.84	4.82E-17	2.78	2.53E-19
225766_s_at	transportin 1	TNPO1	4.08	1.26E-17	2.45	1.45E-09
225776_at	RNA binding motif, single stranded interacting protein 2	RBMS2	0.26	1.40E-15	0.36	3.47E-14
225778_at	RNA binding motif, single stranded interacting protein 2	RBMS2	0.41	1.08E-16	0.41	1.45E-15
225782_at	methionine sulfoxide reductase B3	MSRB3	0.30	1.11E-10	0.33	7.28E-10
225790_at	methionine sulfoxide reductase B3	MSRB3	0.33	8.45E-09	0.26	3.28E-10

Probeset ID	Gene title	Gene symbol	ESP (GSE18842) Fold change	ESP (GSE18842) Adjusted P	TWN (GSE19804) Fold change	TWN (GSE19804) Adjusted P
225792_at	hook homolog 1 (Drosophila)	HOOK1	2.51	6.31E-06	2.39	4.64E-10
225816_at	PHD finger protein 17	PHF17	0.27	1.94E-10	0.48	2.67E-12
225817_at	cingulin-like 1	CGNL1	0.12	5.55E-16	0.26	6.06E-12
225820_at	PHD finger protein 17	PHF17	0.35	2.38E-08	0.38	4.53E-13
225836_s_at	chromosome 12 open reading frame 32	C12orf32	3.01	1.60E-14	2.62	3.54E-14
225837_at	chromosome 12 open reading frame 32	C12orf32	2.52	5.87E-11	2.37	5.11E-14
225846_at	epithelial splicing regulatory protein 1	ESRP1	3.09	8.53E-11	2.26	3.24E-07
225855_at	erythrocyte membrane protein band 4.1 like 5	EPB41L5	0.12	2.58E-21	0.20	8.80E-13
225895_at	synaptopodin 2	SYNPO2	0.17	2.26E-12	0.26	9.44E-11
225911_at	nephronectin	NPNT	0.18	1.12E-11	0.25	5.63E-10
225913_at	NKF3 kinase family member	PEAK1	0.29	9.13E-17	0.38	2.42E-14
225914_s_at	calcium binding protein 39-like	CAB39L	0.27	2.52E-16	0.33	2.06E-13
225939_at	eukaryotic translation initiation factor 4E family member 3	EIF4E3	0.30	2.99E-17	0.36	5.24E-15
225943_at	neurolysin (metallopeptidase M3 family)	NLN	3.91	7.89E-16	3.19	3.53E-14
225944_at	neurolysin (metallopeptidase M3 family)	NLN	2.89	2.15E-14	3.28	1.98E-15
225946_at	Ras association (RalGDS/AF-6) domain family (N-terminal) member 8	RASSF8	0.32	9.98E-09	0.30	3.88E-14
225947_at	myosin XIX	MYO19	2.68	1.21E-13	2.12	1.95E-12
225968_at	prickle homolog 2 (Drosophila)	PRICKLE2	0.39	3.20E-10	0.31	4.81E-12
226016_at	CD47 molecule	CD47	0.25	1.00E-14	0.44	4.24E-11
226018_at	chromosome 7 open reading frame 41	C7orf41	0.14	2.28E-18	0.25	8.79E-13
226022_at	SAM and SH3 domain containing 1	SASH1	0.29	4.13E-14	0.25	2.11E-17
226028_at	roundabout, axon guidance receptor, homolog 4 (Drosophila)	ROBO4	0.10	5.86E-20	0.11	4.16E-20
226056_at	Rho GTPase activating protein 31	ARHGAP31	0.21	4.38E-19	0.35	2.23E-16
226065_at	prickle homolog 1 (Drosophila)	PRICKLE1	0.46	3.36E-05	0.49	3.41E-05
226071_at	ADAMTS-like 4	ADAMTSL4	0.12	6.92E-18	0.28	3.99E-12
226103_at	nexilin (F actin binding protein)	NEXN	0.20	1.12E-12	0.32	2.51E-08
226121_at	dehydrogenase/reductase (SDR family) member 13	DHRS13	3.12	8.69E-13	2.08	1.43E-10
226140_s_at	OTU domain containing 1	OTUD1	0.30	8.43E-17	0.30	3.04E-18
226145_s_at	Fraser syndrome 1	FRAS1	0.41	6.30E-04	0.15	2.17E-13
226150_at	phosphatidic acid phosphatase type 2 domain containing 1B	PPAPDC1B	2.42	7.92E-07	2.41	6.62E-15
226171_at	zinc finger, DHHC-type containing 3	ZDHHC3	0.46	2.71E-08	0.41	4.79E-13
226188_at	lectin, galactoside-binding-like	LGALSL	0.38	1.04E-08	0.40	4.58E-12
226198_at	target of myb1-like 2 (chicken)	TOM1L2	0.39	3.84E-13	0.45	3.78E-16
226219_at	Rho GTPase activating protein 30	ARHGAP30	0.24	7.66E-12	0.41	8.89E-09
226226_at	transmembrane protein 45B	TMEM45B	2.17	1.40E-03	2.88	1.85E-07
226228_at	aquaporin 4	AQP4	0.01	2.30E-15	0.08	1.25E-08
226244_at	C-type lectin domain family 14, member A	CLEC14A	0.14	1.29E-17	0.18	2.09E-18
226245_at	potassium channel tetramerisation domain containing 1	KCTD1	5.85	1.07E-10	2.58	5.83E-09
226267_at	Jun dimerization protein 2	JDP2	0.33	2.88E-15	0.41	2.43E-14
226280_at	BCL2/adenovirus E1B 19kDa interacting protein 2	BNIP2	0.44	4.22E-12	0.32	5.84E-15
226287_at	coiled-coil domain containing 34	CCDC34	7.90	3.42E-20	2.89	1.04E-12
226303_at	phosphoglucomutase 5	PGM5	0.08	4.91E-20	0.17	3.82E-14
226380_at	protein tyrosine phosphatase, non-receptor type 21	PTPN21	0.09	1.07E-19	0.18	4.31E-17
226452_at	pyruvate dehydrogenase kinase, isozyme 1	PDK1	4.23	1.23E-14	2.40	1.12E-11
226485_at	V-set and immunoglobulin domain containing 10	VSIG10	0.24	1.20E-13	0.43	3.89E-07
226490_at	NHS-like 1	NHSL1	0.38	2.15E-08	0.28	5.20E-11
226565_at	transmembrane protein 99	TMEM99	2.85	4.51E-12	2.71	3.07E-13

Probeset ID	Gene title	Gene symbol	ESP (GSE18842) Fold change	ESP (GSE18842) Adjusted P	TWN (GSE19804) Fold change	TWN (GSE19804) Adjusted P
226607_at	chromosome 20 open reading frame 194	C20orf194	0.40	1.67E-13	0.43	2.68E-14
226614_s_at	family with sequence similarity 167, member A	FAM167A	0.17	4.81E-13	0.19	8.40E-16
226615_at	xenotropic and polytropic retrovirus receptor 1	XPR1	2.15	7.13E-08	2.10	1.56E-08
226622_at	mucin 20, cell surface associated	MUC20	3.11	8.80E-05	6.17	3.53E-13
226625_at	transforming growth factor, beta receptor III	TGFBR3	0.18	1.57E-12	0.15	4.65E-17
226627_at	septin 8	SEPT8	0.11	3.55E-21	0.26	6.25E-15
226633_at	RAB8B, member RAS oncogene family	RAB8B	0.38	7.45E-14	0.44	3.82E-14
226641_at	ankyrin repeat domain 44	ANKRD44	0.23	4.44E-13	0.47	7.03E-10
226673_at	SH2 domain containing 3C	SH2D3C	0.17	1.94E-17	0.19	9.87E-18
226701_at	gap junction protein, alpha 5, 40kDa	GJA5	0.29	1.82E-09	0.35	2.07E-08
226713_at	coiled-coil domain containing 50	CCDC50	0.20	5.29E-15	0.25	1.50E-14
226743_at	schlafen family member 11	SLFN11	0.32	2.55E-09	0.48	9.33E-07
226751_at	cannabinoid receptor interacting protein 1	CNRIP1	0.21	2.28E-15	0.34	2.82E-12
226766_at	roundabout, axon guidance receptor, homolog 2 (Drosophila)	ROBO2	0.21	8.71E-11	0.32	1.88E-06
226769_at	fin bud initiation factor homolog (zebrafish)	FIBIN	0.23	4.26E-07	0.15	5.58E-13
226777_at	ADAM metallopeptidase domain 12	ADAM12	26.85	3.03E-19	8.08	6.53E-10
226795_at	leucine-rich repeats and calponin homology (CH) domain containing 1	LRCH1	0.41	2.29E-13	0.42	2.82E-15
226818_at	macrophage expressed 1	MPEG1	0.41	4.90E-06	0.49	3.25E-06
226828_s_at	hairy/enhancer-of-split related with YRPW motif-like	HEYL	0.32	1.98E-11	0.38	1.86E-10
226872_at	regulatory factor X, 2 (influences HLA class II expression)	RFX2	0.19	4.84E-13	0.22	5.26E-11
226905_at	family with sequence similarity 101, member B	FAM101B	0.34	3.75E-11	0.35	3.34E-11
226908_at	leucine-rich repeats and immunoglobulin-like domains 3	LRIG3	3.15	5.75E-12	2.92	2.22E-10
226933_s_at	Inhibitor of DNA binding 4, dominant negative helix-loop-helix protein	ID4	0.26	1.64E-10	0.28	7.71E-13
226936_at	centromere protein W	CENPW	6.60	5.11E-15	2.19	1.88E-06
226950_at	activin A receptor type II-like 1	ACVRL1	0.16	1.24E-14	0.12	3.81E-20
226955_at	actin filament associated protein 1-like 1	AFAP1L1	0.27	7.03E-11	0.14	2.31E-16
226974_at	neural precursor cell expressed, developmentally down-regulated 4-like, E3 ubiquitin protein ligase	NEDD4L	0.14	1.71E-16	0.17	1.37E-12
226984_at	FYVE, RhoGEF and PH domain containing 5	FGD5	0.26	4.52E-15	0.26	2.52E-18
226985_at	FYVE, RhoGEF and PH domain containing 5	FGD5	0.15	1.11E-18	0.19	2.64E-17
226997_at	ADAM metallopeptidase with thrombospondin type 1 motif, 12	ADAMTS12	7.60	2.86E-16	3.05	2.73E-10
227006_at	protein phosphatase 1, regulatory (inhibitor) subunit 14A	PPP1R14A	0.11	5.98E-19	0.21	7.45E-13
227013_at	LATS, large tumor suppressor, homolog 2 (Drosophila)	LATS2	0.30	1.38E-14	0.38	3.08E-13
227014_at	aspartate beta-hydroxylase domain containing 2	ASPHD2	2.80	1.75E-08	2.13	3.11E-05
227046_at	solute carrier family 39 (metal ion transporter), member 11	SLC39A11	2.41	1.14E-10	2.68	3.53E-14
227084_at	dystrobrevin, alpha	DTNA	0.46	5.49E-06	0.49	1.09E-04
227088_at	phosphodiesterase 5A, cGMP-specific	PDE5A	0.20	1.88E-16	0.22	6.65E-13
227094_at	dehydrogenase E1 and transketolase domain containing 1	DHTKD1	2.05	1.26E-08	2.33	2.88E-17
227099_s_at	chromosome 11 open reading frame 96	C11orf96	0.19	1.35E-11	0.33	6.55E-14
227126_at	protein tyrosine phosphatase, receptor type, G	PTPRG	0.38	1.41E-11	0.49	3.54E-07
227131_at	mitogen-activated protein kinase kinase kinase 3	MAP3K3	0.34	1.63E-22	0.46	3.35E-15
227139_s_at	Hermansky-Pudlak syndrome 3	HPS3	2.00	2.44E-12	2.08	1.58E-14
227148_at	pleckstrin homology domain containing, family H (with MyTH4 domain) member 2	PLEKHH2	0.09	1.23E-19	0.21	1.19E-13
227174_at	WD repeat domain 72	WDR72	37.46	2.89E-12	3.95	4.13E-06
227178_at	CUGBP, Elav-like family member 2	CELF2	0.13	3.57E-20	0.22	7.82E-17
227197_at	Rho guanine nucleotide exchange factor (GEF) 26	ARHGEF26	0.21	1.71E-13	0.13	1.27E-14

Probeset ID	Gene title	Gene symbol	ESP (GSE18842) Fold change	ESP (GSE18842) Adjusted P	TWN (GSE19804) Fold change	TWN (GSE19804) Adjusted P
227198_at	AF4/FMR2 family, member 3	AFF3	0.05	1.59E-18	0.10	1.44E-14
227212_s_at	PHD finger protein 19	PHF19	2.64	1.27E-12	2.11	1.04E-09
227224_at	Ral GEF with PH domain and SH3 binding motif 2	RALGPS2	3.97	3.49E-17	2.51	1.15E-09
227230_s_at	KIAA1211	KIAA1211	2.45	3.28E-08	2.75	1.79E-06
227236_at	tetraspanin 2	TSPAN2	0.36	1.82E-06	0.42	3.62E-07
227247_at	pleckstrin homology domain containing, family A (phosphoinositide binding specific) member 8	PLEKHA8	2.65	2.42E-14	2.59	1.05E-11
227253_at	ceruloplasmin (ferroxidase)	CP	4.46	2.88E-05	8.33	5.40E-10
227265_at	fibrinogen-like 2	FGL2	0.29	7.73E-08	0.44	3.02E-08
227289_at	protocadherin 17	PCDH17	0.31	9.56E-11	0.22	1.91E-13
227329_at	zinc finger and BTB domain containing 46	ZBTB46	0.44	3.44E-12	0.45	2.16E-08
227341_at	BEN domain containing 7	BEND7	0.26	3.61E-11	0.42	4.40E-08
227345_at	tumor necrosis factor receptor superfamily, member 10d, decoy with truncated death domain	TNFRSF10D	0.32	3.00E-09	0.30	1.21E-10
227350_at	helicase, lymphoid-specific	HELLS	5.49	5.87E-16	2.57	2.25E-09
227371_at	BAI1-associated protein 2-like 1	BAIAP2L1	5.74	2.52E-16	2.79	3.30E-12
227372_s_at	BAI1-associated protein 2-like 1	BAIAP2L1	5.17	1.03E-17	2.97	1.80E-12
227399_at	vestigial like 3 (Drosophila)	VGLL3	0.25	7.79E-12	0.31	3.53E-10
227417_at	mitochondrial amidoxime reducing component 2	MARC2	0.10	3.26E-19	0.34	2.60E-08
227419_x_at	placenta-specific 9	PLAC9	0.06	1.50E-21	0.13	1.48E-16
227448_at	arginine and glutamate rich 1	ARGLU1	0.18	5.78E-16	0.26	1.20E-15
227458_at	CD274 molecule	CD274	0.35	4.16E-04	0.18	7.88E-11
227463_at	angiotensin I converting enzyme (peptidyl-dipeptidase A) 1	ACE	0.23	1.23E-14	0.20	1.00E-17
227480_at	sushi domain containing 2	SUSD2	0.03	2.40E-16	0.27	4.54E-05
227529_s_at	A kinase (PRKA) anchor protein 12	AKAP12	0.17	9.28E-12	0.09	1.19E-15
227530_at	A kinase (PRKA) anchor protein 12	AKAP12	0.16	2.20E-11	0.13	2.52E-14
227559_at	NADH dehydrogenase (ubiquinone) complex I, assembly factor 4	NDUFAF4	0.49	3.62E-07	0.45	5.19E-13
227613_at	zinc finger protein 331	ZNF331	0.19	3.63E-17	0.23	5.76E-13
227628_at	glutathione peroxidase 8 (putative)	GPX8	4.66	1.87E-15	3.24	3.67E-09
227645_at	phosphoinositide-3-kinase, regulatory subunit 5	PIK3R5	0.19	2.15E-14	0.39	9.21E-10
227646_at	early B-cell factor 1	EBF1	0.40	7.75E-07	0.29	1.10E-10
227662_at	synaptopodin 2	SYNPO2	0.12	1.97E-13	0.20	6.99E-10
227687_at	hydrolethalus syndrome 1	HYLS1	2.83	6.98E-11	2.13	6.77E-10
227697_at	suppressor of cytokine signaling 3	SOCS3	0.24	1.43E-12	0.22	1.22E-12
227719_at	SMAD family member 9	SMAD9	0.13	1.12E-12	0.23	3.83E-13
227721_at	C3 and PZP-like, alpha-2-macroglobulin domain containing 8	CPAMD8	0.14	8.77E-21	0.47	1.07E-06
227727_at	MAS-related GPR, member F	MRGPRF	0.42	2.52E-06	0.42	1.49E-06
227758_at	RAS-like, estrogen-regulated, growth inhibitor	RERG	0.20	1.31E-13	0.41	2.27E-07
227771_at	leukemia inhibitory factor receptor alpha	LIFR	0.20	1.28E-12	0.27	3.35E-12
227780_s_at	endothelial cell surface expressed chemotaxis and apoptosis regulator	ECSCR	0.10	8.63E-22	0.15	4.94E-18
227801_at	tripartite motif containing 59	TRIM59	8.41	1.12E-17	3.39	7.97E-12
227804_at	TLC domain containing 1	TLCD1	3.22	1.78E-10	3.81	3.73E-12
227817_at	protein kinase C, beta	PRKCB	0.41	7.08E-11	0.40	6.65E-10
227848_at	phosphatidylethanolamine-binding protein 4	PEBP4	0.02	3.53E-20	0.08	7.77E-11
227856_at	chromosome 4 open reading frame 32	C4orf32	0.29	6.38E-12	0.44	1.80E-09
227859_at	DnaJ (Hsp40) homolog, subfamily C, member 27	DNAJC27	0.42	1.58E-10	0.33	6.69E-18
227874_at	endomucin	EMCN	0.04	7.67E-22	0.06	1.05E-17
227923_at	SH3 and multiple ankyrin repeat domains 3	SHANK3	0.23	2.99E-18	0.15	5.05E-18
227929_at	lin-7 homolog A (C. elegans)	LIN7A	0.15	3.24E-15	0.08	5.43E-20
227945_at	TBC1 (tre-2/USP6, BUB2, cdc16) domain family, member 1	TBC1D1	0.29	2.07E-16	0.42	1.15E-11
227947_at	phosphatase and actin regulator 2	PHACTR2	0.27	7.75E-18	0.39	6.85E-15

Probeset ID	Gene title	Gene symbol	ESP (GSE18842) Fold change	Adjusted P	TWN (GSE19804) Fold change	Adjusted P
227948_at	FYVE, RhoGEF and PH domain containing 4	FGD4	0.22	1.63E-17	0.49	5.46E-08
227949_at	phosphatase and actin regulator 3	PHACTR3	0.35	3.85E-04	0.31	8.68E-05
227983_at	Rab interacting lysosomal protein-like 2	RILPL2	0.32	1.01E-18	0.46	5.75E-15
228007_at	centrosomal protein 85kDa-like	CEP85L	0.43	2.07E-11	0.49	2.47E-07
228038_at	SRY (sex determining region Y)-box 2	SOX2	51.87	2.35E-13	3.30	8.32E-05
228042_at	ADP-ribosylarginine hydrolase	ADPRH	0.27	1.92E-18	0.38	1.51E-14
228062_at	nucleosome assembly protein 1-like 5	NAP1L5	0.37	1.84E-09	0.33	3.78E-12
228063_s_at	nucleosome assembly protein 1-like 5	NAP1L5	0.39	7.05E-10	0.43	5.03E-12
228067_at	chromosome 2 open reading frame 55	C2orf55	0.19	8.46E-13	0.42	1.91E-10
228069_at	family with sequence similarity 54, member A	FAM54A	6.05	8.13E-14	2.42	1.70E-06
228071_at	GTPase, IMAP family member 7	GIMAP7	0.15	1.66E-13	0.26	5.47E-13
228094_at	adhesion molecule, interacts with CXADR antigen 1	AMICA1	0.12	9.46E-19	0.34	4.63E-11
228097_at	myosin regulatory light chain interacting protein	MYLIP	0.27	3.23E-11	0.38	6.15E-09
228141_at	glutathione peroxidase 8 (putative)	GPX8	2.16	6.86E-07	2.09	2.15E-06
228143_at	ceruloplasmin (ferroxidase)	CP	2.44	1.67E-03	9.32	1.48E-11
228153_at	ring finger protein 144B	RNF144B	0.29	7.68E-16	0.40	1.39E-13
228174_at	suppressor of cancer cell invasion	SCAI	0.19	6.47E-18	0.26	2.18E-16
228185_at	zinc finger protein 25	ZNF25	0.42	1.24E-12	0.48	1.02E-10
228186_s_at	R-spondin 3	RSPO3	0.33	5.51E-06	0.21	2.07E-10
228218_at	limbic system-associated membrane protein	LSAMP	0.27	1.26E-10	0.27	2.97E-11
228232_s_at	V-set and immunoglobulin domain containing 2	VSIG2	0.14	5.83E-17	0.33	3.11E-07
228255_at	transmembrane protein 237	TMEM237	0.49	2.73E-05	0.30	3.44E-11
228263_at	GRP1 (general receptor for phosphoinositides 1)-associated scaffold protein	GRASP	0.36	7.21E-17	0.37	8.53E-18
228268_at	flavin containing monooxygenase 2 (non-functional)	FMO2	0.05	1.67E-19	0.10	1.13E-16
228273_at	proline rich 11	PRR11	10.84	1.99E-21	2.87	6.15E-09
228286_at	Gen endonuclease homolog 1 (Drosophila)	GEN1	2.41	1.71E-08	2.02	2.06E-08
228293_at	DEP domain containing 7	DEPDC7	4.55	2.53E-12	3.37	3.24E-09
228311_at	B-cell CLL/lymphoma 6, member B	BCL6B	0.22	5.14E-15	0.25	8.13E-17
228323_at	cancer susceptibility candidate 5	CASC5	12.75	4.46E-19	3.13	1.21E-07
228325_at	KIAA0146	KIAA0146	0.16	3.21E-14	0.21	6.39E-13
228333_at	zinc finger E-box binding homeobox 2	ZEB2	0.19	2.47E-15	0.33	2.30E-10
228335_at	claudin 11	CLDN11	0.26	5.27E-10	0.20	3.14E-13
228339_at	endothelial cell surface expressed chemotaxis and apoptosis regulator	ECSCR	0.12	3.19E-19	0.18	5.04E-18
228347_at	SIX homeobox 1	SIX1	22.56	1.26E-16	9.88	1.95E-12
228368_at	Rho GTPase activating protein 20	ARHGAP20	0.31	3.03E-07	0.44	3.43E-05
228372_at	chromosome 10 open reading frame 128	C10orf128	0.13	2.86E-16	0.37	2.20E-10
228376_at	glycoprotein, alpha-galactosyltransferase 1 pseudogene	GGTA1P	0.18	1.41E-10	0.46	2.58E-07
228396_at	protein kinase, cGMP-dependent, type I	PRKG1	0.41	3.38E-10	0.46	4.98E-09
228411_at	par-3 partitioning defective 3 homolog B (C. elegans)	PARD3B	0.24	9.98E-15	0.38	1.49E-14
228434_at	butyrophilin-like 9	BTNL9	0.04	8.00E-20	0.05	3.81E-20
228456_s_at	CDP-diacylglycerol synthase (phosphatidate cytidylyltransferase) 2	CDS2	0.47	1.52E-10	0.43	5.79E-15
228480_at	VAMP (vesicle-associated membrane protein)-associated protein A, 33kDa	VAPA	0.11	8.80E-21	0.22	1.53E-11
228486_at	solute carrier family 44, member 1	SLC44A1	0.39	1.96E-10	0.48	2.38E-09
228496_s_at	Cysteine rich transmembrane BMP regulator 1 (chordin-like)	CRIM1	0.46	1.12E-07	0.45	1.28E-13
228504_at	sodium channel, voltage-gated, type VII, alpha subunit	SCN7A	0.03	1.13E-19	0.15	2.57E-10
228523_at	nanos homolog 1 (Drosophila)	NANOS1	2.63	5.99E-06	2.66	1.46E-08
228532_at	chromosome 1 open reading frame 162	C1orf162	0.10	1.27E-15	0.37	9.38E-11

Probeset ID	Gene title	Gene symbol	ESP (GSE18842) Fold change	ESP (GSE18842) Adjusted P	TWN (GSE19804) Fold change	TWN (GSE19804) Adjusted P
228548_at	RAP1A, member of RAS oncogene family	RAP1A	0.20	7.00E-19	0.23	1.35E-15
228597_at	MIS18 kinetochore protein homolog A (S. pombe)	MIS18A	3.22	8.03E-16	2.00	1.28E-09
228603_at	ARP3 actin-related protein 3 homolog (yeast)	ACTR3	0.37	6.55E-14	0.38	1.32E-11
228618_at	platelet endothelial aggregation receptor 1	PEAR1	0.28	7.25E-17	0.15	2.52E-19
228653_at	sterile alpha motif domain containing 5	SAMD5	0.41	3.23E-06	0.31	3.79E-09
228665_at	cysteine/tyrosine-rich 1	CYYR1	0.13	3.90E-21	0.20	1.96E-16
228692_at	phosphatidylinositol-3,4,5-trisphosphate-dependent Rac exchange factor 2	PREX2	0.23	1.64E-13	0.25	5.04E-11
228693_at	coiled-coil domain containing 50	CCDC50	0.17	8.17E-15	0.22	4.25E-14
228697_at	histidine triad nucleotide binding protein 3	HINT3	0.27	3.45E-14	0.44	3.57E-06
228698_at	SRY (sex determining region Y)-box 7	SOX7	0.13	3.59E-12	0.10	8.24E-18
228709_at	translocated promoter region, nuclear basket protein	TPR	0.45	1.19E-08	0.41	7.49E-11
228716_at	thyroid hormone receptor, beta	THRB	0.44	5.82E-09	0.49	1.02E-06
228728_at	cadherin-like and PC-esterase domain containing 1	CPED1	0.16	2.23E-14	0.30	8.93E-13
228731_at	guanylate cyclase 1, soluble, alpha 2	GUCY1A2	0.19	1.09E-14	0.21	1.35E-12
228732_at	guanylate cyclase 1, soluble, alpha 2	GUCY1A2	0.43	1.44E-09	0.31	3.08E-12
228737_at	TOX high mobility group box family member 2	TOX2	0.26	5.90E-10	0.18	1.18E-13
228739_at	cystin 1	CYS1	0.26	7.45E-11	0.33	2.11E-08
228748_at	CD59 molecule, complement regulatory protein	CD59	0.43	6.07E-08	0.49	3.80E-08
228766_at	CD36 molecule (thrombospondin receptor)	CD36	0.04	1.46E-19	0.06	1.15E-16
228782_at	secretoglobin, family 3A, member 2	SCGB3A2	0.05	4.10E-09	0.18	2.24E-04
228793_at	jumonji domain containing 1C	JMJD1C	0.15	1.41E-13	0.48	1.02E-06
228827_at	runt-related transcription factor 1; translocated to, 1 (cyclin D-related)	RUNX1T1	0.26	1.09E-09	0.29	4.29E-09
228850_s_at	slit homolog 2 (Drosophila)	SLIT2	0.16	6.46E-17	0.21	4.26E-14
228863_at	protocadherin 17	PCDH17	0.34	2.53E-11	0.24	2.18E-14
228875_at	family with sequence similarity 162, member B	FAM162B	0.12	4.45E-18	0.20	1.34E-14
228885_at	MAM domain containing 2	MAMDC2	0.03	1.98E-17	0.11	4.33E-12
228890_at	atonal homolog 8 (Drosophila)	ATOH8	0.10	2.94E-18	0.18	1.00E-13
228905_at	pericentriolar material 1	PCM1	0.36	5.22E-13	0.36	4.32E-11
228915_at	dachshund homolog 1 (Drosophila)	DACH1	0.11	1.77E-15	0.09	2.68E-15
228962_at	phosphodiesterase 4D, cAMP-specific	PDE4D	0.31	2.10E-12	0.36	6.15E-09
228967_at	eukaryotic translation initiation factor 1	EIF1	0.21	2.17E-21	0.24	5.11E-15
229019_at	zinc finger protein 385B	ZNF385B	0.06	6.43E-17	0.34	3.86E-05
229070_at	androgen-dependent TFPI-regulating protein	ADTRP	0.10	1.84E-16	0.27	2.00E-08
229127_at	junctional adhesion molecule 2	JAM2	0.09	3.58E-20	0.13	4.35E-17
229147_at	Ras association (RalGDS/AF-6) domain family member 6	RASSF6	2.57	5.09E-05	2.37	6.98E-07
229172_at	heat shock 70kD protein 12B	HSPA12B	0.19	2.75E-15	0.15	2.51E-18
229222_at	acyl-CoA synthetase short-chain family member 3	ACSS3	0.23	1.15E-14	0.31	1.86E-10
229256_at	phosphoglucomutase 2-like 1	PGM2L1	2.30	8.23E-08	2.81	5.77E-10
229292_at	erythrocyte membrane protein band 4.1 like 5	EPB41L5	0.38	5.78E-07	0.49	3.16E-05
229302_at	transmembrane protein 178A	TMEM178A	0.15	5.80E-12	0.15	3.91E-15
229308_at	ankyrin repeat domain 29	ANKRD29	0.04	1.24E-21	0.08	1.21E-15
229309_at	adrenoceptor beta 1	ADRB1	0.05	4.51E-18	0.06	3.70E-14
229377_at	growth hormone regulated TBC protein 1	GRTP1	4.09	6.01E-13	3.82	1.60E-11
229427_at	sema domain, seven thrombospondin repeats (type 1 and type 1-like), transmembrane domain (TM) and short cytoplasmic domain, (semaphorin) 5A	SEMA5A	0.40	2.62E-06	0.17	1.45E-16
229461_x_at	neuronal growth regulator 1	NEGR1	0.19	1.28E-13	0.30	2.91E-08
229518_at	family with sequence similarity 46, member B	FAM46B	0.19	7.69E-12	0.13	2.31E-16
229553_at	phosphoglucomutase 2-like 1	PGM2L1	3.69	3.21E-14	4.03	6.85E-15

Probeset ID	Gene title	Gene symbol	ESP (GSE18842) Fold change	ESP (GSE18842) Adjusted P	TWN (GSE19804) Fold change	TWN (GSE19804) Adjusted P
229584_at	leucine-rich repeat kinase 2	LRRK2	0.03	1.08E-15	0.14	3.38E-09
229723_at	T-cell activation RhoGTPase activating protein	TAGAP	0.32	2.35E-07	0.46	3.46E-05
229779_at	collagen, type IV, alpha 4	COL4A4	0.15	2.60E-14	0.38	2.04E-07
229797_at	mucolipin 3	MCOLN3	0.30	1.87E-07	0.28	1.21E-09
229802_at	WNT1 inducible signaling pathway protein 1	WISP1	12.22	9.39E-15	4.43	1.39E-08
229844_at	forkhead box P1	FOXP1	0.28	1.43E-16	0.38	3.73E-17
229886_at	chromosome 5 open reading frame 34	C5orf34	3.32	2.31E-13	2.34	1.74E-09
229887_at	ALS2 C-terminal like	ALS2CL	0.38	1.31E-13	0.47	1.81E-14
229893_at	FERM domain containing 3	FRMD3	0.19	7.99E-16	0.11	4.36E-20
229902_at	fms-related tyrosine kinase 4	FLT4	0.43	3.81E-11	0.31	2.12E-13
229910_at	Src homology 2 domain containing E	SHE	0.12	1.18E-13	0.43	2.12E-06
230008_at	thrombospondin, type I, domain containing 7A	THSD7A	0.29	1.63E-10	0.38	7.79E-09
230036_at	sterile alpha motif domain containing 9-like	SAMD9L	0.27	7.93E-10	0.45	6.31E-07
230061_at	transmembrane 4 L six family member 18	TM4SF18	0.16	7.57E-13	0.42	5.54E-06
230104_s_at	tubulin polymerization promoting protein	TPPP	0.19	1.22E-11	0.26	1.40E-08
230130_at	Slit homolog 2 (Drosophila)	SLIT2	0.16	2.33E-14	0.16	1.77E-13
230151_at	SPRY domain containing 7	SPRYD7	0.33	1.11E-10	0.27	8.20E-16
230250_at	protein tyrosine phosphatase, receptor type, B	PTPRB	0.06	4.41E-20	0.12	1.58E-17
230252_at	lysophosphatidic acid receptor 5	LPAR5	2.69	1.59E-06	2.00	2.06E-07
230318_at	serpin peptidase inhibitor, clade A (alpha-1 antiproteinase, antitrypsin), member 1	SERPINA1	0.27	1.27E-04	0.42	1.19E-03
230360_at	gliomedin	GLDN	0.05	9.77E-20	0.34	1.38E-06
230372_at	hyaluronan synthase 2	HAS2	0.33	6.30E-07	0.48	4.68E-04
230403_at	regulatory factor X, 3 (influences HLA class II expression)	RFX3	0.45	1.84E-08	0.49	1.47E-09
230472_at	iroquois homeobox 1	IRX1	0.32	2.72E-05	0.39	3.79E-05
230479_at	eukaryotic translation initiation factor 3, subunit F	EIF3F	0.40	3.35E-13	0.43	1.19E-13
230482_at	ST6 (alpha-N-acetyl-neuraminyl-2,3-beta-galactosyl-1,3)-N-acetylgalactosaminide alpha-2,6-sialyltransferase 5	ST6GALNAC5	0.25	5.24E-11	0.10	3.34E-18
230560_at	syntaxin binding protein 6 (amisyn)	STXBP6	0.05	1.78E-16	0.04	6.69E-18
230636_s_at	Kruppel-like factor 9	KLF9	0.22	1.08E-15	0.27	1.25E-12
230645_at	FERM domain containing 3	FRMD3	0.10	6.30E-17	0.10	3.38E-18
230708_at	prickle homolog 1 (Drosophila)	PRICKLE1	0.45	2.12E-06	0.38	4.48E-08
230720_at	ring finger protein 182	RNF182	0.31	1.92E-08	0.17	1.94E-15
230730_at	sarcoglycan, delta (35kDa dystrophin-associated glycoprotein)	SGCD	0.49	3.32E-05	0.47	3.75E-06
230748_at	solute carrier family 16, member 6 (monocarboxylic acid transporter 7)	SLC16A6	0.29	6.73E-08	0.34	4.08E-11
230867_at	collagen, type VI, alpha 6	COL6A6	0.07	7.42E-19	0.09	3.52E-14
230933_at	Destrin (actin depolymerizing factor)	DSTN	0.40	5.26E-16	0.41	1.20E-10
230943_at	SRY (sex determining region Y)-box 17	SOX17	0.09	1.12E-17	0.13	4.35E-17
230951_at	erythrocyte membrane protein band 4.1 like 5	EPB41L5	0.22	1.90E-10	0.33	4.61E-07
230992_at	butyrophilin-like 9	BTNL9	0.38	1.35E-07	0.42	4.19E-07
231001_at	fin bud initiation factor homolog (zebrafish)	FIBIN	0.29	2.89E-07	0.13	1.42E-17
231240_at	deiodinase, iodothyronine, type II	DIO2	6.12	5.73E-10	2.99	8.34E-06
231411_at	Lipoma HMGIC fusion partner	LHFP	0.35	2.46E-16	0.35	2.41E-13
231810_at	BRI3 binding protein	BRI3BP	3.39	8.23E-15	2.11	8.77E-12
231842_at	KIAA1462	KIAA1462	0.30	1.22E-09	0.17	1.80E-16
231887_s_at	KIAA1274	KIAA1274	0.31	3.08E-14	0.33	2.34E-13
231899_at	zinc finger CCCH-type containing 12C	ZC3H12C	0.39	1.08E-11	0.34	3.84E-16
231945_at	filamin A interacting protein 1	FILIP1	0.33	7.03E-09	0.34	1.03E-08
231947_at	myc target 1	MYCT1	0.14	6.04E-21	0.16	9.87E-18
231991_at	chromosome 20 open reading frame 160	C20orf160	0.11	1.95E-20	0.12	4.00E-19
232056_at	sciellin	SCEL	0.09	4.56E-13	0.14	2.19E-09
232060_at	receptor tyrosine kinase-like orphan receptor 1	ROR1	0.17	1.32E-12	0.37	3.85E-10

Probeset ID	Gene title	Gene symbol	ESP (GSE18842) Fold change	Adjusted P	TWN (GSE19804) Fold change	Adjusted P
232065_x_at	centromere protein L	CENPL	2.77	8.58E-12	2.25	8.61E-11
232068_s_at	toll-like receptor 4	TLR4	0.23	8.68E-14	0.23	5.92E-12
232080_at	HECT, C2 and WW domain containing E3 ubiquitin protein ligase 2	HECW2	0.29	1.93E-12	0.27	9.42E-13
232122_s_at	ventricular zone expressed PH domain homolog 1 (zebrafish)	VEPH1	0.02	1.38E-23	0.12	4.15E-10
232204_at	early B-cell factor 1	EBF1	0.48	3.19E-05	0.33	3.33E-11
232231_at	runt-related transcription factor 2	RUNX2	2.88	1.60E-10	2.69	6.31E-10
232235_at	dermatan sulfate epimerase-like	DSEL	0.44	4.82E-06	0.41	3.89E-08
232267_at	G protein-coupled receptor 133	GPR133	0.08	1.17E-14	0.23	2.76E-09
232458_at	Collagen, type III, alpha 1	COL3A1	2.25	1.03E-03	5.91	3.03E-11
232578_at	claudin 18	CLDN18	0.00	4.14E-23	0.02	7.40E-15
232761_at	cytochrome c oxidase subunit IV isoform 2 (lung)	COX4I2	0.19	1.00E-19	0.24	4.06E-19
233252_s_at	spermatid perinuclear RNA binding protein	STRBP	2.75	6.38E-11	2.16	2.00E-14
233337_s_at	seizure related 6 homolog (mouse)-like 2	SEZ6L2	2.50	5.14E-07	4.77	2.14E-13
233496_s_at	cofilin 2 (muscle)	CFL2	0.44	2.28E-12	0.32	1.95E-10
233903_s_at	Rho guanine nucleotide exchange factor (GEF) 26	ARHGEF26	0.25	4.36E-11	0.10	8.98E-18
234725_s_at	sema domain, immunoglobulin domain (Ig), transmembrane domain (TM) and short cytoplasmic domain, (semaphorin) 4B	SEMA4B	4.76	1.58E-12	2.15	5.33E-10
234996_at	calcitonin receptor-like	CALCRL	0.16	7.81E-18	0.14	7.17E-17
234998_at	RAB11A, member RAS oncogene family	RAB11A	0.32	5.33E-12	0.32	5.46E-15
235019_at	carboxypeptidase M	CPM	0.12	8.23E-13	0.48	2.77E-05
235044_at	cysteine/tyrosine-rich 1	CYYR1	0.16	2.64E-17	0.19	4.62E-15
235051_at	coiled-coil domain containing 50	CCDC50	0.33	1.69E-10	0.28	1.65E-13
235086_at	thrombospondin 1	THBS1	0.35	2.12E-06	0.38	5.11E-05
235108_at	potassium channel, subfamily K, member 3	KCNK3	0.10	5.16E-19	0.09	1.53E-20
235131_at	ras homolog family member J	RHOJ	0.32	1.76E-11	0.31	1.32E-11
235148_at	keratinocyte associated protein 3	KRTCAP3	2.36	1.28E-07	2.50	2.04E-10
235165_at	par-6 partitioning defective 6 homolog beta (C. elegans)	PARD6B	0.24	5.03E-13	0.45	3.03E-07
235173_at	uncharacterized LOC401093	LOC401093	0.44	1.22E-08	0.32	4.80E-15
235199_at	ring finger protein 125, E3 ubiquitin protein ligase	RNF125	0.26	5.31E-12	0.38	2.05E-10
235210_s_at	somatomedin B and thrombospondin, type 1 domain containing	SBSPON	0.33	4.82E-05	0.19	2.68E-09
235228_at	coiled-coil domain containing 85A	CCDC85A	0.12	9.13E-16	0.06	6.66E-20
235279_at	parvin, alpha	PARVA	0.44	4.65E-10	0.50	5.26E-13
235301_at	KIAA1324-like	KIAA1324L	0.24	1.25E-14	0.26	2.60E-09
235306_at	GTPase, IMAP family member 8	GIMAP8	0.11	1.28E-15	0.17	6.29E-17
235320_at	ADP-ribosylation factor-like 6	ARL6	0.43	3.06E-11	0.39	1.71E-12
235324_at	serine/arginine-rich splicing factor 3	SRSF3	0.38	4.64E-13	0.49	2.21E-12
235427_at	CASP8 and FADD-like apoptosis regulator	CFLAR	0.15	1.38E-17	0.33	4.06E-12
235489_at	ras homolog family member J	RHOJ	0.12	1.59E-16	0.14	7.47E-16
235549_at	ring finger protein 144B	RNF144B	0.39	7.43E-12	0.38	1.12E-10
235561_at	thioredoxin-like 1	TXNL1	0.26	2.92E-13	0.20	6.75E-16
235568_at	chromosome 19 open reading frame 59	C19orf59	0.02	2.18E-21	0.06	8.81E-15
235570_at	RNA binding motif, single stranded interacting protein 3	RBMS3	0.14	4.96E-16	0.31	3.88E-11
235593_at	zinc finger E-box binding homeobox 2	ZEB2	0.21	9.16E-14	0.39	2.06E-10
235651_at	tetratricopeptide repeat domain 22	TTC22	3.83	2.84E-09	2.43	3.89E-08
235666_at	integrin, alpha 8	ITGA8	0.08	6.34E-17	0.21	1.97E-13
235670_at	syntaxin 11	STX11	0.07	6.38E-20	0.06	4.13E-20
235867_at	glutathione S-transferase mu 3 (brain)	GSTM3	0.37	4.73E-09	0.39	2.31E-11
235885_at	purinergic receptor P2Y, G-protein coupled, 12	P2RY12	0.22	1.89E-16	0.38	4.40E-07
235979_at	complement component 7	C7	0.24	5.51E-13	0.38	5.13E-09

Probeset ID	Gene title	Gene symbol	ESP (GSE18842) Fold change	ESP (GSE18842) Adjusted P	TWN (GSE19804) Fold change	TWN (GSE19804) Adjusted P
236029_at	FAT tumor suppressor homolog 3 (Drosophila)	FAT3	0.17	1.45E-11	0.13	4.66E-15
236262_at	multimerin 2	MMRN2	0.16	4.99E-14	0.14	2.10E-17
236313_at	cyclin-dependent kinase inhibitor 2B (p15, inhibits CDK4)	CDKN2B	0.21	1.22E-09	0.24	1.82E-10
236335_at	guanylate cyclase 1, soluble, alpha 2	GUCY1A2	0.19	2.25E-14	0.23	1.90E-11
236600_at	spastic paraplegia 20 (Troyer syndrome)	SPG20	0.35	2.36E-13	0.35	1.52E-13
236892_s_at	HOXB cluster antisense RNA 3 (non-protein coding)	HOXB-AS3	0.30	6.32E-09	0.31	2.08E-11
237252_at	thrombomodulin	THBD	0.31	1.18E-07	0.15	1.20E-16
238018_at	family with sequence similarity 150, member B	FAM150B	0.04	2.39E-25	0.07	6.45E-17
238066_at	retinol binding protein 7, cellular	RBP7	0.35	5.16E-06	0.36	5.59E-09
238075_at	checkpoint kinase 1	CHEK1	8.00	7.46E-19	2.73	1.19E-07
238222_at	gastrokine 2	GKN2	0.01	5.54E-23	0.03	1.29E-13
238332_at	ankyrin repeat domain 29	ANKRD29	0.09	7.59E-15	0.09	2.14E-16
238429_at	transmembrane protein 71	TMEM71	0.19	1.90E-11	0.39	2.06E-05
238439_at	ankyrin repeat domain 22	ANKRD22	7.34	2.04E-11	8.05	1.01E-13
238447_at	RNA binding motif, single stranded interacting protein 3	RBMS3	0.17	6.67E-17	0.39	4.60E-09
238458_at	EF-hand domain family, member A2	EFHA2	0.45	1.86E-11	0.34	1.68E-11
238472_at	F-box protein 9	FBXO9	0.26	8.16E-16	0.49	3.11E-07
238505_at	ADP-ribosylarginine hydrolase	ADPRH	0.43	5.91E-10	0.47	5.99E-10
238567_at	sphingosine-1-phosphate phosphatase 2	SGPP2	5.86	1.19E-09	5.29	2.12E-09
238587_at	ubiquitin associated and SH3 domain containing B	UBASH3B	0.31	3.80E-11	0.42	1.76E-11
238725_at	interferon regulatory factor 1	IRF1	0.27	1.95E-08	0.32	3.56E-10
238868_at	uveal autoantigen with coiled-coil domains and ankyrin repeats	UACA	0.33	1.08E-09	0.21	9.36E-14
238906_s_at	ras homolog family member J	RHOJ	0.16	1.32E-15	0.12	2.06E-16
238909_at	S100 calcium binding protein A10	S100A10	0.08	1.03E-17	0.21	7.95E-13
238996_x_at	aldolase A, fructose-bisphosphate	ALDOA	2.44	7.05E-17	2.02	3.62E-11
239135_at	calcineurin-like phosphoesterase domain containing 1	CPPED1	0.34	9.77E-20	0.37	2.72E-13
239146_at	claudin domain containing 1	CLDND1	0.31	2.70E-15	0.30	1.02E-15
239148_at	MARVEL domain containing 3	MARVELD 3	3.05	4.67E-12	2.49	1.06E-13
239155_at	coxsackie virus and adenovirus receptor	CXADR	3.19	1.40E-07	2.28	1.43E-06
239196_at	ankyrin repeat domain 22	ANKRD22	6.53	3.99E-12	5.44	1.73E-13
239272_at	matrix metallopeptidase 28	MMP28	0.10	7.58E-17	0.39	3.99E-05
239349_at	C1q and tumor necrosis factor related protein 7	C1QTNF7	0.10	4.83E-18	0.19	5.45E-11
239433_at	leucine rich repeat containing 8 family, member E	LRRC8E	2.85	1.38E-06	2.32	6.91E-08
239492_at	SEC14-like 4 (S. cerevisiae)	SEC14L4	0.22	4.03E-10	0.30	3.81E-09
239650_at	NCK-associated protein 5	NCKAP5	0.10	1.82E-16	0.05	1.89E-21
240715_at	T-box 5	TBX5	0.23	4.25E-14	0.35	1.07E-09
241396_at	neural precursor cell expressed, developmentally down-regulated 4-like, E3 ubiquitin protein ligase	NEDD4L	0.29	7.35E-10	0.29	2.48E-07
241436_at	sodium channel, non-voltage-gated 1, gamma subunit	SCNN1G	0.23	1.35E-07	0.21	2.62E-09
241765_at	carboxypeptidase M	CPM	0.17	1.21E-12	0.41	2.45E-07
241782_at	nebulette	NEBL	0.12	5.45E-20	0.12	4.46E-17
241789_at	RNA binding motif, single stranded interacting protein 3	RBMS3	0.17	4.45E-18	0.33	2.08E-09
241926_s_at	v-ets erythroblastosis virus E26 oncogene homolog (avian)	ERG	0.26	1.50E-14	0.21	1.21E-14
241981_at	family with sequence similarity 20, member A	FAM20A	0.23	1.40E-09	0.41	2.22E-06
242268_at	CUGBP, Elav-like family member 2	CELF2	0.18	1.88E-16	0.40	1.12E-05
242283_at	dynein, axonemal, heavy chain 14	DNAH14	6.38	2.46E-16	4.50	8.46E-15
242290_at	transforming, acidic coiled-coil containing protein	TACC1	0.31	4.32E-10	0.45	6.29E-08

Probeset ID	Gene title	Gene symbol	ESP (GSE18842) Fold change	ESP (GSE18842) Adjusted P	TWN (GSE19804) Fold change	TWN (GSE19804) Adjusted P
	1					
242388_x_at	T-cell activation RhoGTPase activating protein	TAGAP	0.46	5.03E-07	0.44	1.88E-06
242722_at	LIM domain 7	LMO7	0.18	1.48E-12	0.36	7.46E-10
242828_at	fidgetin	FIGN	0.49	3.87E-05	0.45	5.30E-07
242851_at	KIAA1919	KIAA1919	2.06	4.07E-09	2.00	4.16E-09
242913_at	chloride intracellular channel 6	CLIC6	2.04	3.66E-04	3.25	2.46E-08
242945_at	family with sequence similarity 20, member A	FAM20A	0.21	1.70E-10	0.35	2.41E-07
242963_at	sphingomyelin synthase 2	SGMS2	0.21	5.66E-10	0.44	2.46E-05
243141_at	sphingomyelin synthase 2	SGMS2	0.19	3.06E-11	0.41	3.62E-05
243296_at	Nicotinamide phosphoribosyltransferase	NAMPT	0.15	2.79E-11	0.25	3.16E-09
243481_at	ras homolog family member J	RHOJ	0.18	3.12E-17	0.18	4.60E-16
243501_at	ATP synthase, H+ transporting, mitochondrial Fo complex, subunit B1	ATP5F1	0.43	5.48E-12	0.46	1.21E-12
243661_at	zinc finger protein 273	ZNF273	3.11	8.97E-11	2.12	2.29E-07
243681_at	SH3 and multiple ankyrin repeat domains 2	SHANK2	0.25	3.37E-14	0.29	6.99E-10
244317_at	KIAA1324-like	KIAA1324L	0.41	2.02E-08	0.39	1.37E-05
244455_at	potassium channel, subfamily T, member 2	KCNT2	0.08	5.96E-18	0.10	9.08E-19
244546_at	cytochrome c, somatic	CYCS	0.39	7.73E-08	0.49	1.12E-06
244650_at	family with sequence similarity 105, member A	FAM105A	0.24	2.31E-12	0.34	9.18E-16
244741_s_at	uncharacterized LOC100128252	LOC100128252	0.32	2.78E-06	0.41	2.65E-06
244745_at	RAS-like, estrogen-regulated, growth inhibitor	RERG	0.23	4.46E-13	0.32	4.67E-08
244774_at	phosphatase and actin regulator 2	PHACTR2	0.34	3.12E-12	0.44	7.51E-07
244779_at	zinc finger, DHHC-type containing 2	ZDHHC2	0.37	6.26E-09	0.41	1.36E-08
244780_at	sphingosine-1-phosphate phosphatase 2	SGPP2	2.07	7.42E-03	6.86	1.96E-08
266_s_at	CD24 molecule	CD24	3.66	1.82E-08	3.79	1.80E-10
33322_i_at	stratifin	SFN	3.53	2.09E-06	3.41	6.24E-10
33323_r_at	stratifin	SFN	3.52	1.11E-05	4.06	8.13E-10
34210_at	CD52 molecule	CD52	0.08	1.13E-18	0.35	3.41E-09
36711_at	v-maf musculoaponeurotic fibrosarcoma oncogene homolog F (avian)	MAFF	0.32	2.12E-10	0.27	3.42E-14
36829_at	period homolog 1 (Drosophila)	PER1	0.34	1.69E-10	0.36	5.91E-09
36936_at	tissue specific transplantation antigen P35B	TSTA3	2.00	4.05E-07	2.40	7.44E-13
37004_at	surfactant protein B	SFTPB	0.08	1.80E-06	0.40	4.59E-03
37028_at	protein phosphatase 1, regulatory subunit 15A	PPP1R15A	0.20	3.66E-16	0.23	1.07E-18
37512_at	hydroxysteroid (17-beta) dehydrogenase 6 homolog (mouse)	HSD17B6	0.07	1.56E-17	0.24	1.47E-05
38037_at	heparin-binding EGF-like growth factor	HBEGF	0.16	1.55E-11	0.10	3.35E-16
38149_at	Rho GTPase activating protein 25	ARHGAP25	0.30	5.49E-13	0.37	1.49E-09
38691_s_at	surfactant protein C	SFTPC	0.01	3.61E-13	0.10	3.43E-07
39402_at	interleukin 1, beta	IL1B	0.22	7.93E-08	0.27	1.95E-08
396_f_at	erythropoietin receptor	EPOR	0.41	7.27E-15	0.49	3.67E-12
40560_at	T-box 2	TBX2	0.21	4.90E-15	0.24	5.58E-12
40665_at	flavin containing monooxygenase 3	FMO3	0.31	1.27E-07	0.36	2.55E-07
40687_at	gap junction protein, alpha 4, 37kDa	GJA4	0.25	4.92E-15	0.32	8.40E-16
41386_i_at	lysine (K)-specific demethylase 6B	KDM6B	0.23	5.56E-13	0.45	7.94E-09
41577_at	protein phosphatase 1, regulatory subunit 16B	PPP1R16B	0.23	2.15E-12	0.27	2.51E-13
41644_at	SAM and SH3 domain containing 1	SASH1	0.30	4.18E-15	0.25	8.55E-18
43511_s_at	arrestin, beta 1	ARRB1	0.11	1.58E-20	0.20	9.53E-20
45288_at	abhydrolase domain containing 6	ABHD6	0.37	2.15E-11	0.37	1.97E-14
45297_at	EH-domain containing 2	EHD2	0.44	9.79E-10	0.44	1.14E-11
45749_at	family with sequence similarity 65, member A	FAM65A	0.37	4.34E-14	0.41	3.08E-19
49111_at	arrestin, beta 1	ARRB1	0.12	4.37E-19	0.18	4.16E-20
53991_at	DENN/MADD domain containing 2A	DENND2A	0.26	1.33E-16	0.27	4.57E-15
57588_at	solute carrier family 24	SLC24A3	0.46	9.34E-06	0.43	1.62E-07

Probeset ID	Gene title	Gene symbol	ESP (GSE18842) Fold change	ESP (GSE18842) Adjusted P	TWN (GSE19804) Fold change	TWN (GSE19804) Adjusted P
	(sodium/potassium/calcium exchanger), member 3					
60474_at	fermitin family member 1	FERMT1	18.49	8.85E-20	6.05	1.65E-13
61297_at	CASK interacting protein 2	CASKIN2	0.32	8.65E-13	0.26	6.43E-20
61734_at	reticulocalbin 3, EF-hand calcium binding domain	RCN3	2.76	1.08E-08	2.30	3.02E-09
63305_at	PBX/knotted 1 homeobox 2	PKNOX2	0.25	1.82E-16	0.18	4.00E-19
823_at	chemokine (C-X3-C motif) ligand 1	CX3CL1	0.47	4.80E-04	0.32	5.99E-09

Table S3: The overlap between the NFE2L2 associated genes and the dysregulated genes in lung tumor tissues.

Probeset ID	Gene title	Gene symbol
213664_at	solute carrier family 1 (neuronal/epithelial high affinity glutamate transporter, system Xag), member 1	SLC1A1
217287_s_at	transient receptor potential cation channel, subfamily C, member 6	TRPC6
209821_at	interleukin 33	IL33
224189_x_at	ets homologous factor	EHF
227948_at	FYVE, RhoGEF and PH domain containing 4	FGD4
225207_at	pyruvate dehydrogenase kinase, isozyme 4	PDK4
230645_at	FERM domain containing 3	FRMD3
212327_at	LIM and calponin homology domains 1	LIMCH1
221748_s_at	tensin 1	TNS1
225645_at	ets homologous factor	EHF
204619_s_at	versican	VCAN
230730_at	sarcoglycan, delta (35kDa dystrophin-associated glycoprotein)	SGCD
204622_x_at	nuclear receptor subfamily 4, group A, member 2	NR4A2
219850_s_at	ets homologous factor	EHF
205654_at	complement component 4 binding protein, alpha	C4BPA
209498_at	carcinoembryonic antigen-related cell adhesion molecule 1 (biliary glycoprotein)	CEACAM1
218313_s_at	UDP-N-acetyl-alpha-D-galactosamine:polypeptide N-acetylgalactosaminyltransferase 7	GALNT7
228748_at	CD59 molecule, complement regulatory protein	CD59
205083_at	aldehyde oxidase 1	AOX1
211571_s_at	versican	VCAN
215076_s_at	collagen, type III, alpha 1	COL3A1
230104_s_at	tubulin polymerization promoting protein	TPPP
207761_s_at	methyltransferase like 7A	METTL7A
208944_at	transforming growth factor, beta receptor II (70/80kDa)	TGFBR2
207714_s_at	serpin peptidase inhibitor, clade H (heat shock protein 47), member 1, (collagen binding protein 1)	SERPINH1
202202_s_at	laminin, alpha 4	LAMA4
212328_at	LIM and calponin homology domains 1	LIMCH1
201578_at	podocalyxin-like	PODXL
203914_x_at	hydroxyprostaglandin dehydrogenase 15-(NAD)	HPGD
218876_at	tubulin polymerization-promoting protein family member 3	TPPP3
226016_at	CD47 molecule	CD47
226145_s_at	Fraser syndrome 1	FRAS1
205433_at	butyrylcholinesterase	BCHE
218309_at	calcium/calmodulin-dependent protein kinase II inhibitor 1	CAMK2N1
211548_s_at	hydroxyprostaglandin dehydrogenase 15-(NAD)	HPGD
204797_s_at	echinoderm microtubule associated protein like 1	EML1
223218_s_at	nuclear factor of kappa light polypeptide gene enhancer in B-cells inhibitor, zeta	NFKBIZ
215646_s_at	versican	VCAN
218901_at	phospholipid scramblase 4	PLSCR4
221011_s_at	limb bud and heart development homolog (mouse)	LBH
212325_at	LIM and calponin homology domains 1	LIMCH1
219682_s_at	T-box 3	TBX3
201852_x_at	collagen, type III, alpha 1	COL3A1
222587_s_at	UDP-N-acetyl-alpha-D-galactosamine:polypeptide N-acetylgalactosaminyltransferase 7	GALNT7
204620_s_at	versican	VCAN
216623_x_at	TOX high mobility group box family member 3	TOX3
201984_s_at	epidermal growth factor receptor	EGFR
235199_at	ring finger protein 125, E3 ubiquitin protein ligase	RNF125
212444_at	G protein-coupled receptor, family C, group 5, member A	GPRC5A
219993_at	SRY (sex determining region Y)-box 17	SOX17
210095_s_at	insulin-like growth factor binding protein 3	IGFBP3
203060_s_at	3'-phosphoadenosine 5'-phosphosulfate synthase 2	PAPSS2
204326_x_at	metallothionein 1X	MT1X

Probeset ID	Gene title	Gene symbol
225299_at	myosin VB	MYO5B
225144_at	bone morphogenetic protein receptor, type II (serine/threonine kinase)	BMPR2
213894_at	thrombospondin, type I, domain containing 7A	THSD7A
230720_at	ring finger protein 182	RNF182
213675_at	parvin, alpha	PARVA
222853_at	fibronectin leucine rich transmembrane protein 3	FLRT3
221731_x_at	versican	VCAN
212143_s_at	insulin-like growth factor binding protein 3	IGFBP3
204748_at	prostaglandin-endoperoxide synthase 2 (prostaglandin G/H synthase and cyclooxygenase)	PTGS2
223217_s_at	nuclear factor of kappa light polypeptide gene enhancer in B-cells inhibitor, zeta	NFKBIZ
211161_s_at	collagen, type III, alpha 1	COL3A1
229584_at	leucine-rich repeat kinase 2	LRRK2
202388_at	regulator of G-protein signaling 2, 24kDa	RGS2
229893_at	FERM domain containing 3	FRMD3
216248_s_at	nuclear receptor subfamily 4, group A, member 2	NR4A2
211685_s_at	neurocalcin delta	NCALD
223821_s_at	sushi domain containing 4	SUSD4
204688_at	sarcoglycan, epsilon	SGCE
220327_at	vestigial like 3 (Drosophila)	VGLL3
202794_at	inositol polyphosphate-1-phosphatase	INPP1
222453_at	cytochrome b reductase 1	CYBRD1
204621_s_at	nuclear receptor subfamily 4, group A, member 2	NR4A2
1554997_a_at	prostaglandin-endoperoxide synthase 2 (prostaglandin G/H synthase and cyclooxygenase)	PTGS2
230008_at	thrombospondin, type I, domain containing 7A	THSD7A
227458_at	CD274 molecule	CD274
205407_at	reversion-inducing-cysteine-rich protein with kazal motifs	RECK
227046_at	solute carrier family 39 (metal ion transporter), member 11	SLC39A11
203058_s_at	3'-phosphoadenosine 5'-phosphosulfate synthase 2	PAPSS2
219274_at	tetraspanin 12	TSPAN12
228038_at	SRY (sex determining region Y)-box 2	SOX2
219250_s_at	fibronectin leucine rich transmembrane protein 3	FLRT3
203888_at	thrombomodulin	THBD
209772_s_at	CD24 molecule	CD24
219010_at	chromosome 1 open reading frame 106	C1orf106
204678_s_at	potassium channel, subfamily K, member 1	KCNK1
231945_at	filamin A interacting protein 1	FILIP1
227983_at	Rab interacting lysosomal protein-like 2	RILPL2
228737_at	TOX high mobility group box family member 2	TOX2
221489_s_at	sprouty homolog 4 (Drosophila)	SPRY4
223395_at	ABI family, member 3 (NESH) binding protein	ABI3BP
203887_s_at	thrombomodulin	THBD
217771_at	golgi membrane protein 1	GOLM1
218736_s_at	palmdelphin	PALMD
211549_s_at	hydroxyprostaglandin dehydrogenase 15-(NAD)	HPGD
201286_at	syndecan 1	SDC1
212488_at	collagen, type V, alpha 1	COL5A1
212190_at	serpin peptidase inhibitor, clade E (nexin, plasminogen activator inhibitor type 1), member 2	SERPINE2
228962_at	phosphodiesterase 4D, cAMP-specific	PDE4D
230403_at	regulatory factor X, 3 (influences HLA class II expression)	RFX3
217862_at	protein inhibitor of activated STAT, 1	PIAS1
212372_at	myosin, heavy chain 10, non-muscle	MYH10
204990_s_at	integrin, beta 4	ITGB4
201843_s_at	EGF containing fibulin-like extracellular matrix protein 1	EFEMP1
234998_at	RAB11A, member RAS oncogene family	RAB11A
203372_s_at	suppressor of cytokine signaling 2	SOCS2
228905_at	pericentriolar material 1	PCM1

Probeset ID	Gene title	Gene symbol
204017_at	KDEL (Lys-Asp-Glu-Leu) endoplasmic reticulum protein retention receptor 3	KDELR3
203186_s_at	S100 calcium binding protein A4	S100A4
200911_s_at	transforming, acidic coiled-coil containing protein 1	TACC1
203185_at	Ras association (RalGDS/AF-6) domain family member 2	RASSF2
227126_at	protein tyrosine phosphatase, receptor type, G	PTPRG
204519_s_at	plasmolipin	PLLP
226795_at	leucine-rich repeats and calponin homology (CH) domain containing 1	LRCH1
225237_s_at	musashi homolog 2 (Drosophila)	MSI2
205289_at	bone morphogenetic protein 2	BMP2
238868_at	uveal autoantigen with coiled-coil domains and ankyrin repeats	UACA
204719_at	ATP-binding cassette, sub-family A (ABC1), member 8	ABCA8
207069_s_at	SMAD family member 6	SMAD6
200904_at	major histocompatibility complex, class I, E	HLA-E
225946_at	Ras association (RalGDS/AF-6) domain family (N-terminal) member 8	RASSF8
205992_s_at	interleukin 15	IL15
226633_at	RAB8B, member RAS oncogene family	RAB8B
208581_x_at	metallothionein 1X	MT1X
228293_at	DEP domain containing 7	DEPDC7
202497_x_at	solute carrier family 2 (facilitated glucose transporter), member 3	SLC2A3
237252_at	thrombomodulin	THBD
203325_s_at	collagen, type V, alpha 1	COL5A1
212489_at	collagen, type V, alpha 1	COL5A1
220180_at	coiled-coil domain containing 68	CCDC68
226150_at	phosphatidic acid phosphatase type 2 domain containing 1B	PPAPDC1B
225240_s_at	musashi homolog 2 (Drosophila)	MSI2
202897_at	signal-regulatory protein alpha	SIRPA
226485_at	V-set and immunoglobulin domain containing 10	VSIG10
239650_at	NCK-associated protein 5	NCKAP5
204073_s_at	chromosome 11 open reading frame 9	C11orf9
206858_s_at	homeobox C6	HOXC6
203895_at	phospholipase C, beta 4	PLCB4
201842_s_at	EGF containing fibulin-like extracellular matrix protein 1	EFEMP1
204745_x_at	metallothionein 1G	MT1G
209189_at	FBJ murine osteosarcoma viral oncogene homolog	FOS
207808_s_at	protein S (alpha)	PROS1
227417_at	mitochondrial amidoxime reducing component 2	MARC2
204115_at	guanine nucleotide binding protein (G protein), gamma 11	GNG11
235561_at	thioredoxin-like 1	TXNL1
203035_s_at	protein inhibitor of activated STAT, 3	PIAS3
214920_at	thrombospondin, type I, domain containing 7A	THSD7A
226607_at	chromosome 20 open reading frame 194	C20orf194
201983_s_at	epidermal growth factor receptor	EGFR
202499_s_at	solute carrier family 2 (facilitated glucose transporter), member 3	SLC2A3
205609_at	angiopoietin 1	ANGPT1
230061_at	transmembrane 4 L six family member 18	TM4SF18
212427_at	KIAA0368	KIAA0368
226777_at	ADAM metallopeptidase domain 12	ADAM12
203813_s_at	slit homolog 3 (Drosophila)	SLIT3
204679_at	potassium channel, subfamily K, member 1	KCNK1
204796_at	echinoderm microtubule associated protein like 1	EML1
205952_at	potassium channel, subfamily K, member 3	KCNK3
226713_at	coiled-coil domain containing 50	CCDC50
225666_at	transmembrane and tetratricopeptide repeat containing 4	TMTC4
218546_at	chromosome 1 open reading frame 115	C1orf115
223492_s_at	leucine rich repeat (in FLII) interacting protein 1	LRRFIP1
224674_at	tweety homolog 3 (Drosophila)	TTYH3

Probeset ID	Gene title	Gene symbol
207265_s_at	KDEL (Lys-Asp-Glu-Leu) endoplasmic reticulum protein retention receptor 3	KDELR3
226198_at	target of myb1-like 2 (chicken)	TOM1L2
225387_at	tetraspanin 5	TSPAN5
227148_at	pleckstrin homology domain containing, family H (with MyTH4 domain) member 2	PLEKHH2
209292_at	inhibitor of DNA binding 4, dominant negative helix-loop-helix protein	ID4
209710_at	GATA binding protein 2	GATA2
235427_at	CASP8 and FADD-like apoptosis regulator	CFLAR
228890_at	atonal homolog 8 (Drosophila)	ATOH8
230479_at	eukaryotic translation initiation factor 3, subunit F	EIF3F
228255_at	transmembrane protein 237	TMEM237
203373_at	suppressor of cytokine signaling 2	SOCS2
238222_at	gastrokine 2	GKN2
49111_at	arrestin, beta 1	ARRB1
212636_at	QKI, KH domain containing, RNA binding	QKI
229802_at	WNT1 inducible signaling pathway protein 1	WISP1
221747_at	tensin 1	TNS1
212450_at	SECIS binding protein 2-like	SECISBP2L
227131_at	mitogen-activated protein kinase kinase kinase 3	MAP3K3
202085_at	tight junction protein 2 (zona occludens 2)	TJP2
244650_at	family with sequence similarity 105, member A	FAM105A
219694_at	family with sequence similarity 105, member A	FAM105A
228603_at	ARP3 actin-related protein 3 homolog (yeast)	ACTR3
204343_at	ATP-binding cassette, sub-family A (ABC1), member 3	ABCA3
205442_at	microfibrillar-associated protein 3-like	MFAP3L
202718_at	insulin-like growth factor binding protein 2, 36kDa	IGFBP2
226615_at	xenotropic and polytropic retrovirus receptor 1	XPR1
211330_s_at	hemochromatosis	HFE
228709_at	translocated promoter region, nuclear basket protein	TPR
225817_at	cingulin-like 1	CGNL1
239272_at	matrix metallopeptidase 28	MMP28
202995_s_at	fibulin 1	FBLN1
221276_s_at	syncoilin, intermediate filament protein	SYNC
235019_at	carboxypeptidase M	CPM
209355_s_at	phosphatidic acid phosphatase type 2B	PPAP2B
202073_at	optineurin	OPTN
226641_at	ankyrin repeat domain 44	ANKRD44
239492_at	SEC14-like 4 (S. cerevisiae)	SEC14L4
229377_at	growth hormone regulated TBC protein 1	GRTP1
203543_s_at	Kruppel-like factor 9	KLF9
215617_at	spermatogenesis associated, serine-rich 2-like	SPATS2L
204897_at	prostaglandin E receptor 4 (subtype EP4)	PTGER4
235279_at	parvin, alpha	PARVA
236335_at	guanylate cyclase 1, soluble, alpha 2	GUCY1A2
212942_s_at	KIAA1199	KIAA1199
228185_at	zinc finger protein 25	ZNF25
211776_s_at	erythrocyte membrane protein band 4.1-like 3	EPB41L3
225540_at	microtubule-associated protein 2	MAP2
205681_at	BCL2-related protein A1	BCL2A1
209921_at	solute carrier family 7 (anionic amino acid transporter light chain, xc- system), member 11	SLC7A11
223484_at	chromosome 15 open reading frame 48	C15orf48
243296_at	Nicotinamide phosphoribosyltransferase	NAMPT
203002_at	angiomotin like 2	AMOTL2
33323_r_at	stratifin	SFN
33322_i_at	stratifin	SFN
209735_at	ATP-binding cassette, sub-family G (WHITE), member 2	ABCG2
201348_at	glutathione peroxidase 3 (plasma)	GPX3

Probeset ID	Gene title	Gene symbol
209875_s_at	secreted phosphoprotein 1	SPP1
221541_at	cysteine-rich secretory protein LCCL domain containing 2	CRISPLD2
227697_at	suppressor of cytokine signaling 3	SOCS3
217678_at	solute carrier family 7 (anionic amino acid transporter light chain, xc- system), member 11	SLC7A11
227771_at	leukemia inhibitory factor receptor alpha	LIFR
228186_s_at	R-spondin 3	RSPO3
214096_s_at	serine hydroxymethyltransferase 2 (mitochondrial)	SHMT2
201858_s_at	serglycin	SRGN
212872_s_at	mediator complex subunit 20	MED20
219054_at	natriuretic peptide receptor C/guanylate cyclase C (atrionatriuretic peptide receptor C)	NPR3
204702_s_at	nuclear factor (erythroid-derived 2)-like 3	NFE2L3
227006_at	protein phosphatase 1, regulatory (inhibitor) subunit 14A	PPP1R14A
221841_s_at	Kruppel-like factor 4 (gut)	KLF4
206074_s_at	high mobility group AT-hook 1	HMGA1
201387_s_at	ubiquitin carboxyl-terminal esterase L1 (ubiquitin thiolesterase)	UCHL1
226905_at	family with sequence similarity 101, member B	FAM101B
209949_at	neutrophil cytosolic factor 2	NCF2
201467_s_at	NAD(P)H dehydrogenase, quinone 1	NQO1
225943_at	neurolysin (metallopeptidase M3 family)	NLN
225575_at	leukemia inhibitory factor receptor alpha	LIFR
231240_at	deiodinase, iodothyronine, type II	DIO2
206157_at	pentraxin 3, long	PTX3
225944_at	neurolysin (metallopeptidase M3 family)	NLN
219014_at	placenta-specific 8	PLAC8
202643_s_at	tumor necrosis factor, alpha-induced protein 3	TNFAIP3
210517_s_at	A kinase (PRKA) anchor protein 12	AKAP12
203324_s_at	caveolin 2	CAV2
203910_at	Rho GTPase activating protein 29	ARHGAP29
201797_s_at	valyl-tRNA synthetase	VARS
209433_s_at	phosphoribosyl pyrophosphate amidotransferase	PPAT
219295_s_at	procollagen C-endopeptidase enhancer 2	PCOLCE2
227530_at	A kinase (PRKA) anchor protein 12	AKAP12
223452_s_at	atlastin GTPase 3	ATL3
224480_s_at	1-acylglycerol-3-phosphate O-acyltransferase 9	AGPAT9
224833_at	v-ets erythroblastosis virus E26 oncogene homolog 1 (avian)	ETS1
201468_s_at	NAD(P)H dehydrogenase, quinone 1	NQO1
201466_s_at	jun proto-oncogene	JUN
201890_at	ribonucleotide reductase M2	RRM2
242283_at	dynein, axonemal, heavy chain 14	DNAH14
201289_at	cysteine-rich, angiogenic inducer, 61	CYR61
209773_s_at	ribonucleotide reductase M2	RRM2
207980_s_at	Cbp/p300-interacting transactivator, with Glu/Asp-rich carboxy-terminal domain, 2	CITED2
209434_s_at	phosphoribosyl pyrophosphate amidotransferase	PPAT
201563_at	sorbitol dehydrogenase	SORD
211668_s_at	plasminogen activator, urokinase	PLAU
204802_at	Ras-related associated with diabetes	RRAD
205129_at	nucleophosmin/nucleoplasmin 3	NPM3
238332_at	ankyrin repeat domain 29	ANKRD29
203323_at	caveolin 2	CAV2
218305_at	importin 4	IPO4
214091_s_at	glutathione peroxidase 3 (plasma)	GPX3
205479_s_at	plasminogen activator, urokinase	PLAU
210764_s_at	cysteine-rich, angiogenic inducer, 61	CYR61
227529_s_at	A kinase (PRKA) anchor protein 12	AKAP12
205034_at	cyclin E2	CCNE2
202644_s_at	tumor necrosis factor, alpha-induced protein 3	TNFAIP3

Probeset ID	Gene title	Gene symbol
218723_s_at	regulator of cell cycle	RGCC
202672_s_at	activating transcription factor 3	ATF3
212828_at	synaptojanin 2	SYNJ2
205876_at	leukemia inhibitory factor receptor alpha	LIFR
235165_at	par-6 partitioning defective 6 homolog beta (C. elegans)	PARD6B
218756_s_at	dehydrogenase/reductase (SDR family) member 11	DHRS11
218486_at	Kruppel-like factor 11	KLF11
227197_at	Rho guanine nucleotide exchange factor (GEF) 26	ARHGEF26
204803_s_at	Ras-related associated with diabetes	RRAD
222931_s_at	threonine synthase-like 1 (S. cerevisiae)	THNSL1
220266_s_at	Kruppel-like factor 4 (gut)	KLF4
216598_s_at	chemokine (C-C motif) ligand 2	CCL2
225571_at	leukemia inhibitory factor receptor alpha	LIFR
205993_s_at	T-box 2	TBX2
230560_at	syntaxin binding protein 6 (amisyn)	STXBP6
206359_at	suppressor of cytokine signaling 3	SOCS3
213844_at	homeobox A5	HOXA5
227247_at	pleckstrin homology domain containing, family A (phosphoinositide binding specific) member 8	PLEKHA8
219232_s_at	egl nine homolog 3 (C. elegans)	EGLN3
225328_at	F-box protein 32	FBXO32
229309_at	adrenoceptor beta 1	ADRB1
233903_s_at	Rho guanine nucleotide exchange factor (GEF) 26	ARHGEF26

Table S4. Multivariate Cox proportional hazards regression of survival in the validation cohorts with the same covariate

		Recurrence-free survival			Overall survival		
Cohort	Covariate	HR	95% CI	*P*-value	HR	95% CI	*P*-value
JPN	NAMS + vs. -	2.94	(1.64, 5.29)	3.1×10^{-4}	4.47	(1.78, 11.20)	1.4×10^{-3}
	Age (per year)	1.05	(1.01, 1.09)	1.6×10^{-2}	1.04	(0.99, 1.09)	1.2×10^{-1}
	Gender male vs. female	1.00	(0.61, 1.65)	9.9×10^{-1}	1.12	(0.57, 2.21)	7.4×10^{-1}
	Stage	2.37	(1.39, 4.03)	1.5×10^{-3}	2.79	(1.38, 5.64)	4.1×10^{-3}
SWE	NAMS + vs. -	2.72	(1.32, 5.61)	6.6×10^{-3}	1.43	(1.01, 2.04)	4.5×10^{-2}
	Age (per year)	1.00	(0.96, 1.03)	8.7×10^{-1}	1.03	(1.01, 1.05)	5.7×10^{-3}
	Gender male vs. female	0.80	(0.43, 1.48)	4.8×10^{-1}	0.98	(0.69, 1.38)	9.1×10^{-1}
	Stage	1.11	(0.75, 1.63)	6.1×10^{-1}	1.24	(1.02, 1.50)	3.3×10^{-2}
CAN	NAMS + vs. -	2.23	(1.18, 4.20)	1.3×10^{-2}	1.65	(1.00, 2.71)	4.9×10^{-2}
	Age (per year)	1.00	(0.97, 1.03)	8.4×10^{-1}	1.02	(0.99, 1.04)	1.7×10^{-1}
	Gender male vs. female	1.72	(0.97, 3.07)	6.4×10^{-2}	2.03	(1.24, 3.31)	4.5×10^{-3}
	Stage	1.80	(1.02, 3.17)	4.3×10^{-2}	1.67	(1.03, 2.72)	3.7×10^{-2}

Note – HR: hazard ratio; CI: confidence interval

Table S5. List of GEO datasets used in this study.

**Cohort**	Accession	Role
	GSE38332	Discovery of Nrf2-influenced genes
**ESP**	GSE18842	Identification the differentially expressed genes between normal and tumor lung tissues
**TWN**	GSE19804	Identification the differentially expressed genes between normal and tumor lung tissues
**KOR**	GSE8894	Discovery cohort in the recurrence-free survival study
**USA**	GSE3141	Discovery cohort in the overall survival study
**JPN**	GSE31210	Validation cohort in both recurrence-free survival and overall survival studies
**SWE**	GSE37745	Validation cohort in both recurrence-free survival and overall survival studies
**CAN**	GSE50081	Validation cohort in both recurrence-free survival and overall survival studies

Note: All of these eight microarray datasets are generated on a platform of Affymetrix GeneChip Human Genome U133 Plus 2.0 Array.
